# Upregulation of miR-181a-5p and miR-125b-2-3p in the Maternal Circulation of Fetuses with Rh-Negative Hemolytic Disease of the Fetus and Newborn Could Be Related to Dysfunction of Placental Function

**DOI:** 10.1155/2022/2594091

**Published:** 2022-09-21

**Authors:** Xiao-Hui Xie, Mei Jiang, Zheng-Ai Xiong, Xiao-Qin Huang

**Affiliations:** ^1^Department of Obstetrics and Gynecology, The First People's Hospital of Neijiang City, Neijiang, Sichuan 641000, China; ^2^Chongqing Medical University, Chongqing 400016, China; ^3^Department of Obstetrics and Gynecology, The Second Affiliated Hospital of Chongqing Medical University, Chongqing Medical University, Chongqing 400010, China

## Abstract

The transplacental transfer of maternal antibodies to the fetus is a critical mechanism for infant protection and perinatal disease. Hemolytic disease of the fetus and newborn (HDFN) is a representative fetal disease caused by transplacental transfer of maternal IgG antibodies. However, it is unclear whether placental-related miRNAs are expressed in Rh-HDFN. Through the investigation of the miR-181a-5p and miR-125b-2-3p levels in maternal plasma using qPCR, we found that both miR-181a-5p and miR-125b-2-3p were highly expressed in maternal plasma of newborns with Rh-HDFN compared with healthy controls, indicating the potential roles of these two miRNAs in Rh-HDFN. To demonstrate whether dysregulation of miR-125b-2-3p and miR-181a-5p contributes to Rh-HDFN development, we analyze the placental miRNA-/mRNA sequencing data (GSE73714) using weighted gene coexpression network analysis (WGCNA), miRNA target predictive databases, and DAVID (Database for Annotation, Visualization, and Integrated Discovery). The results showed that miR-125b-2-3p and miR-181a-5p could regulate several biological processes including cytoplasmic microtubule organization and angiogenesis. Moreover, core promoter sequence-specific DNA binding and protein binding were highly enriched molecular functions, indicating the potential roles of transcriptional regulation. Further pathway enrichment showed that miR-181a-5p and miR-125b-2-3p could regulate several biological pathways that were closely related to placental function, including the FoxO signaling pathway, focal adhesion, mTOR signaling pathway, and central carbon metabolism in cancer. In conclusion, the present results first revealed miRNA expression in the maternal circulation of newborns with Rh-HDFN, which could be caused by dysfunction of the placenta.

## 1. Introduction

The transplacental transfer of maternal IgG antibodies to the fetus is a critical mechanism for infant protection, while their humoral response is inefficient [1]. However, in some circumstances, the placental transmission of antibodies is detrimental to the neonate [2]. Hemolytic disease of the fetus and newborn (HDFN) is a representative fetal disease caused by transplacental transfer of maternal IgG antibodies, a severe complication of maternal and fetal blood group incompatibility [3]. ABO isoimmunization is the common etiology of HDFN, while Rh isoimmunization rarely occurs in the Chinese population [4]. Although it remains the leading cause of HDFN in the European population, thus far, there have been only a few publications about Rh-negative HDFN (Rh-HDFN) in Chinese patients, mainly because of ethnic specificity [4–6]. With the development of Rh-D immunoprophylaxis, the risk of Rh-HDFN was decreased. Two percent of newborns suffer from Rh-HDFN, which may cause severe complications, such as kernicterus, demise, miscarriage, or perinatal death [7]. Because sensitized maternal IgG antibodies in fetal circulation are the leading cause of Rh-HDNF, the placenta plays a central regulatory role in sensitized IgG transfer. Therefore, any biologically active molecules that affect the function of the placenta may become modulators of Rh-HDFN, including miRNAs [8].

Normal proliferation/differentiation of human placental trophoblasts contributes to the functional normality of the placenta [9]. Several studies have suggested that placental trophoblasts at the feto-maternal interface produce a broad repertoire of miRNA species, which not only have an important epigenetic role during the development of the fetus but also play a crucial role in pregnancy-related complications and infant diseases [8, 10–12]. A previous study showed that miR-181a-5p and miR-125b-2-3p were highly expressed in villous trophoblasts and some villous stromal cells [13, 14]. In addition, their results suggested that miRNAs could be secreted from human trophoblast cells into maternal plasma and that the amount of miRNA is related to the intracellular level of miRNAs. In particular, a recent study showed that overexpression of miR-181a-5p compromised the integrity of the endothelial barrier in the placenta, further indicating the critical roles of miRNA in the placental barrier [15]. However, the role of miRNAs remains unclear in Rh-HDFN. In the present study, two miRNAs, miR-181a-5p and miR-125b-2-3p, were further investigated in the plasma of pregnant women in the perinatal period to analyze their potential value in Rh-HDFN, given their functional role in the placenta and heart tissue of the fetus with Down's syndrome [11, 16]. Meanwhile, the target genes of miR-181a-5p and miR-125b-2-3p and their associated pathways were identified by the weighted correlation network analysis (WGCNA) and the DAVID (Database for Annotation, Visualization, and Integrated Discovery) database to explain the potential roles of miRNAs in Rh-HDFN.

## 2. Materials and Methods

### 2.1. General Data

We enrolled 26 Chinese pregnancies in the present study, including 13 pregnancies with Rh-HDFN newborns and 13 pregnancies with healthy newborns. They were admitted to the Second Affiliated Hospital of Chongqing Medical University and the First People's Hospital of Neijiang, Sichuan Province, from January 2016 to September 2020. These pregnancies were divided into 2 groups: the Rh-HDFN group (*n* = 13 pregnancies) and the control group (*n* = 13 pregnancies). All enrolled pregnancies were clinically diagnosed by physical examination and laboratory investigations, including routine blood tests, liver function tests, kidney function tests, and coagulated and anticoagulated blood tests from mothers and newborns according to the Chinese National Standardized Protocols for Clinical Laboratory, 4th version, polybrene tests, Coombs tests, antibodies against the Rh group (anti-D, anti-E, anti-e, anti-C, and anti-c), and the ant-A and anti-B groups. Patients' inclusion criteria were an uncomplicated pregnancy, gestational age at venipuncture between 20-42 weeks, high-risk pregnancies with Rh-HDFN newborns, and pregnancies with healthy newborns. Pregnancies' exclusion criteria were those with pregnancies with severe liver dysfunction, kidney dysfunction, severe infectious diseases, cognitive disorder, communication disorder, and pregnancies who did not cooperate with the experiment. The research ethics committee approved the research protocol for experimental and clinical studies at the Faculty of the First People's Hospital of Neijiang City, Sichuan Province, and written informed consent was obtained from all pregnancies enrolled in the study. Whole peripheral blood samples of all pregnancies were withdrawn in EDTA vacutainer tubes. All blood samples were processed at 4°C and then stored at -80°C for final miRNA extraction. The placentas were collected and placed in 5 volumes of RNAlater solution (ThermoFisher Scientific, Shanghai, China) within 15 minutes of delivery. All samples were stored at −4°C overnight and then moved to -20°C until laboratory procedures were performed.

### 2.2. miRNA Isolation

miRNA was extracted from 200 *μ*L of plasma using the miRcute serum/plasma miRNA isolation kit (Tiangen Biotech, Beijing, China) following the manufacturer's instructions. After washing miRNAs were eluted in 30 *μ*L DEPC water. The RNA quantity was assessed by NanoDrop 2000C (Thermo Scientific). The cDNA was synthesized using the miRcute miRNA First-Strand cDNA Synthesis Kit (Tiangen Biotech, Beijing, China) on an ABI Veriti Thermal Cycler PCR System (Thermo Fisher Scientific, MA, USA).

### 2.3. Real-Time PCR

The cDNA was synthesized using the miRcute miRNA First-Strand cDNA Synthesis Kit (Tiangen Biotech, Beijing, China) on an ABI Thermal Cycler PCR System (Thermo Fisher Scientific, MA, USA). The target miRNAs were amplified using the SYBR PCR Master Mix kit. RNU6-1 was used as the endogenous reference control. Negative controls were also used to exclude any potential contamination. PCR amplification 40 cycles were followed, consisting of 15 seconds DNA denaturation at 95°C, 30 seconds annealing, and amplification at 60°C. The primer sequences for each miRNA included in the study are shown in Table 1, and all primers are designed and synthesized by Shanghai Sangon Biotechnology Co., Ltd.

### 2.4. Enrichment of the miR-181a-5p- and miR-125b-2-3p-Related mRNAs by WGCNA

Publicly available RNA-Seq and miRNA-Seq data were obtained (GEO dataset ID: GSE73714) from paired villous trophoblast and decidual basalis specimens collected from spontaneous idiopathic preterm birth or term birth [17]. The expression of miR-181a-5p and miR-125b-2-3p, sex, and gestational age placental compartment was obtained from miRNA-seq of GSE73714. mRNA quantification with log2 (RSEM>1) was included in the WGCNA. The “WGCNA” package was used to enrich the miR-181a-5p- and miR-125b-2-3p-related mRNA based on the expression of miRNAs and mRNAs from the placenta as previously performed methods [18]. A power of 8 was selected, following which the networks' connectivity distributions approximate the power law, indicating that the network possesses scale-free topology.

### 2.5. Prediction of Target Genes of Differential miRNAs by Database

The target genes of miR-181a-5p and miR-125b-2-3p were identified by using the miRWalk, miRanda, miRDB, RNA22, and TargetScan databases in the miRWalk 2.0 online tool (http://zmf.umm.uni-heidelberg.de/apps/zmf/mirwalk2/miRretsys-self.html). The target genes were further reduced by selecting those commonly predicted by at least three databases.

### 2.6. Gene Ontology (GO) and Kyoto Encyclopedia of Genes and Genomes (KEGG) Analyses

The list of identified target genes of miR-181a-5p and miR-125b-2-3 was uploaded to the DAVID (https://david.ncifcrf.gov) for functional annotation including biological process, cell component, molecular function, and KEGG pathways. Those significant GO terms and pathways were selected (*p* < 0.05), and the top 15 GO terms and pathways were visualized using the “ggplot2” package in R 4.0.5.

### 2.7. Statistical Methods

SPSS20.0 software (Bizinsight (Beijing) Information Technology Co., Ltd) was used for statistical analysis, and the experimental graphics were drawn using GraphPad Prism 7 software. The measurement values are presented as the mean ± standard deviation (mean ± SD), and the *t*-test was used to analyze the two groups. The comparison between the two groups was analyzed by *t*-test. *p* < 0.05 was considered a significant difference.

## 3. Results

### 3.1. Differential Expression of miR-181a-5p and miR-125b-2-3p in Maternal Peripheral Blood of Fetuses with Rh-HDFN and Healthy Controls

A total of 26 pregnant women who met our inclusion criteria were included in this study, including 13 controls with normal pregnancies and 13 patients with Rh-HDFN.  Table 2 indicates the general characteristics of the subjects in the study. While no significant difference was noted in the terms of body mass index (BMI) (*p* = 0.170), gestational age (*p* = 0.605), Hb (*p* = 0.098), ALT (*p* = 0.523), AST (*p* = 0.846), cholesterol level (*p* = 0.988), T-bil (*p* = 0.715), and direct-bil levels (*p* = 0.815), the maternal age from Rh-HDFN group was older than the normal group (*p* = 0.050).

**Figure 1:**
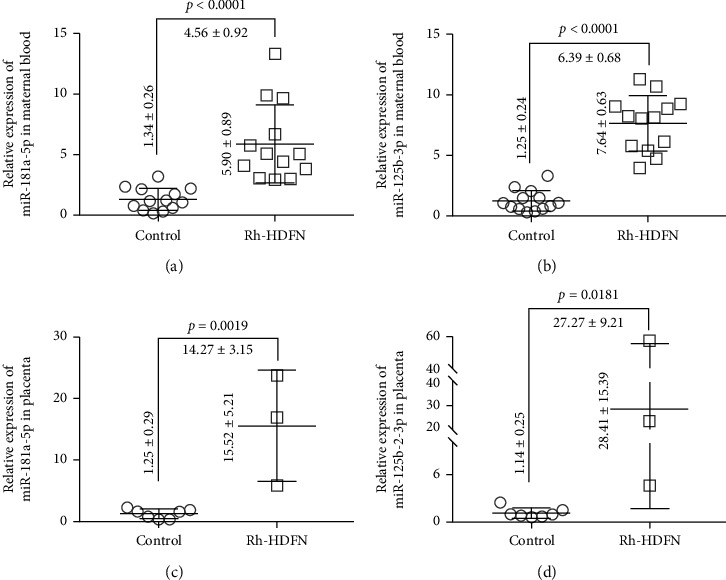
The expression of miR-181a-5p and miR-125b-2-3p in maternal plasma or placenta of Rh-HDFN fetuses and healthy controls. (a) The expression of miR-181a-5p in maternal plasma of Rh-HDFN fetuses and healthy controls. (b) The expression of miR-125b-2-3p in maternal plasma of Rh-HDFN fetuses and healthy controls. (c) The expression of miR-181a-5p in the placenta of Rh-HDFN fetuses and healthy controls. (d) The expression of miR-125b-2-3p in the placenta of the Rh-HDFN fetuses and the healthy controls.

The data presented in Figures 1(a) and 1(b) demonstrates that there was a highly significant increase in the expression of miR-181a-5p (4.56 ± 0.92-fold change, *p* < 0.0001) and miR-125b-2-3p (6.39 ± 0.68-fold change, *p* < 0.0001) in maternal peripheral blood of the RH-HDFN group compared with the control group. To further evaluate whether the expression of miR-181a-5p and miR-125b-2-3p was also differential in the placenta between the RH-HDFN and the healthy control, we collected three placental of RH-HDFN and seven healthy placentas. As shown Figures 1(c) and 1(d), both miR-181a-5p (14.27 ± 3.15-fold change, *p* = 0.0019) and miR-125b-2-3p (27.27 ± 9.21-fold change, *p* = 0.0181) are much higher expressed in placentas of the RH-HDFN than the healthy control.

### 3.2. Construction of a Weighted Coexpression Network Based on Placental mRNA Expression Profiles Related to miR-181a-5p and miR-125b-2-3p

**Figure 2:**
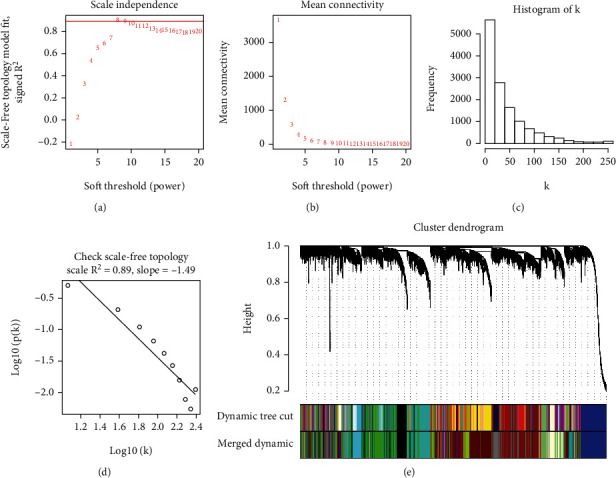
The construction of weighted gene coexpression network analysis (WGCNA) related to miR-181a-5p and miR-125b-2-3p in the placenta. (a) Scale independence soft threshold selection in WGCNA when the square value was more than the red standard line (0.9) indicated. (b) The calculation of mean connectivity according to power (*β*) values. (c) The histogram of connectivity distribution verified that the selected *β* value is approached without scale. (d) The image of the correlation shows that the selected *β* value followed the standard of scale-free topology. (e) The dynamic cut tree of the miRNA-related gene modules. The similar gene modules were merged based on the minimum number of genes in the module being 50.

To investigate whether the functions of miR-181a-5p and miR-125b-2-3p were related to placental mRNA expression, WGCNA was employed. A coexpression network was constructed by using the placental miRNA and mRNA expression profile data. To obtain a network that meets the scale-free topology criterion, different soft-thresholding power ranges from 1 to 20 were calculated. The soft thresholding power value was selected as *β* = 8 to ensure that a scale-free network (scale-free *R*2 = 0.81) was constructed (Figures 2(a)–2(d)). Then, the genes were clustered into modules by hierarchical clustering according to expression values, and the most similar modules were merged by setting the MEDissThres cutting line to 0.2 (Figure 2(e)). Finally, 22 mRNA gene modules were identified after merging similar modules with a similarity higher than 80% (Figure 2(e)). The size of mRNA modules ranges from a minimum of 64 mRNAs in the light yellow module to the 1255 mRNAs of the turquoise module. Only 99 mRNAs were not classified in any of the correlated modules and were then grouped in the gray module (Table S1).

### 3.3. Identification of miR-181a-5p- and miR-125b-2-3p-Related Functional Modules in the Placenta

**Figure 3:**
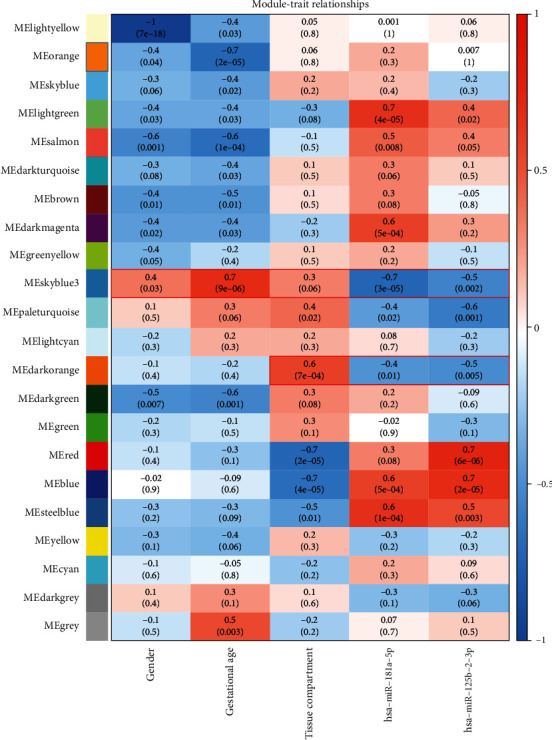
WGCNA enriched genes coexpressed with miR-181a-5p and miR-125b-2-3p in the placenta. Pearson correlation coefficient between the module eigengene of modules and the clinical features, miR-181a-5p, and miR-125b-2-3p expression analyzed via WGCNA. Upper numbers in each box indicate the correlation coefficient, and lower numbers in brackets indicate the corresponding *p* values. The red box indicates the module of interest in the study. *p* < 0.05 was considered statistically significant.

The association between several gene expression modules was examined, including the expression of miR-181a-5p and miR-125b-2-3p, as the main parameters. Interestingly, a similar set of gene modules was clustered between the expression of miR-181a-5p and miR-125b-2-3p (Figure 3). In parallel, we examined the association between each module and some clinical traits, including sex, gestational age, and placental tissue compartment (villous trophoblast and basal plate decidual basalis). Most of the clinical traits that impacted gene expression were independent from each other (Figure 3).

Regarding modules related to the expression of miR-181a-5p and miR-125b-2-3p, some of them were positively correlated with the expression of miR-181a-5p and/or miR-125b-2-3p, while some of them were negatively correlated with the expression of miR-181a-5p and/or miR-125b-2-3p. Interestingly, these modules related to the expression of miR-181a-5p and miR-125b-2-3p were always negatively correlated with gestational age or placental tissue compartment. Given that miRNAs are key negative regulators of gene expression, we further examined the specific modules (skyblue 3 and dark orange) that showed the strongest negative correlation with both miR-181a-5p and miR-125b-2-3p expression and a positive correlation with the other clinical traits (Figure 3).

### 3.4. Identification of miR-181a-5p and miR-125b-2-3p Target Genes in the Placenta by Combining the Results from WGCNA and the Database

**Figure 4:**
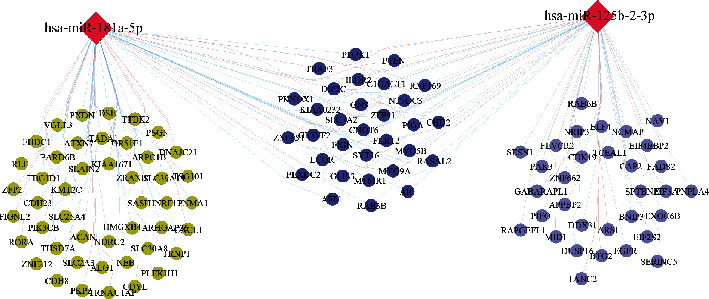
The regulatory network of miR-181a-5p and miR-125b-2-3p. The regulatory genes of miR-181a-5p are shown as green circles. The regulatory genes of miR-125b-2-3p are shown as light blue circles, and the common genes regulated by miR-181a-5p and miR-125b-2-3p are shown as dark blue circles. The color of the line represents the module to which this gene belongs.

Because miRNAs play biological functions by binding to their specific target genes, we first used the miRWalk, miRanda, miRDB, RNA22, and TargetScan databases to predict target genes for miR-125b-2-3p and miR-181a-5p. The genes that were commonly predicted by at least three databases were used as putative target genes. In total, 5239 putative target genes were identified (Supplementary Table S2), including 2308 target genes of miR-125b-2-3p and 2931 target genes of miR-181a-5p. To more accurately identify miRNA targets, the inversely correlated module genes (skyblue 3 and dark orange) with miR-125b-2-3p or miR-181a-5p expression were intersected with predictive target mRNAs in the database. Finally, 109 target genes were identified, including 31 targets that were commonly regulated by miR-125b-2-3p and miR-181a-5p (Supplementary Table S3). The miRNA–mRNA (miR-125b-2-3p, miR-181a-5p, and their target genes) regulatory network is shown in Figure 4.

### 3.5. The Regulatory Roles of miR-181a-5p and miR-125b-2-3p in Placental Function

**Figure 5:**
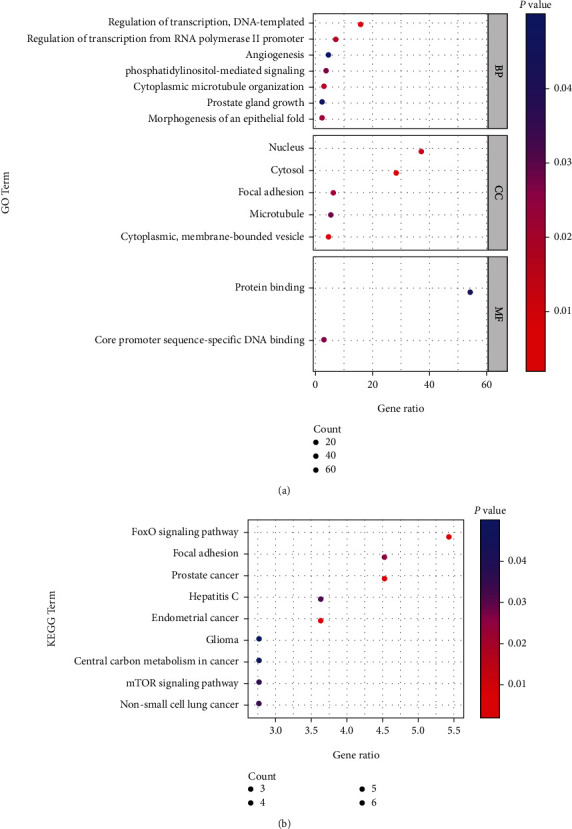
The GO and KEGG pathways of targets of miR-181a-5p and miR-125b-2-3p. (a) The enriched GO terms of target genes of miR-181a-5p and miR-125b-2-3p, including biological process (BP), cellular component (CC), and molecular function (MF). (b) The enriched KEGG pathways of miR-181a-5p and miR-125b-2-3p target genes.

To assess the biological functions of miR-181a-5p and miR-125b-2-3p in the placenta, their functions and pathways of targeted genes were analyzed by using the DAVID database. In total, 109 target genes were enriched in 14 gene ontology (GO) terms, including seven biological processes, five cell components, and two molecular functions (Figure 5(a) and Supplementary Table S4). The enriched biological processes included regulation of transcription, cytoplasmic microtubule organization, morphogenesis of an epithelial fold, phosphatidylinositol-mediated signaling, and angiogenesis, which were linked to placental function, and maintenance of the functional integrity of the placental barrier. Core promoter sequence-specific DNA binding and protein binding were highly enriched molecular functions, further indicating the potential roles of transcriptional regulation.

Furthermore, the target genes of miR-181a-5p and miR-125b-2-3p were enriched in several pathways, including the FoxO signaling pathway, prostate cancer, endometrial cancer, focal adhesion, hepatitis C, non-small cell lung cancer, mTOR signaling pathway, and central carbon metabolism in cancer, which correlated with placental function (Figure 5(b) and Supplementary Table S5).

## 4. Discussion

The placenta is a transient organ that not only plays a central role in maternal and fetal health during pregnancy but also plays a crucial role in the transplacental transfer of maternal IgG antibodies in Rh-HDFN [19]. Although Rh-D immunoprophylaxis has developed, the incidence of Rh-HDFN has dramatically dropped in recent decades. The precise prediction of Rh-HDFN is one of the final goals in maternal-fetal medicine to avoid severe complications and consequences [20]. Given the critical roles of miRNAs in placental development and functions, miRNAs are considered ideal candidate biomarkers that are ascribable to their stability and tissue specificity.

Our study revealed significantly higher expression of miR-125b-2-3p and miR-181a-5p in maternal peripheral blood of newborns with Rh-HDFN than in that of healthy newborns. The expression of miR-125b-2-3p and miR-181a-5p was 6.39- and 4.56-fold higher in maternal peripheral blood of newborns with Rh-HDFN than in normal newborns, respectively. Such expression patterns could serve as potential noninvasive biomarkers for the prediction of Rh-HDFN. Although several previous studies confirmed the diagnostic potential for miRNAs as biomarkers for pregnancy-specific diseases, the present study first reported the roles of miRNAs in Rh-HDFN [11]. MiR-181a-5p has been extensively studied in developing benign and malignant diseases [21]. In reproductive systems, miR-181a-5p was reported to mediate the effects of anti-Müllerian hormone on follicular development, trophoblast differentiation, and placental development [9, 22]. Similarly, miR-125b-2-3p has been recognized for its diverse role in vascular diseases and carcinogenesis [23, 24]. Recent studies demonstrated that miR-125b-2-3p was upregulated in preeclamptic placentas and could be exported from the human placenta into maternal circulation [25]. Although IgG antibody transplacental transfer was thought to be a crucial factor in Rh-HDFN, there was evidence that IgG transfer depends on maternal levels of total IgG, gestational age, placental integrity, and IgG subclass [1], suggesting the necessity and rationality to explain Rh-HDFN by studying placenta-related miRNAs and mRNAs. The present study provided the first evidence that high expression of miRNAs in the placenta could be detected in the maternal circulation of RH-HDFN. In addition, compared with maternal blood, the differential expression of miR-181a-5p and miR-125b-2-3p in the placenta between the RH-HDFN and the healthy control was more noticeable, suggesting that placental miRNA could be considered a new biomarker for monitoring RH-HDFN development. However, an obvious limitation of this study was that a limited sample was used. Due to the rarity of Rh-HDFN in Chinese individuals, it is necessary to collect more samples to validate further the roles of miRNAs from maternal peripheral blood in predicting Rh-HDFN.

By combining abnormal expression of miR-125b-2-3p and miR-181a-5p in maternal circulation of Rh-HDFN and the reported roles of miR-125b-2-3p and miR-181a-5p in maternal circulation and placenta, miR-125b-2-3p and miR-181a-5p probably play an important role in Rh-HDFN. However, whether dysregulation of miR-125b-2-3p and miR-181a-5p contributes to Rh-HDFN development remains unknown. We constructed a coexpression network of miR-125b-2-3p/miR-181a-5p and placental mRNA expression to clarify this. Several modules related to the expression of miR-181a-5p and miR-125b-2-3p were identified, which were always negatively correlated with gestational age or placental tissue compartment. In particular, the skyblue 3 and dark orange modules showed the strongest correlation with the expression of both miR-181a-5p and miR-125b-2-3p, indicating their modulatory roles in the two module genes. Given that miRNAs play biological functions by regulating their target genes, we first predicted target genes for miR-125b-2-3p and miR-181a-5p using several databases of miRNA target genes. To accurately identify the target genes of miR-125b-2-3p and miR-181a-5p, the intersecting genes of predictive target mRNAs in the database and genes in the skyblue 3 and dark orange modules were identified as target genes of miR-125b-2-3p and miR-181a-5p. By analyzing the functions or pathways of identified target genes, we found that the enriched biological processes included regulation of transcription, cytoplasmic microtubule organization, morphogenesis of an epithelial fold, phosphatidylinositol-mediated signaling, and angiogenesis, which were linked to placental function and maintenance of the placental barrier. Core promoter sequence-specific DNA binding and protein binding were highly enriched molecular functions, further indicating the potential roles of transcriptional regulation.

Furthermore, the target genes of miR-181a-5p and miR-125b-2-3p were enriched in several cancer-related pathways, including prostate cancer, endometrial cancer, non-small cell lung cancer, and central carbon metabolism in cancer. This result was consistent with the previous finding that several pathways are shared between the placenta and cancer cells at a molecular level [26]. Moreover, the focal adhesion, FoxO signaling pathway, and mTOR signaling pathway have important roles in placental function [27–30]. Recent evidence suggests that the FoxO signaling pathway is essential for placental morphogenesis in the developing embryo and regulates placental barrier permeability [27]. In addition, placental mTOR signaling not only plays a vital role in the regulation of fetal growth [28] but also plays an important role in placental growth signaling sensors, linking maternal nutrient and growth factor concentrations to amino acid transport by regulating placental barrier permeability [31]. Thus, the above results indicated that miR-181a-5p and miR-125b-2-3p could be functional to maintain the permeability and integration of the placental barrier, although some experiments in Rh-HDFN placenta should be performed in the future to verify our hypothesis.

## 5. Conclusion

In conclusion, we first revealed the abnormal expression of miR-181a-5p and miR-125b-2-3p in the maternal circulation of newborns with Rh-HDFN, which could play a regulatory role in placental function. Therefore, miRNAs that are specifically expressed in the placenta will be alternative and promising biomarkers to predict Rh-HDFN.

## Figures and Tables

**Table 1:** Primers sequence in the present study.

Gene symbol	Primer sequence from 5′-3′	MirBase/NCBI reference no.
miR-181a-5p	F: CATTCAACGCTGTCGGTGAGT R: CTACGTCGTATCGTCATCTGAC	MIMAT0000256
miR-125b-2-3p	F: ACAAGTCAGGCTCTTGGGAC R: CTACGTCGTATCGTCATCTGAC	MIMAT0004603
RNU6-1	F: TGGCCCCTGCGCAAGGATG R: CTACGTCGTATCGTCATCTGAC	NR_004394.1

**Table 2:** Clinical demographic data in the Rh-HDFN and control groups.

	Control	Rh-HDFN	*p* *value*
	Mean ± SD	Mean ± SD	
Maternal age (years)	34.00 ± 0.69	36.46 ± 0.97	0.050
BMI (kg/m^2^)	28.59 ± 0.69	27.00 ± 0.90	0.170
Gestational age (weeks)	38.79 ± 0.30	39.00 ± 0.26	0.605
Hb (g/L)	125.1 ± 4.31	116.5 ± 2.45	0.098
ALT (U/L)	19.77 ± 4.55	28.85 ± 13.25	0.523
AST (U/L)	24.85 ± 3.34	26.08 ± 5.28	0.846
Cholesterol (mmol/L)	5.83 ± 0.44	5.87 ± 0.21	0.988
T-bil (*μ*mol/L)	8.43 ± 0.81	7.84 ± 1.38	0.715
Direct-bil (*μ*mol/L)	2.65 ± 0.21	2.56 ± 0.29	0.815

## Data Availability

All original data for this study are included in the article/Supplementary Material. miRNA sequencing and RNA sequencing were openly obtained from the GEO dataset (ID: GSE73714).

## Additional Points

*Information pertaining to writing assistance*. No writing assistance was utilized in the production of this manuscript.

## Supplementary Materials

Supplementary Materials The following are available online, Table S1: The module color and the number of genes in each module, Table S2. The predictive target genes of miR-181a-5p and miR-125b-2-3p in different databases are shown in Table S3. The potential target genes of miR-181a-5p and miR-125b-2-3p by combinational analysis of results from WGCNA and database prediction are shown in Table S4. The GO analysis of target genes of miR-181a-5p and miR-125b-2-3p, Table S5. KEGG pathway analysis of target genes of miR-181a-5p and miR-125b-2-3p.
