# Supplementary material for: Upregulation of miR-181a-5p and miR-125b-2-3p in the Maternal Circulation of Fetuses with Rh-Negative Hemolytic Disease of the Fetus and Newborn Could Be Related to Dysfunction of Placental Function
Source: Dis Markers. 2022 Sep 21;2022:2594091. doi: 10.1155/2022/2594091 (PMC9519318; doi:10.1155/2022/2594091)
Supplement: Supplementary Materials — The following are available online, Table S1: The module color and the number of genes in each module, Table S2. The predictive target genes of miR-181a-5p and miR-125b-2-3p in different databases are shown in Table S3. The potential target genes of miR-181a-5p and miR-125b-2-3p by combinational analysis of results from WGCNA and database prediction are shown in Table S4. The GO analysis of target genes of miR-181a-5p and miR-125b-2-3p, Table S5. KEGG pathway analysis of target genes of miR-181a-5p and miR-125b-2-3p. [file 2594091.f1.docx]

**Supplementary information**

1. **Supplementary Tables**

**Table S1:** The modules color and number of genes in each module

**Table S2.** The predictive target genes of miR-181a-5p and miR-125b-2-3p in different database

**Table S3.** The potential target genes of miR-181a-5p and miR-125b-2-3p by combinational analysis of results from WGCNA and database prediction

**Table S4.** The GO analysis of target genes of miR-181a-5p and miR-125b-2-3p

**Table S5.** The KEGG pathway analysis of target genes of miR-181a-5p and miR-125b-2-3p

| **Table S1: The modules color and number of genes in each module** | |
| --- | --- |
| **Modules Color** | **Number of gene** |
| turquoise | 1255 |
| blue | 1188 |
| brown | 1136 |
| yellow | 932 |
| green | 615 |
| red | 604 |
| black | 501 |
| pink | 491 |
| magenta | 460 |
| purple | 425 |
| greenyellow | 373 |
| tan | 363 |
| salmon | 340 |
| cyan | 327 |
| midnightblue | 327 |
| lightcyan | 314 |
| grey60 | 296 |
| lightgreen | 268 |
| skyblue3 | 261 |
| royalblue | 249 |
| darkred | 234 |
| darkgreen | 220 |
| darkturquoise | 218 |
| darkgrey | 208 |
| orange | 190 |
| darkorange | 177 |
| white | 162 |
| skyblue | 161 |
| saddlebrown | 149 |
| steelblue | 148 |
| paleturquoise | 108 |
| violet | 108 |
| grey | 99 |
| darkolivegreen | 92 |
| darkmagenta | 86 |
| sienna3 | 83 |
| yellowgreen | 78 |
| lightyellow | 64 |

| **Table S2. The predictive target genes of miR-181a-5p and miR-125b-2-3p in different database** | | | | | | | | | |
| --- | --- | --- | --- | --- | --- | --- | --- | --- | --- |
| **miRNA** | **MIMATid** | **Gene** | **RefseqID** | **miRWalk** | **miRanda** | **miRDB** | **RNA22** | **Targetscan** | **SUM** |
| hsa-miR-125b-2-3p | MIMAT0004603 | CALCR | NM_001164737 | 1 | 1 | 1 | 1 | 1 | 5 |
| hsa-miR-125b-2-3p | MIMAT0004603 | CENPC | NM_001812 | 1 | 1 | 1 | 1 | 1 | 5 |
| hsa-miR-125b-2-3p | MIMAT0004603 | COL5A2 | NM_000393 | 1 | 1 | 1 | 1 | 1 | 5 |
| hsa-miR-125b-2-3p | MIMAT0004603 | CRKL | NM_005207 | 1 | 1 | 1 | 1 | 1 | 5 |
| hsa-miR-125b-2-3p | MIMAT0004603 | EYA3 | NM_001990 | 1 | 1 | 1 | 1 | 1 | 5 |
| hsa-miR-125b-2-3p | MIMAT0004603 | AFF2 | NM_002025 | 1 | 1 | 1 | 1 | 1 | 5 |
| hsa-miR-125b-2-3p | MIMAT0004603 | GRID1 | NM_017551 | 1 | 1 | 1 | 1 | 1 | 5 |
| hsa-miR-125b-2-3p | MIMAT0004603 | HNRNPU | NM_031844 | 1 | 1 | 1 | 1 | 1 | 5 |
| hsa-miR-125b-2-3p | MIMAT0004603 | MAP3K1 | NM_005921 | 1 | 1 | 1 | 1 | 1 | 5 |
| hsa-miR-125b-2-3p | MIMAT0004603 | MFAP1 | NM_005926 | 1 | 1 | 1 | 1 | 1 | 5 |
| hsa-miR-125b-2-3p | MIMAT0004603 | NCAM1 | NM_001242607 | 1 | 1 | 1 | 1 | 1 | 5 |
| hsa-miR-125b-2-3p | MIMAT0004603 | PEG3 | NM_006210 | 1 | 1 | 1 | 1 | 1 | 5 |
| hsa-miR-125b-2-3p | MIMAT0004603 | PTGS2 | NM_000963 | 1 | 1 | 1 | 1 | 1 | 5 |
| hsa-miR-125b-2-3p | MIMAT0004603 | RP2 | NM_006915 | 1 | 1 | 1 | 1 | 1 | 5 |
| hsa-miR-125b-2-3p | MIMAT0004603 | SCN9A | NM_002977 | 1 | 1 | 1 | 1 | 1 | 5 |
| hsa-miR-125b-2-3p | MIMAT0004603 | SEL1L | NM_005065 | 1 | 1 | 1 | 1 | 1 | 5 |
| hsa-miR-125b-2-3p | MIMAT0004603 | SNCA | NM_000345 | 1 | 1 | 1 | 1 | 1 | 5 |
| hsa-miR-125b-2-3p | MIMAT0004603 | SSR1 | NM_003144 | 1 | 1 | 1 | 1 | 1 | 5 |
| hsa-miR-125b-2-3p | MIMAT0004603 | SSR3 | NM_007107 | 1 | 1 | 1 | 1 | 1 | 5 |
| hsa-miR-125b-2-3p | MIMAT0004603 | TAF11 | NM_005643 | 1 | 1 | 1 | 1 | 1 | 5 |
| hsa-miR-125b-2-3p | MIMAT0004603 | TNF | NM_000594 | 1 | 1 | 1 | 1 | 1 | 5 |
| hsa-miR-125b-2-3p | MIMAT0004603 | TRAF3 | NM_145725 | 1 | 1 | 1 | 1 | 1 | 5 |
| hsa-miR-125b-2-3p | MIMAT0004603 | UBE2E1 | NM_003341 | 1 | 1 | 1 | 1 | 1 | 5 |
| hsa-miR-125b-2-3p | MIMAT0004603 | WEE1 | NM_003390 | 1 | 1 | 1 | 1 | 1 | 5 |
| hsa-miR-125b-2-3p | MIMAT0004603 | YES1 | NM_005433 | 1 | 1 | 1 | 1 | 1 | 5 |
| hsa-miR-125b-2-3p | MIMAT0004603 | ZIC3 | NM_003413 | 1 | 1 | 1 | 1 | 1 | 5 |
| hsa-miR-125b-2-3p | MIMAT0004603 | ZNF182 | NM_001178099 | 1 | 1 | 1 | 1 | 1 | 5 |
| hsa-miR-125b-2-3p | MIMAT0004603 | FZD3 | NM_017412 | 1 | 1 | 1 | 1 | 1 | 5 |
| hsa-miR-125b-2-3p | MIMAT0004603 | PIK3R3 | NM_003629 | 1 | 1 | 1 | 1 | 1 | 5 |
| hsa-miR-125b-2-3p | MIMAT0004603 | CDC14B | NM_033331 | 1 | 1 | 1 | 1 | 1 | 5 |
| hsa-miR-125b-2-3p | MIMAT0004603 | TMEM257 | NM_004709 | 1 | 1 | 1 | 1 | 1 | 5 |
| hsa-miR-125b-2-3p | MIMAT0004603 | VAPA | NM_003574 | 1 | 1 | 1 | 1 | 1 | 5 |
| hsa-miR-125b-2-3p | MIMAT0004603 | PHACTR2 | NM_001100164 | 1 | 1 | 1 | 1 | 1 | 5 |
| hsa-miR-125b-2-3p | MIMAT0004603 | DAZAP2 | NM_014764 | 1 | 1 | 1 | 1 | 1 | 5 |
| hsa-miR-125b-2-3p | MIMAT0004603 | BCL2L11 | NM_001204108 | 1 | 1 | 1 | 1 | 1 | 5 |
| hsa-miR-125b-2-3p | MIMAT0004603 | PGRMC2 | NM_006320 | 1 | 1 | 1 | 1 | 1 | 5 |
| hsa-miR-125b-2-3p | MIMAT0004603 | NPC2 | NM_006432 | 1 | 1 | 1 | 1 | 1 | 5 |
| hsa-miR-125b-2-3p | MIMAT0004603 | HBS1L | NM_006620 | 1 | 1 | 1 | 1 | 1 | 5 |
| hsa-miR-125b-2-3p | MIMAT0004603 | NUDT4 | NM_199040 | 1 | 1 | 1 | 1 | 1 | 5 |
| hsa-miR-125b-2-3p | MIMAT0004603 | CNKSR2 | NM_014927 | 1 | 1 | 1 | 1 | 1 | 5 |
| hsa-miR-125b-2-3p | MIMAT0004603 | PCNX | NM_014982 | 1 | 1 | 1 | 1 | 1 | 5 |
| hsa-miR-125b-2-3p | MIMAT0004603 | TRAM1 | NM_014294 | 1 | 1 | 1 | 1 | 1 | 5 |
| hsa-miR-125b-2-3p | MIMAT0004603 | TSPAN12 | NM_012338 | 1 | 1 | 1 | 1 | 1 | 5 |
| hsa-miR-125b-2-3p | MIMAT0004603 | ARFIP1 | NM_001025595 | 1 | 1 | 1 | 1 | 1 | 5 |
| hsa-miR-125b-2-3p | MIMAT0004603 | SOX8 | NM_014587 | 1 | 1 | 1 | 1 | 1 | 5 |
| hsa-miR-125b-2-3p | MIMAT0004603 | FAM53C | NM_001135647 | 1 | 1 | 1 | 1 | 1 | 5 |
| hsa-miR-125b-2-3p | MIMAT0004603 | RAB6B | NM_016577 | 1 | 1 | 1 | 1 | 1 | 5 |
| hsa-miR-125b-2-3p | MIMAT0004603 | GPR85 | NM_001146265 | 1 | 1 | 1 | 1 | 1 | 5 |
| hsa-miR-125b-2-3p | MIMAT0004603 | PRR13 | NM_018457 | 1 | 1 | 1 | 1 | 1 | 5 |
| hsa-miR-125b-2-3p | MIMAT0004603 | EPB41L4B | NM_019114 | 1 | 1 | 1 | 1 | 1 | 5 |
| hsa-miR-125b-2-3p | MIMAT0004603 | EGLN1 | NM_022051 | 1 | 1 | 1 | 1 | 1 | 5 |
| hsa-miR-125b-2-3p | MIMAT0004603 | RRN3 | NM_018427 | 1 | 1 | 1 | 1 | 1 | 5 |
| hsa-miR-125b-2-3p | MIMAT0004603 | VPS13C | NM_001018088 | 1 | 1 | 1 | 1 | 1 | 5 |
| hsa-miR-125b-2-3p | MIMAT0004603 | DEPDC1 | NM_001114120 | 1 | 1 | 1 | 1 | 1 | 5 |
| hsa-miR-125b-2-3p | MIMAT0004603 | NSFL1C | NM_001206736 | 1 | 1 | 1 | 1 | 1 | 5 |
| hsa-miR-125b-2-3p | MIMAT0004603 | CTNNBIP1 | NM_020248 | 1 | 1 | 1 | 1 | 1 | 5 |
| hsa-miR-125b-2-3p | MIMAT0004603 | LPAR5 | NM_020400 | 1 | 1 | 1 | 1 | 1 | 5 |
| hsa-miR-125b-2-3p | MIMAT0004603 | NUCKS1 | NM_022731 | 1 | 1 | 1 | 1 | 1 | 5 |
| hsa-miR-125b-2-3p | MIMAT0004603 | ZYG11B | NM_024646 | 1 | 1 | 1 | 1 | 1 | 5 |
| hsa-miR-125b-2-3p | MIMAT0004603 | CSRNP3 | NM_001172173 | 1 | 1 | 1 | 1 | 1 | 5 |
| hsa-miR-125b-2-3p | MIMAT0004603 | C16orf70 | NM_025187 | 1 | 1 | 1 | 1 | 1 | 5 |
| hsa-miR-125b-2-3p | MIMAT0004603 | RHBDD1 | NM_001167608 | 1 | 1 | 1 | 1 | 1 | 5 |
| hsa-miR-125b-2-3p | MIMAT0004603 | MCHR2 | NM_001040179 | 1 | 1 | 1 | 1 | 1 | 5 |
| hsa-miR-125b-2-3p | MIMAT0004603 | PPARGC1B | NM_133263 | 1 | 1 | 1 | 1 | 1 | 5 |
| hsa-miR-125b-2-3p | MIMAT0004603 | SREK1 | NM_139168 | 1 | 1 | 1 | 1 | 1 | 5 |
| hsa-miR-125b-2-3p | MIMAT0004603 | ZNF555 | NM_152791 | 1 | 1 | 1 | 1 | 1 | 5 |
| hsa-miR-125b-2-3p | MIMAT0004603 | RNF38 | NM_194328 | 1 | 1 | 1 | 1 | 1 | 5 |
| hsa-miR-125b-2-3p | MIMAT0004603 | CREBRF | NM_153607 | 1 | 1 | 1 | 1 | 1 | 5 |
| hsa-miR-125b-2-3p | MIMAT0004603 | FAM84B | NM_174911 | 1 | 1 | 1 | 1 | 1 | 5 |
| hsa-miR-125b-2-3p | MIMAT0004603 | FAM46D | NM_001170574 | 1 | 1 | 1 | 1 | 1 | 5 |
| hsa-miR-125b-2-3p | MIMAT0004603 | ZNF485 | NM_145312 | 1 | 1 | 1 | 1 | 1 | 5 |
| hsa-miR-125b-2-3p | MIMAT0004603 | RNF169 | NM_001098638 | 1 | 1 | 1 | 1 | 1 | 5 |
| hsa-miR-125b-2-3p | MIMAT0004603 | PRTG | NM_173814 | 1 | 1 | 1 | 1 | 1 | 5 |
| hsa-miR-125b-2-3p | MIMAT0004603 | KIAA2022 | NM_001008537 | 1 | 1 | 1 | 1 | 1 | 5 |
| hsa-miR-125b-2-3p | MIMAT0004603 | LIN28B | NM_001004317 | 1 | 1 | 1 | 1 | 1 | 5 |
| hsa-miR-125b-2-3p | MIMAT0004603 | C11orf87 | NM_207645 | 1 | 1 | 1 | 1 | 1 | 5 |
| hsa-miR-125b-2-3p | MIMAT0004603 | CCSER1 | NM_001145065 | 1 | 1 | 1 | 1 | 1 | 5 |
| hsa-miR-125b-2-3p | MIMAT0004603 | CDKN2B | NM_078487 | 1 | 1 | 1 | 1 | 1 | 5 |
| hsa-miR-125b-2-3p | MIMAT0004603 | GABRA4 | NM_000809 | 1 | 1 | 1 | 1 | 1 | 5 |
| hsa-miR-125b-2-3p | MIMAT0004603 | GK2 | NM_033214 | 1 | 1 | 1 | 1 | 1 | 5 |
| hsa-miR-125b-2-3p | MIMAT0004603 | IRF8 | NM_002163 | 1 | 1 | 1 | 1 | 1 | 5 |
| hsa-miR-125b-2-3p | MIMAT0004603 | MDH1 | NM_005917 | 1 | 1 | 1 | 1 | 1 | 5 |
| hsa-miR-125b-2-3p | MIMAT0004603 | NPAT | NM_002519 | 1 | 1 | 1 | 1 | 1 | 5 |
| hsa-miR-125b-2-3p | MIMAT0004603 | PCNA | NM_002592 | 1 | 1 | 1 | 1 | 1 | 5 |
| hsa-miR-125b-2-3p | MIMAT0004603 | RRM2 | NM_001165931 | 1 | 1 | 1 | 1 | 1 | 5 |
| hsa-miR-125b-2-3p | MIMAT0004603 | SLC18A2 | NM_003054 | 1 | 1 | 1 | 1 | 1 | 5 |
| hsa-miR-125b-2-3p | MIMAT0004603 | DEGS1 | NM_003676 | 1 | 1 | 1 | 1 | 1 | 5 |
| hsa-miR-125b-2-3p | MIMAT0004603 | KIAA0430 | NM_001184998 | 1 | 1 | 1 | 1 | 1 | 5 |
| hsa-miR-125b-2-3p | MIMAT0004603 | SV2B | NM_014848 | 1 | 1 | 1 | 1 | 1 | 5 |
| hsa-miR-125b-2-3p | MIMAT0004603 | PARP2 | NM_005484 | 1 | 1 | 1 | 1 | 1 | 5 |
| hsa-miR-125b-2-3p | MIMAT0004603 | EDAR | NM_022336 | 1 | 1 | 1 | 1 | 1 | 5 |
| hsa-miR-125b-2-3p | MIMAT0004603 | POP1 | NM_001145860 | 1 | 1 | 1 | 1 | 1 | 5 |
| hsa-miR-125b-2-3p | MIMAT0004603 | EXOSC2 | NM_014285 | 1 | 1 | 1 | 1 | 1 | 5 |
| hsa-miR-125b-2-3p | MIMAT0004603 | NKIRAS1 | NM_020345 | 1 | 1 | 1 | 1 | 1 | 5 |
| hsa-miR-125b-2-3p | MIMAT0004603 | NDFIP2 | NM_019080 | 1 | 1 | 1 | 1 | 1 | 5 |
| hsa-miR-125b-2-3p | MIMAT0004603 | KIAA1551 | NM_018169 | 1 | 1 | 1 | 1 | 1 | 5 |
| hsa-miR-125b-2-3p | MIMAT0004603 | TM9SF3 | NM_020123 | 1 | 1 | 1 | 1 | 1 | 5 |
| hsa-miR-125b-2-3p | MIMAT0004603 | SMIM8 | NM_001042493 | 1 | 1 | 1 | 1 | 1 | 5 |
| hsa-miR-125b-2-3p | MIMAT0004603 | OR51E2 | NM_030774 | 1 | 1 | 1 | 1 | 1 | 5 |
| hsa-miR-125b-2-3p | MIMAT0004603 | CCNL2 | NM_030937 | 1 | 1 | 1 | 1 | 1 | 5 |
| hsa-miR-125b-2-3p | MIMAT0004603 | DIRC2 | NM_032839 | 1 | 1 | 1 | 1 | 1 | 5 |
| hsa-miR-125b-2-3p | MIMAT0004603 | GORAB | NM_152281 | 1 | 1 | 1 | 1 | 1 | 5 |
| hsa-miR-125b-2-3p | MIMAT0004603 | SPECC1 | NM_152904 | 1 | 1 | 1 | 1 | 1 | 5 |
| hsa-miR-125b-2-3p | MIMAT0004603 | UBXN2B | NM_001077619 | 1 | 1 | 1 | 1 | 1 | 5 |
| hsa-miR-125b-2-3p | MIMAT0004603 | FOXR2 | NM_198451 | 1 | 1 | 1 | 1 | 1 | 5 |
| hsa-miR-125b-2-3p | MIMAT0004603 | ARHGAP42 | NM_152432 | 1 | 1 | 1 | 1 | 1 | 5 |
| hsa-miR-125b-2-3p | MIMAT0004603 | MIPOL1 | NM_001195296 | 1 | 1 | 1 | 1 | 1 | 5 |
| hsa-miR-125b-2-3p | MIMAT0004603 | FAM217A | NM_173563 | 1 | 1 | 1 | 1 | 1 | 5 |
| hsa-miR-125b-2-3p | MIMAT0004603 | MZT1 | NM_001071775 | 1 | 1 | 1 | 1 | 1 | 5 |
| hsa-miR-125b-2-3p | MIMAT0004603 | ADCYAP1R1 | NM_001199635 | 1 | 1 | 0 | 1 | 1 | 4 |
| hsa-miR-125b-2-3p | MIMAT0004603 | AMD1 | NM_001634 | 1 | 1 | 1 | 0 | 1 | 4 |
| hsa-miR-125b-2-3p | MIMAT0004603 | ANK1 | NM_001142446 | 1 | 1 | 0 | 1 | 1 | 4 |
| hsa-miR-125b-2-3p | MIMAT0004603 | AR | NM_000044 | 1 | 1 | 0 | 1 | 1 | 4 |
| hsa-miR-125b-2-3p | MIMAT0004603 | RERE | NM_012102 | 1 | 1 | 0 | 1 | 1 | 4 |
| hsa-miR-125b-2-3p | MIMAT0004603 | ATP2B4 | NM_001001396 | 1 | 1 | 0 | 1 | 1 | 4 |
| hsa-miR-125b-2-3p | MIMAT0004603 | ATP6V1C1 | NM_001695 | 1 | 1 | 1 | 0 | 1 | 4 |
| hsa-miR-125b-2-3p | MIMAT0004603 | BAG1 | NM_001172415 | 1 | 1 | 1 | 0 | 1 | 4 |
| hsa-miR-125b-2-3p | MIMAT0004603 | BCAT1 | NM_005504 | 1 | 1 | 0 | 1 | 1 | 4 |
| hsa-miR-125b-2-3p | MIMAT0004603 | BDH1 | NM_203314 | 1 | 1 | 0 | 1 | 1 | 4 |
| hsa-miR-125b-2-3p | MIMAT0004603 | BNIP3L | XM_005273616 | 1 | 1 | 0 | 1 | 1 | 4 |
| hsa-miR-125b-2-3p | MIMAT0004603 | LDLRAD4 | NM_001003674 | 1 | 1 | 0 | 1 | 1 | 4 |
| hsa-miR-125b-2-3p | MIMAT0004603 | CACNA1E | XM_005245477 | 1 | 1 | 0 | 1 | 1 | 4 |
| hsa-miR-125b-2-3p | MIMAT0004603 | CAMK2D | NM_001221 | 1 | 1 | 0 | 1 | 1 | 4 |
| hsa-miR-125b-2-3p | MIMAT0004603 | CASP3 | NM_004346 | 1 | 1 | 1 | 0 | 1 | 4 |
| hsa-miR-125b-2-3p | MIMAT0004603 | CHD2 | XM_005254836 | 1 | 1 | 0 | 1 | 1 | 4 |
| hsa-miR-125b-2-3p | MIMAT0004603 | COL1A1 | NM_000088 | 1 | 1 | 1 | 0 | 1 | 4 |
| hsa-miR-125b-2-3p | MIMAT0004603 | CPD | NM_001304 | 1 | 1 | 0 | 1 | 1 | 4 |
| hsa-miR-125b-2-3p | MIMAT0004603 | DGKA | NM_201444 | 1 | 1 | 0 | 1 | 1 | 4 |
| hsa-miR-125b-2-3p | MIMAT0004603 | DLG3 | NM_021120 | 1 | 1 | 0 | 1 | 1 | 4 |
| hsa-miR-125b-2-3p | MIMAT0004603 | DNM1 | XM_005251768 | 1 | 1 | 0 | 1 | 1 | 4 |
| hsa-miR-125b-2-3p | MIMAT0004603 | DYNC1LI2 | NM_006141 | 1 | 1 | 0 | 1 | 1 | 4 |
| hsa-miR-125b-2-3p | MIMAT0004603 | DPYSL2 | NM_001197293 | 1 | 1 | 0 | 1 | 1 | 4 |
| hsa-miR-125b-2-3p | MIMAT0004603 | DR1 | NM_001938 | 1 | 1 | 1 | 0 | 1 | 4 |
| hsa-miR-125b-2-3p | MIMAT0004603 | RCAN1 | NM_004414 | 1 | 1 | 0 | 1 | 1 | 4 |
| hsa-miR-125b-2-3p | MIMAT0004603 | EDN2 | NM_001956 | 0 | 1 | 1 | 1 | 1 | 4 |
| hsa-miR-125b-2-3p | MIMAT0004603 | EFNA5 | NM_001962 | 1 | 1 | 0 | 1 | 1 | 4 |
| hsa-miR-125b-2-3p | MIMAT0004603 | EGFR | XM_005271746 | 1 | 1 | 0 | 1 | 1 | 4 |
| hsa-miR-125b-2-3p | MIMAT0004603 | EIF1AX | NM_001412 | 1 | 1 | 0 | 1 | 1 | 4 |
| hsa-miR-125b-2-3p | MIMAT0004603 | EIF4A2 | NM_001967 | 1 | 1 | 1 | 0 | 1 | 4 |
| hsa-miR-125b-2-3p | MIMAT0004603 | ELK4 | NM_001973 | 1 | 1 | 0 | 1 | 1 | 4 |
| hsa-miR-125b-2-3p | MIMAT0004603 | ENO2 | XM_005253674 | 1 | 1 | 0 | 1 | 1 | 4 |
| hsa-miR-125b-2-3p | MIMAT0004603 | ERBB4 | XM_005246375 | 1 | 1 | 0 | 1 | 1 | 4 |
| hsa-miR-125b-2-3p | MIMAT0004603 | ETS1 | NM_005238 | 1 | 1 | 0 | 1 | 1 | 4 |
| hsa-miR-125b-2-3p | MIMAT0004603 | ACSL4 | NM_022977 | 1 | 1 | 0 | 1 | 1 | 4 |
| hsa-miR-125b-2-3p | MIMAT0004603 | GABRG1 | NM_173536 | 1 | 1 | 0 | 1 | 1 | 4 |
| hsa-miR-125b-2-3p | MIMAT0004603 | GBAS | NM_001483 | 1 | 1 | 1 | 0 | 1 | 4 |
| hsa-miR-125b-2-3p | MIMAT0004603 | GMFB | XM_005267541 | 1 | 1 | 0 | 1 | 1 | 4 |
| hsa-miR-125b-2-3p | MIMAT0004603 | GRIN2A | NM_001134407 | 1 | 1 | 0 | 1 | 1 | 4 |
| hsa-miR-125b-2-3p | MIMAT0004603 | GTF3C2 | XM_005264272 | 1 | 1 | 0 | 1 | 1 | 4 |
| hsa-miR-125b-2-3p | MIMAT0004603 | HIP1 | XM_005250304 | 1 | 1 | 0 | 1 | 1 | 4 |
| hsa-miR-125b-2-3p | MIMAT0004603 | HTR4 | XM_005268444 | 1 | 1 | 0 | 1 | 1 | 4 |
| hsa-miR-125b-2-3p | MIMAT0004603 | ISL1 | NM_002202 | 1 | 1 | 0 | 1 | 1 | 4 |
| hsa-miR-125b-2-3p | MIMAT0004603 | ITGAV | XM_005246536 | 1 | 1 | 0 | 1 | 1 | 4 |
| hsa-miR-125b-2-3p | MIMAT0004603 | IVD | NM_002225 | 0 | 1 | 1 | 1 | 1 | 4 |
| hsa-miR-125b-2-3p | MIMAT0004603 | KCNA2 | XM_005270842 | 1 | 1 | 0 | 1 | 1 | 4 |
| hsa-miR-125b-2-3p | MIMAT0004603 | KCNC1 | NM_004976 | 1 | 1 | 0 | 1 | 1 | 4 |
| hsa-miR-125b-2-3p | MIMAT0004603 | AFF3 | XM_005263941 | 1 | 1 | 0 | 1 | 1 | 4 |
| hsa-miR-125b-2-3p | MIMAT0004603 | MXD1 | NM_002357 | 1 | 1 | 0 | 1 | 1 | 4 |
| hsa-miR-125b-2-3p | MIMAT0004603 | SMAD3 | XM_005254383 | 1 | 1 | 0 | 1 | 1 | 4 |
| hsa-miR-125b-2-3p | MIMAT0004603 | MAF | NM_001031804 | 1 | 1 | 0 | 1 | 1 | 4 |
| hsa-miR-125b-2-3p | MIMAT0004603 | MAPT | NM_001123066 | 1 | 1 | 0 | 1 | 1 | 4 |
| hsa-miR-125b-2-3p | MIMAT0004603 | MDM4 | NM_002393 | 1 | 1 | 0 | 1 | 1 | 4 |
| hsa-miR-125b-2-3p | MIMAT0004603 | MECP2 | XM_005274683 | 1 | 1 | 0 | 1 | 1 | 4 |
| hsa-miR-125b-2-3p | MIMAT0004603 | MFAP3 | NM_001242336 | 1 | 1 | 0 | 1 | 1 | 4 |
| hsa-miR-125b-2-3p | MIMAT0004603 | FOXO4 | NM_005938 | 1 | 1 | 0 | 1 | 1 | 4 |
| hsa-miR-125b-2-3p | MIMAT0004603 | MME | NM_007289 | 1 | 1 | 0 | 1 | 1 | 4 |
| hsa-miR-125b-2-3p | MIMAT0004603 | MTAP | NM_002451 | 1 | 1 | 0 | 1 | 1 | 4 |
| hsa-miR-125b-2-3p | MIMAT0004603 | MTHFR | XM_005263459 | 1 | 1 | 0 | 1 | 1 | 4 |
| hsa-miR-125b-2-3p | MIMAT0004603 | MXI1 | XM_005269845 | 1 | 1 | 0 | 1 | 1 | 4 |
| hsa-miR-125b-2-3p | MIMAT0004603 | NKTR | XM_005265177 | 1 | 1 | 0 | 1 | 1 | 4 |
| hsa-miR-125b-2-3p | MIMAT0004603 | NPY1R | XM_005263031 | 1 | 1 | 0 | 1 | 1 | 4 |
| hsa-miR-125b-2-3p | MIMAT0004603 | PAK1 | NM_001128620 | 1 | 1 | 0 | 1 | 1 | 4 |
| hsa-miR-125b-2-3p | MIMAT0004603 | PAX6 | NM_000280 | 1 | 1 | 0 | 1 | 1 | 4 |
| hsa-miR-125b-2-3p | MIMAT0004603 | PBX1 | XM_005245228 | 1 | 1 | 0 | 1 | 1 | 4 |
| hsa-miR-125b-2-3p | MIMAT0004603 | PCDH9 | NM_203487 | 1 | 1 | 0 | 1 | 1 | 4 |
| hsa-miR-125b-2-3p | MIMAT0004603 | PDE3A | XM_005253389 | 1 | 1 | 0 | 1 | 1 | 4 |
| hsa-miR-125b-2-3p | MIMAT0004603 | PDK3 | NM_001142386 | 1 | 1 | 0 | 1 | 1 | 4 |
| hsa-miR-125b-2-3p | MIMAT0004603 | PDPK1 | XM_005255356 | 1 | 1 | 0 | 1 | 1 | 4 |
| hsa-miR-125b-2-3p | MIMAT0004603 | PHKG2 | NM_000294 | 1 | 1 | 0 | 1 | 1 | 4 |
| hsa-miR-125b-2-3p | MIMAT0004603 | PIGA | NM_002641 | 1 | 1 | 0 | 1 | 1 | 4 |
| hsa-miR-125b-2-3p | MIMAT0004603 | PLAG1 | NM_002655 | 1 | 1 | 0 | 1 | 1 | 4 |
| hsa-miR-125b-2-3p | MIMAT0004603 | PLIN1 | NM_002666 | 1 | 1 | 0 | 1 | 1 | 4 |
| hsa-miR-125b-2-3p | MIMAT0004603 | PNN | NM_002687 | 1 | 1 | 1 | 0 | 1 | 4 |
| hsa-miR-125b-2-3p | MIMAT0004603 | PPM1A | XM_005267777 | 1 | 1 | 0 | 1 | 1 | 4 |
| hsa-miR-125b-2-3p | MIMAT0004603 | PPP2CA | NM_002715 | 1 | 1 | 1 | 0 | 1 | 4 |
| hsa-miR-125b-2-3p | MIMAT0004603 | PPP3R1 | NM_000945 | 1 | 1 | 0 | 1 | 1 | 4 |
| hsa-miR-125b-2-3p | MIMAT0004603 | PPP6C | NM_001123355 | 1 | 1 | 0 | 1 | 1 | 4 |
| hsa-miR-125b-2-3p | MIMAT0004603 | PRKAR1A | NM_212471 | 1 | 1 | 1 | 0 | 1 | 4 |
| hsa-miR-125b-2-3p | MIMAT0004603 | MAPK1 | NM_002745 | 1 | 1 | 0 | 1 | 1 | 4 |
| hsa-miR-125b-2-3p | MIMAT0004603 | PRPSAP2 | XM_005256724 | 1 | 1 | 0 | 1 | 1 | 4 |
| hsa-miR-125b-2-3p | MIMAT0004603 | PTGER3 | NM_198715 | 1 | 1 | 0 | 1 | 1 | 4 |
| hsa-miR-125b-2-3p | MIMAT0004603 | PTGFRN | NM_020440 | 1 | 1 | 0 | 1 | 1 | 4 |
| hsa-miR-125b-2-3p | MIMAT0004603 | PTMA | NM_001099285 | 1 | 1 | 0 | 1 | 1 | 4 |
| hsa-miR-125b-2-3p | MIMAT0004603 | PTPN4 | NM_002830 | 1 | 1 | 0 | 1 | 1 | 4 |
| hsa-miR-125b-2-3p | MIMAT0004603 | RANBP2 | XM_005264002 | 1 | 1 | 0 | 1 | 1 | 4 |
| hsa-miR-125b-2-3p | MIMAT0004603 | ARID4A | XM_005267964 | 1 | 1 | 0 | 1 | 1 | 4 |
| hsa-miR-125b-2-3p | MIMAT0004603 | ROBO2 | NM_002942 | 1 | 1 | 0 | 1 | 1 | 4 |
| hsa-miR-125b-2-3p | MIMAT0004603 | RPL22 | NM_000983 | 1 | 1 | 0 | 1 | 1 | 4 |
| hsa-miR-125b-2-3p | MIMAT0004603 | RYR3 | NM_001036 | 1 | 1 | 0 | 1 | 1 | 4 |
| hsa-miR-125b-2-3p | MIMAT0004603 | SCN4B | NM_174934 | 1 | 1 | 0 | 1 | 1 | 4 |
| hsa-miR-125b-2-3p | MIMAT0004603 | CXCL12 | NM_000609 | 1 | 1 | 0 | 1 | 1 | 4 |
| hsa-miR-125b-2-3p | MIMAT0004603 | TRAPPC2 | NM_001011658 | 1 | 1 | 0 | 1 | 1 | 4 |
| hsa-miR-125b-2-3p | MIMAT0004603 | SFRP2 | NM_003013 | 1 | 1 | 0 | 1 | 1 | 4 |
| hsa-miR-125b-2-3p | MIMAT0004603 | SLC1A2 | NM_004171 | 1 | 1 | 0 | 1 | 1 | 4 |
| hsa-miR-125b-2-3p | MIMAT0004603 | SLC7A2 | NM_001008539 | 1 | 1 | 0 | 1 | 1 | 4 |
| hsa-miR-125b-2-3p | MIMAT0004603 | SLC20A1 | NM_005415 | 1 | 1 | 1 | 0 | 1 | 4 |
| hsa-miR-125b-2-3p | MIMAT0004603 | SLC22A3 | NM_021977 | 1 | 1 | 0 | 1 | 1 | 4 |
| hsa-miR-125b-2-3p | MIMAT0004603 | SNAI2 | NM_003068 | 1 | 1 | 1 | 0 | 1 | 4 |
| hsa-miR-125b-2-3p | MIMAT0004603 | SMARCC1 | NM_003074 | 1 | 1 | 0 | 1 | 1 | 4 |
| hsa-miR-125b-2-3p | MIMAT0004603 | SNRPF | NM_003095 | 1 | 1 | 1 | 0 | 1 | 4 |
| hsa-miR-125b-2-3p | MIMAT0004603 | SORD | NM_003104 | 1 | 1 | 1 | 0 | 1 | 4 |
| hsa-miR-125b-2-3p | MIMAT0004603 | SORL1 | NM_003105 | 1 | 1 | 0 | 1 | 1 | 4 |
| hsa-miR-125b-2-3p | MIMAT0004603 | SPTBN1 | NM_003128 | 1 | 1 | 0 | 1 | 1 | 4 |
| hsa-miR-125b-2-3p | MIMAT0004603 | SYT4 | NM_020783 | 1 | 1 | 0 | 1 | 1 | 4 |
| hsa-miR-125b-2-3p | MIMAT0004603 | TAL1 | NM_003189 | 1 | 1 | 0 | 1 | 1 | 4 |
| hsa-miR-125b-2-3p | MIMAT0004603 | TCEB1 | NM_001204858 | 1 | 1 | 0 | 1 | 1 | 4 |
| hsa-miR-125b-2-3p | MIMAT0004603 | TCEB3 | NM_003198 | 1 | 1 | 0 | 1 | 1 | 4 |
| hsa-miR-125b-2-3p | MIMAT0004603 | GCFC2 | NM_003203 | 1 | 1 | 0 | 1 | 1 | 4 |
| hsa-miR-125b-2-3p | MIMAT0004603 | TERF2 | NM_005652 | 1 | 1 | 0 | 1 | 1 | 4 |
| hsa-miR-125b-2-3p | MIMAT0004603 | TFRC | NM_003234 | 1 | 1 | 0 | 1 | 1 | 4 |
| hsa-miR-125b-2-3p | MIMAT0004603 | TIA1 | XM_005264529 | 1 | 1 | 0 | 1 | 1 | 4 |
| hsa-miR-125b-2-3p | MIMAT0004603 | TPD52L1 | NM_001003395 | 1 | 1 | 0 | 1 | 1 | 4 |
| hsa-miR-125b-2-3p | MIMAT0004603 | TTC3 | XM_005261054 | 1 | 1 | 0 | 1 | 1 | 4 |
| hsa-miR-125b-2-3p | MIMAT0004603 | UBE2A | NM_003336 | 1 | 1 | 0 | 1 | 1 | 4 |
| hsa-miR-125b-2-3p | MIMAT0004603 | UBE2D2 | NM_181838 | 1 | 1 | 1 | 0 | 1 | 4 |
| hsa-miR-125b-2-3p | MIMAT0004603 | UBE2G1 | NM_003342 | 1 | 1 | 0 | 1 | 1 | 4 |
| hsa-miR-125b-2-3p | MIMAT0004603 | UGDH | NM_003359 | 1 | 1 | 0 | 1 | 1 | 4 |
| hsa-miR-125b-2-3p | MIMAT0004603 | UMOD | NM_001278614 | 1 | 1 | 0 | 1 | 1 | 4 |
| hsa-miR-125b-2-3p | MIMAT0004603 | VLDLR | NM_003383 | 1 | 1 | 0 | 1 | 1 | 4 |
| hsa-miR-125b-2-3p | MIMAT0004603 | WRB | XM_005261061 | 1 | 1 | 0 | 1 | 1 | 4 |
| hsa-miR-125b-2-3p | MIMAT0004603 | XK | NM_021083 | 1 | 1 | 0 | 1 | 1 | 4 |
| hsa-miR-125b-2-3p | MIMAT0004603 | XRCC5 | XM_005246836 | 1 | 1 | 0 | 1 | 1 | 4 |
| hsa-miR-125b-2-3p | MIMAT0004603 | YWHAB | NM_003404 | 1 | 1 | 0 | 1 | 1 | 4 |
| hsa-miR-125b-2-3p | MIMAT0004603 | ZIC1 | NM_003412 | 1 | 1 | 0 | 1 | 1 | 4 |
| hsa-miR-125b-2-3p | MIMAT0004603 | ZNF24 | NM_006965 | 1 | 1 | 0 | 1 | 1 | 4 |
| hsa-miR-125b-2-3p | MIMAT0004603 | ZNF207 | XM_005258028 | 1 | 1 | 0 | 1 | 1 | 4 |
| hsa-miR-125b-2-3p | MIMAT0004603 | ZFAND5 | NM_001102420 | 1 | 1 | 1 | 0 | 1 | 4 |
| hsa-miR-125b-2-3p | MIMAT0004603 | EVI5 | NM_005665 | 1 | 1 | 0 | 1 | 1 | 4 |
| hsa-miR-125b-2-3p | MIMAT0004603 | BTG2 | NM_006763 | 1 | 1 | 0 | 1 | 1 | 4 |
| hsa-miR-125b-2-3p | MIMAT0004603 | FZD5 | NM_003468 | 1 | 1 | 0 | 1 | 1 | 4 |
| hsa-miR-125b-2-3p | MIMAT0004603 | HMGA2 | NM_003483 | 1 | 1 | 0 | 1 | 1 | 4 |
| hsa-miR-125b-2-3p | MIMAT0004603 | FZD4 | NM_012193 | 1 | 1 | 0 | 1 | 1 | 4 |
| hsa-miR-125b-2-3p | MIMAT0004603 | EEA1 | NM_003566 | 1 | 1 | 1 | 0 | 1 | 4 |
| hsa-miR-125b-2-3p | MIMAT0004603 | ATRN | NM_139321 | 1 | 1 | 0 | 1 | 1 | 4 |
| hsa-miR-125b-2-3p | MIMAT0004603 | DGKE | NM_003647 | 1 | 1 | 0 | 1 | 1 | 4 |
| hsa-miR-125b-2-3p | MIMAT0004603 | KLF7 | NM_003709 | 1 | 1 | 0 | 1 | 1 | 4 |
| hsa-miR-125b-2-3p | MIMAT0004603 | RNMT | NM_003799 | 1 | 1 | 0 | 1 | 1 | 4 |
| hsa-miR-125b-2-3p | MIMAT0004603 | RAB11A | NM_004663 | 1 | 1 | 0 | 1 | 1 | 4 |
| hsa-miR-125b-2-3p | MIMAT0004603 | NRP1 | NM_003873 | 1 | 1 | 0 | 1 | 1 | 4 |
| hsa-miR-125b-2-3p | MIMAT0004603 | HERC3 | XM_005263327 | 1 | 1 | 0 | 1 | 1 | 4 |
| hsa-miR-125b-2-3p | MIMAT0004603 | BSN | NM_003458 | 1 | 1 | 0 | 1 | 1 | 4 |
| hsa-miR-125b-2-3p | MIMAT0004603 | BAZ1B | NM_032408 | 0 | 1 | 1 | 1 | 1 | 4 |
| hsa-miR-125b-2-3p | MIMAT0004603 | SLC7A6 | NM_001076785 | 1 | 1 | 0 | 1 | 1 | 4 |
| hsa-miR-125b-2-3p | MIMAT0004603 | EBAG9 | NM_004215 | 1 | 1 | 0 | 1 | 1 | 4 |
| hsa-miR-125b-2-3p | MIMAT0004603 | MAP3K13 | NM_004721 | 1 | 1 | 0 | 1 | 1 | 4 |
| hsa-miR-125b-2-3p | MIMAT0004603 | DLGAP2 | XM_005266038 | 1 | 1 | 0 | 1 | 1 | 4 |
| hsa-miR-125b-2-3p | MIMAT0004603 | CCPG1 | NM_004748 | 1 | 1 | 1 | 0 | 1 | 4 |
| hsa-miR-125b-2-3p | MIMAT0004603 | EIF2AK3 | NM_004836 | 1 | 1 | 0 | 1 | 1 | 4 |
| hsa-miR-125b-2-3p | MIMAT0004603 | AKAP7 | NM_016377 | 1 | 1 | 0 | 1 | 1 | 4 |
| hsa-miR-125b-2-3p | MIMAT0004603 | STXBP5L | NM_014980 | 1 | 1 | 0 | 1 | 1 | 4 |
| hsa-miR-125b-2-3p | MIMAT0004603 | SPTLC2 | NM_004863 | 1 | 1 | 0 | 1 | 1 | 4 |
| hsa-miR-125b-2-3p | MIMAT0004603 | FAM53B | XM_005270300 | 1 | 1 | 0 | 1 | 1 | 4 |
| hsa-miR-125b-2-3p | MIMAT0004603 | NOS1AP | NM_001126060 | 1 | 1 | 0 | 1 | 1 | 4 |
| hsa-miR-125b-2-3p | MIMAT0004603 | FRMPD4 | NM_014728 | 1 | 1 | 1 | 0 | 1 | 4 |
| hsa-miR-125b-2-3p | MIMAT0004603 | HDAC4 | NM_006037 | 1 | 1 | 0 | 1 | 1 | 4 |
| hsa-miR-125b-2-3p | MIMAT0004603 | KIAA0247 | NM_014734 | 1 | 1 | 0 | 1 | 1 | 4 |
| hsa-miR-125b-2-3p | MIMAT0004603 | PHF16 | NM_014735 | 0 | 1 | 1 | 1 | 1 | 4 |
| hsa-miR-125b-2-3p | MIMAT0004603 | RASSF2 | XM_005260895 | 1 | 1 | 0 | 1 | 1 | 4 |
| hsa-miR-125b-2-3p | MIMAT0004603 | RABGAP1L | XM_005245680 | 1 | 1 | 0 | 1 | 1 | 4 |
| hsa-miR-125b-2-3p | MIMAT0004603 | ARNT2 | XM_005254811 | 1 | 1 | 0 | 1 | 1 | 4 |
| hsa-miR-125b-2-3p | MIMAT0004603 | ZBTB5 | XM_005251634 | 1 | 1 | 0 | 1 | 1 | 4 |
| hsa-miR-125b-2-3p | MIMAT0004603 | WDR1 | NM_017491 | 1 | 1 | 0 | 1 | 1 | 4 |
| hsa-miR-125b-2-3p | MIMAT0004603 | USP15 | NM_001252078 | 0 | 1 | 1 | 1 | 1 | 4 |
| hsa-miR-125b-2-3p | MIMAT0004603 | SH2B3 | XM_005253818 | 1 | 1 | 0 | 1 | 1 | 4 |
| hsa-miR-125b-2-3p | MIMAT0004603 | ACTR3 | NM_005721 | 1 | 1 | 1 | 0 | 1 | 4 |
| hsa-miR-125b-2-3p | MIMAT0004603 | WASF2 | NM_006990 | 1 | 1 | 0 | 1 | 1 | 4 |
| hsa-miR-125b-2-3p | MIMAT0004603 | CNIH1 | XM_005267248 | 1 | 1 | 0 | 1 | 1 | 4 |
| hsa-miR-125b-2-3p | MIMAT0004603 | LHFPL2 | NM_005779 | 1 | 1 | 0 | 1 | 1 | 4 |
| hsa-miR-125b-2-3p | MIMAT0004603 | CALCRL | NM_005795 | 1 | 1 | 1 | 0 | 1 | 4 |
| hsa-miR-125b-2-3p | MIMAT0004603 | OLIG2 | XM_005260908 | 1 | 1 | 0 | 1 | 1 | 4 |
| hsa-miR-125b-2-3p | MIMAT0004603 | SPRY3 | NM_005840 | 1 | 1 | 0 | 1 | 1 | 4 |
| hsa-miR-125b-2-3p | MIMAT0004603 | LANCL1 | XM_005246243 | 1 | 1 | 0 | 1 | 1 | 4 |
| hsa-miR-125b-2-3p | MIMAT0004603 | IKZF1 | NM_006060 | 1 | 1 | 0 | 1 | 1 | 4 |
| hsa-miR-125b-2-3p | MIMAT0004603 | TRDN | XM_005266793 | 1 | 1 | 0 | 1 | 1 | 4 |
| hsa-miR-125b-2-3p | MIMAT0004603 | HMG20A | XM_005254123 | 1 | 1 | 0 | 1 | 1 | 4 |
| hsa-miR-125b-2-3p | MIMAT0004603 | CAP2 | XM_005248801 | 1 | 1 | 0 | 1 | 1 | 4 |
| hsa-miR-125b-2-3p | MIMAT0004603 | SYNCRIP | NM_001159677 | 1 | 1 | 0 | 1 | 1 | 4 |
| hsa-miR-125b-2-3p | MIMAT0004603 | STK25 | NM_001271977 | 1 | 1 | 0 | 1 | 1 | 4 |
| hsa-miR-125b-2-3p | MIMAT0004603 | CHERP | XM_005259709 | 1 | 1 | 0 | 1 | 1 | 4 |
| hsa-miR-125b-2-3p | MIMAT0004603 | PNMA2 | XM_005273377 | 1 | 1 | 0 | 1 | 1 | 4 |
| hsa-miR-125b-2-3p | MIMAT0004603 | NFAT5 | XM_005255777 | 1 | 1 | 0 | 1 | 1 | 4 |
| hsa-miR-125b-2-3p | MIMAT0004603 | TRAF3IP2 | NM_147686 | 1 | 1 | 0 | 1 | 1 | 4 |
| hsa-miR-125b-2-3p | MIMAT0004603 | KDM5B | NM_006618 | 1 | 1 | 0 | 1 | 1 | 4 |
| hsa-miR-125b-2-3p | MIMAT0004603 | ARPP19 | XM_005254132 | 1 | 1 | 0 | 1 | 1 | 4 |
| hsa-miR-125b-2-3p | MIMAT0004603 | FAM114A2 | XM_005268360 | 1 | 1 | 0 | 1 | 1 | 4 |
| hsa-miR-125b-2-3p | MIMAT0004603 | BLCAP | NM_001167820 | 1 | 1 | 0 | 1 | 1 | 4 |
| hsa-miR-125b-2-3p | MIMAT0004603 | DBF4 | NM_006716 | 1 | 1 | 1 | 0 | 1 | 4 |
| hsa-miR-125b-2-3p | MIMAT0004603 | SPIN1 | XM_005251669 | 1 | 1 | 0 | 1 | 1 | 4 |
| hsa-miR-125b-2-3p | MIMAT0004603 | RBPMS | XM_005273387 | 1 | 1 | 0 | 1 | 1 | 4 |
| hsa-miR-125b-2-3p | MIMAT0004603 | KAT7 | NM_007067 | 1 | 1 | 0 | 1 | 1 | 4 |
| hsa-miR-125b-2-3p | MIMAT0004603 | ADAMTS6 | NM_197941 | 1 | 1 | 0 | 1 | 1 | 4 |
| hsa-miR-125b-2-3p | MIMAT0004603 | PHLDA1 | NM_007350 | 1 | 1 | 1 | 0 | 1 | 4 |
| hsa-miR-125b-2-3p | MIMAT0004603 | ZNF652 | NM_001145365 | 1 | 1 | 1 | 0 | 1 | 4 |
| hsa-miR-125b-2-3p | MIMAT0004603 | CPEB3 | XM_005269630 | 1 | 1 | 0 | 1 | 1 | 4 |
| hsa-miR-125b-2-3p | MIMAT0004603 | ZNF510 | NM_014930 | 1 | 1 | 0 | 1 | 1 | 4 |
| hsa-miR-125b-2-3p | MIMAT0004603 | NLGN1 | XM_005247231 | 1 | 1 | 0 | 1 | 1 | 4 |
| hsa-miR-125b-2-3p | MIMAT0004603 | RAB18 | NM_001256410 | 1 | 1 | 0 | 1 | 1 | 4 |
| hsa-miR-125b-2-3p | MIMAT0004603 | RPIA | NM_144563 | 1 | 1 | 0 | 1 | 1 | 4 |
| hsa-miR-125b-2-3p | MIMAT0004603 | ELL2 | NM_012081 | 1 | 1 | 1 | 0 | 1 | 4 |
| hsa-miR-125b-2-3p | MIMAT0004603 | TMCC1 | NM_001017395 | 1 | 1 | 0 | 1 | 1 | 4 |
| hsa-miR-125b-2-3p | MIMAT0004603 | SMG1 | XM_005255183 | 1 | 1 | 0 | 1 | 1 | 4 |
| hsa-miR-125b-2-3p | MIMAT0004603 | WAPAL | NM_015045 | 1 | 1 | 0 | 1 | 1 | 4 |
| hsa-miR-125b-2-3p | MIMAT0004603 | KDM4C | NM_015061 | 1 | 1 | 0 | 1 | 1 | 4 |
| hsa-miR-125b-2-3p | MIMAT0004603 | ERC1 | NM_178040 | 1 | 1 | 0 | 1 | 1 | 4 |
| hsa-miR-125b-2-3p | MIMAT0004603 | PEG10 | NM_001040152 | 1 | 1 | 1 | 0 | 1 | 4 |
| hsa-miR-125b-2-3p | MIMAT0004603 | KIF1B | XM_005263433 | 1 | 1 | 0 | 1 | 1 | 4 |
| hsa-miR-125b-2-3p | MIMAT0004603 | TNRC6B | XM_005261393 | 1 | 1 | 0 | 1 | 1 | 4 |
| hsa-miR-125b-2-3p | MIMAT0004603 | POGZ | XM_005245000 | 1 | 1 | 0 | 1 | 1 | 4 |
| hsa-miR-125b-2-3p | MIMAT0004603 | 8-Sep | NM_015146 | 1 | 1 | 0 | 1 | 1 | 4 |
| hsa-miR-125b-2-3p | MIMAT0004603 | FBXL7 | NM_012304 | 1 | 1 | 0 | 1 | 1 | 4 |
| hsa-miR-125b-2-3p | MIMAT0004603 | FAF2 | NM_014613 | 1 | 1 | 0 | 1 | 1 | 4 |
| hsa-miR-125b-2-3p | MIMAT0004603 | BICD2 | NM_001003800 | 1 | 1 | 0 | 1 | 1 | 4 |
| hsa-miR-125b-2-3p | MIMAT0004603 | MED13L | XM_005253861 | 1 | 1 | 0 | 1 | 1 | 4 |
| hsa-miR-125b-2-3p | MIMAT0004603 | ADNP | NM_015339 | 1 | 1 | 0 | 1 | 1 | 4 |
| hsa-miR-125b-2-3p | MIMAT0004603 | SIRT1 | NM_012238 | 1 | 1 | 1 | 0 | 1 | 4 |
| hsa-miR-125b-2-3p | MIMAT0004603 | RBFOX2 | XM_005261428 | 1 | 1 | 0 | 1 | 1 | 4 |
| hsa-miR-125b-2-3p | MIMAT0004603 | CORO1C | NM_001276471 | 1 | 1 | 0 | 1 | 1 | 4 |
| hsa-miR-125b-2-3p | MIMAT0004603 | CD2AP | NM_012120 | 1 | 1 | 0 | 1 | 1 | 4 |
| hsa-miR-125b-2-3p | MIMAT0004603 | SLC7A11 | NM_014331 | 1 | 1 | 0 | 1 | 1 | 4 |
| hsa-miR-125b-2-3p | MIMAT0004603 | PITPNB | NM_012399 | 1 | 1 | 0 | 1 | 1 | 4 |
| hsa-miR-125b-2-3p | MIMAT0004603 | FLRT2 | XM_005267490 | 1 | 1 | 0 | 1 | 1 | 4 |
| hsa-miR-125b-2-3p | MIMAT0004603 | ZNF318 | NM_014345 | 1 | 1 | 0 | 1 | 1 | 4 |
| hsa-miR-125b-2-3p | MIMAT0004603 | IPCEF1 | NM_001130699 | 1 | 1 | 0 | 1 | 1 | 4 |
| hsa-miR-125b-2-3p | MIMAT0004603 | DAZAP1 | NM_170711 | 1 | 1 | 1 | 0 | 1 | 4 |
| hsa-miR-125b-2-3p | MIMAT0004603 | DIEXF | NM_014388 | 1 | 1 | 1 | 0 | 1 | 4 |
| hsa-miR-125b-2-3p | MIMAT0004603 | GHITM | NM_014394 | 1 | 1 | 1 | 0 | 1 | 4 |
| hsa-miR-125b-2-3p | MIMAT0004603 | SESN1 | NM_014454 | 0 | 1 | 1 | 1 | 1 | 4 |
| hsa-miR-125b-2-3p | MIMAT0004603 | EML4 | XM_005264267 | 1 | 1 | 0 | 1 | 1 | 4 |
| hsa-miR-125b-2-3p | MIMAT0004603 | PDZRN4 | NM_001164595 | 1 | 1 | 1 | 0 | 1 | 4 |
| hsa-miR-125b-2-3p | MIMAT0004603 | PACSIN1 | NM_020804 | 1 | 1 | 0 | 1 | 1 | 4 |
| hsa-miR-125b-2-3p | MIMAT0004603 | ZBTB21 | XM_005261122 | 1 | 1 | 0 | 1 | 1 | 4 |
| hsa-miR-125b-2-3p | MIMAT0004603 | AK3 | NM_016282 | 1 | 1 | 1 | 0 | 1 | 4 |
| hsa-miR-125b-2-3p | MIMAT0004603 | ST8SIA3 | NM_015879 | 1 | 1 | 0 | 1 | 1 | 4 |
| hsa-miR-125b-2-3p | MIMAT0004603 | ZNF706 | NM_001267708 | 1 | 1 | 0 | 1 | 1 | 4 |
| hsa-miR-125b-2-3p | MIMAT0004603 | WBP5 | NM_016303 | 1 | 1 | 1 | 0 | 1 | 4 |
| hsa-miR-125b-2-3p | MIMAT0004603 | KCNK9 | XM_005250954 | 1 | 1 | 0 | 1 | 1 | 4 |
| hsa-miR-125b-2-3p | MIMAT0004603 | HPCAL4 | NM_016257 | 1 | 1 | 0 | 1 | 1 | 4 |
| hsa-miR-125b-2-3p | MIMAT0004603 | PTPLAD1 | NM_016395 | 1 | 1 | 0 | 1 | 1 | 4 |
| hsa-miR-125b-2-3p | MIMAT0004603 | PEX5L | NM_016559 | 1 | 1 | 0 | 1 | 1 | 4 |
| hsa-miR-125b-2-3p | MIMAT0004603 | RSF1 | XM_005274051 | 1 | 1 | 0 | 1 | 1 | 4 |
| hsa-miR-125b-2-3p | MIMAT0004603 | GALNT7 | NM_017423 | 1 | 1 | 1 | 0 | 1 | 4 |
| hsa-miR-125b-2-3p | MIMAT0004603 | PRKAG3 | NM_017431 | 0 | 1 | 1 | 1 | 1 | 4 |
| hsa-miR-125b-2-3p | MIMAT0004603 | ATAD2B | XM_005264372 | 1 | 1 | 0 | 1 | 1 | 4 |
| hsa-miR-125b-2-3p | MIMAT0004603 | ANKIB1 | NM_019004 | 1 | 1 | 0 | 1 | 1 | 4 |
| hsa-miR-125b-2-3p | MIMAT0004603 | MTMR12 | XM_005248313 | 1 | 1 | 0 | 1 | 1 | 4 |
| hsa-miR-125b-2-3p | MIMAT0004603 | 5-Mar | XM_005269924 | 1 | 1 | 0 | 1 | 1 | 4 |
| hsa-miR-125b-2-3p | MIMAT0004603 | CNGB3 | NM_019098 | 1 | 1 | 0 | 1 | 1 | 4 |
| hsa-miR-125b-2-3p | MIMAT0004603 | LEPROT | XM_005270951 | 1 | 1 | 0 | 1 | 1 | 4 |
| hsa-miR-125b-2-3p | MIMAT0004603 | IL17RD | XM_005265238 | 1 | 1 | 0 | 1 | 1 | 4 |
| hsa-miR-125b-2-3p | MIMAT0004603 | FAM46C | XM_005270960 | 1 | 1 | 0 | 1 | 1 | 4 |
| hsa-miR-125b-2-3p | MIMAT0004603 | C4orf27 | NM_017867 | 1 | 1 | 1 | 0 | 1 | 4 |
| hsa-miR-125b-2-3p | MIMAT0004603 | PRPF39 | XM_005267787 | 1 | 1 | 0 | 1 | 1 | 4 |
| hsa-miR-125b-2-3p | MIMAT0004603 | USP47 | XM_005252997 | 1 | 1 | 0 | 1 | 1 | 4 |
| hsa-miR-125b-2-3p | MIMAT0004603 | PDPR | XM_005256016 | 1 | 1 | 0 | 1 | 1 | 4 |
| hsa-miR-125b-2-3p | MIMAT0004603 | CXorf57 | NM_018015 | 0 | 1 | 1 | 1 | 1 | 4 |
| hsa-miR-125b-2-3p | MIMAT0004603 | SLC38A4 | NM_018018 | 0 | 1 | 1 | 1 | 1 | 4 |
| hsa-miR-125b-2-3p | MIMAT0004603 | BSDC1 | XM_005270985 | 1 | 1 | 0 | 1 | 1 | 4 |
| hsa-miR-125b-2-3p | MIMAT0004603 | PCMTD2 | XM_005260222 | 1 | 1 | 0 | 1 | 1 | 4 |
| hsa-miR-125b-2-3p | MIMAT0004603 | ZNF654 | XM_005264762 | 1 | 1 | 0 | 1 | 1 | 4 |
| hsa-miR-125b-2-3p | MIMAT0004603 | SLC39A9 | NM_018375 | 1 | 1 | 0 | 1 | 1 | 4 |
| hsa-miR-125b-2-3p | MIMAT0004603 | FAM46A | XM_005248731 | 1 | 1 | 0 | 1 | 1 | 4 |
| hsa-miR-125b-2-3p | MIMAT0004603 | PRPF40A | XM_005246679 | 1 | 1 | 0 | 1 | 1 | 4 |
| hsa-miR-125b-2-3p | MIMAT0004603 | ASF1B | NM_018154 | 1 | 1 | 0 | 1 | 1 | 4 |
| hsa-miR-125b-2-3p | MIMAT0004603 | PRR11 | NM_018304 | 1 | 1 | 1 | 0 | 1 | 4 |
| hsa-miR-125b-2-3p | MIMAT0004603 | LRIF1 | XM_005271029 | 1 | 1 | 0 | 1 | 1 | 4 |
| hsa-miR-125b-2-3p | MIMAT0004603 | DOK5 | NM_018431 | 1 | 1 | 0 | 1 | 1 | 4 |
| hsa-miR-125b-2-3p | MIMAT0004603 | GNG12 | NM_018841 | 1 | 1 | 0 | 1 | 1 | 4 |
| hsa-miR-125b-2-3p | MIMAT0004603 | NRIP3 | NM_020645 | 1 | 1 | 0 | 1 | 1 | 4 |
| hsa-miR-125b-2-3p | MIMAT0004603 | SAR1A | NM_001142648 | 1 | 1 | 0 | 1 | 1 | 4 |
| hsa-miR-125b-2-3p | MIMAT0004603 | CASS4 | NM_001164116 | 1 | 1 | 0 | 1 | 1 | 4 |
| hsa-miR-125b-2-3p | MIMAT0004603 | PELI2 | NM_021255 | 1 | 1 | 1 | 0 | 1 | 4 |
| hsa-miR-125b-2-3p | MIMAT0004603 | ZNF248 | NM_021045 | 1 | 1 | 0 | 1 | 1 | 4 |
| hsa-miR-125b-2-3p | MIMAT0004603 | SLC4A10 | NM_001178016 | 1 | 1 | 0 | 1 | 1 | 4 |
| hsa-miR-125b-2-3p | MIMAT0004603 | SLC24A3 | NM_020689 | 1 | 1 | 0 | 1 | 1 | 4 |
| hsa-miR-125b-2-3p | MIMAT0004603 | NLGN4X | XM_005274567 | 1 | 1 | 0 | 1 | 1 | 4 |
| hsa-miR-125b-2-3p | MIMAT0004603 | NUFIP2 | NM_020772 | 1 | 1 | 0 | 1 | 1 | 4 |
| hsa-miR-125b-2-3p | MIMAT0004603 | MIB1 | NM_020774 | 1 | 1 | 0 | 1 | 1 | 4 |
| hsa-miR-125b-2-3p | MIMAT0004603 | TAOK1 | NM_020791 | 1 | 1 | 1 | 0 | 1 | 4 |
| hsa-miR-125b-2-3p | MIMAT0004603 | FAM135A | XM_005248743 | 1 | 1 | 0 | 1 | 1 | 4 |
| hsa-miR-125b-2-3p | MIMAT0004603 | RANBP10 | XM_005256071 | 1 | 1 | 0 | 1 | 1 | 4 |
| hsa-miR-125b-2-3p | MIMAT0004603 | DENND1A | XM_005252110 | 1 | 1 | 0 | 1 | 1 | 4 |
| hsa-miR-125b-2-3p | MIMAT0004603 | GPR107 | NM_001136557 | 1 | 1 | 0 | 1 | 1 | 4 |
| hsa-miR-125b-2-3p | MIMAT0004603 | PCTP | NM_001102402 | 1 | 1 | 0 | 1 | 1 | 4 |
| hsa-miR-125b-2-3p | MIMAT0004603 | GNB4 | NM_021629 | 1 | 1 | 1 | 0 | 1 | 4 |
| hsa-miR-125b-2-3p | MIMAT0004603 | SPCS3 | NM_021928 | 1 | 1 | 0 | 1 | 1 | 4 |
| hsa-miR-125b-2-3p | MIMAT0004603 | PROK2 | NM_001126128 | 1 | 1 | 0 | 1 | 1 | 4 |
| hsa-miR-125b-2-3p | MIMAT0004603 | SLC22A23 | XM_005249285 | 1 | 1 | 0 | 1 | 1 | 4 |
| hsa-miR-125b-2-3p | MIMAT0004603 | PRDM16 | XM_005244772 | 1 | 1 | 0 | 1 | 1 | 4 |
| hsa-miR-125b-2-3p | MIMAT0004603 | C5orf28 | NM_022483 | 1 | 1 | 0 | 1 | 1 | 4 |
| hsa-miR-125b-2-3p | MIMAT0004603 | SUDS3 | NM_022491 | 1 | 1 | 0 | 1 | 1 | 4 |
| hsa-miR-125b-2-3p | MIMAT0004603 | S100PBP | XM_005271121 | 1 | 1 | 0 | 1 | 1 | 4 |
| hsa-miR-125b-2-3p | MIMAT0004603 | USP46 | NM_001134223 | 1 | 1 | 1 | 0 | 1 | 4 |
| hsa-miR-125b-2-3p | MIMAT0004603 | ZBTB10 | NM_001105539 | 1 | 1 | 0 | 1 | 1 | 4 |
| hsa-miR-125b-2-3p | MIMAT0004603 | C12orf49 | XM_005253937 | 1 | 1 | 0 | 1 | 1 | 4 |
| hsa-miR-125b-2-3p | MIMAT0004603 | FAM57A | NM_024792 | 1 | 1 | 0 | 1 | 1 | 4 |
| hsa-miR-125b-2-3p | MIMAT0004603 | PYROXD1 | NM_024854 | 1 | 1 | 1 | 0 | 1 | 4 |
| hsa-miR-125b-2-3p | MIMAT0004603 | VCPIP1 | NM_025054 | 1 | 1 | 0 | 1 | 1 | 4 |
| hsa-miR-125b-2-3p | MIMAT0004603 | CPEB4 | NM_030627 | 1 | 1 | 0 | 1 | 1 | 4 |
| hsa-miR-125b-2-3p | MIMAT0004603 | CYB5B | NM_030579 | 1 | 1 | 0 | 1 | 1 | 4 |
| hsa-miR-125b-2-3p | MIMAT0004603 | JHDM1D | NM_030647 | 1 | 1 | 0 | 1 | 1 | 4 |
| hsa-miR-125b-2-3p | MIMAT0004603 | FAM49A | NM_030797 | 0 | 1 | 1 | 1 | 1 | 4 |
| hsa-miR-125b-2-3p | MIMAT0004603 | TSPAN14 | XM_005270192 | 1 | 1 | 0 | 1 | 1 | 4 |
| hsa-miR-125b-2-3p | MIMAT0004603 | TMEM47 | NM_031442 | 0 | 1 | 1 | 1 | 1 | 4 |
| hsa-miR-125b-2-3p | MIMAT0004603 | SH3BGRL2 | NM_031469 | 1 | 1 | 0 | 1 | 1 | 4 |
| hsa-miR-125b-2-3p | MIMAT0004603 | FSD1L | XM_005252254 | 1 | 1 | 0 | 1 | 1 | 4 |
| hsa-miR-125b-2-3p | MIMAT0004603 | SLC10A7 | XM_005263276 | 1 | 1 | 0 | 1 | 1 | 4 |
| hsa-miR-125b-2-3p | MIMAT0004603 | LCOR | NM_032440 | 1 | 1 | 0 | 1 | 1 | 4 |
| hsa-miR-125b-2-3p | MIMAT0004603 | GLYR1 | XM_005255637 | 1 | 1 | 0 | 1 | 1 | 4 |
| hsa-miR-125b-2-3p | MIMAT0004603 | UBASH3B | NM_032873 | 1 | 1 | 0 | 1 | 1 | 4 |
| hsa-miR-125b-2-3p | MIMAT0004603 | REPS1 | NM_031922 | 1 | 1 | 0 | 1 | 1 | 4 |
| hsa-miR-125b-2-3p | MIMAT0004603 | TSPYL5 | NM_033512 | 1 | 1 | 0 | 1 | 1 | 4 |
| hsa-miR-125b-2-3p | MIMAT0004603 | SSH2 | XM_005258058 | 1 | 1 | 0 | 1 | 1 | 4 |
| hsa-miR-125b-2-3p | MIMAT0004603 | NAV1 | XM_005245572 | 1 | 1 | 0 | 1 | 1 | 4 |
| hsa-miR-125b-2-3p | MIMAT0004603 | KLHL6 | NM_130446 | 1 | 1 | 0 | 1 | 1 | 4 |
| hsa-miR-125b-2-3p | MIMAT0004603 | ANKRD40 | NM_052855 | 1 | 1 | 0 | 1 | 1 | 4 |
| hsa-miR-125b-2-3p | MIMAT0004603 | ALKBH8 | NM_138775 | 0 | 1 | 1 | 1 | 1 | 4 |
| hsa-miR-125b-2-3p | MIMAT0004603 | MTDH | NM_178812 | 1 | 1 | 0 | 1 | 1 | 4 |
| hsa-miR-125b-2-3p | MIMAT0004603 | CDHR1 | NM_033100 | 1 | 1 | 0 | 1 | 1 | 4 |
| hsa-miR-125b-2-3p | MIMAT0004603 | OPALIN | NM_001040103 | 1 | 1 | 0 | 1 | 1 | 4 |
| hsa-miR-125b-2-3p | MIMAT0004603 | MMGT1 | NM_173470 | 1 | 1 | 0 | 1 | 1 | 4 |
| hsa-miR-125b-2-3p | MIMAT0004603 | FOXP2 | NM_148898 | 1 | 1 | 0 | 1 | 1 | 4 |
| hsa-miR-125b-2-3p | MIMAT0004603 | CEP41 | NM_018718 | 1 | 1 | 0 | 1 | 1 | 4 |
| hsa-miR-125b-2-3p | MIMAT0004603 | RWDD2A | XM_005248647 | 1 | 1 | 0 | 1 | 1 | 4 |
| hsa-miR-125b-2-3p | MIMAT0004603 | PCMTD1 | NM_052937 | 1 | 1 | 1 | 0 | 1 | 4 |
| hsa-miR-125b-2-3p | MIMAT0004603 | ZNF641 | NM_152320 | 1 | 1 | 0 | 1 | 1 | 4 |
| hsa-miR-125b-2-3p | MIMAT0004603 | FGD4 | XM_005253304 | 1 | 1 | 0 | 1 | 1 | 4 |
| hsa-miR-125b-2-3p | MIMAT0004603 | JDP2 | NM_130469 | 1 | 1 | 0 | 1 | 1 | 4 |
| hsa-miR-125b-2-3p | MIMAT0004603 | TMEM170A | XM_005255795 | 1 | 1 | 0 | 1 | 1 | 4 |
| hsa-miR-125b-2-3p | MIMAT0004603 | C1orf173 | NM_001002912 | 1 | 1 | 0 | 1 | 1 | 4 |
| hsa-miR-125b-2-3p | MIMAT0004603 | KLF17 | NM_173484 | 1 | 1 | 0 | 1 | 1 | 4 |
| hsa-miR-125b-2-3p | MIMAT0004603 | C22orf39 | NM_173793 | 1 | 1 | 0 | 1 | 1 | 4 |
| hsa-miR-125b-2-3p | MIMAT0004603 | FAM168B | NM_001009993 | 1 | 1 | 0 | 1 | 1 | 4 |
| hsa-miR-125b-2-3p | MIMAT0004603 | KCNH8 | NM_144633 | 0 | 1 | 1 | 1 | 1 | 4 |
| hsa-miR-125b-2-3p | MIMAT0004603 | DCBLD2 | XM_005247118 | 1 | 1 | 0 | 1 | 1 | 4 |
| hsa-miR-125b-2-3p | MIMAT0004603 | CPEB2 | NM_001177382 | 1 | 1 | 0 | 1 | 1 | 4 |
| hsa-miR-125b-2-3p | MIMAT0004603 | DCAF12L1 | NM_178470 | 1 | 1 | 1 | 0 | 1 | 4 |
| hsa-miR-125b-2-3p | MIMAT0004603 | HDX | NM_001177479 | 1 | 1 | 0 | 1 | 1 | 4 |
| hsa-miR-125b-2-3p | MIMAT0004603 | TLDC2 | NM_080628 | 1 | 1 | 0 | 1 | 1 | 4 |
| hsa-miR-125b-2-3p | MIMAT0004603 | DUSP19 | NM_080876 | 1 | 1 | 0 | 1 | 1 | 4 |
| hsa-miR-125b-2-3p | MIMAT0004603 | SAMD8 | NM_144660 | 1 | 1 | 0 | 1 | 1 | 4 |
| hsa-miR-125b-2-3p | MIMAT0004603 | GSG1L | NM_001109763 | 1 | 1 | 0 | 1 | 1 | 4 |
| hsa-miR-125b-2-3p | MIMAT0004603 | C1orf52 | NM_198077 | 1 | 1 | 1 | 0 | 1 | 4 |
| hsa-miR-125b-2-3p | MIMAT0004603 | RC3H1 | NM_172071 | 1 | 1 | 0 | 1 | 1 | 4 |
| hsa-miR-125b-2-3p | MIMAT0004603 | FAM117B | NM_173511 | 1 | 1 | 0 | 1 | 1 | 4 |
| hsa-miR-125b-2-3p | MIMAT0004603 | CCDC50 | NM_178335 | 1 | 1 | 0 | 1 | 1 | 4 |
| hsa-miR-125b-2-3p | MIMAT0004603 | SPICE1 | NM_144718 | 1 | 1 | 0 | 1 | 1 | 4 |
| hsa-miR-125b-2-3p | MIMAT0004603 | MBLAC2 | NM_203406 | 0 | 1 | 1 | 1 | 1 | 4 |
| hsa-miR-125b-2-3p | MIMAT0004603 | SRFBP1 | NM_152546 | 1 | 1 | 1 | 0 | 1 | 4 |
| hsa-miR-125b-2-3p | MIMAT0004603 | GIMAP8 | XM_005249950 | 1 | 1 | 0 | 1 | 1 | 4 |
| hsa-miR-125b-2-3p | MIMAT0004603 | DENND5B | NM_144973 | 1 | 1 | 0 | 1 | 1 | 4 |
| hsa-miR-125b-2-3p | MIMAT0004603 | SGMS2 | XM_005262793 | 1 | 1 | 0 | 1 | 1 | 4 |
| hsa-miR-125b-2-3p | MIMAT0004603 | DCP2 | NM_152624 | 1 | 1 | 0 | 1 | 1 | 4 |
| hsa-miR-125b-2-3p | MIMAT0004603 | TMEM64 | NM_001008495 | 1 | 1 | 0 | 1 | 1 | 4 |
| hsa-miR-125b-2-3p | MIMAT0004603 | ASXL1 | NM_015338 | 1 | 1 | 0 | 1 | 1 | 4 |
| hsa-miR-125b-2-3p | MIMAT0004603 | PGAM5 | NM_001170543 | 1 | 1 | 0 | 1 | 1 | 4 |
| hsa-miR-125b-2-3p | MIMAT0004603 | AGO4 | NM_017629 | 1 | 1 | 0 | 1 | 1 | 4 |
| hsa-miR-125b-2-3p | MIMAT0004603 | ZNF585A | XM_005258615 | 1 | 1 | 0 | 1 | 1 | 4 |
| hsa-miR-125b-2-3p | MIMAT0004603 | SPRED2 | NM_181784 | 1 | 1 | 1 | 0 | 1 | 4 |
| hsa-miR-125b-2-3p | MIMAT0004603 | C4orf46 | NM_001008393 | 1 | 1 | 0 | 1 | 1 | 4 |
| hsa-miR-125b-2-3p | MIMAT0004603 | C9orf91 | XM_005251792 | 1 | 1 | 0 | 1 | 1 | 4 |
| hsa-miR-125b-2-3p | MIMAT0004603 | TMEM26 | NM_178505 | 1 | 1 | 0 | 1 | 1 | 4 |
| hsa-miR-125b-2-3p | MIMAT0004603 | ZCCHC24 | NM_153367 | 1 | 1 | 0 | 1 | 1 | 4 |
| hsa-miR-125b-2-3p | MIMAT0004603 | DOK6 | NM_152721 | 1 | 1 | 0 | 1 | 1 | 4 |
| hsa-miR-125b-2-3p | MIMAT0004603 | SMIM13 | NM_001135575 | 1 | 1 | 1 | 0 | 1 | 4 |
| hsa-miR-125b-2-3p | MIMAT0004603 | ATXN7L1 | XM_005250220 | 1 | 1 | 0 | 1 | 1 | 4 |
| hsa-miR-125b-2-3p | MIMAT0004603 | SLC35F1 | NM_001029858 | 1 | 1 | 1 | 0 | 1 | 4 |
| hsa-miR-125b-2-3p | MIMAT0004603 | STXBP4 | XM_005257185 | 1 | 1 | 0 | 1 | 1 | 4 |
| hsa-miR-125b-2-3p | MIMAT0004603 | HEPACAM2 | XM_005250247 | 1 | 1 | 0 | 1 | 1 | 4 |
| hsa-miR-125b-2-3p | MIMAT0004603 | LCORL | XM_005248145 | 1 | 1 | 0 | 1 | 1 | 4 |
| hsa-miR-125b-2-3p | MIMAT0004603 | MCOLN2 | NM_153259 | 1 | 1 | 0 | 1 | 1 | 4 |
| hsa-miR-125b-2-3p | MIMAT0004603 | ZNF549 | NM_001199295 | 1 | 1 | 0 | 1 | 1 | 4 |
| hsa-miR-125b-2-3p | MIMAT0004603 | TMEM196 | NM_152774 | 1 | 1 | 1 | 0 | 1 | 4 |
| hsa-miR-125b-2-3p | MIMAT0004603 | STEAP2 | NM_001244944 | 1 | 1 | 1 | 0 | 1 | 4 |
| hsa-miR-125b-2-3p | MIMAT0004603 | JAKMIP3 | XM_005252678 | 1 | 1 | 0 | 1 | 1 | 4 |
| hsa-miR-125b-2-3p | MIMAT0004603 | RBM20 | NM_001134363 | 1 | 1 | 0 | 1 | 1 | 4 |
| hsa-miR-125b-2-3p | MIMAT0004603 | C14orf177 | NM_182560 | 0 | 1 | 1 | 1 | 1 | 4 |
| hsa-miR-125b-2-3p | MIMAT0004603 | TPRX1 | XM_005258788 | 1 | 1 | 0 | 1 | 1 | 4 |
| hsa-miR-125b-2-3p | MIMAT0004603 | HCAR2 | XM_005253569 | 1 | 1 | 0 | 1 | 1 | 4 |
| hsa-miR-125b-2-3p | MIMAT0004603 | RSPO2 | NM_178565 | 1 | 1 | 0 | 1 | 1 | 4 |
| hsa-miR-125b-2-3p | MIMAT0004603 | ATXN1L | NM_001137675 | 1 | 1 | 1 | 0 | 1 | 4 |
| hsa-miR-125b-2-3p | MIMAT0004603 | TMEM110 | NM_198563 | 1 | 1 | 0 | 1 | 1 | 4 |
| hsa-miR-125b-2-3p | MIMAT0004603 | SKIDA1 | NM_207371 | 0 | 1 | 1 | 1 | 1 | 4 |
| hsa-miR-125b-2-3p | MIMAT0004603 | BEND4 | NM_207406 | 0 | 1 | 1 | 1 | 1 | 4 |
| hsa-miR-125b-2-3p | MIMAT0004603 | SAMD12 | NM_001101676 | 1 | 1 | 0 | 1 | 1 | 4 |
| hsa-miR-125b-2-3p | MIMAT0004603 | ASAH2B | XM_005270059 | 1 | 1 | 0 | 1 | 1 | 4 |
| hsa-miR-125b-2-3p | MIMAT0004603 | CCDC152 | NM_001134848 | 1 | 1 | 0 | 1 | 1 | 4 |
| hsa-miR-125b-2-3p | MIMAT0004603 | TMEM194B | NM_001142645 | 1 | 1 | 1 | 0 | 1 | 4 |
| hsa-miR-125b-2-3p | MIMAT0004603 | ZNF605 | NM_183238 | 1 | 1 | 0 | 1 | 1 | 4 |
| hsa-miR-125b-2-3p | MIMAT0004603 | BIRC5 | NM_001012271 | 1 | 1 | 0 | 1 | 1 | 4 |
| hsa-miR-125b-2-3p | MIMAT0004603 | ASAH1 | NM_177924 | 1 | 1 | 0 | 1 | 1 | 4 |
| hsa-miR-125b-2-3p | MIMAT0004603 | ATP1A2 | NM_000702 | 1 | 1 | 0 | 1 | 1 | 4 |
| hsa-miR-125b-2-3p | MIMAT0004603 | ALDH7A1 | NM_001182 | 1 | 1 | 0 | 1 | 1 | 4 |
| hsa-miR-125b-2-3p | MIMAT0004603 | BID | NM_197966 | 1 | 1 | 0 | 1 | 1 | 4 |
| hsa-miR-125b-2-3p | MIMAT0004603 | BMPR1A | NM_004329 | 1 | 1 | 1 | 0 | 1 | 4 |
| hsa-miR-125b-2-3p | MIMAT0004603 | BOK | NM_032515 | 1 | 1 | 0 | 1 | 1 | 4 |
| hsa-miR-125b-2-3p | MIMAT0004603 | CAPNS1 | XM_005259296 | 1 | 1 | 0 | 1 | 1 | 4 |
| hsa-miR-125b-2-3p | MIMAT0004603 | CASP9 | NM_001229 | 1 | 1 | 0 | 1 | 1 | 4 |
| hsa-miR-125b-2-3p | MIMAT0004603 | CBFA2T3 | NM_005187 | 1 | 1 | 0 | 1 | 1 | 4 |
| hsa-miR-125b-2-3p | MIMAT0004603 | CD36 | NM_001127443 | 1 | 1 | 1 | 0 | 1 | 4 |
| hsa-miR-125b-2-3p | MIMAT0004603 | CDC5L | NM_001253 | 1 | 1 | 0 | 1 | 1 | 4 |
| hsa-miR-125b-2-3p | MIMAT0004603 | CDH5 | NM_001795 | 1 | 1 | 0 | 1 | 1 | 4 |
| hsa-miR-125b-2-3p | MIMAT0004603 | CDK9 | NM_001261 | 1 | 1 | 0 | 1 | 1 | 4 |
| hsa-miR-125b-2-3p | MIMAT0004603 | CHM | NM_000390 | 1 | 1 | 0 | 1 | 1 | 4 |
| hsa-miR-125b-2-3p | MIMAT0004603 | COL5A1 | NM_000093 | 0 | 1 | 1 | 1 | 1 | 4 |
| hsa-miR-125b-2-3p | MIMAT0004603 | COMT | NM_000754 | 1 | 1 | 0 | 1 | 1 | 4 |
| hsa-miR-125b-2-3p | MIMAT0004603 | COX7A2 | NM_001865 | 1 | 1 | 0 | 1 | 1 | 4 |
| hsa-miR-125b-2-3p | MIMAT0004603 | CRX | NM_000554 | 1 | 1 | 0 | 1 | 1 | 4 |
| hsa-miR-125b-2-3p | MIMAT0004603 | CRY1 | NM_004075 | 0 | 1 | 1 | 1 | 1 | 4 |
| hsa-miR-125b-2-3p | MIMAT0004603 | CSTB | NM_000100 | 1 | 1 | 1 | 0 | 1 | 4 |
| hsa-miR-125b-2-3p | MIMAT0004603 | CTNS | NM_004937 | 1 | 1 | 1 | 0 | 1 | 4 |
| hsa-miR-125b-2-3p | MIMAT0004603 | CTSV | NM_001333 | 1 | 1 | 1 | 0 | 1 | 4 |
| hsa-miR-125b-2-3p | MIMAT0004603 | E2F6 | NM_198256 | 1 | 1 | 1 | 0 | 1 | 4 |
| hsa-miR-125b-2-3p | MIMAT0004603 | EIF5 | NM_001969 | 1 | 1 | 0 | 1 | 1 | 4 |
| hsa-miR-125b-2-3p | MIMAT0004603 | ELF4 | NM_001421 | 1 | 1 | 0 | 1 | 1 | 4 |
| hsa-miR-125b-2-3p | MIMAT0004603 | EPHA3 | NM_005233 | 1 | 1 | 1 | 0 | 1 | 4 |
| hsa-miR-125b-2-3p | MIMAT0004603 | ESR1 | NM_001122742 | 1 | 1 | 0 | 1 | 1 | 4 |
| hsa-miR-125b-2-3p | MIMAT0004603 | PTK2B | NM_173174 | 1 | 1 | 0 | 1 | 1 | 4 |
| hsa-miR-125b-2-3p | MIMAT0004603 | FGF1 | NM_000800 | 1 | 1 | 0 | 1 | 1 | 4 |
| hsa-miR-125b-2-3p | MIMAT0004603 | GNAO1 | NM_138736 | 1 | 1 | 0 | 1 | 1 | 4 |
| hsa-miR-125b-2-3p | MIMAT0004603 | GRM1 | XM_005266950 | 1 | 1 | 0 | 1 | 1 | 4 |
| hsa-miR-125b-2-3p | MIMAT0004603 | HAS3 | XM_005255919 | 1 | 1 | 0 | 1 | 1 | 4 |
| hsa-miR-125b-2-3p | MIMAT0004603 | HCCS | NM_005333 | 1 | 1 | 0 | 1 | 1 | 4 |
| hsa-miR-125b-2-3p | MIMAT0004603 | HLA-DOA | NM_002119 | 1 | 1 | 0 | 1 | 1 | 4 |
| hsa-miR-125b-2-3p | MIMAT0004603 | IFNAR1 | NM_000629 | 0 | 1 | 1 | 1 | 1 | 4 |
| hsa-miR-125b-2-3p | MIMAT0004603 | IGF2 | NM_000612 | 1 | 1 | 0 | 1 | 1 | 4 |
| hsa-miR-125b-2-3p | MIMAT0004603 | RBPJ | NM_203284 | 1 | 1 | 1 | 0 | 1 | 4 |
| hsa-miR-125b-2-3p | MIMAT0004603 | IL4R | NM_001257407 | 1 | 1 | 0 | 1 | 1 | 4 |
| hsa-miR-125b-2-3p | MIMAT0004603 | IL12B | NM_002187 | 1 | 1 | 0 | 1 | 1 | 4 |
| hsa-miR-125b-2-3p | MIMAT0004603 | ITGA2 | NM_002203 | 1 | 1 | 0 | 1 | 1 | 4 |
| hsa-miR-125b-2-3p | MIMAT0004603 | KCNMA1 | NM_002247 | 1 | 1 | 1 | 0 | 1 | 4 |
| hsa-miR-125b-2-3p | MIMAT0004603 | KIF11 | NM_004523 | 1 | 1 | 0 | 1 | 1 | 4 |
| hsa-miR-125b-2-3p | MIMAT0004603 | LPP | XM_005247445 | 1 | 1 | 0 | 1 | 1 | 4 |
| hsa-miR-125b-2-3p | MIMAT0004603 | MAZ | NM_001042539 | 1 | 1 | 0 | 1 | 1 | 4 |
| hsa-miR-125b-2-3p | MIMAT0004603 | MKLN1 | NM_001145354 | 1 | 1 | 0 | 1 | 1 | 4 |
| hsa-miR-125b-2-3p | MIMAT0004603 | MYO5B | NM_001080467 | 1 | 1 | 0 | 1 | 1 | 4 |
| hsa-miR-125b-2-3p | MIMAT0004603 | NRL | XM_005267708 | 1 | 1 | 0 | 1 | 1 | 4 |
| hsa-miR-125b-2-3p | MIMAT0004603 | NSF | NM_006178 | 1 | 1 | 0 | 1 | 1 | 4 |
| hsa-miR-125b-2-3p | MIMAT0004603 | NTRK2 | XM_005252001 | 1 | 1 | 0 | 1 | 1 | 4 |
| hsa-miR-125b-2-3p | MIMAT0004603 | DDR2 | XM_005245221 | 1 | 1 | 0 | 1 | 1 | 4 |
| hsa-miR-125b-2-3p | MIMAT0004603 | PALM | XM_005259566 | 1 | 1 | 0 | 1 | 1 | 4 |
| hsa-miR-125b-2-3p | MIMAT0004603 | PCBP2 | NM_005016 | 1 | 1 | 0 | 1 | 1 | 4 |
| hsa-miR-125b-2-3p | MIMAT0004603 | PDE6A | NM_000440 | 1 | 1 | 0 | 1 | 1 | 4 |
| hsa-miR-125b-2-3p | MIMAT0004603 | PGM5 | NM_021965 | 1 | 1 | 0 | 1 | 1 | 4 |
| hsa-miR-125b-2-3p | MIMAT0004603 | PHEX | NM_000444 | 1 | 1 | 0 | 1 | 1 | 4 |
| hsa-miR-125b-2-3p | MIMAT0004603 | PIK3CG | NM_002649 | 1 | 1 | 0 | 1 | 1 | 4 |
| hsa-miR-125b-2-3p | MIMAT0004603 | PI4KB | NM_001198773 | 1 | 1 | 0 | 1 | 1 | 4 |
| hsa-miR-125b-2-3p | MIMAT0004603 | PMCH | NM_002674 | 1 | 1 | 1 | 0 | 1 | 4 |
| hsa-miR-125b-2-3p | MIMAT0004603 | POLR2D | XM_005263693 | 1 | 1 | 0 | 1 | 1 | 4 |
| hsa-miR-125b-2-3p | MIMAT0004603 | POU2F1 | NM_001198783 | 1 | 1 | 0 | 1 | 1 | 4 |
| hsa-miR-125b-2-3p | MIMAT0004603 | PPA1 | NM_021129 | 1 | 1 | 0 | 1 | 1 | 4 |
| hsa-miR-125b-2-3p | MIMAT0004603 | PPIA | NM_021130 | 1 | 1 | 1 | 0 | 1 | 4 |
| hsa-miR-125b-2-3p | MIMAT0004603 | RABIF | NM_002871 | 1 | 1 | 1 | 0 | 1 | 4 |
| hsa-miR-125b-2-3p | MIMAT0004603 | RFNG | NM_002917 | 1 | 1 | 0 | 1 | 1 | 4 |
| hsa-miR-125b-2-3p | MIMAT0004603 | ABCE1 | NM_002940 | 1 | 1 | 1 | 0 | 1 | 4 |
| hsa-miR-125b-2-3p | MIMAT0004603 | RPL9 | XM_005262661 | 1 | 1 | 0 | 1 | 1 | 4 |
| hsa-miR-125b-2-3p | MIMAT0004603 | RPL28 | NM_001136134 | 1 | 1 | 0 | 1 | 1 | 4 |
| hsa-miR-125b-2-3p | MIMAT0004603 | ATXN1 | NM_000332 | 1 | 1 | 0 | 1 | 1 | 4 |
| hsa-miR-125b-2-3p | MIMAT0004603 | SCD | NM_005063 | 1 | 1 | 1 | 0 | 1 | 4 |
| hsa-miR-125b-2-3p | MIMAT0004603 | CXCL5 | NM_002994 | 1 | 1 | 0 | 1 | 1 | 4 |
| hsa-miR-125b-2-3p | MIMAT0004603 | SDC4 | NM_002999 | 1 | 1 | 0 | 1 | 1 | 4 |
| hsa-miR-125b-2-3p | MIMAT0004603 | SGCB | NM_000232 | 1 | 1 | 1 | 0 | 1 | 4 |
| hsa-miR-125b-2-3p | MIMAT0004603 | SGCD | NM_000337 | 1 | 1 | 0 | 1 | 1 | 4 |
| hsa-miR-125b-2-3p | MIMAT0004603 | ST8SIA1 | NM_003034 | 1 | 1 | 1 | 0 | 1 | 4 |
| hsa-miR-125b-2-3p | MIMAT0004603 | SLC8A3 | NM_183002 | 1 | 1 | 0 | 1 | 1 | 4 |
| hsa-miR-125b-2-3p | MIMAT0004603 | SLC19A1 | XM_005261163 | 1 | 1 | 0 | 1 | 1 | 4 |
| hsa-miR-125b-2-3p | MIMAT0004603 | HLTF | NM_003071 | 1 | 1 | 0 | 1 | 1 | 4 |
| hsa-miR-125b-2-3p | MIMAT0004603 | TROVE2 | NM_001173524 | 1 | 1 | 0 | 1 | 1 | 4 |
| hsa-miR-125b-2-3p | MIMAT0004603 | SYK | NM_003177 | 1 | 1 | 0 | 1 | 1 | 4 |
| hsa-miR-125b-2-3p | MIMAT0004603 | SYN2 | NM_003178 | 1 | 1 | 1 | 0 | 1 | 4 |
| hsa-miR-125b-2-3p | MIMAT0004603 | TAF1 | XM_005262295 | 1 | 1 | 0 | 1 | 1 | 4 |
| hsa-miR-125b-2-3p | MIMAT0004603 | THBD | NM_000361 | 1 | 1 | 0 | 1 | 1 | 4 |
| hsa-miR-125b-2-3p | MIMAT0004603 | TLL2 | NM_012465 | 1 | 1 | 0 | 1 | 1 | 4 |
| hsa-miR-125b-2-3p | MIMAT0004603 | TRPC1 | NM_001251845 | 1 | 1 | 1 | 0 | 1 | 4 |
| hsa-miR-125b-2-3p | MIMAT0004603 | TSNAX | NM_005999 | 0 | 1 | 1 | 1 | 1 | 4 |
| hsa-miR-125b-2-3p | MIMAT0004603 | UCHL1 | NM_004181 | 1 | 1 | 0 | 1 | 1 | 4 |
| hsa-miR-125b-2-3p | MIMAT0004603 | XG | XM_005274587 | 1 | 1 | 0 | 1 | 1 | 4 |
| hsa-miR-125b-2-3p | MIMAT0004603 | MKRN3 | NM_005664 | 1 | 1 | 1 | 0 | 1 | 4 |
| hsa-miR-125b-2-3p | MIMAT0004603 | ZNF200 | NM_003454 | 1 | 1 | 0 | 1 | 1 | 4 |
| hsa-miR-125b-2-3p | MIMAT0004603 | ALDH5A1 | NM_170740 | 1 | 1 | 1 | 0 | 1 | 4 |
| hsa-miR-125b-2-3p | MIMAT0004603 | SLC7A5 | NM_003486 | 1 | 1 | 0 | 1 | 1 | 4 |
| hsa-miR-125b-2-3p | MIMAT0004603 | ELL | NM_006532 | 1 | 1 | 0 | 1 | 1 | 4 |
| hsa-miR-125b-2-3p | MIMAT0004603 | LZTR1 | NM_006767 | 1 | 1 | 0 | 1 | 1 | 4 |
| hsa-miR-125b-2-3p | MIMAT0004603 | PNPLA4 | NM_004650 | 1 | 1 | 0 | 1 | 1 | 4 |
| hsa-miR-125b-2-3p | MIMAT0004603 | SMC1A | NM_001281463 | 1 | 1 | 0 | 1 | 1 | 4 |
| hsa-miR-125b-2-3p | MIMAT0004603 | SOAT2 | NM_003578 | 1 | 1 | 0 | 1 | 1 | 4 |
| hsa-miR-125b-2-3p | MIMAT0004603 | DUSP11 | NM_003584 | 1 | 1 | 1 | 0 | 1 | 4 |
| hsa-miR-125b-2-3p | MIMAT0004603 | GCM1 | NM_003643 | 1 | 1 | 0 | 1 | 1 | 4 |
| hsa-miR-125b-2-3p | MIMAT0004603 | AGPS | NM_003659 | 1 | 1 | 0 | 1 | 1 | 4 |
| hsa-miR-125b-2-3p | MIMAT0004603 | B3GALNT1 | NM_001038628 | 1 | 1 | 0 | 1 | 1 | 4 |
| hsa-miR-125b-2-3p | MIMAT0004603 | CDS2 | NM_003818 | 1 | 1 | 0 | 1 | 1 | 4 |
| hsa-miR-125b-2-3p | MIMAT0004603 | PEX11A | NM_003847 | 1 | 1 | 0 | 1 | 1 | 4 |
| hsa-miR-125b-2-3p | MIMAT0004603 | NR1I2 | NM_003889 | 1 | 1 | 0 | 1 | 1 | 4 |
| hsa-miR-125b-2-3p | MIMAT0004603 | USP13 | NM_003940 | 1 | 1 | 0 | 1 | 1 | 4 |
| hsa-miR-125b-2-3p | MIMAT0004603 | STBD1 | NM_003943 | 1 | 1 | 0 | 1 | 1 | 4 |
| hsa-miR-125b-2-3p | MIMAT0004603 | LPAR2 | NM_004720 | 1 | 1 | 0 | 1 | 1 | 4 |
| hsa-miR-125b-2-3p | MIMAT0004603 | ZMYM4 | NM_005095 | 1 | 1 | 1 | 0 | 1 | 4 |
| hsa-miR-125b-2-3p | MIMAT0004603 | ZMYM6 | XM_005271337 | 1 | 1 | 0 | 1 | 1 | 4 |
| hsa-miR-125b-2-3p | MIMAT0004603 | ZMYM5 | NM_001142684 | 1 | 1 | 1 | 0 | 1 | 4 |
| hsa-miR-125b-2-3p | MIMAT0004603 | ZNF264 | NM_003417 | 1 | 1 | 1 | 0 | 1 | 4 |
| hsa-miR-125b-2-3p | MIMAT0004603 | RASAL2 | NM_170692 | 1 | 1 | 0 | 1 | 1 | 4 |
| hsa-miR-125b-2-3p | MIMAT0004603 | ADAMTS3 | NM_014243 | 1 | 1 | 0 | 1 | 1 | 4 |
| hsa-miR-125b-2-3p | MIMAT0004603 | H6PD | XM_005263539 | 1 | 1 | 0 | 1 | 1 | 4 |
| hsa-miR-125b-2-3p | MIMAT0004603 | ABCG1 | XM_005261209 | 1 | 1 | 0 | 1 | 1 | 4 |
| hsa-miR-125b-2-3p | MIMAT0004603 | CEP57 | NM_001243776 | 0 | 1 | 1 | 1 | 1 | 4 |
| hsa-miR-125b-2-3p | MIMAT0004603 | BCLAF1 | XM_005267237 | 1 | 1 | 0 | 1 | 1 | 4 |
| hsa-miR-125b-2-3p | MIMAT0004603 | ZBTB39 | NM_014830 | 1 | 1 | 0 | 1 | 1 | 4 |
| hsa-miR-125b-2-3p | MIMAT0004603 | TECPR2 | NM_014844 | 1 | 1 | 0 | 1 | 1 | 4 |
| hsa-miR-125b-2-3p | MIMAT0004603 | KBTBD11 | NM_014867 | 1 | 1 | 0 | 1 | 1 | 4 |
| hsa-miR-125b-2-3p | MIMAT0004603 | DGCR2 | NM_005137 | 0 | 1 | 1 | 1 | 1 | 4 |
| hsa-miR-125b-2-3p | MIMAT0004603 | BCL2L10 | NM_020396 | 1 | 1 | 1 | 0 | 1 | 4 |
| hsa-miR-125b-2-3p | MIMAT0004603 | TSPAN3 | NM_005724 | 1 | 1 | 0 | 1 | 1 | 4 |
| hsa-miR-125b-2-3p | MIMAT0004603 | DHRS9 | NM_005771 | 1 | 1 | 1 | 0 | 1 | 4 |
| hsa-miR-125b-2-3p | MIMAT0004603 | APPBP2 | NM_006380 | 1 | 1 | 0 | 1 | 1 | 4 |
| hsa-miR-125b-2-3p | MIMAT0004603 | ATG7 | NM_006395 | 1 | 1 | 0 | 1 | 1 | 4 |
| hsa-miR-125b-2-3p | MIMAT0004603 | LEFTY1 | NM_020997 | 1 | 1 | 0 | 1 | 1 | 4 |
| hsa-miR-125b-2-3p | MIMAT0004603 | ZNF275 | NM_001080485 | 1 | 1 | 1 | 0 | 1 | 4 |
| hsa-miR-125b-2-3p | MIMAT0004603 | UGT2A1 | NM_006798 | 1 | 1 | 1 | 0 | 1 | 4 |
| hsa-miR-125b-2-3p | MIMAT0004603 | MSL3 | NM_078628 | 1 | 1 | 0 | 1 | 1 | 4 |
| hsa-miR-125b-2-3p | MIMAT0004603 | TMED2 | XM_005253544 | 1 | 1 | 0 | 1 | 1 | 4 |
| hsa-miR-125b-2-3p | MIMAT0004603 | MAPRE2 | NM_001143827 | 1 | 1 | 0 | 1 | 1 | 4 |
| hsa-miR-125b-2-3p | MIMAT0004603 | IMMT | XM_005264108 | 1 | 1 | 0 | 1 | 1 | 4 |
| hsa-miR-125b-2-3p | MIMAT0004603 | SLC35D2 | NM_007001 | 1 | 1 | 0 | 1 | 1 | 4 |
| hsa-miR-125b-2-3p | MIMAT0004603 | FGFR1OP | NM_007045 | 1 | 1 | 0 | 1 | 1 | 4 |
| hsa-miR-125b-2-3p | MIMAT0004603 | POLR3A | NM_007055 | 0 | 1 | 1 | 1 | 1 | 4 |
| hsa-miR-125b-2-3p | MIMAT0004603 | LDB3 | XM_005269464 | 1 | 1 | 0 | 1 | 1 | 4 |
| hsa-miR-125b-2-3p | MIMAT0004603 | PADI2 | NM_007365 | 1 | 1 | 0 | 1 | 1 | 4 |
| hsa-miR-125b-2-3p | MIMAT0004603 | CHP1 | NM_007236 | 1 | 1 | 0 | 1 | 1 | 4 |
| hsa-miR-125b-2-3p | MIMAT0004603 | DNAJC8 | NM_014280 | 1 | 1 | 0 | 1 | 1 | 4 |
| hsa-miR-125b-2-3p | MIMAT0004603 | RHOBTB3 | NM_014899 | 1 | 1 | 0 | 1 | 1 | 4 |
| hsa-miR-125b-2-3p | MIMAT0004603 | AAK1 | NM_014911 | 1 | 1 | 1 | 0 | 1 | 4 |
| hsa-miR-125b-2-3p | MIMAT0004603 | PLA2R1 | NM_001007267 | 1 | 1 | 1 | 0 | 1 | 4 |
| hsa-miR-125b-2-3p | MIMAT0004603 | FBXO21 | XM_005253857 | 1 | 1 | 0 | 1 | 1 | 4 |
| hsa-miR-125b-2-3p | MIMAT0004603 | FNBP1 | XM_005251817 | 1 | 1 | 0 | 1 | 1 | 4 |
| hsa-miR-125b-2-3p | MIMAT0004603 | ZHX3 | XM_005260343 | 1 | 1 | 0 | 1 | 1 | 4 |
| hsa-miR-125b-2-3p | MIMAT0004603 | SWAP70 | NM_015055 | 1 | 1 | 1 | 0 | 1 | 4 |
| hsa-miR-125b-2-3p | MIMAT0004603 | MYCBP2 | NM_015057 | 1 | 1 | 1 | 0 | 1 | 4 |
| hsa-miR-125b-2-3p | MIMAT0004603 | TBC1D2B | NM_144572 | 1 | 1 | 0 | 1 | 1 | 4 |
| hsa-miR-125b-2-3p | MIMAT0004603 | EFR3A | NM_015137 | 1 | 1 | 0 | 1 | 1 | 4 |
| hsa-miR-125b-2-3p | MIMAT0004603 | GPD1L | NM_015141 | 0 | 1 | 1 | 1 | 1 | 4 |
| hsa-miR-125b-2-3p | MIMAT0004603 | JMJD6 | NM_001081461 | 1 | 1 | 0 | 1 | 1 | 4 |
| hsa-miR-125b-2-3p | MIMAT0004603 | SMCHD1 | NM_015295 | 1 | 1 | 0 | 1 | 1 | 4 |
| hsa-miR-125b-2-3p | MIMAT0004603 | KHNYN | NM_015299 | 1 | 1 | 0 | 1 | 1 | 4 |
| hsa-miR-125b-2-3p | MIMAT0004603 | SIK3 | XM_005271481 | 1 | 1 | 0 | 1 | 1 | 4 |
| hsa-miR-125b-2-3p | MIMAT0004603 | SEZ6L | NM_021115 | 1 | 1 | 0 | 1 | 1 | 4 |
| hsa-miR-125b-2-3p | MIMAT0004603 | ATP6V0A2 | NM_012463 | 1 | 1 | 0 | 1 | 1 | 4 |
| hsa-miR-125b-2-3p | MIMAT0004603 | FLRT3 | XM_005260682 | 1 | 1 | 0 | 1 | 1 | 4 |
| hsa-miR-125b-2-3p | MIMAT0004603 | BCL2L13 | NM_015367 | 1 | 1 | 0 | 1 | 1 | 4 |
| hsa-miR-125b-2-3p | MIMAT0004603 | DSTYK | NM_015375 | 0 | 1 | 1 | 1 | 1 | 4 |
| hsa-miR-125b-2-3p | MIMAT0004603 | ZNF345 | NM_003419 | 1 | 1 | 1 | 0 | 1 | 4 |
| hsa-miR-125b-2-3p | MIMAT0004603 | STAP1 | XM_005265675 | 1 | 1 | 0 | 1 | 1 | 4 |
| hsa-miR-125b-2-3p | MIMAT0004603 | PCDH11X | NM_032968 | 1 | 1 | 0 | 1 | 1 | 4 |
| hsa-miR-125b-2-3p | MIMAT0004603 | RABGEF1 | XM_005250280 | 1 | 1 | 0 | 1 | 1 | 4 |
| hsa-miR-125b-2-3p | MIMAT0004603 | TMOD2 | NM_014548 | 1 | 1 | 0 | 1 | 1 | 4 |
| hsa-miR-125b-2-3p | MIMAT0004603 | TRPM5 | NM_014555 | 1 | 1 | 0 | 1 | 1 | 4 |
| hsa-miR-125b-2-3p | MIMAT0004603 | MDFIC | NM_001166345 | 1 | 1 | 1 | 0 | 1 | 4 |
| hsa-miR-125b-2-3p | MIMAT0004603 | SLC40A1 | XM_005246505 | 1 | 1 | 0 | 1 | 1 | 4 |
| hsa-miR-125b-2-3p | MIMAT0004603 | CHST11 | NM_018413 | 1 | 1 | 0 | 1 | 1 | 4 |
| hsa-miR-125b-2-3p | MIMAT0004603 | F11R | NM_016946 | 1 | 1 | 0 | 1 | 1 | 4 |
| hsa-miR-125b-2-3p | MIMAT0004603 | TMED5 | NM_001167830 | 1 | 1 | 0 | 1 | 1 | 4 |
| hsa-miR-125b-2-3p | MIMAT0004603 | POLR1D | XM_005266414 | 1 | 1 | 0 | 1 | 1 | 4 |
| hsa-miR-125b-2-3p | MIMAT0004603 | DACT1 | NM_016651 | 1 | 1 | 0 | 1 | 1 | 4 |
| hsa-miR-125b-2-3p | MIMAT0004603 | NT5DC3 | NM_001031701 | 1 | 1 | 0 | 1 | 1 | 4 |
| hsa-miR-125b-2-3p | MIMAT0004603 | ZAK | NM_016653 | 1 | 1 | 1 | 0 | 1 | 4 |
| hsa-miR-125b-2-3p | MIMAT0004603 | S1PR5 | XM_005259937 | 1 | 1 | 0 | 1 | 1 | 4 |
| hsa-miR-125b-2-3p | MIMAT0004603 | CSNK1G1 | NM_022048 | 1 | 1 | 0 | 1 | 1 | 4 |
| hsa-miR-125b-2-3p | MIMAT0004603 | BRWD1 | NM_033656 | 1 | 1 | 0 | 1 | 1 | 4 |
| hsa-miR-125b-2-3p | MIMAT0004603 | KLHL28 | NM_017658 | 1 | 1 | 1 | 0 | 1 | 4 |
| hsa-miR-125b-2-3p | MIMAT0004603 | ELOVL2 | NM_017770 | 1 | 1 | 0 | 1 | 1 | 4 |
| hsa-miR-125b-2-3p | MIMAT0004603 | FAM208B | NM_017782 | 1 | 1 | 1 | 0 | 1 | 4 |
| hsa-miR-125b-2-3p | MIMAT0004603 | CMTM6 | NM_017801 | 1 | 1 | 0 | 1 | 1 | 4 |
| hsa-miR-125b-2-3p | MIMAT0004603 | MED9 | NM_018019 | 1 | 1 | 0 | 1 | 1 | 4 |
| hsa-miR-125b-2-3p | MIMAT0004603 | RALGPS2 | XM_005245297 | 1 | 1 | 0 | 1 | 1 | 4 |
| hsa-miR-125b-2-3p | MIMAT0004603 | GOLPH3L | NM_018178 | 1 | 1 | 1 | 0 | 1 | 4 |
| hsa-miR-125b-2-3p | MIMAT0004603 | SETD5 | XM_005265299 | 1 | 1 | 0 | 1 | 1 | 4 |
| hsa-miR-125b-2-3p | MIMAT0004603 | RAVER2 | NM_018211 | 1 | 1 | 0 | 1 | 1 | 4 |
| hsa-miR-125b-2-3p | MIMAT0004603 | ASXL2 | NM_018263 | 1 | 1 | 0 | 1 | 1 | 4 |
| hsa-miR-125b-2-3p | MIMAT0004603 | ECHDC2 | NM_001198962 | 1 | 1 | 1 | 0 | 1 | 4 |
| hsa-miR-125b-2-3p | MIMAT0004603 | VPS53 | NM_001128159 | 1 | 1 | 0 | 1 | 1 | 4 |
| hsa-miR-125b-2-3p | MIMAT0004603 | MIS18BP1 | NM_018353 | 1 | 1 | 1 | 0 | 1 | 4 |
| hsa-miR-125b-2-3p | MIMAT0004603 | BMP2K | XM_005263117 | 1 | 1 | 0 | 1 | 1 | 4 |
| hsa-miR-125b-2-3p | MIMAT0004603 | KRBOX4 | NM_001129899 | 0 | 1 | 1 | 1 | 1 | 4 |
| hsa-miR-125b-2-3p | MIMAT0004603 | PEX26 | NM_001127649 | 1 | 1 | 0 | 1 | 1 | 4 |
| hsa-miR-125b-2-3p | MIMAT0004603 | KDM4D | NM_018039 | 1 | 1 | 0 | 1 | 1 | 4 |
| hsa-miR-125b-2-3p | MIMAT0004603 | TENM3 | NM_001080477 | 1 | 1 | 1 | 0 | 1 | 4 |
| hsa-miR-125b-2-3p | MIMAT0004603 | VPS35 | NM_018206 | 1 | 1 | 1 | 0 | 1 | 4 |
| hsa-miR-125b-2-3p | MIMAT0004603 | TMEM30A | NM_018247 | 1 | 1 | 0 | 1 | 1 | 4 |
| hsa-miR-125b-2-3p | MIMAT0004603 | DCP1A | NM_018403 | 1 | 1 | 1 | 0 | 1 | 4 |
| hsa-miR-125b-2-3p | MIMAT0004603 | LANCL2 | NM_018697 | 1 | 1 | 0 | 1 | 1 | 4 |
| hsa-miR-125b-2-3p | MIMAT0004603 | AGPAT4 | NM_020133 | 1 | 1 | 0 | 1 | 1 | 4 |
| hsa-miR-125b-2-3p | MIMAT0004603 | TMEM167B | NM_020141 | 1 | 1 | 0 | 1 | 1 | 4 |
| hsa-miR-125b-2-3p | MIMAT0004603 | C1GALT1 | NM_020156 | 1 | 1 | 1 | 0 | 1 | 4 |
| hsa-miR-125b-2-3p | MIMAT0004603 | PDXP | NM_020315 | 1 | 1 | 0 | 1 | 1 | 4 |
| hsa-miR-125b-2-3p | MIMAT0004603 | MTA3 | XM_005264459 | 1 | 1 | 0 | 1 | 1 | 4 |
| hsa-miR-125b-2-3p | MIMAT0004603 | KCNT1 | XM_005263407 | 1 | 1 | 0 | 1 | 1 | 4 |
| hsa-miR-125b-2-3p | MIMAT0004603 | KIAA1462 | NM_020848 | 1 | 1 | 0 | 1 | 1 | 4 |
| hsa-miR-125b-2-3p | MIMAT0004603 | RBAK | NM_021163 | 1 | 1 | 0 | 1 | 1 | 4 |
| hsa-miR-125b-2-3p | MIMAT0004603 | BCORL1 | XM_005262452 | 1 | 1 | 0 | 1 | 1 | 4 |
| hsa-miR-125b-2-3p | MIMAT0004603 | MRPS14 | NM_022100 | 1 | 1 | 0 | 1 | 1 | 4 |
| hsa-miR-125b-2-3p | MIMAT0004603 | CRTC3 | NM_022769 | 1 | 1 | 0 | 1 | 1 | 4 |
| hsa-miR-125b-2-3p | MIMAT0004603 | DCLRE1B | NM_022836 | 1 | 1 | 0 | 1 | 1 | 4 |
| hsa-miR-125b-2-3p | MIMAT0004603 | TMEM237 | NM_152388 | 1 | 1 | 0 | 1 | 1 | 4 |
| hsa-miR-125b-2-3p | MIMAT0004603 | MTMR9 | NM_015458 | 1 | 1 | 0 | 1 | 1 | 4 |
| hsa-miR-125b-2-3p | MIMAT0004603 | GID4 | NM_024052 | 1 | 1 | 0 | 1 | 1 | 4 |
| hsa-miR-125b-2-3p | MIMAT0004603 | TAF1D | NM_024116 | 1 | 1 | 1 | 0 | 1 | 4 |
| hsa-miR-125b-2-3p | MIMAT0004603 | BRCC3 | NM_024332 | 1 | 1 | 1 | 0 | 1 | 4 |
| hsa-miR-125b-2-3p | MIMAT0004603 | DCAF10 | NM_024345 | 1 | 1 | 0 | 1 | 1 | 4 |
| hsa-miR-125b-2-3p | MIMAT0004603 | CCNJL | NM_024565 | 1 | 1 | 0 | 1 | 1 | 4 |
| hsa-miR-125b-2-3p | MIMAT0004603 | ARHGAP10 | XM_005263214 | 1 | 1 | 0 | 1 | 1 | 4 |
| hsa-miR-125b-2-3p | MIMAT0004603 | PARP8 | NM_001178055 | 1 | 1 | 0 | 1 | 1 | 4 |
| hsa-miR-125b-2-3p | MIMAT0004603 | VASH2 | NM_001136475 | 1 | 1 | 1 | 0 | 1 | 4 |
| hsa-miR-125b-2-3p | MIMAT0004603 | GSTCD | NM_024751 | 1 | 1 | 1 | 0 | 1 | 4 |
| hsa-miR-125b-2-3p | MIMAT0004603 | MMRN2 | XM_005270153 | 1 | 1 | 0 | 1 | 1 | 4 |
| hsa-miR-125b-2-3p | MIMAT0004603 | TREML2 | NM_024807 | 1 | 1 | 0 | 1 | 1 | 4 |
| hsa-miR-125b-2-3p | MIMAT0004603 | THSD4 | NM_024817 | 1 | 1 | 0 | 1 | 1 | 4 |
| hsa-miR-125b-2-3p | MIMAT0004603 | CNTNAP3 | NM_033655 | 1 | 1 | 1 | 0 | 1 | 4 |
| hsa-miR-125b-2-3p | MIMAT0004603 | SLC35E1 | NM_024881 | 1 | 1 | 0 | 1 | 1 | 4 |
| hsa-miR-125b-2-3p | MIMAT0004603 | C14orf159 | NM_001102366 | 1 | 1 | 0 | 1 | 1 | 4 |
| hsa-miR-125b-2-3p | MIMAT0004603 | ZNF614 | NM_025040 | 1 | 1 | 0 | 1 | 1 | 4 |
| hsa-miR-125b-2-3p | MIMAT0004603 | WDR82 | NM_025222 | 1 | 1 | 0 | 1 | 1 | 4 |
| hsa-miR-125b-2-3p | MIMAT0004603 | SRCIN1 | NM_025248 | 1 | 1 | 0 | 1 | 1 | 4 |
| hsa-miR-125b-2-3p | MIMAT0004603 | ANP32E | XM_005245513 | 1 | 1 | 0 | 1 | 1 | 4 |
| hsa-miR-125b-2-3p | MIMAT0004603 | SBF2 | NM_030962 | 1 | 1 | 1 | 0 | 1 | 4 |
| hsa-miR-125b-2-3p | MIMAT0004603 | PCDH11Y | NM_032973 | 1 | 1 | 0 | 1 | 1 | 4 |
| hsa-miR-125b-2-3p | MIMAT0004603 | SYT16 | XM_005268123 | 1 | 1 | 0 | 1 | 1 | 4 |
| hsa-miR-125b-2-3p | MIMAT0004603 | ANKRD27 | NM_032139 | 1 | 1 | 0 | 1 | 1 | 4 |
| hsa-miR-125b-2-3p | MIMAT0004603 | TMEM164 | XM_005262206 | 1 | 1 | 0 | 1 | 1 | 4 |
| hsa-miR-125b-2-3p | MIMAT0004603 | DCUN1D5 | NM_032299 | 1 | 1 | 1 | 0 | 1 | 4 |
| hsa-miR-125b-2-3p | MIMAT0004603 | MCM8 | NM_001281521 | 1 | 1 | 0 | 1 | 1 | 4 |
| hsa-miR-125b-2-3p | MIMAT0004603 | SFT2D3 | NM_032740 | 1 | 1 | 0 | 1 | 1 | 4 |
| hsa-miR-125b-2-3p | MIMAT0004603 | TMTC4 | NM_032813 | 1 | 1 | 0 | 1 | 1 | 4 |
| hsa-miR-125b-2-3p | MIMAT0004603 | KIAA1644 | XM_005261790 | 1 | 1 | 0 | 1 | 1 | 4 |
| hsa-miR-125b-2-3p | MIMAT0004603 | KIAA1755 | NM_001029864 | 1 | 1 | 0 | 1 | 1 | 4 |
| hsa-miR-125b-2-3p | MIMAT0004603 | EFCAB11 | NM_145231 | 1 | 1 | 1 | 0 | 1 | 4 |
| hsa-miR-125b-2-3p | MIMAT0004603 | L3MBTL4 | XM_005258168 | 1 | 1 | 0 | 1 | 1 | 4 |
| hsa-miR-125b-2-3p | MIMAT0004603 | MYOZ3 | NM_001122853 | 1 | 1 | 0 | 1 | 1 | 4 |
| hsa-miR-125b-2-3p | MIMAT0004603 | SYTL5 | NM_001163335 | 0 | 1 | 1 | 1 | 1 | 4 |
| hsa-miR-125b-2-3p | MIMAT0004603 | SLC46A1 | NM_080669 | 1 | 1 | 0 | 1 | 1 | 4 |
| hsa-miR-125b-2-3p | MIMAT0004603 | BTBD9 | XM_005248841 | 1 | 1 | 0 | 1 | 1 | 4 |
| hsa-miR-125b-2-3p | MIMAT0004603 | PNMA5 | NM_001103150 | 1 | 1 | 0 | 1 | 1 | 4 |
| hsa-miR-125b-2-3p | MIMAT0004603 | C1QTNF3 | NM_181435 | 1 | 1 | 1 | 0 | 1 | 4 |
| hsa-miR-125b-2-3p | MIMAT0004603 | GBP4 | NM_052941 | 1 | 1 | 0 | 1 | 1 | 4 |
| hsa-miR-125b-2-3p | MIMAT0004603 | SSX2IP | NM_001166417 | 1 | 1 | 0 | 1 | 1 | 4 |
| hsa-miR-125b-2-3p | MIMAT0004603 | IQCK | NM_153208 | 1 | 1 | 0 | 1 | 1 | 4 |
| hsa-miR-125b-2-3p | MIMAT0004603 | EARS2 | NM_001083614 | 1 | 1 | 0 | 1 | 1 | 4 |
| hsa-miR-125b-2-3p | MIMAT0004603 | COX6B2 | NM_144613 | 1 | 1 | 1 | 0 | 1 | 4 |
| hsa-miR-125b-2-3p | MIMAT0004603 | PIFO | XM_005270472 | 1 | 1 | 0 | 1 | 1 | 4 |
| hsa-miR-125b-2-3p | MIMAT0004603 | LRRC15 | NM_001135057 | 1 | 1 | 0 | 1 | 1 | 4 |
| hsa-miR-125b-2-3p | MIMAT0004603 | DPCR1 | NM_080870 | 1 | 1 | 0 | 1 | 1 | 4 |
| hsa-miR-125b-2-3p | MIMAT0004603 | HGSNAT | XM_005273409 | 1 | 1 | 0 | 1 | 1 | 4 |
| hsa-miR-125b-2-3p | MIMAT0004603 | BEND2 | NM_153346 | 1 | 1 | 0 | 1 | 1 | 4 |
| hsa-miR-125b-2-3p | MIMAT0004603 | GAB3 | NM_001081573 | 1 | 1 | 0 | 1 | 1 | 4 |
| hsa-miR-125b-2-3p | MIMAT0004603 | ALG10B | NM_001013620 | 1 | 1 | 1 | 0 | 1 | 4 |
| hsa-miR-125b-2-3p | MIMAT0004603 | DPY19L3 | NM_207325 | 1 | 1 | 0 | 1 | 1 | 4 |
| hsa-miR-125b-2-3p | MIMAT0004603 | ZNF558 | XM_005259755 | 1 | 1 | 0 | 1 | 1 | 4 |
| hsa-miR-125b-2-3p | MIMAT0004603 | SYT6 | NM_001270805 | 1 | 1 | 0 | 1 | 1 | 4 |
| hsa-miR-125b-2-3p | MIMAT0004603 | B3GALNT2 | NM_152490 | 1 | 1 | 1 | 0 | 1 | 4 |
| hsa-miR-125b-2-3p | MIMAT0004603 | KLB | NM_175737 | 1 | 1 | 0 | 1 | 1 | 4 |
| hsa-miR-125b-2-3p | MIMAT0004603 | ATP6V0E2 | NM_145230 | 1 | 1 | 0 | 1 | 1 | 4 |
| hsa-miR-125b-2-3p | MIMAT0004603 | RDH10 | NM_172037 | 0 | 1 | 1 | 1 | 1 | 4 |
| hsa-miR-125b-2-3p | MIMAT0004603 | SLC35G1 | XM_005269584 | 1 | 1 | 0 | 1 | 1 | 4 |
| hsa-miR-125b-2-3p | MIMAT0004603 | CLEC14A | NM_175060 | 0 | 1 | 1 | 1 | 1 | 4 |
| hsa-miR-125b-2-3p | MIMAT0004603 | IFNLR1 | NM_170743 | 1 | 1 | 0 | 1 | 1 | 4 |
| hsa-miR-125b-2-3p | MIMAT0004603 | PPIAL4A | NM_178230 | 1 | 1 | 0 | 1 | 1 | 4 |
| hsa-miR-125b-2-3p | MIMAT0004603 | KBTBD12 | XM_005247167 | 1 | 1 | 0 | 1 | 1 | 4 |
| hsa-miR-125b-2-3p | MIMAT0004603 | OLFML2A | NM_182487 | 1 | 1 | 0 | 1 | 1 | 4 |
| hsa-miR-125b-2-3p | MIMAT0004603 | SYNPO2 | NM_001128933 | 1 | 1 | 0 | 1 | 1 | 4 |
| hsa-miR-125b-2-3p | MIMAT0004603 | METTL15 | NM_001113528 | 1 | 1 | 1 | 0 | 1 | 4 |
| hsa-miR-125b-2-3p | MIMAT0004603 | ZNF627 | NM_145295 | 1 | 1 | 0 | 1 | 1 | 4 |
| hsa-miR-125b-2-3p | MIMAT0004603 | TXLNA | NM_175852 | 1 | 1 | 0 | 1 | 1 | 4 |
| hsa-miR-125b-2-3p | MIMAT0004603 | ANKS6 | NM_173551 | 1 | 1 | 0 | 1 | 1 | 4 |
| hsa-miR-125b-2-3p | MIMAT0004603 | VMA21 | NM_001017980 | 1 | 1 | 0 | 1 | 1 | 4 |
| hsa-miR-125b-2-3p | MIMAT0004603 | BEND6 | XM_005248890 | 1 | 1 | 0 | 1 | 1 | 4 |
| hsa-miR-125b-2-3p | MIMAT0004603 | FOXK1 | NM_001037165 | 1 | 1 | 0 | 1 | 1 | 4 |
| hsa-miR-125b-2-3p | MIMAT0004603 | ATP6V1C2 | NM_001039362 | 1 | 1 | 1 | 0 | 1 | 4 |
| hsa-miR-125b-2-3p | MIMAT0004603 | MSRB3 | NM_001193460 | 0 | 1 | 1 | 1 | 1 | 4 |
| hsa-miR-125b-2-3p | MIMAT0004603 | UBN2 | NM_173569 | 1 | 1 | 1 | 0 | 1 | 4 |
| hsa-miR-125b-2-3p | MIMAT0004603 | SCML4 | NM_198081 | 1 | 1 | 1 | 0 | 1 | 4 |
| hsa-miR-125b-2-3p | MIMAT0004603 | ZNF493 | NM_001076678 | 1 | 1 | 0 | 1 | 1 | 4 |
| hsa-miR-125b-2-3p | MIMAT0004603 | RPUSD3 | NM_173659 | 1 | 1 | 0 | 1 | 1 | 4 |
| hsa-miR-125b-2-3p | MIMAT0004603 | GPRIN3 | XM_005262936 | 1 | 1 | 0 | 1 | 1 | 4 |
| hsa-miR-125b-2-3p | MIMAT0004603 | PPIL6 | NM_001111298 | 1 | 1 | 0 | 1 | 1 | 4 |
| hsa-miR-125b-2-3p | MIMAT0004603 | DPY19L4 | NM_181787 | 1 | 1 | 0 | 1 | 1 | 4 |
| hsa-miR-125b-2-3p | MIMAT0004603 | KRTAP8-1 | NM_175857 | 1 | 1 | 0 | 1 | 1 | 4 |
| hsa-miR-125b-2-3p | MIMAT0004603 | C12orf74 | NM_001178097 | 1 | 1 | 0 | 1 | 1 | 4 |
| hsa-miR-125b-2-3p | MIMAT0004603 | NAT8L | NM_178557 | 1 | 1 | 0 | 1 | 1 | 4 |
| hsa-miR-125b-2-3p | MIMAT0004603 | GLDN | NM_181789 | 1 | 1 | 0 | 1 | 1 | 4 |
| hsa-miR-125b-2-3p | MIMAT0004603 | ZKSCAN2 | XM_005255303 | 1 | 1 | 0 | 1 | 1 | 4 |
| hsa-miR-125b-2-3p | MIMAT0004603 | PDDC1 | NM_182612 | 1 | 1 | 1 | 0 | 1 | 4 |
| hsa-miR-125b-2-3p | MIMAT0004603 | C12orf75 | NM_001145199 | 1 | 1 | 1 | 0 | 1 | 4 |
| hsa-miR-125b-2-3p | MIMAT0004603 | FAM211B | NM_207644 | 1 | 1 | 0 | 1 | 1 | 4 |
| hsa-miR-125b-2-3p | MIMAT0004603 | TEX19 | XM_005256369 | 1 | 1 | 0 | 1 | 1 | 4 |
| hsa-miR-125b-2-3p | MIMAT0004603 | NCMAP | NM_001010980 | 1 | 1 | 0 | 1 | 1 | 4 |
| hsa-miR-125b-2-3p | MIMAT0004603 | DEFB132 | NM_207469 | 1 | 1 | 0 | 1 | 1 | 4 |
| hsa-miR-125b-2-3p | MIMAT0004603 | SLC25A53 | NM_001012755 | 1 | 1 | 0 | 1 | 1 | 4 |
| hsa-miR-125b-2-3p | MIMAT0004603 | FNDC9 | NM_001001343 | 1 | 1 | 0 | 1 | 1 | 4 |
| hsa-miR-125b-2-3p | MIMAT0004603 | ZNF705A | XM_005253377 | 1 | 1 | 0 | 1 | 1 | 4 |
| hsa-miR-125b-2-3p | MIMAT0004603 | UGT2A2 | NM_001105677 | 1 | 1 | 1 | 0 | 1 | 4 |
| hsa-miR-125b-2-3p | MIMAT0004603 | TMPPE | NM_001039770 | 1 | 1 | 0 | 1 | 1 | 4 |
| hsa-miR-125b-2-3p | MIMAT0004603 | PPIAL4G | NM_001123068 | 1 | 1 | 0 | 1 | 1 | 4 |
| hsa-miR-125b-2-3p | MIMAT0004603 | PPIAL4B | NM_001143883 | 1 | 1 | 0 | 1 | 1 | 4 |
| hsa-miR-125b-2-3p | MIMAT0004603 | PPIAL4C | NM_001135789 | 1 | 1 | 0 | 1 | 1 | 4 |
| hsa-miR-125b-2-3p | MIMAT0004603 | TMEM170B | NM_001100829 | 1 | 1 | 0 | 1 | 1 | 4 |
| hsa-miR-125b-2-3p | MIMAT0004603 | FAM47E-STBD1 | NM_001242939 | 1 | 1 | 0 | 1 | 1 | 4 |
| hsa-miR-125b-2-3p | MIMAT0004603 | MYZAP | NM_152451 | 1 | 1 | 1 | 0 | 1 | 4 |
| hsa-miR-125b-2-3p | MIMAT0004603 | ABCA1 | NM_005502 | 0 | 1 | 0 | 1 | 1 | 3 |
| hsa-miR-125b-2-3p | MIMAT0004603 | ABL1 | XM_005272177 | 1 | 0 | 0 | 1 | 1 | 3 |
| hsa-miR-125b-2-3p | MIMAT0004603 | ACADSB | NM_001609 | 1 | 0 | 0 | 1 | 1 | 3 |
| hsa-miR-125b-2-3p | MIMAT0004603 | ACVR2A | NM_001278579 | 1 | 0 | 0 | 1 | 1 | 3 |
| hsa-miR-125b-2-3p | MIMAT0004603 | ADAR | NM_001193495 | 1 | 0 | 0 | 1 | 1 | 3 |
| hsa-miR-125b-2-3p | MIMAT0004603 | ADCYAP1 | XM_005258081 | 1 | 1 | 0 | 0 | 1 | 3 |
| hsa-miR-125b-2-3p | MIMAT0004603 | ADH5 | NM_000671 | 1 | 0 | 0 | 1 | 1 | 3 |
| hsa-miR-125b-2-3p | MIMAT0004603 | ADRA2B | NM_000682 | 1 | 0 | 0 | 1 | 1 | 3 |
| hsa-miR-125b-2-3p | MIMAT0004603 | AP2B1 | XM_005257940 | 0 | 1 | 0 | 1 | 1 | 3 |
| hsa-miR-125b-2-3p | MIMAT0004603 | AGT | NM_000029 | 1 | 0 | 0 | 1 | 1 | 3 |
| hsa-miR-125b-2-3p | MIMAT0004603 | ALCAM | NM_001627 | 1 | 1 | 0 | 0 | 1 | 3 |
| hsa-miR-125b-2-3p | MIMAT0004603 | ALDH3A2 | NM_001031806 | 1 | 0 | 0 | 1 | 1 | 3 |
| hsa-miR-125b-2-3p | MIMAT0004603 | APBB2 | NM_004307 | 0 | 1 | 0 | 1 | 1 | 3 |
| hsa-miR-125b-2-3p | MIMAT0004603 | APC | XM_005271975 | 1 | 0 | 0 | 1 | 1 | 3 |
| hsa-miR-125b-2-3p | MIMAT0004603 | BIRC2 | NM_001256163 | 0 | 1 | 0 | 1 | 1 | 3 |
| hsa-miR-125b-2-3p | MIMAT0004603 | XIAP | NM_001167 | 0 | 1 | 0 | 1 | 1 | 3 |
| hsa-miR-125b-2-3p | MIMAT0004603 | AQP4 | XM_005258257 | 1 | 0 | 0 | 1 | 1 | 3 |
| hsa-miR-125b-2-3p | MIMAT0004603 | ARCN1 | NM_001655 | 0 | 1 | 0 | 1 | 1 | 3 |
| hsa-miR-125b-2-3p | MIMAT0004603 | TRIM23 | NM_001656 | 1 | 0 | 0 | 1 | 1 | 3 |
| hsa-miR-125b-2-3p | MIMAT0004603 | ASPH | XM_005251235 | 1 | 1 | 0 | 0 | 1 | 3 |
| hsa-miR-125b-2-3p | MIMAT0004603 | ATP7A | XM_005262147 | 1 | 0 | 0 | 1 | 1 | 3 |
| hsa-miR-125b-2-3p | MIMAT0004603 | BARD1 | NM_000465 | 1 | 1 | 0 | 0 | 1 | 3 |
| hsa-miR-125b-2-3p | MIMAT0004603 | BCL2L1 | NM_138578 | 1 | 0 | 0 | 1 | 1 | 3 |
| hsa-miR-125b-2-3p | MIMAT0004603 | ZFP36L2 | NM_006887 | 1 | 0 | 0 | 1 | 1 | 3 |
| hsa-miR-125b-2-3p | MIMAT0004603 | CACNA2D1 | XM_005250569 | 1 | 0 | 0 | 1 | 1 | 3 |
| hsa-miR-125b-2-3p | MIMAT0004603 | CAMLG | NM_001745 | 1 | 0 | 0 | 1 | 1 | 3 |
| hsa-miR-125b-2-3p | MIMAT0004603 | CASQ1 | NM_001231 | 1 | 0 | 0 | 1 | 1 | 3 |
| hsa-miR-125b-2-3p | MIMAT0004603 | RUNX1T1 | NM_001198625 | 1 | 0 | 0 | 1 | 1 | 3 |
| hsa-miR-125b-2-3p | MIMAT0004603 | CBLB | XM_005247854 | 0 | 1 | 0 | 1 | 1 | 3 |
| hsa-miR-125b-2-3p | MIMAT0004603 | CCNB1 | NM_031966 | 1 | 0 | 0 | 1 | 1 | 3 |
| hsa-miR-125b-2-3p | MIMAT0004603 | SCARB2 | NM_005506 | 0 | 1 | 0 | 1 | 1 | 3 |
| hsa-miR-125b-2-3p | MIMAT0004603 | CD47 | NM_001777 | 0 | 1 | 1 | 0 | 1 | 3 |
| hsa-miR-125b-2-3p | MIMAT0004603 | CDH6 | NM_004932 | 1 | 0 | 0 | 1 | 1 | 3 |
| hsa-miR-125b-2-3p | MIMAT0004603 | CDKN2D | NM_001800 | 0 | 1 | 0 | 1 | 1 | 3 |
| hsa-miR-125b-2-3p | MIMAT0004603 | CEBPG | NM_001806 | 0 | 1 | 0 | 1 | 1 | 3 |
| hsa-miR-125b-2-3p | MIMAT0004603 | CHRNE | NM_000080 | 1 | 0 | 0 | 1 | 1 | 3 |
| hsa-miR-125b-2-3p | MIMAT0004603 | CREM | NM_183013 | 1 | 1 | 0 | 0 | 1 | 3 |
| hsa-miR-125b-2-3p | MIMAT0004603 | CSE1L | NM_001316 | 0 | 1 | 1 | 0 | 1 | 3 |
| hsa-miR-125b-2-3p | MIMAT0004603 | CSNK1G3 | XM_005271892 | 1 | 1 | 0 | 0 | 1 | 3 |
| hsa-miR-125b-2-3p | MIMAT0004603 | CXADR | NM_001338 | 1 | 0 | 0 | 1 | 1 | 3 |
| hsa-miR-125b-2-3p | MIMAT0004603 | CYP7A1 | NM_000780 | 1 | 0 | 0 | 1 | 1 | 3 |
| hsa-miR-125b-2-3p | MIMAT0004603 | DACH1 | NM_080759 | 0 | 1 | 0 | 1 | 1 | 3 |
| hsa-miR-125b-2-3p | MIMAT0004603 | DHX15 | NM_001358 | 0 | 1 | 1 | 0 | 1 | 3 |
| hsa-miR-125b-2-3p | MIMAT0004603 | TIMM8A | NM_004085 | 1 | 0 | 0 | 1 | 1 | 3 |
| hsa-miR-125b-2-3p | MIMAT0004603 | DLX1 | NM_178120 | 1 | 0 | 0 | 1 | 1 | 3 |
| hsa-miR-125b-2-3p | MIMAT0004603 | DMD | NM_004010 | 1 | 0 | 0 | 1 | 1 | 3 |
| hsa-miR-125b-2-3p | MIMAT0004603 | DPH1 | XM_005256495 | 1 | 1 | 0 | 0 | 1 | 3 |
| hsa-miR-125b-2-3p | MIMAT0004603 | TSC22D3 | NM_198057 | 0 | 1 | 0 | 1 | 1 | 3 |
| hsa-miR-125b-2-3p | MIMAT0004603 | HBEGF | NM_001945 | 0 | 1 | 0 | 1 | 1 | 3 |
| hsa-miR-125b-2-3p | MIMAT0004603 | EGR1 | NM_001964 | 0 | 1 | 1 | 0 | 1 | 3 |
| hsa-miR-125b-2-3p | MIMAT0004603 | EHHADH | NM_001166415 | 0 | 1 | 0 | 1 | 1 | 3 |
| hsa-miR-125b-2-3p | MIMAT0004603 | EIF4EBP2 | NM_004096 | 0 | 1 | 0 | 1 | 1 | 3 |
| hsa-miR-125b-2-3p | MIMAT0004603 | ELAVL1 | NM_001419 | 1 | 0 | 0 | 1 | 1 | 3 |
| hsa-miR-125b-2-3p | MIMAT0004603 | STOM | NM_004099 | 1 | 0 | 0 | 1 | 1 | 3 |
| hsa-miR-125b-2-3p | MIMAT0004603 | ESRRG | NM_001243509 | 0 | 1 | 0 | 1 | 1 | 3 |
| hsa-miR-125b-2-3p | MIMAT0004603 | ACSL3 | NM_004457 | 1 | 1 | 0 | 0 | 1 | 3 |
| hsa-miR-125b-2-3p | MIMAT0004603 | FGF7 | NM_002009 | 1 | 1 | 0 | 0 | 1 | 3 |
| hsa-miR-125b-2-3p | MIMAT0004603 | FIGF | NM_004469 | 1 | 0 | 0 | 1 | 1 | 3 |
| hsa-miR-125b-2-3p | MIMAT0004603 | FLI1 | NM_001271010 | 1 | 0 | 0 | 1 | 1 | 3 |
| hsa-miR-125b-2-3p | MIMAT0004603 | FMO2 | NM_001460 | 1 | 0 | 0 | 1 | 1 | 3 |
| hsa-miR-125b-2-3p | MIMAT0004603 | FUT1 | NM_000148 | 1 | 0 | 0 | 1 | 1 | 3 |
| hsa-miR-125b-2-3p | MIMAT0004603 | GABPA | NM_002040 | 1 | 0 | 0 | 1 | 1 | 3 |
| hsa-miR-125b-2-3p | MIMAT0004603 | GABRB2 | NM_021911 | 1 | 0 | 0 | 1 | 1 | 3 |
| hsa-miR-125b-2-3p | MIMAT0004603 | GABRE | XM_005274661 | 1 | 0 | 0 | 1 | 1 | 3 |
| hsa-miR-125b-2-3p | MIMAT0004603 | GAD2 | NM_000818 | 0 | 1 | 1 | 0 | 1 | 3 |
| hsa-miR-125b-2-3p | MIMAT0004603 | GCKR | XM_005264257 | 0 | 1 | 0 | 1 | 1 | 3 |
| hsa-miR-125b-2-3p | MIMAT0004603 | GFRA1 | NM_005264 | 1 | 0 | 0 | 1 | 1 | 3 |
| hsa-miR-125b-2-3p | MIMAT0004603 | GJA1 | NM_000165 | 0 | 1 | 0 | 1 | 1 | 3 |
| hsa-miR-125b-2-3p | MIMAT0004603 | GJA5 | XM_005277385 | 0 | 1 | 0 | 1 | 1 | 3 |
| hsa-miR-125b-2-3p | MIMAT0004603 | GLE1 | XM_005251925 | 1 | 0 | 0 | 1 | 1 | 3 |
| hsa-miR-125b-2-3p | MIMAT0004603 | GLI3 | XM_005249704 | 1 | 0 | 0 | 1 | 1 | 3 |
| hsa-miR-125b-2-3p | MIMAT0004603 | GLS | NM_014905 | 1 | 0 | 0 | 1 | 1 | 3 |
| hsa-miR-125b-2-3p | MIMAT0004603 | GNB1 | NM_002074 | 1 | 0 | 0 | 1 | 1 | 3 |
| hsa-miR-125b-2-3p | MIMAT0004603 | GOLGA4 | NM_002078 | 0 | 1 | 1 | 0 | 1 | 3 |
| hsa-miR-125b-2-3p | MIMAT0004603 | GPM6A | NM_201591 | 0 | 1 | 0 | 1 | 1 | 3 |
| hsa-miR-125b-2-3p | MIMAT0004603 | GPM6B | NM_001001995 | 0 | 1 | 1 | 0 | 1 | 3 |
| hsa-miR-125b-2-3p | MIMAT0004603 | FFAR2 | XM_005258817 | 1 | 0 | 0 | 1 | 1 | 3 |
| hsa-miR-125b-2-3p | MIMAT0004603 | GRIN2B | NM_000834 | 1 | 0 | 0 | 1 | 1 | 3 |
| hsa-miR-125b-2-3p | MIMAT0004603 | GRPR | NM_005314 | 1 | 1 | 0 | 0 | 1 | 3 |
| hsa-miR-125b-2-3p | MIMAT0004603 | GSK3B | NM_002093 | 1 | 1 | 0 | 0 | 1 | 3 |
| hsa-miR-125b-2-3p | MIMAT0004603 | H1F0 | NM_005318 | 0 | 1 | 0 | 1 | 1 | 3 |
| hsa-miR-125b-2-3p | MIMAT0004603 | HCRTR2 | NM_001526 | 0 | 1 | 1 | 0 | 1 | 3 |
| hsa-miR-125b-2-3p | MIMAT0004603 | HDC | NM_002112 | 1 | 0 | 0 | 1 | 1 | 3 |
| hsa-miR-125b-2-3p | MIMAT0004603 | HLCS | NM_001242784 | 0 | 1 | 0 | 1 | 1 | 3 |
| hsa-miR-125b-2-3p | MIMAT0004603 | HMGCR | XM_005248492 | 0 | 1 | 0 | 1 | 1 | 3 |
| hsa-miR-125b-2-3p | MIMAT0004603 | HNF4G | NM_004133 | 1 | 0 | 0 | 1 | 1 | 3 |
| hsa-miR-125b-2-3p | MIMAT0004603 | HNRNPH1 | NM_001257293 | 1 | 0 | 0 | 1 | 1 | 3 |
| hsa-miR-125b-2-3p | MIMAT0004603 | HNRNPH2 | NM_019597 | 1 | 0 | 0 | 1 | 1 | 3 |
| hsa-miR-125b-2-3p | MIMAT0004603 | AGFG1 | XM_005246516 | 1 | 1 | 0 | 0 | 1 | 3 |
| hsa-miR-125b-2-3p | MIMAT0004603 | FOXN2 | XM_005264283 | 0 | 1 | 0 | 1 | 1 | 3 |
| hsa-miR-125b-2-3p | MIMAT0004603 | ID2 | NM_002166 | 1 | 0 | 0 | 1 | 1 | 3 |
| hsa-miR-125b-2-3p | MIMAT0004603 | IDS | NM_000202 | 0 | 1 | 0 | 1 | 1 | 3 |
| hsa-miR-125b-2-3p | MIMAT0004603 | CXCR1 | NM_000634 | 1 | 0 | 0 | 1 | 1 | 3 |
| hsa-miR-125b-2-3p | MIMAT0004603 | IRAK2 | NM_001570 | 0 | 1 | 0 | 1 | 1 | 3 |
| hsa-miR-125b-2-3p | MIMAT0004603 | IRF2 | NM_002199 | 1 | 0 | 0 | 1 | 1 | 3 |
| hsa-miR-125b-2-3p | MIMAT0004603 | IRF4 | NM_002460 | 0 | 1 | 0 | 1 | 1 | 3 |
| hsa-miR-125b-2-3p | MIMAT0004603 | ITPR1 | NM_001168272 | 1 | 0 | 0 | 1 | 1 | 3 |
| hsa-miR-125b-2-3p | MIMAT0004603 | KCNC2 | NM_001260497 | 1 | 1 | 0 | 0 | 1 | 3 |
| hsa-miR-125b-2-3p | MIMAT0004603 | KCND3 | NM_004980 | 0 | 1 | 0 | 1 | 1 | 3 |
| hsa-miR-125b-2-3p | MIMAT0004603 | KDR | NM_002253 | 1 | 0 | 0 | 1 | 1 | 3 |
| hsa-miR-125b-2-3p | MIMAT0004603 | KLC1 | XM_005267601 | 1 | 1 | 0 | 0 | 1 | 3 |
| hsa-miR-125b-2-3p | MIMAT0004603 | KRAS | NM_033360 | 1 | 0 | 0 | 1 | 1 | 3 |
| hsa-miR-125b-2-3p | MIMAT0004603 | LIFR | NM_001127671 | 1 | 0 | 0 | 1 | 1 | 3 |
| hsa-miR-125b-2-3p | MIMAT0004603 | LRP6 | NM_002336 | 1 | 0 | 0 | 1 | 1 | 3 |
| hsa-miR-125b-2-3p | MIMAT0004603 | SMAD4 | NM_005359 | 0 | 1 | 0 | 1 | 1 | 3 |
| hsa-miR-125b-2-3p | MIMAT0004603 | SMAD5 | NM_001001419 | 1 | 0 | 0 | 1 | 1 | 3 |
| hsa-miR-125b-2-3p | MIMAT0004603 | SMAD9 | NM_001127217 | 1 | 0 | 0 | 1 | 1 | 3 |
| hsa-miR-125b-2-3p | MIMAT0004603 | MAFG | NM_032711 | 1 | 0 | 0 | 1 | 1 | 3 |
| hsa-miR-125b-2-3p | MIMAT0004603 | MAGEB4 | NM_002367 | 1 | 0 | 0 | 1 | 1 | 3 |
| hsa-miR-125b-2-3p | MIMAT0004603 | MARK1 | NM_018650 | 1 | 1 | 0 | 0 | 1 | 3 |
| hsa-miR-125b-2-3p | MIMAT0004603 | MAT2A | NM_005911 | 1 | 0 | 0 | 1 | 1 | 3 |
| hsa-miR-125b-2-3p | MIMAT0004603 | MATN2 | NM_002380 | 0 | 1 | 0 | 1 | 1 | 3 |
| hsa-miR-125b-2-3p | MIMAT0004603 | MCC | NM_001085377 | 0 | 1 | 0 | 1 | 1 | 3 |
| hsa-miR-125b-2-3p | MIMAT0004603 | MGAT5 | XM_005263666 | 1 | 0 | 0 | 1 | 1 | 3 |
| hsa-miR-125b-2-3p | MIMAT0004603 | MITF | NM_198159 | 1 | 1 | 0 | 0 | 1 | 3 |
| hsa-miR-125b-2-3p | MIMAT0004603 | MKI67 | NM_002417 | 1 | 0 | 0 | 1 | 1 | 3 |
| hsa-miR-125b-2-3p | MIMAT0004603 | AFF1 | XM_005263009 | 1 | 0 | 0 | 1 | 1 | 3 |
| hsa-miR-125b-2-3p | MIMAT0004603 | MPI | NM_002435 | 1 | 1 | 0 | 0 | 1 | 3 |
| hsa-miR-125b-2-3p | MIMAT0004603 | MTRR | XM_005248305 | 1 | 0 | 0 | 1 | 1 | 3 |
| hsa-miR-125b-2-3p | MIMAT0004603 | MYO6 | NM_004999 | 1 | 0 | 0 | 1 | 1 | 3 |
| hsa-miR-125b-2-3p | MIMAT0004603 | MYO10 | NM_012334 | 1 | 1 | 0 | 0 | 1 | 3 |
| hsa-miR-125b-2-3p | MIMAT0004603 | PPP1R12B | XM_005245201 | 1 | 0 | 0 | 1 | 1 | 3 |
| hsa-miR-125b-2-3p | MIMAT0004603 | NHP2L1 | XM_005261620 | 1 | 0 | 0 | 1 | 1 | 3 |
| hsa-miR-125b-2-3p | MIMAT0004603 | NHS | XM_005274539 | 1 | 0 | 0 | 1 | 1 | 3 |
| hsa-miR-125b-2-3p | MIMAT0004603 | NRAS | NM_002524 | 1 | 0 | 0 | 1 | 1 | 3 |
| hsa-miR-125b-2-3p | MIMAT0004603 | ROR1 | NM_005012 | 1 | 0 | 0 | 1 | 1 | 3 |
| hsa-miR-125b-2-3p | MIMAT0004603 | TBC1D25 | NM_002536 | 1 | 0 | 0 | 1 | 1 | 3 |
| hsa-miR-125b-2-3p | MIMAT0004603 | OAZ2 | NM_002537 | 1 | 1 | 0 | 0 | 1 | 3 |
| hsa-miR-125b-2-3p | MIMAT0004603 | OPA1 | NM_130837 | 1 | 1 | 0 | 0 | 1 | 3 |
| hsa-miR-125b-2-3p | MIMAT0004603 | PAFAH1B2 | NM_002572 | 1 | 0 | 0 | 1 | 1 | 3 |
| hsa-miR-125b-2-3p | MIMAT0004603 | PAFAH2 | XM_005245874 | 1 | 1 | 0 | 0 | 1 | 3 |
| hsa-miR-125b-2-3p | MIMAT0004603 | PAK2 | NM_002577 | 1 | 0 | 0 | 1 | 1 | 3 |
| hsa-miR-125b-2-3p | MIMAT0004603 | PAX5 | NM_016734 | 1 | 1 | 0 | 0 | 1 | 3 |
| hsa-miR-125b-2-3p | MIMAT0004603 | PDE7A | NM_001242318 | 1 | 1 | 0 | 0 | 1 | 3 |
| hsa-miR-125b-2-3p | MIMAT0004603 | PFKFB4 | XM_005265231 | 1 | 0 | 0 | 1 | 1 | 3 |
| hsa-miR-125b-2-3p | MIMAT0004603 | PGM3 | NM_001199917 | 1 | 0 | 0 | 1 | 1 | 3 |
| hsa-miR-125b-2-3p | MIMAT0004603 | PHF2 | NM_005392 | 1 | 0 | 0 | 1 | 1 | 3 |
| hsa-miR-125b-2-3p | MIMAT0004603 | PHKA2 | NM_000292 | 1 | 1 | 0 | 0 | 1 | 3 |
| hsa-miR-125b-2-3p | MIMAT0004603 | PIK3C2B | XM_005245260 | 1 | 0 | 0 | 1 | 1 | 3 |
| hsa-miR-125b-2-3p | MIMAT0004603 | PITPNA | NM_006224 | 1 | 0 | 0 | 1 | 1 | 3 |
| hsa-miR-125b-2-3p | MIMAT0004603 | PLAU | NM_001145031 | 1 | 1 | 0 | 0 | 1 | 3 |
| hsa-miR-125b-2-3p | MIMAT0004603 | PLD1 | NM_002662 | 1 | 0 | 0 | 1 | 1 | 3 |
| hsa-miR-125b-2-3p | MIMAT0004603 | PPP3CA | NM_000944 | 1 | 0 | 0 | 1 | 1 | 3 |
| hsa-miR-125b-2-3p | MIMAT0004603 | PKIA | NM_006823 | 1 | 0 | 0 | 1 | 1 | 3 |
| hsa-miR-125b-2-3p | MIMAT0004603 | PRKCE | XM_005264429 | 1 | 1 | 0 | 0 | 1 | 3 |
| hsa-miR-125b-2-3p | MIMAT0004603 | MAPK6 | XM_005254536 | 1 | 1 | 0 | 0 | 1 | 3 |
| hsa-miR-125b-2-3p | MIMAT0004603 | PRLR | NM_000949 | 1 | 0 | 0 | 1 | 1 | 3 |
| hsa-miR-125b-2-3p | MIMAT0004603 | PSAP | NM_001042465 | 1 | 1 | 0 | 0 | 1 | 3 |
| hsa-miR-125b-2-3p | MIMAT0004603 | PTBP1 | XM_005259597 | 1 | 0 | 0 | 1 | 1 | 3 |
| hsa-miR-125b-2-3p | MIMAT0004603 | PTEN | NM_000314 | 1 | 1 | 0 | 0 | 1 | 3 |
| hsa-miR-125b-2-3p | MIMAT0004603 | PTPRB | NM_001109754 | 1 | 0 | 0 | 1 | 1 | 3 |
| hsa-miR-125b-2-3p | MIMAT0004603 | PTPRK | XM_005267081 | 1 | 1 | 0 | 0 | 1 | 3 |
| hsa-miR-125b-2-3p | MIMAT0004603 | PTPRR | NM_002849 | 1 | 0 | 0 | 1 | 1 | 3 |
| hsa-miR-125b-2-3p | MIMAT0004603 | PVRL1 | NM_002855 | 1 | 0 | 0 | 1 | 1 | 3 |
| hsa-miR-125b-2-3p | MIMAT0004603 | RAB3B | NM_002867 | 1 | 0 | 0 | 1 | 1 | 3 |
| hsa-miR-125b-2-3p | MIMAT0004603 | RAB4A | NM_004578 | 1 | 1 | 0 | 0 | 1 | 3 |
| hsa-miR-125b-2-3p | MIMAT0004603 | RAP1GDS1 | NM_001100426 | 1 | 1 | 0 | 0 | 1 | 3 |
| hsa-miR-125b-2-3p | MIMAT0004603 | RASGRF2 | NM_006909 | 1 | 1 | 0 | 0 | 1 | 3 |
| hsa-miR-125b-2-3p | MIMAT0004603 | RBBP4 | NM_005610 | 1 | 1 | 0 | 0 | 1 | 3 |
| hsa-miR-125b-2-3p | MIMAT0004603 | RHD | NM_016124 | 1 | 0 | 0 | 1 | 1 | 3 |
| hsa-miR-125b-2-3p | MIMAT0004603 | RIT1 | NM_006912 | 1 | 1 | 0 | 0 | 1 | 3 |
| hsa-miR-125b-2-3p | MIMAT0004603 | RPL32 | NM_001007074 | 1 | 0 | 0 | 1 | 1 | 3 |
| hsa-miR-125b-2-3p | MIMAT0004603 | RPS6KB1 | NM_001272044 | 1 | 0 | 0 | 1 | 1 | 3 |
| hsa-miR-125b-2-3p | MIMAT0004603 | RREB1 | XM_005249274 | 1 | 0 | 0 | 1 | 1 | 3 |
| hsa-miR-125b-2-3p | MIMAT0004603 | SBF1 | NM_002972 | 1 | 1 | 0 | 0 | 1 | 3 |
| hsa-miR-125b-2-3p | MIMAT0004603 | SCN4A | XM_005257566 | 1 | 1 | 0 | 0 | 1 | 3 |
| hsa-miR-125b-2-3p | MIMAT0004603 | SDHA | NM_004168 | 1 | 0 | 0 | 1 | 1 | 3 |
| hsa-miR-125b-2-3p | MIMAT0004603 | SLC5A3 | NM_006933 | 1 | 0 | 0 | 1 | 1 | 3 |
| hsa-miR-125b-2-3p | MIMAT0004603 | SMARCD1 | XM_005269107 | 1 | 0 | 0 | 1 | 1 | 3 |
| hsa-miR-125b-2-3p | MIMAT0004603 | SMARCE1 | XM_005257607 | 1 | 1 | 0 | 0 | 1 | 3 |
| hsa-miR-125b-2-3p | MIMAT0004603 | SNRPB2 | NM_003092 | 1 | 1 | 0 | 0 | 1 | 3 |
| hsa-miR-125b-2-3p | MIMAT0004603 | SNTB1 | XM_005251031 | 1 | 0 | 0 | 1 | 1 | 3 |
| hsa-miR-125b-2-3p | MIMAT0004603 | SNX1 | NM_003099 | 1 | 0 | 0 | 1 | 1 | 3 |
| hsa-miR-125b-2-3p | MIMAT0004603 | SOX5 | NM_152989 | 1 | 0 | 0 | 1 | 1 | 3 |
| hsa-miR-125b-2-3p | MIMAT0004603 | SP2 | XM_005257611 | 1 | 1 | 0 | 0 | 1 | 3 |
| hsa-miR-125b-2-3p | MIMAT0004603 | SPIB | NM_003121 | 1 | 1 | 0 | 0 | 1 | 3 |
| hsa-miR-125b-2-3p | MIMAT0004603 | STAU1 | XM_005260524 | 1 | 0 | 0 | 1 | 1 | 3 |
| hsa-miR-125b-2-3p | MIMAT0004603 | STC1 | NM_003155 | 1 | 0 | 0 | 1 | 1 | 3 |
| hsa-miR-125b-2-3p | MIMAT0004603 | STK4 | XM_005260533 | 1 | 0 | 0 | 1 | 1 | 3 |
| hsa-miR-125b-2-3p | MIMAT0004603 | STRN | NM_003162 | 1 | 1 | 0 | 0 | 1 | 3 |
| hsa-miR-125b-2-3p | MIMAT0004603 | STX3 | NM_004177 | 1 | 0 | 0 | 1 | 1 | 3 |
| hsa-miR-125b-2-3p | MIMAT0004603 | VAMP2 | XM_005256775 | 1 | 0 | 0 | 1 | 1 | 3 |
| hsa-miR-125b-2-3p | MIMAT0004603 | TACC1 | XM_005273622 | 1 | 0 | 0 | 1 | 1 | 3 |
| hsa-miR-125b-2-3p | MIMAT0004603 | TCF4 | NM_001083962 | 1 | 1 | 0 | 0 | 1 | 3 |
| hsa-miR-125b-2-3p | MIMAT0004603 | TBX3 | NM_016569 | 1 | 0 | 0 | 1 | 1 | 3 |
| hsa-miR-125b-2-3p | MIMAT0004603 | TECTB | XM_005270107 | 1 | 1 | 0 | 0 | 1 | 3 |
| hsa-miR-125b-2-3p | MIMAT0004603 | TEAD1 | NM_021961 | 1 | 0 | 0 | 1 | 1 | 3 |
| hsa-miR-125b-2-3p | MIMAT0004603 | TERF1 | XM_005251291 | 1 | 1 | 0 | 0 | 1 | 3 |
| hsa-miR-125b-2-3p | MIMAT0004603 | TFPI | XM_005246818 | 1 | 0 | 0 | 1 | 1 | 3 |
| hsa-miR-125b-2-3p | MIMAT0004603 | TGFB2 | NM_001135599 | 1 | 1 | 0 | 0 | 1 | 3 |
| hsa-miR-125b-2-3p | MIMAT0004603 | TGFBR1 | NM_004612 | 1 | 0 | 0 | 1 | 1 | 3 |
| hsa-miR-125b-2-3p | MIMAT0004603 | TMF1 | NM_007114 | 1 | 0 | 0 | 1 | 1 | 3 |
| hsa-miR-125b-2-3p | MIMAT0004603 | TRAF6 | NM_145803 | 1 | 1 | 0 | 0 | 1 | 3 |
| hsa-miR-125b-2-3p | MIMAT0004603 | TSN | NM_004622 | 1 | 0 | 0 | 1 | 1 | 3 |
| hsa-miR-125b-2-3p | MIMAT0004603 | SLC35A2 | NM_001042498 | 1 | 0 | 0 | 1 | 1 | 3 |
| hsa-miR-125b-2-3p | MIMAT0004603 | UGCG | XM_005252186 | 1 | 1 | 0 | 0 | 1 | 3 |
| hsa-miR-125b-2-3p | MIMAT0004603 | VEGFC | NM_005429 | 1 | 0 | 0 | 1 | 1 | 3 |
| hsa-miR-125b-2-3p | MIMAT0004603 | EIF4H | NM_022170 | 1 | 0 | 0 | 1 | 1 | 3 |
| hsa-miR-125b-2-3p | MIMAT0004603 | ZIC2 | NM_007129 | 1 | 0 | 0 | 1 | 1 | 3 |
| hsa-miR-125b-2-3p | MIMAT0004603 | TRIM26 | NM_003449 | 1 | 0 | 0 | 1 | 1 | 3 |
| hsa-miR-125b-2-3p | MIMAT0004603 | ZMYM2 | NM_003453 | 1 | 0 | 0 | 1 | 1 | 3 |
| hsa-miR-125b-2-3p | MIMAT0004603 | ZNF226 | XM_005259227 | 1 | 0 | 0 | 1 | 1 | 3 |
| hsa-miR-125b-2-3p | MIMAT0004603 | SLC30A3 | XM_005264551 | 1 | 1 | 0 | 0 | 1 | 3 |
| hsa-miR-125b-2-3p | MIMAT0004603 | SLMAP | XM_005265458 | 1 | 0 | 0 | 1 | 1 | 3 |
| hsa-miR-125b-2-3p | MIMAT0004603 | HSD17B8 | NM_014234 | 1 | 0 | 0 | 1 | 1 | 3 |
| hsa-miR-125b-2-3p | MIMAT0004603 | CCDC6 | NM_005436 | 1 | 1 | 0 | 0 | 1 | 3 |
| hsa-miR-125b-2-3p | MIMAT0004603 | CUL5 | NM_003478 | 0 | 1 | 0 | 1 | 1 | 3 |
| hsa-miR-125b-2-3p | MIMAT0004603 | PTP4A2 | NM_080391 | 1 | 0 | 0 | 1 | 1 | 3 |
| hsa-miR-125b-2-3p | MIMAT0004603 | CUL4A | NM_001278513 | 1 | 0 | 0 | 1 | 1 | 3 |
| hsa-miR-125b-2-3p | MIMAT0004603 | PPFIBP1 | NM_177444 | 1 | 0 | 0 | 1 | 1 | 3 |
| hsa-miR-125b-2-3p | MIMAT0004603 | CGGBP1 | NM_001008390 | 1 | 0 | 0 | 1 | 1 | 3 |
| hsa-miR-125b-2-3p | MIMAT0004603 | DENR | NM_003677 | 1 | 1 | 0 | 0 | 1 | 3 |
| hsa-miR-125b-2-3p | MIMAT0004603 | NCOA1 | XM_005264625 | 1 | 0 | 0 | 1 | 1 | 3 |
| hsa-miR-125b-2-3p | MIMAT0004603 | EIF3A | XM_005270259 | 0 | 1 | 0 | 1 | 1 | 3 |
| hsa-miR-125b-2-3p | MIMAT0004603 | PEA15 | XM_005245562 | 1 | 0 | 0 | 1 | 1 | 3 |
| hsa-miR-125b-2-3p | MIMAT0004603 | MBTPS1 | NM_003791 | 1 | 1 | 0 | 0 | 1 | 3 |
| hsa-miR-125b-2-3p | MIMAT0004603 | SNX3 | NM_003795 | 0 | 1 | 0 | 1 | 1 | 3 |
| hsa-miR-125b-2-3p | MIMAT0004603 | ADAM19 | NM_033274 | 1 | 0 | 0 | 1 | 1 | 3 |
| hsa-miR-125b-2-3p | MIMAT0004603 | TNFRSF11A | NM_003839 | 1 | 0 | 0 | 1 | 1 | 3 |
| hsa-miR-125b-2-3p | MIMAT0004603 | CD84 | NM_001184879 | 1 | 0 | 0 | 1 | 1 | 3 |
| hsa-miR-125b-2-3p | MIMAT0004603 | HCAR3 | NM_006018 | 1 | 1 | 0 | 0 | 1 | 3 |
| hsa-miR-125b-2-3p | MIMAT0004603 | PER2 | NM_022817 | 1 | 0 | 0 | 1 | 1 | 3 |
| hsa-miR-125b-2-3p | MIMAT0004603 | SYNJ1 | NM_203446 | 1 | 0 | 0 | 1 | 1 | 3 |
| hsa-miR-125b-2-3p | MIMAT0004603 | PHOX2B | NM_003924 | 1 | 0 | 0 | 1 | 1 | 3 |
| hsa-miR-125b-2-3p | MIMAT0004603 | SEMA5A | NM_003966 | 1 | 0 | 0 | 1 | 1 | 3 |
| hsa-miR-125b-2-3p | MIMAT0004603 | SPAG9 | NM_001130528 | 1 | 0 | 0 | 1 | 1 | 3 |
| hsa-miR-125b-2-3p | MIMAT0004603 | TBX19 | NM_005149 | 0 | 1 | 0 | 1 | 1 | 3 |
| hsa-miR-125b-2-3p | MIMAT0004603 | TBX18 | NM_001080508 | 1 | 0 | 0 | 1 | 1 | 3 |
| hsa-miR-125b-2-3p | MIMAT0004603 | MTMR6 | NM_004685 | 1 | 0 | 0 | 1 | 1 | 3 |
| hsa-miR-125b-2-3p | MIMAT0004603 | MTMR4 | XM_005257784 | 1 | 0 | 0 | 1 | 1 | 3 |
| hsa-miR-125b-2-3p | MIMAT0004603 | CBFA2T2 | NM_005093 | 1 | 0 | 0 | 1 | 1 | 3 |
| hsa-miR-125b-2-3p | MIMAT0004603 | NEURL | NM_004210 | 1 | 0 | 0 | 1 | 1 | 3 |
| hsa-miR-125b-2-3p | MIMAT0004603 | DGKI | NM_004717 | 1 | 1 | 0 | 0 | 1 | 3 |
| hsa-miR-125b-2-3p | MIMAT0004603 | XPR1 | NM_004736 | 1 | 0 | 0 | 1 | 1 | 3 |
| hsa-miR-125b-2-3p | MIMAT0004603 | DLGAP1 | XM_005258171 | 1 | 0 | 0 | 1 | 1 | 3 |
| hsa-miR-125b-2-3p | MIMAT0004603 | TBRG4 | NM_004749 | 1 | 0 | 0 | 1 | 1 | 3 |
| hsa-miR-125b-2-3p | MIMAT0004603 | STK17B | NM_004226 | 0 | 1 | 0 | 1 | 1 | 3 |
| hsa-miR-125b-2-3p | MIMAT0004603 | SOCS6 | NM_004232 | 1 | 0 | 0 | 1 | 1 | 3 |
| hsa-miR-125b-2-3p | MIMAT0004603 | NRXN3 | XM_005268218 | 1 | 0 | 0 | 1 | 1 | 3 |
| hsa-miR-125b-2-3p | MIMAT0004603 | NRXN1 | NM_001135659 | 1 | 1 | 0 | 0 | 1 | 3 |
| hsa-miR-125b-2-3p | MIMAT0004603 | ZRANB2 | NM_005455 | 1 | 0 | 0 | 1 | 1 | 3 |
| hsa-miR-125b-2-3p | MIMAT0004603 | FADS2 | NM_004265 | 1 | 0 | 0 | 1 | 1 | 3 |
| hsa-miR-125b-2-3p | MIMAT0004603 | MED26 | NM_004831 | 1 | 0 | 0 | 1 | 1 | 3 |
| hsa-miR-125b-2-3p | MIMAT0004603 | ITM2B | NM_021999 | 0 | 1 | 1 | 0 | 1 | 3 |
| hsa-miR-125b-2-3p | MIMAT0004603 | ATG5 | NM_004849 | 1 | 0 | 0 | 1 | 1 | 3 |
| hsa-miR-125b-2-3p | MIMAT0004603 | ONECUT2 | NM_004852 | 1 | 0 | 0 | 1 | 1 | 3 |
| hsa-miR-125b-2-3p | MIMAT0004603 | IGDCC3 | NM_004884 | 1 | 0 | 0 | 1 | 1 | 3 |
| hsa-miR-125b-2-3p | MIMAT0004603 | CXCL14 | NM_004887 | 0 | 1 | 0 | 1 | 1 | 3 |
| hsa-miR-125b-2-3p | MIMAT0004603 | CDC42BPB | NM_006035 | 1 | 0 | 0 | 1 | 1 | 3 |
| hsa-miR-125b-2-3p | MIMAT0004603 | NFE2L3 | NM_004289 | 0 | 1 | 0 | 1 | 1 | 3 |
| hsa-miR-125b-2-3p | MIMAT0004603 | RNF14 | NM_004290 | 1 | 0 | 0 | 1 | 1 | 3 |
| hsa-miR-125b-2-3p | MIMAT0004603 | RALGPS1 | XM_005252318 | 1 | 0 | 0 | 1 | 1 | 3 |
| hsa-miR-125b-2-3p | MIMAT0004603 | HS2ST1 | NM_012262 | 1 | 0 | 0 | 1 | 1 | 3 |
| hsa-miR-125b-2-3p | MIMAT0004603 | DZIP3 | NM_014648 | 1 | 0 | 0 | 1 | 1 | 3 |
| hsa-miR-125b-2-3p | MIMAT0004603 | PHF14 | NM_014660 | 1 | 1 | 0 | 0 | 1 | 3 |
| hsa-miR-125b-2-3p | MIMAT0004603 | RAPGEF2 | XM_005263358 | 1 | 0 | 0 | 1 | 1 | 3 |
| hsa-miR-125b-2-3p | MIMAT0004603 | TRAM2 | NM_012288 | 1 | 0 | 0 | 1 | 1 | 3 |
| hsa-miR-125b-2-3p | MIMAT0004603 | FAM131B | XM_005250073 | 1 | 0 | 0 | 1 | 1 | 3 |
| hsa-miR-125b-2-3p | MIMAT0004603 | MLEC | NM_014730 | 1 | 0 | 0 | 1 | 1 | 3 |
| hsa-miR-125b-2-3p | MIMAT0004603 | TM9SF4 | XM_005260622 | 1 | 0 | 0 | 1 | 1 | 3 |
| hsa-miR-125b-2-3p | MIMAT0004603 | KIAA0232 | NM_014743 | 1 | 0 | 0 | 1 | 1 | 3 |
| hsa-miR-125b-2-3p | MIMAT0004603 | TOMM20 | NM_014765 | 1 | 0 | 0 | 1 | 1 | 3 |
| hsa-miR-125b-2-3p | MIMAT0004603 | SPATA2 | NM_001135773 | 1 | 0 | 0 | 1 | 1 | 3 |
| hsa-miR-125b-2-3p | MIMAT0004603 | ZEB2 | NM_014795 | 1 | 1 | 0 | 0 | 1 | 3 |
| hsa-miR-125b-2-3p | MIMAT0004603 | GAB2 | NM_080491 | 0 | 1 | 0 | 1 | 1 | 3 |
| hsa-miR-125b-2-3p | MIMAT0004603 | CEP350 | XM_005245635 | 1 | 0 | 0 | 1 | 1 | 3 |
| hsa-miR-125b-2-3p | MIMAT0004603 | MAGI2 | XM_005250725 | 0 | 1 | 0 | 1 | 1 | 3 |
| hsa-miR-125b-2-3p | MIMAT0004603 | TOX4 | NM_014828 | 0 | 1 | 0 | 1 | 1 | 3 |
| hsa-miR-125b-2-3p | MIMAT0004603 | FAM20B | NM_014864 | 1 | 0 | 0 | 1 | 1 | 3 |
| hsa-miR-125b-2-3p | MIMAT0004603 | NAALAD2 | XM_005273701 | 1 | 0 | 0 | 1 | 1 | 3 |
| hsa-miR-125b-2-3p | MIMAT0004603 | TNK2 | NM_005781 | 1 | 1 | 0 | 0 | 1 | 3 |
| hsa-miR-125b-2-3p | MIMAT0004603 | STX6 | NM_005819 | 0 | 1 | 0 | 1 | 1 | 3 |
| hsa-miR-125b-2-3p | MIMAT0004603 | GPHN | NM_020806 | 0 | 1 | 0 | 1 | 1 | 3 |
| hsa-miR-125b-2-3p | MIMAT0004603 | STAM2 | NM_005843 | 1 | 0 | 0 | 1 | 1 | 3 |
| hsa-miR-125b-2-3p | MIMAT0004603 | UBE4B | NM_001105562 | 1 | 0 | 0 | 1 | 1 | 3 |
| hsa-miR-125b-2-3p | MIMAT0004603 | SF3A1 | NM_005877 | 1 | 0 | 0 | 1 | 1 | 3 |
| hsa-miR-125b-2-3p | MIMAT0004603 | DNAJA2 | NM_005880 | 1 | 0 | 0 | 1 | 1 | 3 |
| hsa-miR-125b-2-3p | MIMAT0004603 | 6-Mar | NM_005885 | 1 | 1 | 0 | 0 | 1 | 3 |
| hsa-miR-125b-2-3p | MIMAT0004603 | B3GNT3 | NM_014256 | 0 | 1 | 0 | 1 | 1 | 3 |
| hsa-miR-125b-2-3p | MIMAT0004603 | KLF2 | NM_016270 | 1 | 0 | 0 | 1 | 1 | 3 |
| hsa-miR-125b-2-3p | MIMAT0004603 | CEPT1 | NM_001007794 | 1 | 0 | 0 | 1 | 1 | 3 |
| hsa-miR-125b-2-3p | MIMAT0004603 | ATP8A1 | NM_006095 | 0 | 1 | 0 | 1 | 1 | 3 |
| hsa-miR-125b-2-3p | MIMAT0004603 | CRTAP | NM_006371 | 1 | 1 | 0 | 0 | 1 | 3 |
| hsa-miR-125b-2-3p | MIMAT0004603 | SEMA3C | NM_006379 | 0 | 1 | 0 | 1 | 1 | 3 |
| hsa-miR-125b-2-3p | MIMAT0004603 | SLU7 | NM_006425 | 1 | 0 | 0 | 1 | 1 | 3 |
| hsa-miR-125b-2-3p | MIMAT0004603 | SORBS1 | XM_005269403 | 0 | 1 | 0 | 1 | 1 | 3 |
| hsa-miR-125b-2-3p | MIMAT0004603 | IVNS1ABP | XM_005244843 | 1 | 1 | 0 | 0 | 1 | 3 |
| hsa-miR-125b-2-3p | MIMAT0004603 | CAMKK2 | NM_006549 | 0 | 1 | 0 | 1 | 1 | 3 |
| hsa-miR-125b-2-3p | MIMAT0004603 | CELF1 | XM_005252758 | 0 | 1 | 0 | 1 | 1 | 3 |
| hsa-miR-125b-2-3p | MIMAT0004603 | CELF2 | NM_001025077 | 1 | 0 | 0 | 1 | 1 | 3 |
| hsa-miR-125b-2-3p | MIMAT0004603 | MGEA5 | XM_005269453 | 1 | 0 | 0 | 1 | 1 | 3 |
| hsa-miR-125b-2-3p | MIMAT0004603 | AHCYL1 | NM_001242673 | 1 | 0 | 0 | 1 | 1 | 3 |
| hsa-miR-125b-2-3p | MIMAT0004603 | PLK2 | NM_006622 | 0 | 1 | 1 | 0 | 1 | 3 |
| hsa-miR-125b-2-3p | MIMAT0004603 | FRS2 | NM_001278351 | 1 | 1 | 0 | 0 | 1 | 3 |
| hsa-miR-125b-2-3p | MIMAT0004603 | PGRMC1 | NM_006667 | 1 | 0 | 0 | 1 | 1 | 3 |
| hsa-miR-125b-2-3p | MIMAT0004603 | YWHAQ | NM_006826 | 1 | 0 | 0 | 1 | 1 | 3 |
| hsa-miR-125b-2-3p | MIMAT0004603 | METAP2 | NM_006838 | 1 | 1 | 0 | 0 | 1 | 3 |
| hsa-miR-125b-2-3p | MIMAT0004603 | DSTN | XM_005260653 | 1 | 1 | 0 | 0 | 1 | 3 |
| hsa-miR-125b-2-3p | MIMAT0004603 | CPSF6 | XM_005268588 | 1 | 0 | 0 | 1 | 1 | 3 |
| hsa-miR-125b-2-3p | MIMAT0004603 | ABHD2 | NM_007011 | 1 | 1 | 0 | 0 | 1 | 3 |
| hsa-miR-125b-2-3p | MIMAT0004603 | C14orf1 | NM_007176 | 1 | 1 | 0 | 0 | 1 | 3 |
| hsa-miR-125b-2-3p | MIMAT0004603 | PSIP1 | NM_033222 | 0 | 1 | 0 | 1 | 1 | 3 |
| hsa-miR-125b-2-3p | MIMAT0004603 | AKAP10 | NM_007202 | 1 | 0 | 0 | 1 | 1 | 3 |
| hsa-miR-125b-2-3p | MIMAT0004603 | RNF24 | NM_007219 | 1 | 0 | 0 | 1 | 1 | 3 |
| hsa-miR-125b-2-3p | MIMAT0004603 | KLF12 | XM_005266251 | 0 | 1 | 0 | 1 | 1 | 3 |
| hsa-miR-125b-2-3p | MIMAT0004603 | MGAT4A | NM_012214 | 1 | 0 | 0 | 1 | 1 | 3 |
| hsa-miR-125b-2-3p | MIMAT0004603 | NLGN4Y | NM_014893 | 1 | 0 | 0 | 1 | 1 | 3 |
| hsa-miR-125b-2-3p | MIMAT0004603 | RNF44 | XM_005265842 | 0 | 1 | 0 | 1 | 1 | 3 |
| hsa-miR-125b-2-3p | MIMAT0004603 | PPM1E | NM_014906 | 1 | 1 | 0 | 0 | 1 | 3 |
| hsa-miR-125b-2-3p | MIMAT0004603 | ZNF507 | NM_001136156 | 1 | 0 | 0 | 1 | 1 | 3 |
| hsa-miR-125b-2-3p | MIMAT0004603 | INPP5F | NM_014937 | 1 | 0 | 0 | 1 | 1 | 3 |
| hsa-miR-125b-2-3p | MIMAT0004603 | DIS3 | NM_014953 | 0 | 1 | 0 | 1 | 1 | 3 |
| hsa-miR-125b-2-3p | MIMAT0004603 | BTBD3 | NM_014962 | 0 | 1 | 0 | 1 | 1 | 3 |
| hsa-miR-125b-2-3p | MIMAT0004603 | FAN1 | NM_014967 | 1 | 0 | 0 | 1 | 1 | 3 |
| hsa-miR-125b-2-3p | MIMAT0004603 | NT5C2 | XM_005269637 | 1 | 0 | 0 | 1 | 1 | 3 |
| hsa-miR-125b-2-3p | MIMAT0004603 | DIP2C | XM_005252428 | 1 | 0 | 0 | 1 | 1 | 3 |
| hsa-miR-125b-2-3p | MIMAT0004603 | RIMS1 | NM_014989 | 1 | 1 | 0 | 0 | 1 | 3 |
| hsa-miR-125b-2-3p | MIMAT0004603 | MYT1L | XM_005264685 | 1 | 0 | 0 | 1 | 1 | 3 |
| hsa-miR-125b-2-3p | MIMAT0004603 | MON2 | NM_001278472 | 0 | 1 | 0 | 1 | 1 | 3 |
| hsa-miR-125b-2-3p | MIMAT0004603 | TNIK | NM_015028 | 1 | 1 | 0 | 0 | 1 | 3 |
| hsa-miR-125b-2-3p | MIMAT0004603 | HECW1 | XM_005249663 | 0 | 1 | 0 | 1 | 1 | 3 |
| hsa-miR-125b-2-3p | MIMAT0004603 | ZC3H13 | XM_005266303 | 0 | 1 | 0 | 1 | 1 | 3 |
| hsa-miR-125b-2-3p | MIMAT0004603 | CDK19 | NM_015076 | 1 | 0 | 0 | 1 | 1 | 3 |
| hsa-miR-125b-2-3p | MIMAT0004603 | MRPS27 | NM_015084 | 1 | 1 | 0 | 0 | 1 | 3 |
| hsa-miR-125b-2-3p | MIMAT0004603 | CAMTA2 | XM_005256548 | 1 | 1 | 0 | 0 | 1 | 3 |
| hsa-miR-125b-2-3p | MIMAT0004603 | FAM175B | NM_032182 | 0 | 1 | 0 | 1 | 1 | 3 |
| hsa-miR-125b-2-3p | MIMAT0004603 | GANAB | NM_198335 | 1 | 0 | 0 | 1 | 1 | 3 |
| hsa-miR-125b-2-3p | MIMAT0004603 | ANKRD28 | NM_001195098 | 1 | 1 | 0 | 0 | 1 | 3 |
| hsa-miR-125b-2-3p | MIMAT0004603 | MGA | XM_005254243 | 0 | 1 | 0 | 1 | 1 | 3 |
| hsa-miR-125b-2-3p | MIMAT0004603 | TSPYL4 | NM_021648 | 1 | 0 | 0 | 1 | 1 | 3 |
| hsa-miR-125b-2-3p | MIMAT0004603 | KLHL18 | XM_005265001 | 1 | 0 | 0 | 1 | 1 | 3 |
| hsa-miR-125b-2-3p | MIMAT0004603 | WDR7 | NM_015285 | 1 | 1 | 0 | 0 | 1 | 3 |
| hsa-miR-125b-2-3p | MIMAT0004603 | U2SURP | NM_001080415 | 0 | 1 | 0 | 1 | 1 | 3 |
| hsa-miR-125b-2-3p | MIMAT0004603 | SUN1 | XM_005249680 | 1 | 0 | 0 | 1 | 1 | 3 |
| hsa-miR-125b-2-3p | MIMAT0004603 | HAUS5 | NM_015302 | 1 | 0 | 0 | 1 | 1 | 3 |
| hsa-miR-125b-2-3p | MIMAT0004603 | PSD3 | NM_015310 | 1 | 0 | 0 | 1 | 1 | 3 |
| hsa-miR-125b-2-3p | MIMAT0004603 | ARHGEF12 | NM_015313 | 0 | 1 | 0 | 1 | 1 | 3 |
| hsa-miR-125b-2-3p | MIMAT0004603 | KIAA0895 | NM_001199707 | 0 | 1 | 0 | 1 | 1 | 3 |
| hsa-miR-125b-2-3p | MIMAT0004603 | CRTC1 | NM_001098482 | 1 | 0 | 0 | 1 | 1 | 3 |
| hsa-miR-125b-2-3p | MIMAT0004603 | KIAA0947 | NM_015325 | 0 | 1 | 0 | 1 | 1 | 3 |
| hsa-miR-125b-2-3p | MIMAT0004603 | ZFPM2 | NM_012082 | 0 | 1 | 1 | 0 | 1 | 3 |
| hsa-miR-125b-2-3p | MIMAT0004603 | GRIP1 | XM_005268754 | 1 | 0 | 0 | 1 | 1 | 3 |
| hsa-miR-125b-2-3p | MIMAT0004603 | LRRC8B | NM_015350 | 1 | 0 | 0 | 1 | 1 | 3 |
| hsa-miR-125b-2-3p | MIMAT0004603 | KAT6B | NM_012330 | 1 | 0 | 0 | 1 | 1 | 3 |
| hsa-miR-125b-2-3p | MIMAT0004603 | ACAP2 | XM_005269314 | 0 | 1 | 0 | 1 | 1 | 3 |
| hsa-miR-125b-2-3p | MIMAT0004603 | PATZ1 | NM_014323 | 1 | 0 | 0 | 1 | 1 | 3 |
| hsa-miR-125b-2-3p | MIMAT0004603 | KPNA6 | NM_012316 | 1 | 0 | 0 | 1 | 1 | 3 |
| hsa-miR-125b-2-3p | MIMAT0004603 | GSPT2 | NM_018094 | 1 | 1 | 0 | 0 | 1 | 3 |
| hsa-miR-125b-2-3p | MIMAT0004603 | GABARAPL1 | NM_031412 | 0 | 1 | 0 | 1 | 1 | 3 |
| hsa-miR-125b-2-3p | MIMAT0004603 | ATXN10 | NM_013236 | 1 | 0 | 0 | 1 | 1 | 3 |
| hsa-miR-125b-2-3p | MIMAT0004603 | ARMC8 | NM_015396 | 0 | 1 | 0 | 1 | 1 | 3 |
| hsa-miR-125b-2-3p | MIMAT0004603 | C3orf17 | NM_015412 | 0 | 1 | 0 | 1 | 1 | 3 |
| hsa-miR-125b-2-3p | MIMAT0004603 | ATL3 | XM_005273890 | 1 | 1 | 0 | 0 | 1 | 3 |
| hsa-miR-125b-2-3p | MIMAT0004603 | WWTR1 | NM_015472 | 1 | 0 | 0 | 1 | 1 | 3 |
| hsa-miR-125b-2-3p | MIMAT0004603 | HIGD1A | NM_001099668 | 1 | 1 | 0 | 0 | 1 | 3 |
| hsa-miR-125b-2-3p | MIMAT0004603 | OSBPL3 | NM_015550 | 0 | 1 | 0 | 1 | 1 | 3 |
| hsa-miR-125b-2-3p | MIMAT0004603 | GLCE | XM_005254298 | 1 | 0 | 0 | 1 | 1 | 3 |
| hsa-miR-125b-2-3p | MIMAT0004603 | ZNF451 | NM_001257273 | 1 | 0 | 0 | 1 | 1 | 3 |
| hsa-miR-125b-2-3p | MIMAT0004603 | SIPA1L1 | XM_005267516 | 1 | 1 | 0 | 0 | 1 | 3 |
| hsa-miR-125b-2-3p | MIMAT0004603 | FAM169A | XM_005248479 | 1 | 1 | 0 | 0 | 1 | 3 |
| hsa-miR-125b-2-3p | MIMAT0004603 | ERC2 | XM_005265036 | 1 | 0 | 0 | 1 | 1 | 3 |
| hsa-miR-125b-2-3p | MIMAT0004603 | TANC2 | XM_005257201 | 1 | 1 | 0 | 0 | 1 | 3 |
| hsa-miR-125b-2-3p | MIMAT0004603 | GAPVD1 | XM_005251899 | 1 | 0 | 0 | 1 | 1 | 3 |
| hsa-miR-125b-2-3p | MIMAT0004603 | SERBP1 | NM_001018067 | 0 | 1 | 0 | 1 | 1 | 3 |
| hsa-miR-125b-2-3p | MIMAT0004603 | ZBTB20 | XM_005247336 | 1 | 1 | 0 | 0 | 1 | 3 |
| hsa-miR-125b-2-3p | MIMAT0004603 | ARL5A | NM_012097 | 0 | 1 | 0 | 1 | 1 | 3 |
| hsa-miR-125b-2-3p | MIMAT0004603 | KLHL3 | NM_001257194 | 1 | 0 | 0 | 1 | 1 | 3 |
| hsa-miR-125b-2-3p | MIMAT0004603 | TSPAN13 | NM_014399 | 0 | 1 | 0 | 1 | 1 | 3 |
| hsa-miR-125b-2-3p | MIMAT0004603 | FOXP1 | NM_001244808 | 1 | 0 | 0 | 1 | 1 | 3 |
| hsa-miR-125b-2-3p | MIMAT0004603 | PDE7B | XM_005266931 | 1 | 0 | 0 | 1 | 1 | 3 |
| hsa-miR-125b-2-3p | MIMAT0004603 | TNFRSF21 | NM_014452 | 1 | 0 | 0 | 1 | 1 | 3 |
| hsa-miR-125b-2-3p | MIMAT0004603 | PCDH17 | XM_005266358 | 1 | 0 | 0 | 1 | 1 | 3 |
| hsa-miR-125b-2-3p | MIMAT0004603 | RBMX | NM_002139 | 1 | 0 | 0 | 1 | 1 | 3 |
| hsa-miR-125b-2-3p | MIMAT0004603 | TMEM97 | NM_014573 | 1 | 0 | 0 | 1 | 1 | 3 |
| hsa-miR-125b-2-3p | MIMAT0004603 | CYP2S1 | NM_030622 | 1 | 0 | 0 | 1 | 1 | 3 |
| hsa-miR-125b-2-3p | MIMAT0004603 | CNOT7 | XM_005273481 | 1 | 0 | 0 | 1 | 1 | 3 |
| hsa-miR-125b-2-3p | MIMAT0004603 | TNPO2 | XM_005259880 | 0 | 1 | 0 | 1 | 1 | 3 |
| hsa-miR-125b-2-3p | MIMAT0004603 | DUOX2 | XM_005254421 | 1 | 0 | 0 | 1 | 1 | 3 |
| hsa-miR-125b-2-3p | MIMAT0004603 | ARHGEF4 | XM_005263681 | 0 | 1 | 0 | 1 | 1 | 3 |
| hsa-miR-125b-2-3p | MIMAT0004603 | RNF141 | NM_016422 | 1 | 0 | 0 | 1 | 1 | 3 |
| hsa-miR-125b-2-3p | MIMAT0004603 | CDON | XM_005271584 | 1 | 0 | 0 | 1 | 1 | 3 |
| hsa-miR-125b-2-3p | MIMAT0004603 | ISOC1 | NM_016048 | 1 | 0 | 0 | 1 | 1 | 3 |
| hsa-miR-125b-2-3p | MIMAT0004603 | IER3IP1 | NM_016097 | 1 | 0 | 0 | 1 | 1 | 3 |
| hsa-miR-125b-2-3p | MIMAT0004603 | HN1 | NM_001002033 | 1 | 0 | 0 | 1 | 1 | 3 |
| hsa-miR-125b-2-3p | MIMAT0004603 | CYB5R4 | NM_016230 | 0 | 1 | 1 | 0 | 1 | 3 |
| hsa-miR-125b-2-3p | MIMAT0004603 | RAPGEFL1 | NM_016339 | 1 | 0 | 0 | 1 | 1 | 3 |
| hsa-miR-125b-2-3p | MIMAT0004603 | TAOK3 | NM_016281 | 1 | 0 | 0 | 1 | 1 | 3 |
| hsa-miR-125b-2-3p | MIMAT0004603 | REV1 | NM_016316 | 1 | 0 | 0 | 1 | 1 | 3 |
| hsa-miR-125b-2-3p | MIMAT0004603 | DCDC2 | NM_016356 | 1 | 0 | 0 | 1 | 1 | 3 |
| hsa-miR-125b-2-3p | MIMAT0004603 | CHMP5 | NM_016410 | 1 | 0 | 0 | 1 | 1 | 3 |
| hsa-miR-125b-2-3p | MIMAT0004603 | KLF13 | NM_015995 | 1 | 0 | 0 | 1 | 1 | 3 |
| hsa-miR-125b-2-3p | MIMAT0004603 | CHMP3 | NM_016079 | 1 | 1 | 0 | 0 | 1 | 3 |
| hsa-miR-125b-2-3p | MIMAT0004603 | ASB4 | NM_016116 | 1 | 0 | 0 | 1 | 1 | 3 |
| hsa-miR-125b-2-3p | MIMAT0004603 | NAA38 | NM_016200 | 1 | 1 | 0 | 0 | 1 | 3 |
| hsa-miR-125b-2-3p | MIMAT0004603 | HECA | NM_016217 | 1 | 0 | 0 | 1 | 1 | 3 |
| hsa-miR-125b-2-3p | MIMAT0004603 | ACSL5 | NM_203380 | 1 | 0 | 0 | 1 | 1 | 3 |
| hsa-miR-125b-2-3p | MIMAT0004603 | EMCN | NM_016242 | 0 | 1 | 0 | 1 | 1 | 3 |
| hsa-miR-125b-2-3p | MIMAT0004603 | C9orf78 | NM_016520 | 1 | 0 | 0 | 1 | 1 | 3 |
| hsa-miR-125b-2-3p | MIMAT0004603 | CHIC1 | NM_001039840 | 1 | 1 | 0 | 0 | 1 | 3 |
| hsa-miR-125b-2-3p | MIMAT0004603 | NUP54 | NM_017426 | 0 | 1 | 0 | 1 | 1 | 3 |
| hsa-miR-125b-2-3p | MIMAT0004603 | ADAM22 | XM_005250444 | 1 | 0 | 0 | 1 | 1 | 3 |
| hsa-miR-125b-2-3p | MIMAT0004603 | RAB24 | NM_001031677 | 0 | 1 | 0 | 1 | 1 | 3 |
| hsa-miR-125b-2-3p | MIMAT0004603 | POLE3 | NM_017443 | 1 | 1 | 0 | 0 | 1 | 3 |
| hsa-miR-125b-2-3p | MIMAT0004603 | ERRFI1 | NM_018948 | 0 | 1 | 0 | 1 | 1 | 3 |
| hsa-miR-125b-2-3p | MIMAT0004603 | RBM27 | XM_005268466 | 0 | 1 | 0 | 1 | 1 | 3 |
| hsa-miR-125b-2-3p | MIMAT0004603 | HES2 | XM_005263478 | 1 | 0 | 0 | 1 | 1 | 3 |
| hsa-miR-125b-2-3p | MIMAT0004603 | RSBN1 | NM_018364 | 0 | 1 | 1 | 0 | 1 | 3 |
| hsa-miR-125b-2-3p | MIMAT0004603 | HAUS6 | NM_017645 | 0 | 1 | 0 | 1 | 1 | 3 |
| hsa-miR-125b-2-3p | MIMAT0004603 | SAMD9 | NM_017654 | 1 | 0 | 0 | 1 | 1 | 3 |
| hsa-miR-125b-2-3p | MIMAT0004603 | APTX | NM_001195249 | 1 | 0 | 0 | 1 | 1 | 3 |
| hsa-miR-125b-2-3p | MIMAT0004603 | MFSD6 | NM_017694 | 1 | 0 | 0 | 1 | 1 | 3 |
| hsa-miR-125b-2-3p | MIMAT0004603 | PAQR5 | NM_001104554 | 1 | 0 | 0 | 1 | 1 | 3 |
| hsa-miR-125b-2-3p | MIMAT0004603 | INO80D | XM_005246658 | 0 | 1 | 0 | 1 | 1 | 3 |
| hsa-miR-125b-2-3p | MIMAT0004603 | RNF125 | NM_017831 | 1 | 0 | 0 | 1 | 1 | 3 |
| hsa-miR-125b-2-3p | MIMAT0004603 | TXNL4B | NM_017853 | 1 | 0 | 0 | 1 | 1 | 3 |
| hsa-miR-125b-2-3p | MIMAT0004603 | TMEM248 | XM_005250482 | 1 | 1 | 0 | 0 | 1 | 3 |
| hsa-miR-125b-2-3p | MIMAT0004603 | OXR1 | XM_005250993 | 0 | 1 | 0 | 1 | 1 | 3 |
| hsa-miR-125b-2-3p | MIMAT0004603 | SOBP | NM_018013 | 1 | 1 | 0 | 0 | 1 | 3 |
| hsa-miR-125b-2-3p | MIMAT0004603 | FIGN | XM_005246661 | 1 | 1 | 0 | 0 | 1 | 3 |
| hsa-miR-125b-2-3p | MIMAT0004603 | SLC25A36 | XM_005247575 | 1 | 1 | 0 | 0 | 1 | 3 |
| hsa-miR-125b-2-3p | MIMAT0004603 | VPS13D | NM_015378 | 1 | 0 | 0 | 1 | 1 | 3 |
| hsa-miR-125b-2-3p | MIMAT0004603 | RIC8B | XM_005268998 | 0 | 1 | 0 | 1 | 1 | 3 |
| hsa-miR-125b-2-3p | MIMAT0004603 | RPRD1A | XM_005258294 | 1 | 0 | 0 | 1 | 1 | 3 |
| hsa-miR-125b-2-3p | MIMAT0004603 | C11orf57 | NM_018195 | 0 | 1 | 0 | 1 | 1 | 3 |
| hsa-miR-125b-2-3p | MIMAT0004603 | CCDC25 | NM_018246 | 1 | 0 | 0 | 1 | 1 | 3 |
| hsa-miR-125b-2-3p | MIMAT0004603 | GIMAP4 | NM_018326 | 1 | 0 | 0 | 1 | 1 | 3 |
| hsa-miR-125b-2-3p | MIMAT0004603 | AGPAT5 | NM_018361 | 1 | 1 | 0 | 0 | 1 | 3 |
| hsa-miR-125b-2-3p | MIMAT0004603 | LIN7C | NM_018362 | 1 | 0 | 0 | 1 | 1 | 3 |
| hsa-miR-125b-2-3p | MIMAT0004603 | SLC35C1 | NM_018389 | 1 | 0 | 0 | 1 | 1 | 3 |
| hsa-miR-125b-2-3p | MIMAT0004603 | IMPACT | NM_018439 | 1 | 0 | 0 | 1 | 1 | 3 |
| hsa-miR-125b-2-3p | MIMAT0004603 | MCM10 | NM_182751 | 0 | 1 | 0 | 1 | 1 | 3 |
| hsa-miR-125b-2-3p | MIMAT0004603 | ETNK1 | NM_018638 | 1 | 1 | 0 | 0 | 1 | 3 |
| hsa-miR-125b-2-3p | MIMAT0004603 | CDV3 | NM_001134422 | 1 | 1 | 0 | 0 | 1 | 3 |
| hsa-miR-125b-2-3p | MIMAT0004603 | PPP1R9A | NM_001166160 | 1 | 0 | 0 | 1 | 1 | 3 |
| hsa-miR-125b-2-3p | MIMAT0004603 | SMEK1 | NM_032560 | 1 | 0 | 0 | 1 | 1 | 3 |
| hsa-miR-125b-2-3p | MIMAT0004603 | KANSL3 | XM_005263984 | 0 | 1 | 0 | 1 | 1 | 3 |
| hsa-miR-125b-2-3p | MIMAT0004603 | FRMD4A | XM_005252489 | 0 | 1 | 0 | 1 | 1 | 3 |
| hsa-miR-125b-2-3p | MIMAT0004603 | BTBD7 | NM_001002860 | 1 | 0 | 0 | 1 | 1 | 3 |
| hsa-miR-125b-2-3p | MIMAT0004603 | ENAH | NM_001008493 | 1 | 1 | 0 | 0 | 1 | 3 |
| hsa-miR-125b-2-3p | MIMAT0004603 | EXOC1 | XM_005265751 | 1 | 0 | 0 | 1 | 1 | 3 |
| hsa-miR-125b-2-3p | MIMAT0004603 | H2AFJ | NM_177925 | 1 | 0 | 0 | 1 | 1 | 3 |
| hsa-miR-125b-2-3p | MIMAT0004603 | CSGALNACT1 | NM_001130518 | 1 | 0 | 0 | 1 | 1 | 3 |
| hsa-miR-125b-2-3p | MIMAT0004603 | KDM3A | NM_018433 | 1 | 1 | 0 | 0 | 1 | 3 |
| hsa-miR-125b-2-3p | MIMAT0004603 | WWC3 | NM_015691 | 1 | 0 | 0 | 1 | 1 | 3 |
| hsa-miR-125b-2-3p | MIMAT0004603 | 3-Sep | NM_019106 | 1 | 1 | 0 | 0 | 1 | 3 |
| hsa-miR-125b-2-3p | MIMAT0004603 | UBFD1 | NM_019116 | 1 | 0 | 0 | 1 | 1 | 3 |
| hsa-miR-125b-2-3p | MIMAT0004603 | C21orf62 | NM_001162495 | 1 | 1 | 0 | 0 | 1 | 3 |
| hsa-miR-125b-2-3p | MIMAT0004603 | RNF20 | NM_019592 | 1 | 0 | 0 | 1 | 1 | 3 |
| hsa-miR-125b-2-3p | MIMAT0004603 | GPCPD1 | XM_005260759 | 1 | 0 | 0 | 1 | 1 | 3 |
| hsa-miR-125b-2-3p | MIMAT0004603 | CYP26B1 | NM_019885 | 0 | 1 | 1 | 0 | 1 | 3 |
| hsa-miR-125b-2-3p | MIMAT0004603 | FSTL5 | NM_020116 | 1 | 0 | 0 | 1 | 1 | 3 |
| hsa-miR-125b-2-3p | MIMAT0004603 | SMARCAD1 | NM_001128429 | 1 | 1 | 0 | 0 | 1 | 3 |
| hsa-miR-125b-2-3p | MIMAT0004603 | PAK6 | NM_001276718 | 0 | 1 | 0 | 1 | 1 | 3 |
| hsa-miR-125b-2-3p | MIMAT0004603 | C11orf30 | XM_005274106 | 1 | 0 | 0 | 1 | 1 | 3 |
| hsa-miR-125b-2-3p | MIMAT0004603 | ATXN7L3 | XM_005257526 | 1 | 1 | 0 | 0 | 1 | 3 |
| hsa-miR-125b-2-3p | MIMAT0004603 | SLC17A6 | NM_020346 | 0 | 1 | 0 | 1 | 1 | 3 |
| hsa-miR-125b-2-3p | MIMAT0004603 | GOPC | NM_020399 | 1 | 0 | 0 | 1 | 1 | 3 |
| hsa-miR-125b-2-3p | MIMAT0004603 | SLC39A10 | XM_005246691 | 1 | 0 | 0 | 1 | 1 | 3 |
| hsa-miR-125b-2-3p | MIMAT0004603 | SENP7 | NM_020654 | 1 | 0 | 0 | 1 | 1 | 3 |
| hsa-miR-125b-2-3p | MIMAT0004603 | CYP20A1 | NM_177538 | 1 | 1 | 0 | 0 | 1 | 3 |
| hsa-miR-125b-2-3p | MIMAT0004603 | C3orf14 | XM_005265339 | 1 | 0 | 0 | 1 | 1 | 3 |
| hsa-miR-125b-2-3p | MIMAT0004603 | STRIP2 | NM_020704 | 0 | 1 | 0 | 1 | 1 | 3 |
| hsa-miR-125b-2-3p | MIMAT0004603 | TBC1D24 | NM_001199107 | 1 | 1 | 0 | 0 | 1 | 3 |
| hsa-miR-125b-2-3p | MIMAT0004603 | ERMN | NM_020711 | 0 | 1 | 0 | 1 | 1 | 3 |
| hsa-miR-125b-2-3p | MIMAT0004603 | CNOT6 | XM_005265953 | 1 | 1 | 0 | 0 | 1 | 3 |
| hsa-miR-125b-2-3p | MIMAT0004603 | ZNF512B | XM_005260226 | 1 | 0 | 0 | 1 | 1 | 3 |
| hsa-miR-125b-2-3p | MIMAT0004603 | GRAMD1B | XM_005271620 | 1 | 0 | 0 | 1 | 1 | 3 |
| hsa-miR-125b-2-3p | MIMAT0004603 | USP31 | NM_020718 | 1 | 0 | 0 | 1 | 1 | 3 |
| hsa-miR-125b-2-3p | MIMAT0004603 | ZNF608 | XM_005272039 | 0 | 1 | 0 | 1 | 1 | 3 |
| hsa-miR-125b-2-3p | MIMAT0004603 | MTUS1 | NM_001001924 | 1 | 0 | 0 | 1 | 1 | 3 |
| hsa-miR-125b-2-3p | MIMAT0004603 | SRGAP1 | XM_005269042 | 1 | 0 | 0 | 1 | 1 | 3 |
| hsa-miR-125b-2-3p | MIMAT0004603 | KCTD16 | XM_005268492 | 1 | 1 | 0 | 0 | 1 | 3 |
| hsa-miR-125b-2-3p | MIMAT0004603 | KLHL42 | NM_020782 | 1 | 0 | 0 | 1 | 1 | 3 |
| hsa-miR-125b-2-3p | MIMAT0004603 | FNIP2 | XM_005263156 | 0 | 1 | 0 | 1 | 1 | 3 |
| hsa-miR-125b-2-3p | MIMAT0004603 | DIP2B | NM_173602 | 1 | 0 | 0 | 1 | 1 | 3 |
| hsa-miR-125b-2-3p | MIMAT0004603 | KIAA1467 | NM_020853 | 1 | 0 | 0 | 1 | 1 | 3 |
| hsa-miR-125b-2-3p | MIMAT0004603 | SHROOM3 | NM_020859 | 1 | 0 | 0 | 1 | 1 | 3 |
| hsa-miR-125b-2-3p | MIMAT0004603 | NYAP2 | XM_005246708 | 1 | 0 | 0 | 1 | 1 | 3 |
| hsa-miR-125b-2-3p | MIMAT0004603 | TNRC6C | NM_001142640 | 0 | 1 | 0 | 1 | 1 | 3 |
| hsa-miR-125b-2-3p | MIMAT0004603 | SEMA4G | XM_005270009 | 1 | 0 | 0 | 1 | 1 | 3 |
| hsa-miR-125b-2-3p | MIMAT0004603 | RBM25 | XM_005267956 | 1 | 0 | 0 | 1 | 1 | 3 |
| hsa-miR-125b-2-3p | MIMAT0004603 | NTN4 | NM_021229 | 0 | 1 | 0 | 1 | 1 | 3 |
| hsa-miR-125b-2-3p | MIMAT0004603 | PLEKHA1 | XM_005270017 | 0 | 1 | 0 | 1 | 1 | 3 |
| hsa-miR-125b-2-3p | MIMAT0004603 | ZNF667 | NM_022103 | 0 | 1 | 0 | 1 | 1 | 3 |
| hsa-miR-125b-2-3p | MIMAT0004603 | GOLPH3 | NM_022130 | 1 | 0 | 0 | 1 | 1 | 3 |
| hsa-miR-125b-2-3p | MIMAT0004603 | CLSTN2 | NM_022131 | 1 | 0 | 0 | 1 | 1 | 3 |
| hsa-miR-125b-2-3p | MIMAT0004603 | NSD1 | NM_022455 | 1 | 0 | 0 | 1 | 1 | 3 |
| hsa-miR-125b-2-3p | MIMAT0004603 | LMBR1 | XM_005249554 | 1 | 1 | 0 | 0 | 1 | 3 |
| hsa-miR-125b-2-3p | MIMAT0004603 | GREM2 | NM_022469 | 1 | 0 | 0 | 1 | 1 | 3 |
| hsa-miR-125b-2-3p | MIMAT0004603 | GZF1 | XM_005260799 | 1 | 0 | 0 | 1 | 1 | 3 |
| hsa-miR-125b-2-3p | MIMAT0004603 | EBF2 | NM_022659 | 1 | 0 | 0 | 1 | 1 | 3 |
| hsa-miR-125b-2-3p | MIMAT0004603 | PARP12 | NM_022750 | 0 | 1 | 0 | 1 | 1 | 3 |
| hsa-miR-125b-2-3p | MIMAT0004603 | CREB3L2 | NM_194071 | 1 | 0 | 0 | 1 | 1 | 3 |
| hsa-miR-125b-2-3p | MIMAT0004603 | DDX31 | XM_005272206 | 1 | 0 | 0 | 1 | 1 | 3 |
| hsa-miR-125b-2-3p | MIMAT0004603 | RMND5A | NM_022780 | 1 | 0 | 0 | 1 | 1 | 3 |
| hsa-miR-125b-2-3p | MIMAT0004603 | ELOVL1 | NM_001256399 | 0 | 1 | 0 | 1 | 1 | 3 |
| hsa-miR-125b-2-3p | MIMAT0004603 | FAM129B | NM_022833 | 1 | 0 | 0 | 1 | 1 | 3 |
| hsa-miR-125b-2-3p | MIMAT0004603 | CDCP1 | NM_022842 | 1 | 0 | 0 | 1 | 1 | 3 |
| hsa-miR-125b-2-3p | MIMAT0004603 | BCL11B | NM_138576 | 1 | 0 | 0 | 1 | 1 | 3 |
| hsa-miR-125b-2-3p | MIMAT0004603 | RTN4R | NM_023004 | 0 | 1 | 1 | 0 | 1 | 3 |
| hsa-miR-125b-2-3p | MIMAT0004603 | MARCKSL1 | NM_023009 | 1 | 0 | 0 | 1 | 1 | 3 |
| hsa-miR-125b-2-3p | MIMAT0004603 | PLEKHA3 | NM_019091 | 0 | 1 | 1 | 0 | 1 | 3 |
| hsa-miR-125b-2-3p | MIMAT0004603 | KCTD15 | NM_024076 | 1 | 0 | 0 | 1 | 1 | 3 |
| hsa-miR-125b-2-3p | MIMAT0004603 | WDR77 | NM_024102 | 1 | 0 | 0 | 1 | 1 | 3 |
| hsa-miR-125b-2-3p | MIMAT0004603 | LRRC2 | NM_024512 | 1 | 0 | 0 | 1 | 1 | 3 |
| hsa-miR-125b-2-3p | MIMAT0004603 | FYCO1 | XM_005265483 | 1 | 0 | 0 | 1 | 1 | 3 |
| hsa-miR-125b-2-3p | MIMAT0004603 | FAT4 | XM_005263210 | 0 | 1 | 0 | 1 | 1 | 3 |
| hsa-miR-125b-2-3p | MIMAT0004603 | PLEKHF2 | NM_024613 | 1 | 1 | 0 | 0 | 1 | 3 |
| hsa-miR-125b-2-3p | MIMAT0004603 | QTRTD1 | NM_024638 | 1 | 0 | 0 | 1 | 1 | 3 |
| hsa-miR-125b-2-3p | MIMAT0004603 | NOL9 | NM_024654 | 1 | 0 | 0 | 1 | 1 | 3 |
| hsa-miR-125b-2-3p | MIMAT0004603 | TBL1XR1 | NM_024665 | 1 | 1 | 0 | 0 | 1 | 3 |
| hsa-miR-125b-2-3p | MIMAT0004603 | CLIP4 | XM_005264561 | 0 | 1 | 0 | 1 | 1 | 3 |
| hsa-miR-125b-2-3p | MIMAT0004603 | ZFHX4 | NM_024721 | 0 | 1 | 0 | 1 | 1 | 3 |
| hsa-miR-125b-2-3p | MIMAT0004603 | CPSF7 | NM_024811 | 1 | 0 | 0 | 1 | 1 | 3 |
| hsa-miR-125b-2-3p | MIMAT0004603 | SYNPO2L | NM_001114133 | 1 | 0 | 0 | 1 | 1 | 3 |
| hsa-miR-125b-2-3p | MIMAT0004603 | DHDDS | NM_024887 | 1 | 0 | 0 | 1 | 1 | 3 |
| hsa-miR-125b-2-3p | MIMAT0004603 | ATAT1 | XM_005249420 | 1 | 0 | 0 | 1 | 1 | 3 |
| hsa-miR-125b-2-3p | MIMAT0004603 | CPED1 | NM_024913 | 0 | 1 | 0 | 1 | 1 | 3 |
| hsa-miR-125b-2-3p | MIMAT0004603 | WWC2 | NM_024949 | 0 | 1 | 0 | 1 | 1 | 3 |
| hsa-miR-125b-2-3p | MIMAT0004603 | PGAP1 | XM_005246866 | 1 | 1 | 0 | 0 | 1 | 3 |
| hsa-miR-125b-2-3p | MIMAT0004603 | DCAF17 | NM_025000 | 0 | 1 | 0 | 1 | 1 | 3 |
| hsa-miR-125b-2-3p | MIMAT0004603 | EDC3 | NM_001142443 | 0 | 1 | 0 | 1 | 1 | 3 |
| hsa-miR-125b-2-3p | MIMAT0004603 | HKDC1 | NM_025130 | 0 | 1 | 0 | 1 | 1 | 3 |
| hsa-miR-125b-2-3p | MIMAT0004603 | ACSF2 | NM_025149 | 1 | 0 | 0 | 1 | 1 | 3 |
| hsa-miR-125b-2-3p | MIMAT0004603 | RUFY1 | NM_001040451 | 1 | 0 | 0 | 1 | 1 | 3 |
| hsa-miR-125b-2-3p | MIMAT0004603 | TNKS2 | NM_025235 | 1 | 0 | 0 | 1 | 1 | 3 |
| hsa-miR-125b-2-3p | MIMAT0004603 | ASXL3 | XM_005258356 | 1 | 1 | 0 | 0 | 1 | 3 |
| hsa-miR-125b-2-3p | MIMAT0004603 | CEP44 | NM_001040157 | 0 | 1 | 0 | 1 | 1 | 3 |
| hsa-miR-125b-2-3p | MIMAT0004603 | DUSP16 | NM_030640 | 1 | 1 | 0 | 0 | 1 | 3 |
| hsa-miR-125b-2-3p | MIMAT0004603 | ZFP91 | NM_053023 | 1 | 0 | 0 | 1 | 1 | 3 |
| hsa-miR-125b-2-3p | MIMAT0004603 | CAB39L | NM_030925 | 1 | 0 | 0 | 1 | 1 | 3 |
| hsa-miR-125b-2-3p | MIMAT0004603 | PPP1R14C | NM_030949 | 0 | 1 | 1 | 0 | 1 | 3 |
| hsa-miR-125b-2-3p | MIMAT0004603 | RNF170 | NM_001160223 | 1 | 0 | 0 | 1 | 1 | 3 |
| hsa-miR-125b-2-3p | MIMAT0004603 | NETO1 | XM_005266776 | 0 | 1 | 0 | 1 | 1 | 3 |
| hsa-miR-125b-2-3p | MIMAT0004603 | NUF2 | NM_145697 | 0 | 1 | 1 | 0 | 1 | 3 |
| hsa-miR-125b-2-3p | MIMAT0004603 | FAM107B | NM_031453 | 1 | 0 | 0 | 1 | 1 | 3 |
| hsa-miR-125b-2-3p | MIMAT0004603 | CD99L2 | NM_001242614 | 1 | 0 | 0 | 1 | 1 | 3 |
| hsa-miR-125b-2-3p | MIMAT0004603 | TMTC1 | XM_005253498 | 0 | 1 | 0 | 1 | 1 | 3 |
| hsa-miR-125b-2-3p | MIMAT0004603 | FAM172A | NM_032042 | 1 | 1 | 0 | 0 | 1 | 3 |
| hsa-miR-125b-2-3p | MIMAT0004603 | FAR1 | NM_032228 | 1 | 1 | 0 | 0 | 1 | 3 |
| hsa-miR-125b-2-3p | MIMAT0004603 | SGIP1 | XM_005271263 | 0 | 1 | 0 | 1 | 1 | 3 |
| hsa-miR-125b-2-3p | MIMAT0004603 | NICN1 | NM_032316 | 0 | 1 | 0 | 1 | 1 | 3 |
| hsa-miR-125b-2-3p | MIMAT0004603 | ING5 | NM_032329 | 0 | 1 | 0 | 1 | 1 | 3 |
| hsa-miR-125b-2-3p | MIMAT0004603 | GPR123 | XM_005252695 | 1 | 0 | 0 | 1 | 1 | 3 |
| hsa-miR-125b-2-3p | MIMAT0004603 | ZNF512 | NM_001271318 | 1 | 0 | 0 | 1 | 1 | 3 |
| hsa-miR-125b-2-3p | MIMAT0004603 | JPH4 | NM_032452 | 1 | 1 | 0 | 0 | 1 | 3 |
| hsa-miR-125b-2-3p | MIMAT0004603 | PPAPDC1B | XM_005273661 | 0 | 1 | 0 | 1 | 1 | 3 |
| hsa-miR-125b-2-3p | MIMAT0004603 | RNASE7 | NM_032572 | 1 | 0 | 0 | 1 | 1 | 3 |
| hsa-miR-125b-2-3p | MIMAT0004603 | FAM126A | NM_032581 | 0 | 1 | 0 | 1 | 1 | 3 |
| hsa-miR-125b-2-3p | MIMAT0004603 | TRIM55 | XM_005251316 | 1 | 0 | 0 | 1 | 1 | 3 |
| hsa-miR-125b-2-3p | MIMAT0004603 | ZNF577 | NM_032679 | 1 | 0 | 0 | 1 | 1 | 3 |
| hsa-miR-125b-2-3p | MIMAT0004603 | TRIM52 | NM_032765 | 1 | 1 | 0 | 0 | 1 | 3 |
| hsa-miR-125b-2-3p | MIMAT0004603 | TMEM87B | NM_032824 | 0 | 1 | 0 | 1 | 1 | 3 |
| hsa-miR-125b-2-3p | MIMAT0004603 | FAM104A | XM_005257741 | 0 | 1 | 0 | 1 | 1 | 3 |
| hsa-miR-125b-2-3p | MIMAT0004603 | FBXL20 | NM_032875 | 1 | 1 | 0 | 0 | 1 | 3 |
| hsa-miR-125b-2-3p | MIMAT0004603 | ZC3H12C | XM_005271715 | 0 | 1 | 0 | 1 | 1 | 3 |
| hsa-miR-125b-2-3p | MIMAT0004603 | GTPBP10 | NM_033107 | 0 | 1 | 0 | 1 | 1 | 3 |
| hsa-miR-125b-2-3p | MIMAT0004603 | NAV3 | XM_005269214 | 1 | 0 | 0 | 1 | 1 | 3 |
| hsa-miR-125b-2-3p | MIMAT0004603 | BMF | XM_005254758 | 1 | 0 | 0 | 1 | 1 | 3 |
| hsa-miR-125b-2-3p | MIMAT0004603 | DHX57 | NM_198963 | 1 | 1 | 0 | 0 | 1 | 3 |
| hsa-miR-125b-2-3p | MIMAT0004603 | FMNL3 | NM_175736 | 1 | 1 | 0 | 0 | 1 | 3 |
| hsa-miR-125b-2-3p | MIMAT0004603 | BTF3L4 | NM_152265 | 0 | 1 | 0 | 1 | 1 | 3 |
| hsa-miR-125b-2-3p | MIMAT0004603 | NXPE3 | NM_001134456 | 1 | 1 | 0 | 0 | 1 | 3 |
| hsa-miR-125b-2-3p | MIMAT0004603 | TMEM169 | NM_001142310 | 0 | 1 | 0 | 1 | 1 | 3 |
| hsa-miR-125b-2-3p | MIMAT0004603 | TMEM183A | XM_005245612 | 1 | 1 | 0 | 0 | 1 | 3 |
| hsa-miR-125b-2-3p | MIMAT0004603 | PRDM6 | XM_005272128 | 1 | 0 | 0 | 1 | 1 | 3 |
| hsa-miR-125b-2-3p | MIMAT0004603 | TRMT10A | NM_152292 | 0 | 1 | 0 | 1 | 1 | 3 |
| hsa-miR-125b-2-3p | MIMAT0004603 | CADPS2 | NM_001167940 | 1 | 0 | 0 | 1 | 1 | 3 |
| hsa-miR-125b-2-3p | MIMAT0004603 | ORMDL1 | NM_016467 | 1 | 0 | 0 | 1 | 1 | 3 |
| hsa-miR-125b-2-3p | MIMAT0004603 | TEX261 | NM_144582 | 1 | 0 | 0 | 1 | 1 | 3 |
| hsa-miR-125b-2-3p | MIMAT0004603 | PALM2 | NM_053016 | 1 | 0 | 0 | 1 | 1 | 3 |
| hsa-miR-125b-2-3p | MIMAT0004603 | CCDC85A | NM_001080433 | 0 | 1 | 1 | 0 | 1 | 3 |
| hsa-miR-125b-2-3p | MIMAT0004603 | TMEM200A | NM_052913 | 1 | 1 | 0 | 0 | 1 | 3 |
| hsa-miR-125b-2-3p | MIMAT0004603 | MYSM1 | XM_005270418 | 1 | 0 | 0 | 1 | 1 | 3 |
| hsa-miR-125b-2-3p | MIMAT0004603 | 3-Mar | NM_178450 | 1 | 0 | 0 | 1 | 1 | 3 |
| hsa-miR-125b-2-3p | MIMAT0004603 | NUS1 | NM_138459 | 1 | 0 | 0 | 1 | 1 | 3 |
| hsa-miR-125b-2-3p | MIMAT0004603 | C8orf34 | NM_001195639 | 1 | 0 | 0 | 1 | 1 | 3 |
| hsa-miR-125b-2-3p | MIMAT0004603 | WDR17 | NM_170710 | 1 | 1 | 0 | 0 | 1 | 3 |
| hsa-miR-125b-2-3p | MIMAT0004603 | DACH2 | NM_001139514 | 1 | 0 | 0 | 1 | 1 | 3 |
| hsa-miR-125b-2-3p | MIMAT0004603 | FRMD6 | XM_005267329 | 0 | 1 | 0 | 1 | 1 | 3 |
| hsa-miR-125b-2-3p | MIMAT0004603 | SOCS4 | NM_199421 | 1 | 0 | 0 | 1 | 1 | 3 |
| hsa-miR-125b-2-3p | MIMAT0004603 | SLC24A4 | XM_005267341 | 1 | 1 | 0 | 0 | 1 | 3 |
| hsa-miR-125b-2-3p | MIMAT0004603 | SNX20 | NM_153337 | 1 | 0 | 0 | 1 | 1 | 3 |
| hsa-miR-125b-2-3p | MIMAT0004603 | ZPBP2 | NM_199321 | 1 | 1 | 0 | 0 | 1 | 3 |
| hsa-miR-125b-2-3p | MIMAT0004603 | EMID1 | NM_133455 | 1 | 0 | 0 | 1 | 1 | 3 |
| hsa-miR-125b-2-3p | MIMAT0004603 | TMEM182 | NM_144632 | 1 | 0 | 0 | 1 | 1 | 3 |
| hsa-miR-125b-2-3p | MIMAT0004603 | CHCHD4 | NM_144636 | 1 | 0 | 0 | 1 | 1 | 3 |
| hsa-miR-125b-2-3p | MIMAT0004603 | SGCZ | NM_139167 | 1 | 0 | 0 | 1 | 1 | 3 |
| hsa-miR-125b-2-3p | MIMAT0004603 | SLITRK4 | NM_001184749 | 1 | 0 | 0 | 1 | 1 | 3 |
| hsa-miR-125b-2-3p | MIMAT0004603 | AMER1 | NM_152424 | 1 | 0 | 0 | 1 | 1 | 3 |
| hsa-miR-125b-2-3p | MIMAT0004603 | SIRPA | XM_005260669 | 1 | 0 | 0 | 1 | 1 | 3 |
| hsa-miR-125b-2-3p | MIMAT0004603 | STK35 | NM_080836 | 1 | 0 | 0 | 1 | 1 | 3 |
| hsa-miR-125b-2-3p | MIMAT0004603 | TRUB1 | NM_139169 | 1 | 0 | 0 | 1 | 1 | 3 |
| hsa-miR-125b-2-3p | MIMAT0004603 | SESN3 | NM_144665 | 1 | 1 | 0 | 0 | 1 | 3 |
| hsa-miR-125b-2-3p | MIMAT0004603 | PLEKHA7 | XM_005252801 | 0 | 1 | 0 | 1 | 1 | 3 |
| hsa-miR-125b-2-3p | MIMAT0004603 | RDH12 | NM_152443 | 1 | 0 | 0 | 1 | 1 | 3 |
| hsa-miR-125b-2-3p | MIMAT0004603 | PRIMA1 | NM_178013 | 0 | 1 | 0 | 1 | 1 | 3 |
| hsa-miR-125b-2-3p | MIMAT0004603 | TMCO5A | XM_005254168 | 0 | 1 | 0 | 1 | 1 | 3 |
| hsa-miR-125b-2-3p | MIMAT0004603 | ZFP90 | XM_005255804 | 1 | 1 | 0 | 0 | 1 | 3 |
| hsa-miR-125b-2-3p | MIMAT0004603 | RUNDC1 | XM_005257079 | 0 | 1 | 0 | 1 | 1 | 3 |
| hsa-miR-125b-2-3p | MIMAT0004603 | TMEM56 | NM_001199679 | 1 | 1 | 0 | 0 | 1 | 3 |
| hsa-miR-125b-2-3p | MIMAT0004603 | CDPF1 | NM_207327 | 1 | 1 | 0 | 0 | 1 | 3 |
| hsa-miR-125b-2-3p | MIMAT0004603 | ZNF385B | NM_152520 | 0 | 1 | 0 | 1 | 1 | 3 |
| hsa-miR-125b-2-3p | MIMAT0004603 | CCNYL1 | NM_001142300 | 1 | 0 | 0 | 1 | 1 | 3 |
| hsa-miR-125b-2-3p | MIMAT0004603 | FAM84A | NM_145175 | 1 | 1 | 0 | 0 | 1 | 3 |
| hsa-miR-125b-2-3p | MIMAT0004603 | PAQR3 | NM_001040202 | 1 | 0 | 0 | 1 | 1 | 3 |
| hsa-miR-125b-2-3p | MIMAT0004603 | THAP6 | XM_005262771 | 1 | 1 | 0 | 0 | 1 | 3 |
| hsa-miR-125b-2-3p | MIMAT0004603 | ZMAT2 | XM_005268379 | 1 | 0 | 0 | 1 | 1 | 3 |
| hsa-miR-125b-2-3p | MIMAT0004603 | MBOAT1 | NM_001080480 | 1 | 1 | 0 | 0 | 1 | 3 |
| hsa-miR-125b-2-3p | MIMAT0004603 | RNF217 | XM_005266832 | 1 | 0 | 0 | 1 | 1 | 3 |
| hsa-miR-125b-2-3p | MIMAT0004603 | VKORC1L1 | NM_173517 | 1 | 1 | 0 | 0 | 1 | 3 |
| hsa-miR-125b-2-3p | MIMAT0004603 | AMOTL1 | XM_005273798 | 0 | 1 | 0 | 1 | 1 | 3 |
| hsa-miR-125b-2-3p | MIMAT0004603 | TMEM65 | NM_194291 | 1 | 1 | 0 | 0 | 1 | 3 |
| hsa-miR-125b-2-3p | MIMAT0004603 | MCMDC2 | NM_173518 | 1 | 1 | 0 | 0 | 1 | 3 |
| hsa-miR-125b-2-3p | MIMAT0004603 | SLC5A12 | NM_178498 | 0 | 1 | 0 | 1 | 1 | 3 |
| hsa-miR-125b-2-3p | MIMAT0004603 | TMTC3 | NM_181783 | 1 | 0 | 0 | 1 | 1 | 3 |
| hsa-miR-125b-2-3p | MIMAT0004603 | NOP9 | XM_005267385 | 1 | 0 | 0 | 1 | 1 | 3 |
| hsa-miR-125b-2-3p | MIMAT0004603 | MMAA | NM_172250 | 1 | 1 | 0 | 0 | 1 | 3 |
| hsa-miR-125b-2-3p | MIMAT0004603 | CCDC71L | NM_175884 | 1 | 0 | 0 | 1 | 1 | 3 |
| hsa-miR-125b-2-3p | MIMAT0004603 | COL22A1 | NM_152888 | 0 | 1 | 0 | 1 | 1 | 3 |
| hsa-miR-125b-2-3p | MIMAT0004603 | GLIS3 | NM_001042413 | 1 | 0 | 0 | 1 | 1 | 3 |
| hsa-miR-125b-2-3p | MIMAT0004603 | ADAMTS18 | NM_199355 | 0 | 1 | 0 | 1 | 1 | 3 |
| hsa-miR-125b-2-3p | MIMAT0004603 | CLYBL | NM_206808 | 0 | 1 | 0 | 1 | 1 | 3 |
| hsa-miR-125b-2-3p | MIMAT0004603 | ZNF367 | NM_153695 | 0 | 1 | 0 | 1 | 1 | 3 |
| hsa-miR-125b-2-3p | MIMAT0004603 | LIPH | NM_139248 | 1 | 0 | 0 | 1 | 1 | 3 |
| hsa-miR-125b-2-3p | MIMAT0004603 | FBXO45 | NM_001105573 | 1 | 0 | 0 | 1 | 1 | 3 |
| hsa-miR-125b-2-3p | MIMAT0004603 | TVP23C | NM_001135036 | 1 | 0 | 0 | 1 | 1 | 3 |
| hsa-miR-125b-2-3p | MIMAT0004603 | DENND6A | NM_152678 | 0 | 1 | 0 | 1 | 1 | 3 |
| hsa-miR-125b-2-3p | MIMAT0004603 | TMEM192 | NM_001100389 | 1 | 0 | 0 | 1 | 1 | 3 |
| hsa-miR-125b-2-3p | MIMAT0004603 | NAIF1 | NM_197956 | 1 | 0 | 0 | 1 | 1 | 3 |
| hsa-miR-125b-2-3p | MIMAT0004603 | C2orf69 | NM_153689 | 1 | 0 | 0 | 1 | 1 | 3 |
| hsa-miR-125b-2-3p | MIMAT0004603 | C3orf58 | NM_173552 | 1 | 0 | 0 | 1 | 1 | 3 |
| hsa-miR-125b-2-3p | MIMAT0004603 | SENP5 | NM_152699 | 1 | 0 | 0 | 1 | 1 | 3 |
| hsa-miR-125b-2-3p | MIMAT0004603 | AMER2 | XM_005266279 | 1 | 0 | 0 | 1 | 1 | 3 |
| hsa-miR-125b-2-3p | MIMAT0004603 | RTKN2 | NM_145307 | 1 | 0 | 0 | 1 | 1 | 3 |
| hsa-miR-125b-2-3p | MIMAT0004603 | HNRNPUL2 | NM_001079559 | 1 | 0 | 0 | 1 | 1 | 3 |
| hsa-miR-125b-2-3p | MIMAT0004603 | RBM24 | XM_005248930 | 1 | 1 | 0 | 0 | 1 | 3 |
| hsa-miR-125b-2-3p | MIMAT0004603 | RNF182 | NM_001165032 | 1 | 1 | 0 | 0 | 1 | 3 |
| hsa-miR-125b-2-3p | MIMAT0004603 | FNDC5 | NM_001171941 | 1 | 0 | 0 | 1 | 1 | 3 |
| hsa-miR-125b-2-3p | MIMAT0004603 | RICTOR | XM_005248277 | 0 | 1 | 0 | 1 | 1 | 3 |
| hsa-miR-125b-2-3p | MIMAT0004603 | LCLAT1 | NM_182551 | 0 | 1 | 0 | 1 | 1 | 3 |
| hsa-miR-125b-2-3p | MIMAT0004603 | MMS22L | XM_005266893 | 1 | 0 | 0 | 1 | 1 | 3 |
| hsa-miR-125b-2-3p | MIMAT0004603 | BPIFC | NM_174932 | 1 | 0 | 0 | 1 | 1 | 3 |
| hsa-miR-125b-2-3p | MIMAT0004603 | C8orf46 | NM_152765 | 1 | 0 | 0 | 1 | 1 | 3 |
| hsa-miR-125b-2-3p | MIMAT0004603 | ZDHHC23 | XM_005247266 | 0 | 1 | 0 | 1 | 1 | 3 |
| hsa-miR-125b-2-3p | MIMAT0004603 | NPNT | XM_005262888 | 1 | 0 | 0 | 1 | 1 | 3 |
| hsa-miR-125b-2-3p | MIMAT0004603 | MAP7D2 | NM_001168465 | 1 | 0 | 0 | 1 | 1 | 3 |
| hsa-miR-125b-2-3p | MIMAT0004603 | NEGR1 | NM_173808 | 1 | 1 | 0 | 0 | 1 | 3 |
| hsa-miR-125b-2-3p | MIMAT0004603 | C11orf44 | NM_001271983 | 1 | 0 | 0 | 1 | 1 | 3 |
| hsa-miR-125b-2-3p | MIMAT0004603 | PGM2L1 | NM_173582 | 0 | 1 | 0 | 1 | 1 | 3 |
| hsa-miR-125b-2-3p | MIMAT0004603 | SPRYD4 | NM_207344 | 1 | 0 | 0 | 1 | 1 | 3 |
| hsa-miR-125b-2-3p | MIMAT0004603 | GXYLT1 | NM_173601 | 0 | 1 | 0 | 1 | 1 | 3 |
| hsa-miR-125b-2-3p | MIMAT0004603 | FAM169B | NM_182562 | 0 | 1 | 0 | 1 | 1 | 3 |
| hsa-miR-125b-2-3p | MIMAT0004603 | UNC80 | XM_005246476 | 1 | 0 | 0 | 1 | 1 | 3 |
| hsa-miR-125b-2-3p | MIMAT0004603 | SUMF1 | NM_182760 | 1 | 0 | 0 | 1 | 1 | 3 |
| hsa-miR-125b-2-3p | MIMAT0004603 | C3orf70 | NM_001025266 | 1 | 0 | 0 | 1 | 1 | 3 |
| hsa-miR-125b-2-3p | MIMAT0004603 | LOC285556 | XM_001717423 | 1 | 1 | 0 | 0 | 1 | 3 |
| hsa-miR-125b-2-3p | MIMAT0004603 | ARL10 | XM_005265891 | 1 | 1 | 0 | 0 | 1 | 3 |
| hsa-miR-125b-2-3p | MIMAT0004603 | DTWD2 | NM_173666 | 1 | 1 | 0 | 0 | 1 | 3 |
| hsa-miR-125b-2-3p | MIMAT0004603 | STON1-GTF2A1L | NM_172311 | 1 | 0 | 0 | 1 | 1 | 3 |
| hsa-miR-125b-2-3p | MIMAT0004603 | FMN1 | NM_001277313 | 1 | 1 | 0 | 0 | 1 | 3 |
| hsa-miR-125b-2-3p | MIMAT0004603 | PLCXD3 | NM_001005473 | 1 | 0 | 0 | 1 | 1 | 3 |
| hsa-miR-125b-2-3p | MIMAT0004603 | ZNF530 | NM_020880 | 1 | 0 | 0 | 1 | 1 | 3 |
| hsa-miR-125b-2-3p | MIMAT0004603 | FAM101B | NM_182705 | 1 | 0 | 0 | 1 | 1 | 3 |
| hsa-miR-125b-2-3p | MIMAT0004603 | CCDC172 | NM_198515 | 0 | 1 | 0 | 1 | 1 | 3 |
| hsa-miR-125b-2-3p | MIMAT0004603 | KRT77 | XM_005268853 | 1 | 0 | 0 | 1 | 1 | 3 |
| hsa-miR-125b-2-3p | MIMAT0004603 | ZNF710 | XM_005254908 | 1 | 1 | 0 | 0 | 1 | 3 |
| hsa-miR-125b-2-3p | MIMAT0004603 | SLC26A5 | NM_198999 | 0 | 1 | 1 | 0 | 1 | 3 |
| hsa-miR-125b-2-3p | MIMAT0004603 | ZC3H6 | NM_198581 | 1 | 0 | 0 | 1 | 1 | 3 |
| hsa-miR-125b-2-3p | MIMAT0004603 | C12orf68 | NM_001013635 | 0 | 1 | 0 | 1 | 1 | 3 |
| hsa-miR-125b-2-3p | MIMAT0004603 | IYD | NM_001164694 | 1 | 0 | 0 | 1 | 1 | 3 |
| hsa-miR-125b-2-3p | MIMAT0004603 | C1QL3 | NM_001010908 | 1 | 0 | 0 | 1 | 1 | 3 |
| hsa-miR-125b-2-3p | MIMAT0004603 | ZNF772 | NM_001024596 | 1 | 0 | 0 | 1 | 1 | 3 |
| hsa-miR-125b-2-3p | MIMAT0004603 | PAIP2B | NM_020459 | 1 | 0 | 0 | 1 | 1 | 3 |
| hsa-miR-125b-2-3p | MIMAT0004603 | SNX30 | NM_001012994 | 1 | 0 | 0 | 1 | 1 | 3 |
| hsa-miR-125b-2-3p | MIMAT0004603 | TRIM67 | NM_001004342 | 1 | 1 | 0 | 0 | 1 | 3 |
| hsa-miR-125b-2-3p | MIMAT0004603 | RBMXL1 | NM_001162536 | 1 | 0 | 0 | 1 | 1 | 3 |
| hsa-miR-125b-2-3p | MIMAT0004603 | SFTPA1 | NM_005411 | 0 | 1 | 0 | 1 | 1 | 3 |
| hsa-miR-125b-2-3p | MIMAT0004603 | ZNF814 | NM_001144989 | 1 | 1 | 0 | 0 | 1 | 3 |
| hsa-miR-125b-2-3p | MIMAT0004603 | POM121C | XM_005250082 | 1 | 0 | 0 | 1 | 1 | 3 |
| hsa-miR-125b-2-3p | MIMAT0004603 | KRTAP22-2 | NM_001164434 | 1 | 0 | 0 | 1 | 1 | 3 |
| hsa-miR-125b-2-3p | MIMAT0004603 | ZNF783 | NM_001195220 | 1 | 0 | 0 | 1 | 1 | 3 |
| hsa-miR-125b-2-3p | MIMAT0004603 | FPGT-TNNI3K | NM_001112808 | 0 | 1 | 1 | 0 | 1 | 3 |
| hsa-miR-125b-2-3p | MIMAT0004603 | ABAT | NM_000663 | 1 | 1 | 0 | 0 | 1 | 3 |
| hsa-miR-125b-2-3p | MIMAT0004603 | ACPP | NM_001099 | 1 | 1 | 0 | 0 | 1 | 3 |
| hsa-miR-125b-2-3p | MIMAT0004603 | ACVR2B | XM_005265583 | 1 | 1 | 0 | 0 | 1 | 3 |
| hsa-miR-125b-2-3p | MIMAT0004603 | ANXA8L2 | XM_005272406 | 0 | 1 | 0 | 1 | 1 | 3 |
| hsa-miR-125b-2-3p | MIMAT0004603 | ARF6 | NM_001663 | 1 | 1 | 0 | 0 | 1 | 3 |
| hsa-miR-125b-2-3p | MIMAT0004603 | ARRB1 | NM_004041 | 1 | 1 | 0 | 0 | 1 | 3 |
| hsa-miR-125b-2-3p | MIMAT0004603 | ASL | NM_001024943 | 1 | 1 | 0 | 0 | 1 | 3 |
| hsa-miR-125b-2-3p | MIMAT0004603 | BNIP2 | XM_005254607 | 1 | 1 | 0 | 0 | 1 | 3 |
| hsa-miR-125b-2-3p | MIMAT0004603 | BNIP3 | NM_004052 | 1 | 1 | 0 | 0 | 1 | 3 |
| hsa-miR-125b-2-3p | MIMAT0004603 | BRAF | NM_004333 | 1 | 1 | 0 | 0 | 1 | 3 |
| hsa-miR-125b-2-3p | MIMAT0004603 | CAST | NM_001042440 | 1 | 1 | 0 | 0 | 1 | 3 |
| hsa-miR-125b-2-3p | MIMAT0004603 | TNFSF8 | NM_001244 | 1 | 1 | 0 | 0 | 1 | 3 |
| hsa-miR-125b-2-3p | MIMAT0004603 | CD59 | NM_001127223 | 1 | 1 | 0 | 0 | 1 | 3 |
| hsa-miR-125b-2-3p | MIMAT0004603 | CDH13 | NM_001220488 | 1 | 1 | 0 | 0 | 1 | 3 |
| hsa-miR-125b-2-3p | MIMAT0004603 | CCR5 | NM_000579 | 0 | 1 | 0 | 1 | 1 | 3 |
| hsa-miR-125b-2-3p | MIMAT0004603 | CCR6 | NM_031409 | 0 | 1 | 0 | 1 | 1 | 3 |
| hsa-miR-125b-2-3p | MIMAT0004603 | CNGA4 | XM_005252793 | 1 | 1 | 0 | 0 | 1 | 3 |
| hsa-miR-125b-2-3p | MIMAT0004603 | COX6C | NM_004374 | 1 | 1 | 0 | 0 | 1 | 3 |
| hsa-miR-125b-2-3p | MIMAT0004603 | CPT1B | NM_152245 | 0 | 1 | 0 | 1 | 1 | 3 |
| hsa-miR-125b-2-3p | MIMAT0004603 | CSF2RB | XM_005261340 | 0 | 1 | 0 | 1 | 1 | 3 |
| hsa-miR-125b-2-3p | MIMAT0004603 | CST3 | XM_005260672 | 0 | 1 | 0 | 1 | 1 | 3 |
| hsa-miR-125b-2-3p | MIMAT0004603 | DLX3 | NM_005220 | 0 | 1 | 0 | 1 | 1 | 3 |
| hsa-miR-125b-2-3p | MIMAT0004603 | SARDH | NM_007101 | 0 | 1 | 0 | 1 | 1 | 3 |
| hsa-miR-125b-2-3p | MIMAT0004603 | DNASE1L1 | XM_005274654 | 0 | 1 | 0 | 1 | 1 | 3 |
| hsa-miR-125b-2-3p | MIMAT0004603 | DSG2 | NM_001943 | 1 | 1 | 0 | 0 | 1 | 3 |
| hsa-miR-125b-2-3p | MIMAT0004603 | EDA | NM_001399 | 0 | 1 | 0 | 1 | 1 | 3 |
| hsa-miR-125b-2-3p | MIMAT0004603 | MEGF9 | NM_001080497 | 0 | 1 | 0 | 1 | 1 | 3 |
| hsa-miR-125b-2-3p | MIMAT0004603 | ELF1 | XM_005266277 | 0 | 1 | 0 | 1 | 1 | 3 |
| hsa-miR-125b-2-3p | MIMAT0004603 | CLN8 | XM_005266028 | 1 | 1 | 0 | 0 | 1 | 3 |
| hsa-miR-125b-2-3p | MIMAT0004603 | ERN1 | XM_005257141 | 1 | 1 | 0 | 0 | 1 | 3 |
| hsa-miR-125b-2-3p | MIMAT0004603 | FANCD2 | XM_005264946 | 0 | 1 | 0 | 1 | 1 | 3 |
| hsa-miR-125b-2-3p | MIMAT0004603 | FBLN1 | NM_006487 | 1 | 1 | 0 | 0 | 1 | 3 |
| hsa-miR-125b-2-3p | MIMAT0004603 | FECH | NM_001012515 | 1 | 1 | 0 | 0 | 1 | 3 |
| hsa-miR-125b-2-3p | MIMAT0004603 | FKBP5 | NM_004117 | 1 | 1 | 0 | 0 | 1 | 3 |
| hsa-miR-125b-2-3p | MIMAT0004603 | FOXE3 | NM_012186 | 1 | 1 | 0 | 0 | 1 | 3 |
| hsa-miR-125b-2-3p | MIMAT0004603 | FOXO1 | NM_002015 | 0 | 1 | 0 | 1 | 1 | 3 |
| hsa-miR-125b-2-3p | MIMAT0004603 | FOSB | NM_006732 | 0 | 1 | 0 | 1 | 1 | 3 |
| hsa-miR-125b-2-3p | MIMAT0004603 | GABRB3 | NM_001278631 | 1 | 1 | 0 | 0 | 1 | 3 |
| hsa-miR-125b-2-3p | MIMAT0004603 | B4GALNT1 | NM_001478 | 0 | 1 | 0 | 1 | 1 | 3 |
| hsa-miR-125b-2-3p | MIMAT0004603 | LRRC32 | XM_005273901 | 0 | 1 | 0 | 1 | 1 | 3 |
| hsa-miR-125b-2-3p | MIMAT0004603 | GDF10 | NM_004962 | 0 | 1 | 0 | 1 | 1 | 3 |
| hsa-miR-125b-2-3p | MIMAT0004603 | GPLD1 | NM_001503 | 1 | 1 | 0 | 0 | 1 | 3 |
| hsa-miR-125b-2-3p | MIMAT0004603 | LPAR4 | XM_005262126 | 0 | 1 | 0 | 1 | 1 | 3 |
| hsa-miR-125b-2-3p | MIMAT0004603 | GPR26 | NM_153442 | 1 | 1 | 0 | 0 | 1 | 3 |
| hsa-miR-125b-2-3p | MIMAT0004603 | GRIN2C | NM_000835 | 0 | 1 | 0 | 1 | 1 | 3 |
| hsa-miR-125b-2-3p | MIMAT0004603 | GRM6 | NM_000843 | 0 | 1 | 0 | 1 | 1 | 3 |
| hsa-miR-125b-2-3p | MIMAT0004603 | HK2 | NM_000189 | 0 | 1 | 0 | 1 | 1 | 3 |
| hsa-miR-125b-2-3p | MIMAT0004603 | HLA-DPB1 | NM_002121 | 1 | 1 | 0 | 0 | 1 | 3 |
| hsa-miR-125b-2-3p | MIMAT0004603 | HOXC8 | NM_022658 | 0 | 1 | 1 | 0 | 1 | 3 |
| hsa-miR-125b-2-3p | MIMAT0004603 | HSPA6 | NM_002155 | 0 | 1 | 1 | 0 | 1 | 3 |
| hsa-miR-125b-2-3p | MIMAT0004603 | HSP90AB1 | NM_001271969 | 0 | 1 | 0 | 1 | 1 | 3 |
| hsa-miR-125b-2-3p | MIMAT0004603 | HTR3A | NM_213621 | 0 | 1 | 0 | 1 | 1 | 3 |
| hsa-miR-125b-2-3p | MIMAT0004603 | IDI1 | XM_005252445 | 0 | 1 | 0 | 1 | 1 | 3 |
| hsa-miR-125b-2-3p | MIMAT0004603 | IL9R | NM_176786 | 0 | 1 | 0 | 1 | 1 | 3 |
| hsa-miR-125b-2-3p | MIMAT0004603 | MAOB | XM_005272609 | 1 | 1 | 0 | 0 | 1 | 3 |
| hsa-miR-125b-2-3p | MIMAT0004603 | CHST6 | NM_021615 | 1 | 1 | 0 | 0 | 1 | 3 |
| hsa-miR-125b-2-3p | MIMAT0004603 | MCF2 | XM_005262413 | 0 | 1 | 0 | 1 | 1 | 3 |
| hsa-miR-125b-2-3p | MIMAT0004603 | CIITA | XM_005255320 | 1 | 1 | 0 | 0 | 1 | 3 |
| hsa-miR-125b-2-3p | MIMAT0004603 | MID1 | NM_001193281 | 1 | 1 | 0 | 0 | 1 | 3 |
| hsa-miR-125b-2-3p | MIMAT0004603 | MAP3K9 | NM_033141 | 1 | 1 | 0 | 0 | 1 | 3 |
| hsa-miR-125b-2-3p | MIMAT0004603 | MLLT4 | NM_001040000 | 1 | 1 | 0 | 0 | 1 | 3 |
| hsa-miR-125b-2-3p | MIMAT0004603 | MSR1 | NM_138715 | 1 | 1 | 0 | 0 | 1 | 3 |
| hsa-miR-125b-2-3p | MIMAT0004603 | MTF1 | XM_005270879 | 1 | 1 | 0 | 0 | 1 | 3 |
| hsa-miR-125b-2-3p | MIMAT0004603 | MUC5AC | XM_003960483 | 1 | 1 | 0 | 0 | 1 | 3 |
| hsa-miR-125b-2-3p | MIMAT0004603 | MUSK | XM_005251994 | 1 | 1 | 0 | 0 | 1 | 3 |
| hsa-miR-125b-2-3p | MIMAT0004603 | MYBPH | XM_005245197 | 1 | 1 | 0 | 0 | 1 | 3 |
| hsa-miR-125b-2-3p | MIMAT0004603 | NCK1 | XM_005247498 | 1 | 1 | 0 | 0 | 1 | 3 |
| hsa-miR-125b-2-3p | MIMAT0004603 | NEFL | NM_006158 | 1 | 1 | 0 | 0 | 1 | 3 |
| hsa-miR-125b-2-3p | MIMAT0004603 | NEK3 | NM_152720 | 1 | 1 | 0 | 0 | 1 | 3 |
| hsa-miR-125b-2-3p | MIMAT0004603 | PNP | NM_000270 | 1 | 1 | 0 | 0 | 1 | 3 |
| hsa-miR-125b-2-3p | MIMAT0004603 | NPM1 | XM_005265920 | 1 | 1 | 0 | 0 | 1 | 3 |
| hsa-miR-125b-2-3p | MIMAT0004603 | P2RY2 | NM_176072 | 1 | 1 | 0 | 0 | 1 | 3 |
| hsa-miR-125b-2-3p | MIMAT0004603 | PAK3 | XM_005262131 | 1 | 1 | 0 | 0 | 1 | 3 |
| hsa-miR-125b-2-3p | MIMAT0004603 | PBX2 | NM_002586 | 1 | 1 | 0 | 0 | 1 | 3 |
| hsa-miR-125b-2-3p | MIMAT0004603 | PDCD2 | NM_001199463 | 1 | 1 | 0 | 0 | 1 | 3 |
| hsa-miR-125b-2-3p | MIMAT0004603 | PDE3B | NM_000922 | 1 | 1 | 0 | 0 | 1 | 3 |
| hsa-miR-125b-2-3p | MIMAT0004603 | PIK3R2 | NM_005027 | 1 | 1 | 0 | 0 | 1 | 3 |
| hsa-miR-125b-2-3p | MIMAT0004603 | PLXNA2 | NM_025179 | 1 | 1 | 0 | 0 | 1 | 3 |
| hsa-miR-125b-2-3p | MIMAT0004603 | PML | XM_005254455 | 1 | 1 | 0 | 0 | 1 | 3 |
| hsa-miR-125b-2-3p | MIMAT0004603 | POLR2A | NM_000937 | 1 | 1 | 0 | 0 | 1 | 3 |
| hsa-miR-125b-2-3p | MIMAT0004603 | PRCC | XM_005245313 | 1 | 1 | 0 | 0 | 1 | 3 |
| hsa-miR-125b-2-3p | MIMAT0004603 | PRKCB | NM_002738 | 1 | 1 | 0 | 0 | 1 | 3 |
| hsa-miR-125b-2-3p | MIMAT0004603 | DNAJC3 | NM_006260 | 1 | 1 | 0 | 0 | 1 | 3 |
| hsa-miR-125b-2-3p | MIMAT0004603 | PSMA1 | NM_001143937 | 1 | 1 | 0 | 0 | 1 | 3 |
| hsa-miR-125b-2-3p | MIMAT0004603 | PTPN14 | NM_005401 | 1 | 1 | 0 | 0 | 1 | 3 |
| hsa-miR-125b-2-3p | MIMAT0004603 | PEX19 | NM_002857 | 1 | 1 | 0 | 0 | 1 | 3 |
| hsa-miR-125b-2-3p | MIMAT0004603 | PEX2 | NM_000318 | 1 | 1 | 0 | 0 | 1 | 3 |
| hsa-miR-125b-2-3p | MIMAT0004603 | RAC2 | NM_002872 | 1 | 1 | 0 | 0 | 1 | 3 |
| hsa-miR-125b-2-3p | MIMAT0004603 | RALB | XM_005263728 | 1 | 1 | 0 | 0 | 1 | 3 |
| hsa-miR-125b-2-3p | MIMAT0004603 | RARRES1 | NM_002888 | 1 | 1 | 0 | 0 | 1 | 3 |
| hsa-miR-125b-2-3p | MIMAT0004603 | RBMS2 | NM_002898 | 1 | 1 | 0 | 0 | 1 | 3 |
| hsa-miR-125b-2-3p | MIMAT0004603 | RNF6 | XM_005266485 | 1 | 1 | 0 | 0 | 1 | 3 |
| hsa-miR-125b-2-3p | MIMAT0004603 | RORC | NM_001001523 | 1 | 1 | 0 | 0 | 1 | 3 |
| hsa-miR-125b-2-3p | MIMAT0004603 | RPL13 | NM_033251 | 1 | 1 | 0 | 0 | 1 | 3 |
| hsa-miR-125b-2-3p | MIMAT0004603 | RPL15 | XM_005265363 | 1 | 1 | 0 | 0 | 1 | 3 |
| hsa-miR-125b-2-3p | MIMAT0004603 | RPL37 | NM_000997 | 1 | 1 | 0 | 0 | 1 | 3 |
| hsa-miR-125b-2-3p | MIMAT0004603 | MRPL12 | NM_002949 | 1 | 1 | 0 | 0 | 1 | 3 |
| hsa-miR-125b-2-3p | MIMAT0004603 | RPS3 | NM_001260506 | 1 | 1 | 0 | 0 | 1 | 3 |
| hsa-miR-125b-2-3p | MIMAT0004603 | SC5D | NM_001024956 | 1 | 1 | 0 | 0 | 1 | 3 |
| hsa-miR-125b-2-3p | MIMAT0004603 | SFPQ | XM_005271111 | 1 | 1 | 0 | 0 | 1 | 3 |
| hsa-miR-125b-2-3p | MIMAT0004603 | TRA2B | NM_001243879 | 1 | 1 | 0 | 0 | 1 | 3 |
| hsa-miR-125b-2-3p | MIMAT0004603 | SH3GL2 | NM_003026 | 1 | 1 | 0 | 0 | 1 | 3 |
| hsa-miR-125b-2-3p | MIMAT0004603 | SLC10A2 | NM_000452 | 1 | 1 | 0 | 0 | 1 | 3 |
| hsa-miR-125b-2-3p | MIMAT0004603 | SLC15A2 | NM_021082 | 1 | 1 | 0 | 0 | 1 | 3 |
| hsa-miR-125b-2-3p | MIMAT0004603 | SMS | XM_005274583 | 1 | 1 | 0 | 0 | 1 | 3 |
| hsa-miR-125b-2-3p | MIMAT0004603 | SOX9 | NM_000346 | 1 | 1 | 0 | 0 | 1 | 3 |
| hsa-miR-125b-2-3p | MIMAT0004603 | SPINK2 | NM_001271718 | 1 | 1 | 0 | 0 | 1 | 3 |
| hsa-miR-125b-2-3p | MIMAT0004603 | SPTBN2 | XM_005274192 | 1 | 1 | 0 | 0 | 1 | 3 |
| hsa-miR-125b-2-3p | MIMAT0004603 | TAL2 | NM_005421 | 1 | 1 | 0 | 0 | 1 | 3 |
| hsa-miR-125b-2-3p | MIMAT0004603 | CNTN2 | NM_005076 | 1 | 1 | 0 | 0 | 1 | 3 |
| hsa-miR-125b-2-3p | MIMAT0004603 | TIAL1 | XM_005270109 | 1 | 1 | 0 | 0 | 1 | 3 |
| hsa-miR-125b-2-3p | MIMAT0004603 | TPO | XM_005264698 | 1 | 1 | 0 | 0 | 1 | 3 |
| hsa-miR-125b-2-3p | MIMAT0004603 | TRAF5 | XM_005273248 | 1 | 1 | 0 | 0 | 1 | 3 |
| hsa-miR-125b-2-3p | MIMAT0004603 | TTN | NM_133379 | 1 | 1 | 0 | 0 | 1 | 3 |
| hsa-miR-125b-2-3p | MIMAT0004603 | UCK2 | NM_012474 | 1 | 1 | 0 | 0 | 1 | 3 |
| hsa-miR-125b-2-3p | MIMAT0004603 | UQCRFS1 | NM_006003 | 1 | 1 | 0 | 0 | 1 | 3 |
| hsa-miR-125b-2-3p | MIMAT0004603 | UROS | XM_005270141 | 1 | 1 | 0 | 0 | 1 | 3 |
| hsa-miR-125b-2-3p | MIMAT0004603 | VCL | NM_014000 | 1 | 1 | 0 | 0 | 1 | 3 |
| hsa-miR-125b-2-3p | MIMAT0004603 | NELFA | NM_005663 | 1 | 1 | 0 | 0 | 1 | 3 |
| hsa-miR-125b-2-3p | MIMAT0004603 | ZNF69 | NM_021915 | 1 | 1 | 0 | 0 | 1 | 3 |
| hsa-miR-125b-2-3p | MIMAT0004603 | ZKSCAN8 | NM_001278119 | 1 | 1 | 0 | 0 | 1 | 3 |
| hsa-miR-125b-2-3p | MIMAT0004603 | PRDM2 | NM_012231 | 1 | 1 | 0 | 0 | 1 | 3 |
| hsa-miR-125b-2-3p | MIMAT0004603 | LRP8 | NM_004631 | 1 | 1 | 0 | 0 | 1 | 3 |
| hsa-miR-125b-2-3p | MIMAT0004603 | MAPKAPK3 | NM_001243926 | 1 | 1 | 0 | 0 | 1 | 3 |
| hsa-miR-125b-2-3p | MIMAT0004603 | ST8SIA4 | NM_005668 | 1 | 1 | 0 | 0 | 1 | 3 |
| hsa-miR-125b-2-3p | MIMAT0004603 | MLLT10 | NM_001195627 | 0 | 1 | 1 | 0 | 1 | 3 |
| hsa-miR-125b-2-3p | MIMAT0004603 | TCL1A | NM_021966 | 1 | 1 | 0 | 0 | 1 | 3 |
| hsa-miR-125b-2-3p | MIMAT0004603 | NPRL3 | NM_001243249 | 0 | 1 | 0 | 1 | 1 | 3 |
| hsa-miR-125b-2-3p | MIMAT0004603 | PIAS1 | XM_005254734 | 1 | 1 | 0 | 0 | 1 | 3 |
| hsa-miR-125b-2-3p | MIMAT0004603 | SLC25A12 | NM_003705 | 1 | 1 | 0 | 0 | 1 | 3 |
| hsa-miR-125b-2-3p | MIMAT0004603 | TNKS | XM_005272399 | 0 | 1 | 0 | 1 | 1 | 3 |
| hsa-miR-125b-2-3p | MIMAT0004603 | STX11 | NM_003764 | 1 | 1 | 0 | 0 | 1 | 3 |
| hsa-miR-125b-2-3p | MIMAT0004603 | RIOK3 | NM_003831 | 1 | 1 | 0 | 0 | 1 | 3 |
| hsa-miR-125b-2-3p | MIMAT0004603 | INPP4B | XM_005263325 | 1 | 1 | 0 | 0 | 1 | 3 |
| hsa-miR-125b-2-3p | MIMAT0004603 | SYNGAP1 | XM_005249460 | 1 | 1 | 0 | 0 | 1 | 3 |
| hsa-miR-125b-2-3p | MIMAT0004603 | EIF2S2 | NM_003908 | 0 | 1 | 0 | 1 | 1 | 3 |
| hsa-miR-125b-2-3p | MIMAT0004603 | ENDOU | NM_001172439 | 0 | 1 | 0 | 1 | 1 | 3 |
| hsa-miR-125b-2-3p | MIMAT0004603 | HERC2 | NM_004667 | 0 | 1 | 1 | 0 | 1 | 3 |
| hsa-miR-125b-2-3p | MIMAT0004603 | WASL | NM_003941 | 0 | 1 | 0 | 1 | 1 | 3 |
| hsa-miR-125b-2-3p | MIMAT0004603 | DCLK1 | NM_001195430 | 1 | 1 | 0 | 0 | 1 | 3 |
| hsa-miR-125b-2-3p | MIMAT0004603 | TIAF1 | NM_004740 | 1 | 1 | 0 | 0 | 1 | 3 |
| hsa-miR-125b-2-3p | MIMAT0004603 | TCEAL1 | NM_001006639 | 0 | 1 | 1 | 0 | 1 | 3 |
| hsa-miR-125b-2-3p | MIMAT0004603 | RAB28 | XM_005248216 | 1 | 1 | 0 | 0 | 1 | 3 |
| hsa-miR-125b-2-3p | MIMAT0004603 | HS6ST1 | NM_004807 | 0 | 1 | 0 | 1 | 1 | 3 |
| hsa-miR-125b-2-3p | MIMAT0004603 | ADAMTS1 | NM_006988 | 0 | 1 | 1 | 0 | 1 | 3 |
| hsa-miR-125b-2-3p | MIMAT0004603 | NUP93 | XM_005256263 | 1 | 1 | 0 | 0 | 1 | 3 |
| hsa-miR-125b-2-3p | MIMAT0004603 | RIMS2 | XM_005251108 | 1 | 1 | 0 | 0 | 1 | 3 |
| hsa-miR-125b-2-3p | MIMAT0004603 | KIAA0100 | NM_014680 | 0 | 1 | 0 | 1 | 1 | 3 |
| hsa-miR-125b-2-3p | MIMAT0004603 | SECISBP2L | NM_001193489 | 1 | 1 | 0 | 0 | 1 | 3 |
| hsa-miR-125b-2-3p | MIMAT0004603 | VPRBP | XM_005276754 | 1 | 1 | 0 | 0 | 1 | 3 |
| hsa-miR-125b-2-3p | MIMAT0004603 | ARHGAP32 | NM_001142685 | 0 | 1 | 0 | 1 | 1 | 3 |
| hsa-miR-125b-2-3p | MIMAT0004603 | RAPGEF5 | XM_005249914 | 0 | 1 | 0 | 1 | 1 | 3 |
| hsa-miR-125b-2-3p | MIMAT0004603 | BMS1 | NM_014753 | 1 | 1 | 0 | 0 | 1 | 3 |
| hsa-miR-125b-2-3p | MIMAT0004603 | SLC12A6 | NM_001042496 | 0 | 1 | 1 | 0 | 1 | 3 |
| hsa-miR-125b-2-3p | MIMAT0004603 | HHLA1 | NM_001145095 | 0 | 1 | 0 | 1 | 1 | 3 |
| hsa-miR-125b-2-3p | MIMAT0004603 | ACTR2 | XM_005264080 | 1 | 1 | 0 | 0 | 1 | 3 |
| hsa-miR-125b-2-3p | MIMAT0004603 | LRPPRC | NM_133259 | 0 | 1 | 0 | 1 | 1 | 3 |
| hsa-miR-125b-2-3p | MIMAT0004603 | ZNF197 | XM_005264783 | 0 | 1 | 0 | 1 | 1 | 3 |
| hsa-miR-125b-2-3p | MIMAT0004603 | ZNF443 | XM_005259705 | 1 | 1 | 0 | 0 | 1 | 3 |
| hsa-miR-125b-2-3p | MIMAT0004603 | ARIH2 | XM_005264796 | 0 | 1 | 0 | 1 | 1 | 3 |
| hsa-miR-125b-2-3p | MIMAT0004603 | TAB1 | NM_153497 | 1 | 1 | 0 | 0 | 1 | 3 |
| hsa-miR-125b-2-3p | MIMAT0004603 | VTI1B | XM_005267266 | 1 | 1 | 0 | 0 | 1 | 3 |
| hsa-miR-125b-2-3p | MIMAT0004603 | LEPREL2 | NM_014262 | 1 | 1 | 0 | 0 | 1 | 3 |
| hsa-miR-125b-2-3p | MIMAT0004603 | IGF2BP1 | NM_006546 | 0 | 1 | 0 | 1 | 1 | 3 |
| hsa-miR-125b-2-3p | MIMAT0004603 | SLC12A7 | NM_006598 | 0 | 1 | 0 | 1 | 1 | 3 |
| hsa-miR-125b-2-3p | MIMAT0004603 | ZNF234 | NM_006630 | 0 | 1 | 1 | 0 | 1 | 3 |
| hsa-miR-125b-2-3p | MIMAT0004603 | NEU3 | NM_006656 | 1 | 1 | 0 | 0 | 1 | 3 |
| hsa-miR-125b-2-3p | MIMAT0004603 | FAXDC2 | XM_005268357 | 0 | 1 | 0 | 1 | 1 | 3 |
| hsa-miR-125b-2-3p | MIMAT0004603 | FGL2 | NM_006682 | 0 | 1 | 1 | 0 | 1 | 3 |
| hsa-miR-125b-2-3p | MIMAT0004603 | CFHR3 | XM_005244849 | 1 | 1 | 0 | 0 | 1 | 3 |
| hsa-miR-125b-2-3p | MIMAT0004603 | IFI44L | NM_006820 | 1 | 1 | 0 | 0 | 1 | 3 |
| hsa-miR-125b-2-3p | MIMAT0004603 | ATF7 | XM_005268586 | 0 | 1 | 0 | 1 | 1 | 3 |
| hsa-miR-125b-2-3p | MIMAT0004603 | RAB35 | NM_006861 | 0 | 1 | 0 | 1 | 1 | 3 |
| hsa-miR-125b-2-3p | MIMAT0004603 | LILRB3 | XM_005277289 | 0 | 1 | 0 | 1 | 1 | 3 |
| hsa-miR-125b-2-3p | MIMAT0004603 | RAPGEF4 | NM_007023 | 0 | 1 | 0 | 1 | 1 | 3 |
| hsa-miR-125b-2-3p | MIMAT0004603 | DIDO1 | NM_033081 | 0 | 1 | 0 | 1 | 1 | 3 |
| hsa-miR-125b-2-3p | MIMAT0004603 | ADAMTS5 | NM_007038 | 0 | 1 | 0 | 1 | 1 | 3 |
| hsa-miR-125b-2-3p | MIMAT0004603 | KRR1 | NM_007043 | 1 | 1 | 0 | 0 | 1 | 3 |
| hsa-miR-125b-2-3p | MIMAT0004603 | ERLIN2 | NM_001003791 | 1 | 1 | 0 | 0 | 1 | 3 |
| hsa-miR-125b-2-3p | MIMAT0004603 | NUDT3 | NM_006703 | 1 | 1 | 0 | 0 | 1 | 3 |
| hsa-miR-125b-2-3p | MIMAT0004603 | LZTS1 | XM_005273394 | 0 | 1 | 0 | 1 | 1 | 3 |
| hsa-miR-125b-2-3p | MIMAT0004603 | HPS5 | NM_181507 | 0 | 1 | 0 | 1 | 1 | 3 |
| hsa-miR-125b-2-3p | MIMAT0004603 | SYNRG | XM_005256989 | 1 | 1 | 0 | 0 | 1 | 3 |
| hsa-miR-125b-2-3p | MIMAT0004603 | TFEC | XM_005250231 | 0 | 1 | 0 | 1 | 1 | 3 |
| hsa-miR-125b-2-3p | MIMAT0004603 | IKZF3 | NM_012481 | 1 | 1 | 0 | 0 | 1 | 3 |
| hsa-miR-125b-2-3p | MIMAT0004603 | ZFP30 | XM_005258659 | 0 | 1 | 0 | 1 | 1 | 3 |
| hsa-miR-125b-2-3p | MIMAT0004603 | VASH1 | NM_014909 | 0 | 1 | 0 | 1 | 1 | 3 |
| hsa-miR-125b-2-3p | MIMAT0004603 | LMTK2 | NM_014916 | 1 | 1 | 0 | 0 | 1 | 3 |
| hsa-miR-125b-2-3p | MIMAT0004603 | BAHD1 | XM_005254229 | 0 | 1 | 0 | 1 | 1 | 3 |
| hsa-miR-125b-2-3p | MIMAT0004603 | TRAK1 | XM_005264963 | 1 | 1 | 0 | 0 | 1 | 3 |
| hsa-miR-125b-2-3p | MIMAT0004603 | RRP1B | NM_015056 | 1 | 1 | 0 | 0 | 1 | 3 |
| hsa-miR-125b-2-3p | MIMAT0004603 | GSE1 | XM_005255859 | 0 | 1 | 0 | 1 | 1 | 3 |
| hsa-miR-125b-2-3p | MIMAT0004603 | NUP210 | XM_005264994 | 1 | 1 | 0 | 0 | 1 | 3 |
| hsa-miR-125b-2-3p | MIMAT0004603 | VPS13A | NM_001018038 | 0 | 1 | 1 | 0 | 1 | 3 |
| hsa-miR-125b-2-3p | MIMAT0004603 | EXOC6B | XM_005264223 | 1 | 1 | 0 | 0 | 1 | 3 |
| hsa-miR-125b-2-3p | MIMAT0004603 | PACS2 | NM_001100913 | 1 | 1 | 0 | 0 | 1 | 3 |
| hsa-miR-125b-2-3p | MIMAT0004603 | FAM208A | NM_001112736 | 1 | 1 | 0 | 0 | 1 | 3 |
| hsa-miR-125b-2-3p | MIMAT0004603 | DOCK9 | NM_001130049 | 1 | 1 | 0 | 0 | 1 | 3 |
| hsa-miR-125b-2-3p | MIMAT0004603 | MAU2 | XM_005259837 | 0 | 1 | 0 | 1 | 1 | 3 |
| hsa-miR-125b-2-3p | MIMAT0004603 | SF3B3 | NM_012426 | 1 | 1 | 0 | 0 | 1 | 3 |
| hsa-miR-125b-2-3p | MIMAT0004603 | ABCB10 | NM_012089 | 0 | 1 | 0 | 1 | 1 | 3 |
| hsa-miR-125b-2-3p | MIMAT0004603 | WBP1 | NM_012477 | 1 | 1 | 0 | 0 | 1 | 3 |
| hsa-miR-125b-2-3p | MIMAT0004603 | BACE1 | NM_012104 | 0 | 1 | 0 | 1 | 1 | 3 |
| hsa-miR-125b-2-3p | MIMAT0004603 | NPAP1 | NM_018958 | 0 | 1 | 0 | 1 | 1 | 3 |
| hsa-miR-125b-2-3p | MIMAT0004603 | CNRIP1 | NM_015463 | 1 | 1 | 0 | 0 | 1 | 3 |
| hsa-miR-125b-2-3p | MIMAT0004603 | KIAA1429 | NM_015496 | 0 | 1 | 1 | 0 | 1 | 3 |
| hsa-miR-125b-2-3p | MIMAT0004603 | GORASP2 | NM_015530 | 0 | 1 | 0 | 1 | 1 | 3 |
| hsa-miR-125b-2-3p | MIMAT0004603 | RAB11FIP5 | XM_005264252 | 0 | 1 | 0 | 1 | 1 | 3 |
| hsa-miR-125b-2-3p | MIMAT0004603 | HERC4 | NM_001278187 | 1 | 1 | 0 | 0 | 1 | 3 |
| hsa-miR-125b-2-3p | MIMAT0004603 | FBXW2 | NM_012164 | 1 | 1 | 0 | 0 | 1 | 3 |
| hsa-miR-125b-2-3p | MIMAT0004603 | OR11A1 | XM_005249001 | 0 | 1 | 0 | 1 | 1 | 3 |
| hsa-miR-125b-2-3p | MIMAT0004603 | CKAP2 | NM_001098525 | 1 | 1 | 0 | 0 | 1 | 3 |
| hsa-miR-125b-2-3p | MIMAT0004603 | RANBP6 | NM_001243203 | 1 | 1 | 0 | 0 | 1 | 3 |
| hsa-miR-125b-2-3p | MIMAT0004603 | PDLIM3 | NM_014476 | 1 | 1 | 0 | 0 | 1 | 3 |
| hsa-miR-125b-2-3p | MIMAT0004603 | RAB30 | XM_005273914 | 1 | 1 | 0 | 0 | 1 | 3 |
| hsa-miR-125b-2-3p | MIMAT0004603 | RRP7A | NM_015703 | 1 | 1 | 0 | 0 | 1 | 3 |
| hsa-miR-125b-2-3p | MIMAT0004603 | CDH19 | NM_021153 | 0 | 1 | 0 | 1 | 1 | 3 |
| hsa-miR-125b-2-3p | MIMAT0004603 | WDR91 | NM_014149 | 1 | 1 | 0 | 0 | 1 | 3 |
| hsa-miR-125b-2-3p | MIMAT0004603 | UBQLN1 | XM_005251948 | 1 | 1 | 0 | 0 | 1 | 3 |
| hsa-miR-125b-2-3p | MIMAT0004603 | CD209 | NM_021155 | 0 | 1 | 0 | 1 | 1 | 3 |
| hsa-miR-125b-2-3p | MIMAT0004603 | MED31 | NM_016060 | 0 | 1 | 0 | 1 | 1 | 3 |
| hsa-miR-125b-2-3p | MIMAT0004603 | ASCC1 | XM_005269878 | 1 | 1 | 0 | 0 | 1 | 3 |
| hsa-miR-125b-2-3p | MIMAT0004603 | TNNI3K | NM_015978 | 0 | 1 | 1 | 0 | 1 | 3 |
| hsa-miR-125b-2-3p | MIMAT0004603 | SUV420H1 | NM_016028 | 1 | 1 | 0 | 0 | 1 | 3 |
| hsa-miR-125b-2-3p | MIMAT0004603 | SBDS | NM_016038 | 0 | 1 | 0 | 1 | 1 | 3 |
| hsa-miR-125b-2-3p | MIMAT0004603 | ANGPTL4 | XM_005272484 | 0 | 1 | 0 | 1 | 1 | 3 |
| hsa-miR-125b-2-3p | MIMAT0004603 | NAGPA | NM_016256 | 0 | 1 | 0 | 1 | 1 | 3 |
| hsa-miR-125b-2-3p | MIMAT0004603 | RSL24D1 | NM_016304 | 1 | 1 | 0 | 0 | 1 | 3 |
| hsa-miR-125b-2-3p | MIMAT0004603 | CCDC174 | NM_016474 | 1 | 1 | 0 | 0 | 1 | 3 |
| hsa-miR-125b-2-3p | MIMAT0004603 | RASL12 | XM_005254433 | 0 | 1 | 0 | 1 | 1 | 3 |
| hsa-miR-125b-2-3p | MIMAT0004603 | ZDHHC3 | NM_016598 | 1 | 1 | 0 | 0 | 1 | 3 |
| hsa-miR-125b-2-3p | MIMAT0004603 | ZCCHC17 | NM_016505 | 0 | 1 | 0 | 1 | 1 | 3 |
| hsa-miR-125b-2-3p | MIMAT0004603 | GDE1 | NM_016641 | 0 | 1 | 0 | 1 | 1 | 3 |
| hsa-miR-125b-2-3p | MIMAT0004603 | POLR3K | NM_016310 | 0 | 1 | 1 | 0 | 1 | 3 |
| hsa-miR-125b-2-3p | MIMAT0004603 | ERAP1 | NM_001198541 | 1 | 1 | 0 | 0 | 1 | 3 |
| hsa-miR-125b-2-3p | MIMAT0004603 | ATP8A2 | XM_005266419 | 1 | 1 | 0 | 0 | 1 | 3 |
| hsa-miR-125b-2-3p | MIMAT0004603 | SHC3 | XM_005252052 | 1 | 1 | 0 | 0 | 1 | 3 |
| hsa-miR-125b-2-3p | MIMAT0004603 | GDAP1 | NM_018972 | 0 | 1 | 0 | 1 | 1 | 3 |
| hsa-miR-125b-2-3p | MIMAT0004603 | PLEKHA5 | NM_001143821 | 1 | 1 | 0 | 0 | 1 | 3 |
| hsa-miR-125b-2-3p | MIMAT0004603 | NEURL1B | NM_001142651 | 0 | 1 | 0 | 1 | 1 | 3 |
| hsa-miR-125b-2-3p | MIMAT0004603 | CCDC93 | NM_019044 | 0 | 1 | 0 | 1 | 1 | 3 |
| hsa-miR-125b-2-3p | MIMAT0004603 | DNAJB12 | NM_001002762 | 0 | 1 | 0 | 1 | 1 | 3 |
| hsa-miR-125b-2-3p | MIMAT0004603 | QRICH1 | NM_198880 | 0 | 1 | 1 | 0 | 1 | 3 |
| hsa-miR-125b-2-3p | MIMAT0004603 | UHRF1BP1 | NM_017754 | 1 | 1 | 0 | 0 | 1 | 3 |
| hsa-miR-125b-2-3p | MIMAT0004603 | WHSC1L1 | NM_017778 | 0 | 1 | 1 | 0 | 1 | 3 |
| hsa-miR-125b-2-3p | MIMAT0004603 | ADPRHL2 | NM_017825 | 0 | 1 | 0 | 1 | 1 | 3 |
| hsa-miR-125b-2-3p | MIMAT0004603 | C19orf24 | NM_017914 | 1 | 1 | 0 | 0 | 1 | 3 |
| hsa-miR-125b-2-3p | MIMAT0004603 | PTCD3 | NM_017952 | 0 | 1 | 0 | 1 | 1 | 3 |
| hsa-miR-125b-2-3p | MIMAT0004603 | SLC52A1 | XM_005256709 | 0 | 1 | 0 | 1 | 1 | 3 |
| hsa-miR-125b-2-3p | MIMAT0004603 | RFWD3 | XM_005256021 | 0 | 1 | 0 | 1 | 1 | 3 |
| hsa-miR-125b-2-3p | MIMAT0004603 | ARHGEF10L | XM_005245921 | 0 | 1 | 0 | 1 | 1 | 3 |
| hsa-miR-125b-2-3p | MIMAT0004603 | TMLHE | NM_018196 | 1 | 1 | 0 | 0 | 1 | 3 |
| hsa-miR-125b-2-3p | MIMAT0004603 | UBE2W | NM_001001481 | 1 | 1 | 0 | 0 | 1 | 3 |
| hsa-miR-125b-2-3p | MIMAT0004603 | CSGALNACT2 | XM_005271820 | 1 | 1 | 0 | 0 | 1 | 3 |
| hsa-miR-125b-2-3p | MIMAT0004603 | TRIM36 | NM_018700 | 0 | 1 | 0 | 1 | 1 | 3 |
| hsa-miR-125b-2-3p | MIMAT0004603 | MAML3 | NM_018717 | 1 | 1 | 0 | 0 | 1 | 3 |
| hsa-miR-125b-2-3p | MIMAT0004603 | CCDC132 | NM_001257998 | 1 | 1 | 0 | 0 | 1 | 3 |
| hsa-miR-125b-2-3p | MIMAT0004603 | FLVCR2 | NM_017791 | 0 | 1 | 0 | 1 | 1 | 3 |
| hsa-miR-125b-2-3p | MIMAT0004603 | WDR52 | NM_001164496 | 1 | 1 | 0 | 0 | 1 | 3 |
| hsa-miR-125b-2-3p | MIMAT0004603 | TXLNG | NM_018360 | 0 | 1 | 0 | 1 | 1 | 3 |
| hsa-miR-125b-2-3p | MIMAT0004603 | LRP2BP | NM_018409 | 0 | 1 | 0 | 1 | 1 | 3 |
| hsa-miR-125b-2-3p | MIMAT0004603 | CENPN | NM_001100624 | 0 | 1 | 0 | 1 | 1 | 3 |
| hsa-miR-125b-2-3p | MIMAT0004603 | ZNF395 | XM_005273572 | 0 | 1 | 0 | 1 | 1 | 3 |
| hsa-miR-125b-2-3p | MIMAT0004603 | NCLN | NM_020170 | 0 | 1 | 0 | 1 | 1 | 3 |
| hsa-miR-125b-2-3p | MIMAT0004603 | C14orf132 | NM_001252507 | 1 | 1 | 0 | 0 | 1 | 3 |
| hsa-miR-125b-2-3p | MIMAT0004603 | PRDM11 | NM_001256695 | 1 | 1 | 0 | 0 | 1 | 3 |
| hsa-miR-125b-2-3p | MIMAT0004603 | CABP4 | NM_145200 | 1 | 1 | 0 | 0 | 1 | 3 |
| hsa-miR-125b-2-3p | MIMAT0004603 | TWSG1 | NM_020648 | 0 | 1 | 0 | 1 | 1 | 3 |
| hsa-miR-125b-2-3p | MIMAT0004603 | ENTPD7 | NM_020354 | 1 | 1 | 0 | 0 | 1 | 3 |
| hsa-miR-125b-2-3p | MIMAT0004603 | JPH3 | NM_001271604 | 1 | 1 | 0 | 0 | 1 | 3 |
| hsa-miR-125b-2-3p | MIMAT0004603 | RNF150 | XM_005263150 | 1 | 1 | 0 | 0 | 1 | 3 |
| hsa-miR-125b-2-3p | MIMAT0004603 | ESYT2 | XM_005249551 | 0 | 1 | 0 | 1 | 1 | 3 |
| hsa-miR-125b-2-3p | MIMAT0004603 | KIAA1328 | XM_005258317 | 0 | 1 | 0 | 1 | 1 | 3 |
| hsa-miR-125b-2-3p | MIMAT0004603 | PDP2 | NM_020786 | 1 | 1 | 0 | 0 | 1 | 3 |
| hsa-miR-125b-2-3p | MIMAT0004603 | MICAL3 | NM_001136004 | 1 | 1 | 0 | 0 | 1 | 3 |
| hsa-miR-125b-2-3p | MIMAT0004603 | DPP10 | XM_005263718 | 1 | 1 | 0 | 0 | 1 | 3 |
| hsa-miR-125b-2-3p | MIMAT0004603 | ALS2 | NM_001135745 | 0 | 1 | 1 | 0 | 1 | 3 |
| hsa-miR-125b-2-3p | MIMAT0004603 | SLC7A14 | NM_020949 | 1 | 1 | 0 | 0 | 1 | 3 |
| hsa-miR-125b-2-3p | MIMAT0004603 | SLAMF7 | NM_021181 | 0 | 1 | 0 | 1 | 1 | 3 |
| hsa-miR-125b-2-3p | MIMAT0004603 | SRR | NM_021947 | 0 | 1 | 0 | 1 | 1 | 3 |
| hsa-miR-125b-2-3p | MIMAT0004603 | SH2D4A | NM_022071 | 1 | 1 | 0 | 0 | 1 | 3 |
| hsa-miR-125b-2-3p | MIMAT0004603 | C17orf75 | NM_022344 | 1 | 1 | 0 | 0 | 1 | 3 |
| hsa-miR-125b-2-3p | MIMAT0004603 | PAPD5 | NM_001040284 | 1 | 1 | 0 | 0 | 1 | 3 |
| hsa-miR-125b-2-3p | MIMAT0004603 | NOM1 | NM_138400 | 0 | 1 | 0 | 1 | 1 | 3 |
| hsa-miR-125b-2-3p | MIMAT0004603 | CSMD1 | NM_033225 | 1 | 1 | 0 | 0 | 1 | 3 |
| hsa-miR-125b-2-3p | MIMAT0004603 | FBXL17 | XM_005272049 | 1 | 1 | 0 | 0 | 1 | 3 |
| hsa-miR-125b-2-3p | MIMAT0004603 | PAPOLG | NM_022894 | 1 | 1 | 0 | 0 | 1 | 3 |
| hsa-miR-125b-2-3p | MIMAT0004603 | CASD1 | NM_022900 | 0 | 1 | 1 | 0 | 1 | 3 |
| hsa-miR-125b-2-3p | MIMAT0004603 | REEP1 | XM_005264502 | 0 | 1 | 0 | 1 | 1 | 3 |
| hsa-miR-125b-2-3p | MIMAT0004603 | RASL11B | NM_023940 | 1 | 1 | 0 | 0 | 1 | 3 |
| hsa-miR-125b-2-3p | MIMAT0004603 | TRAK2 | NM_015049 | 1 | 1 | 0 | 0 | 1 | 3 |
| hsa-miR-125b-2-3p | MIMAT0004603 | DUSP26 | XM_005273633 | 0 | 1 | 0 | 1 | 1 | 3 |
| hsa-miR-125b-2-3p | MIMAT0004603 | TMEM185B | NM_024121 | 1 | 1 | 0 | 0 | 1 | 3 |
| hsa-miR-125b-2-3p | MIMAT0004603 | GNPTAB | NM_024312 | 0 | 1 | 0 | 1 | 1 | 3 |
| hsa-miR-125b-2-3p | MIMAT0004603 | CHPF | NM_024536 | 1 | 1 | 0 | 0 | 1 | 3 |
| hsa-miR-125b-2-3p | MIMAT0004603 | MUL1 | XM_005246001 | 0 | 1 | 0 | 1 | 1 | 3 |
| hsa-miR-125b-2-3p | MIMAT0004603 | HMBOX1 | XM_005273634 | 1 | 1 | 0 | 0 | 1 | 3 |
| hsa-miR-125b-2-3p | MIMAT0004603 | SH3TC2 | NM_024577 | 1 | 1 | 0 | 0 | 1 | 3 |
| hsa-miR-125b-2-3p | MIMAT0004603 | SRD5A3 | NM_024592 | 0 | 1 | 0 | 1 | 1 | 3 |
| hsa-miR-125b-2-3p | MIMAT0004603 | MCPH1 | XM_005266034 | 0 | 1 | 0 | 1 | 1 | 3 |
| hsa-miR-125b-2-3p | MIMAT0004603 | MANEA | NM_024641 | 0 | 1 | 0 | 1 | 1 | 3 |
| hsa-miR-125b-2-3p | MIMAT0004603 | ZNF768 | XM_005255577 | 0 | 1 | 0 | 1 | 1 | 3 |
| hsa-miR-125b-2-3p | MIMAT0004603 | DHRS12 | NM_024705 | 1 | 1 | 0 | 0 | 1 | 3 |
| hsa-miR-125b-2-3p | MIMAT0004603 | AGBL2 | XM_005253137 | 0 | 1 | 0 | 1 | 1 | 3 |
| hsa-miR-125b-2-3p | MIMAT0004603 | NAA60 | XM_005255590 | 1 | 1 | 0 | 0 | 1 | 3 |
| hsa-miR-125b-2-3p | MIMAT0004603 | FAM192A | XM_005256156 | 0 | 1 | 0 | 1 | 1 | 3 |
| hsa-miR-125b-2-3p | MIMAT0004603 | EFHD1 | NM_025202 | 1 | 1 | 0 | 0 | 1 | 3 |
| hsa-miR-125b-2-3p | MIMAT0004603 | SPHKAP | NM_030623 | 0 | 1 | 1 | 0 | 1 | 3 |
| hsa-miR-125b-2-3p | MIMAT0004603 | WNT5B | XM_005253793 | 0 | 1 | 0 | 1 | 1 | 3 |
| hsa-miR-125b-2-3p | MIMAT0004603 | SLC38A1 | NM_001278387 | 1 | 1 | 0 | 0 | 1 | 3 |
| hsa-miR-125b-2-3p | MIMAT0004603 | TMX1 | NM_030755 | 1 | 1 | 0 | 0 | 1 | 3 |
| hsa-miR-125b-2-3p | MIMAT0004603 | ACSBG2 | XM_005259652 | 0 | 1 | 0 | 1 | 1 | 3 |
| hsa-miR-125b-2-3p | MIMAT0004603 | ST6GALNAC5 | XM_005271238 | 1 | 1 | 0 | 0 | 1 | 3 |
| hsa-miR-125b-2-3p | MIMAT0004603 | DNAL1 | NM_001201366 | 1 | 1 | 0 | 0 | 1 | 3 |
| hsa-miR-125b-2-3p | MIMAT0004603 | KCTD10 | XM_005253945 | 0 | 1 | 0 | 1 | 1 | 3 |
| hsa-miR-125b-2-3p | MIMAT0004603 | FSCB | NM_032135 | 0 | 1 | 1 | 0 | 1 | 3 |
| hsa-miR-125b-2-3p | MIMAT0004603 | USP44 | XM_005269173 | 1 | 1 | 0 | 0 | 1 | 3 |
| hsa-miR-125b-2-3p | MIMAT0004603 | ZCCHC7 | NM_032226 | 0 | 1 | 1 | 0 | 1 | 3 |
| hsa-miR-125b-2-3p | MIMAT0004603 | LRRC8C | NM_032270 | 0 | 1 | 0 | 1 | 1 | 3 |
| hsa-miR-125b-2-3p | MIMAT0004603 | DDI2 | XM_005246017 | 1 | 1 | 0 | 0 | 1 | 3 |
| hsa-miR-125b-2-3p | MIMAT0004603 | MRPL45 | NM_032351 | 1 | 1 | 0 | 0 | 1 | 3 |
| hsa-miR-125b-2-3p | MIMAT0004603 | CCDC115 | XM_005263825 | 1 | 1 | 0 | 0 | 1 | 3 |
| hsa-miR-125b-2-3p | MIMAT0004603 | TTBK1 | XM_005249449 | 1 | 1 | 0 | 0 | 1 | 3 |
| hsa-miR-125b-2-3p | MIMAT0004603 | SLC9A7 | NM_001257291 | 1 | 1 | 0 | 0 | 1 | 3 |
| hsa-miR-125b-2-3p | MIMAT0004603 | GPT2 | NM_133443 | 0 | 1 | 0 | 1 | 1 | 3 |
| hsa-miR-125b-2-3p | MIMAT0004603 | PLXDC2 | NM_032812 | 1 | 1 | 0 | 0 | 1 | 3 |
| hsa-miR-125b-2-3p | MIMAT0004603 | ARHGAP19 | NM_032900 | 0 | 1 | 0 | 1 | 1 | 3 |
| hsa-miR-125b-2-3p | MIMAT0004603 | USP45 | NM_001080481 | 1 | 1 | 0 | 0 | 1 | 3 |
| hsa-miR-125b-2-3p | MIMAT0004603 | C9orf69 | NM_152833 | 1 | 1 | 0 | 0 | 1 | 3 |
| hsa-miR-125b-2-3p | MIMAT0004603 | LYRM7 | NM_181705 | 0 | 1 | 0 | 1 | 1 | 3 |
| hsa-miR-125b-2-3p | MIMAT0004603 | TGIF2LY | NM_139214 | 0 | 1 | 0 | 1 | 1 | 3 |
| hsa-miR-125b-2-3p | MIMAT0004603 | TBL1Y | XM_005262573 | 1 | 1 | 0 | 0 | 1 | 3 |
| hsa-miR-125b-2-3p | MIMAT0004603 | PPP1R3E | XM_005268195 | 1 | 1 | 0 | 0 | 1 | 3 |
| hsa-miR-125b-2-3p | MIMAT0004603 | OXNAD1 | NM_138381 | 0 | 1 | 0 | 1 | 1 | 3 |
| hsa-miR-125b-2-3p | MIMAT0004603 | ZNF585B | NM_152279 | 1 | 1 | 0 | 0 | 1 | 3 |
| hsa-miR-125b-2-3p | MIMAT0004603 | ZNF670 | NM_033213 | 1 | 1 | 0 | 0 | 1 | 3 |
| hsa-miR-125b-2-3p | MIMAT0004603 | PIGS | NM_033198 | 0 | 1 | 0 | 1 | 1 | 3 |
| hsa-miR-125b-2-3p | MIMAT0004603 | TP53RK | NM_033550 | 1 | 1 | 0 | 0 | 1 | 3 |
| hsa-miR-125b-2-3p | MIMAT0004603 | PLCD3 | NM_133373 | 0 | 1 | 0 | 1 | 1 | 3 |
| hsa-miR-125b-2-3p | MIMAT0004603 | MAL2 | NM_052886 | 1 | 1 | 0 | 0 | 1 | 3 |
| hsa-miR-125b-2-3p | MIMAT0004603 | CSMD2 | NM_052896 | 0 | 1 | 0 | 1 | 1 | 3 |
| hsa-miR-125b-2-3p | MIMAT0004603 | SLC25A25 | NM_001265614 | 0 | 1 | 0 | 1 | 1 | 3 |
| hsa-miR-125b-2-3p | MIMAT0004603 | RNF157 | NM_052916 | 0 | 1 | 0 | 1 | 1 | 3 |
| hsa-miR-125b-2-3p | MIMAT0004603 | OMA1 | XM_005270422 | 1 | 1 | 0 | 0 | 1 | 3 |
| hsa-miR-125b-2-3p | MIMAT0004603 | FCRL3 | XM_005244872 | 1 | 1 | 0 | 0 | 1 | 3 |
| hsa-miR-125b-2-3p | MIMAT0004603 | RBP7 | NM_052960 | 0 | 1 | 0 | 1 | 1 | 3 |
| hsa-miR-125b-2-3p | MIMAT0004603 | IL22RA2 | NM_181310 | 1 | 1 | 0 | 0 | 1 | 3 |
| hsa-miR-125b-2-3p | MIMAT0004603 | COMTD1 | XM_005269513 | 1 | 1 | 0 | 0 | 1 | 3 |
| hsa-miR-125b-2-3p | MIMAT0004603 | CPXM2 | XM_005269528 | 0 | 1 | 0 | 1 | 1 | 3 |
| hsa-miR-125b-2-3p | MIMAT0004603 | MTFMT | NM_139242 | 1 | 1 | 0 | 0 | 1 | 3 |
| hsa-miR-125b-2-3p | MIMAT0004603 | NIPA1 | NM_144599 | 0 | 1 | 0 | 1 | 1 | 3 |
| hsa-miR-125b-2-3p | MIMAT0004603 | ACSM2A | NM_001010845 | 0 | 1 | 0 | 1 | 1 | 3 |
| hsa-miR-125b-2-3p | MIMAT0004603 | OVCA2 | NM_080822 | 1 | 1 | 0 | 0 | 1 | 3 |
| hsa-miR-125b-2-3p | MIMAT0004603 | UBXN10 | XM_005245742 | 1 | 1 | 0 | 0 | 1 | 3 |
| hsa-miR-125b-2-3p | MIMAT0004603 | SYT2 | NM_001136504 | 1 | 1 | 0 | 0 | 1 | 3 |
| hsa-miR-125b-2-3p | MIMAT0004603 | TSHZ2 | NM_173485 | 1 | 1 | 0 | 0 | 1 | 3 |
| hsa-miR-125b-2-3p | MIMAT0004603 | STARD4 | XM_005271881 | 1 | 1 | 0 | 0 | 1 | 3 |
| hsa-miR-125b-2-3p | MIMAT0004603 | STXBP5 | NM_001127715 | 1 | 1 | 0 | 0 | 1 | 3 |
| hsa-miR-125b-2-3p | MIMAT0004603 | PTCHD1 | NM_173495 | 1 | 1 | 0 | 0 | 1 | 3 |
| hsa-miR-125b-2-3p | MIMAT0004603 | TMEM37 | XM_005263597 | 0 | 1 | 0 | 1 | 1 | 3 |
| hsa-miR-125b-2-3p | MIMAT0004603 | SPTY2D1 | NM_194285 | 1 | 1 | 0 | 0 | 1 | 3 |
| hsa-miR-125b-2-3p | MIMAT0004603 | RAD9B | XM_005253849 | 1 | 1 | 0 | 0 | 1 | 3 |
| hsa-miR-125b-2-3p | MIMAT0004603 | B3GALTL | NM_194318 | 0 | 1 | 0 | 1 | 1 | 3 |
| hsa-miR-125b-2-3p | MIMAT0004603 | ZNF417 | NM_152475 | 1 | 1 | 0 | 0 | 1 | 3 |
| hsa-miR-125b-2-3p | MIMAT0004603 | LSM14B | NM_144703 | 0 | 1 | 0 | 1 | 1 | 3 |
| hsa-miR-125b-2-3p | MIMAT0004603 | KANSL1L | XM_005246334 | 1 | 1 | 0 | 0 | 1 | 3 |
| hsa-miR-125b-2-3p | MIMAT0004603 | PPM1K | NM_152542 | 1 | 1 | 0 | 0 | 1 | 3 |
| hsa-miR-125b-2-3p | MIMAT0004603 | PLEKHG4B | NM_052909 | 1 | 1 | 0 | 0 | 1 | 3 |
| hsa-miR-125b-2-3p | MIMAT0004603 | C9orf62 | NM_173520 | 0 | 1 | 0 | 1 | 1 | 3 |
| hsa-miR-125b-2-3p | MIMAT0004603 | AWAT2 | NM_001002254 | 0 | 1 | 0 | 1 | 1 | 3 |
| hsa-miR-125b-2-3p | MIMAT0004603 | DENND1B | XM_005244933 | 1 | 1 | 0 | 0 | 1 | 3 |
| hsa-miR-125b-2-3p | MIMAT0004603 | C1orf177 | NM_152607 | 0 | 1 | 0 | 1 | 1 | 3 |
| hsa-miR-125b-2-3p | MIMAT0004603 | ZNF800 | XM_005250182 | 1 | 1 | 0 | 0 | 1 | 3 |
| hsa-miR-125b-2-3p | MIMAT0004603 | SPIN3 | NM_001010862 | 1 | 1 | 0 | 0 | 1 | 3 |
| hsa-miR-125b-2-3p | MIMAT0004603 | SUPT20HL2 | XM_005274452 | 1 | 1 | 0 | 0 | 1 | 3 |
| hsa-miR-125b-2-3p | MIMAT0004603 | FAM9C | XM_005274463 | 0 | 1 | 0 | 1 | 1 | 3 |
| hsa-miR-125b-2-3p | MIMAT0004603 | GRAMD2 | NM_001012642 | 0 | 1 | 0 | 1 | 1 | 3 |
| hsa-miR-125b-2-3p | MIMAT0004603 | ZNF778 | XM_005256291 | 1 | 1 | 0 | 0 | 1 | 3 |
| hsa-miR-125b-2-3p | MIMAT0004603 | SLFNL1 | XM_005270596 | 1 | 1 | 0 | 0 | 1 | 3 |
| hsa-miR-125b-2-3p | MIMAT0004603 | CENPV | XM_005256512 | 1 | 1 | 0 | 0 | 1 | 3 |
| hsa-miR-125b-2-3p | MIMAT0004603 | TSNARE1 | XM_005250831 | 1 | 1 | 0 | 0 | 1 | 3 |
| hsa-miR-125b-2-3p | MIMAT0004603 | AMER3 | NM_152698 | 1 | 1 | 0 | 0 | 1 | 3 |
| hsa-miR-125b-2-3p | MIMAT0004603 | ATOH7 | NM_145178 | 1 | 1 | 0 | 0 | 1 | 3 |
| hsa-miR-125b-2-3p | MIMAT0004603 | RNASEH1 | XM_005263850 | 1 | 1 | 0 | 0 | 1 | 3 |
| hsa-miR-125b-2-3p | MIMAT0004603 | ANKRD18A | NM_147195 | 1 | 1 | 0 | 0 | 1 | 3 |
| hsa-miR-125b-2-3p | MIMAT0004603 | CALHM1 | NM_001001412 | 0 | 1 | 0 | 1 | 1 | 3 |
| hsa-miR-125b-2-3p | MIMAT0004603 | CCDC108 | NM_152389 | 0 | 1 | 1 | 0 | 1 | 3 |
| hsa-miR-125b-2-3p | MIMAT0004603 | TXNDC8 | XM_005251881 | 1 | 1 | 0 | 0 | 1 | 3 |
| hsa-miR-125b-2-3p | MIMAT0004603 | SERINC5 | NM_001174071 | 1 | 1 | 0 | 0 | 1 | 3 |
| hsa-miR-125b-2-3p | MIMAT0004603 | KSR2 | NM_173598 | 1 | 1 | 0 | 0 | 1 | 3 |
| hsa-miR-125b-2-3p | MIMAT0004603 | ZNF780A | NM_001142579 | 0 | 1 | 0 | 1 | 1 | 3 |
| hsa-miR-125b-2-3p | MIMAT0004603 | RIMKLA | NM_173642 | 1 | 1 | 0 | 0 | 1 | 3 |
| hsa-miR-125b-2-3p | MIMAT0004603 | C20orf197 | NM_173644 | 0 | 1 | 0 | 1 | 1 | 3 |
| hsa-miR-125b-2-3p | MIMAT0004603 | LOC285500 | XM_005275964 | 1 | 1 | 0 | 0 | 1 | 3 |
| hsa-miR-125b-2-3p | MIMAT0004603 | SCAI | NM_173690 | 1 | 1 | 0 | 0 | 1 | 3 |
| hsa-miR-125b-2-3p | MIMAT0004603 | BRINP3 | XM_005245123 | 1 | 1 | 0 | 0 | 1 | 3 |
| hsa-miR-125b-2-3p | MIMAT0004603 | ACER2 | XM_005251448 | 1 | 1 | 0 | 0 | 1 | 3 |
| hsa-miR-125b-2-3p | MIMAT0004603 | VSIG1 | NM_001170553 | 0 | 1 | 1 | 0 | 1 | 3 |
| hsa-miR-125b-2-3p | MIMAT0004603 | KCNT2 | XM_005245133 | 0 | 1 | 0 | 1 | 1 | 3 |
| hsa-miR-125b-2-3p | MIMAT0004603 | OSTN | XM_005247428 | 1 | 1 | 0 | 0 | 1 | 3 |
| hsa-miR-125b-2-3p | MIMAT0004603 | C15orf38 | NM_182616 | 1 | 1 | 0 | 0 | 1 | 3 |
| hsa-miR-125b-2-3p | MIMAT0004603 | ZNF445 | NM_181489 | 0 | 1 | 0 | 1 | 1 | 3 |
| hsa-miR-125b-2-3p | MIMAT0004603 | ILDR2 | NM_199351 | 1 | 1 | 0 | 0 | 1 | 3 |
| hsa-miR-125b-2-3p | MIMAT0004603 | CLEC12B | NM_001129998 | 1 | 1 | 0 | 0 | 1 | 3 |
| hsa-miR-125b-2-3p | MIMAT0004603 | RTL1 | XM_005267632 | 1 | 1 | 0 | 0 | 1 | 3 |
| hsa-miR-125b-2-3p | MIMAT0004603 | TMEM179 | NM_207379 | 1 | 1 | 0 | 0 | 1 | 3 |
| hsa-miR-125b-2-3p | MIMAT0004603 | SBK1 | NM_001024401 | 0 | 1 | 0 | 1 | 1 | 3 |
| hsa-miR-125b-2-3p | MIMAT0004603 | FAM211A | NM_001113567 | 0 | 1 | 0 | 1 | 1 | 3 |
| hsa-miR-125b-2-3p | MIMAT0004603 | TMIGD1 | NM_206832 | 0 | 1 | 1 | 0 | 1 | 3 |
| hsa-miR-125b-2-3p | MIMAT0004603 | LDLRAD1 | NM_001010978 | 1 | 1 | 0 | 0 | 1 | 3 |
| hsa-miR-125b-2-3p | MIMAT0004603 | PABPN1L | NM_001080487 | 0 | 1 | 0 | 1 | 1 | 3 |
| hsa-miR-125b-2-3p | MIMAT0004603 | ZNF805 | NM_001023563 | 1 | 1 | 0 | 0 | 1 | 3 |
| hsa-miR-125b-2-3p | MIMAT0004603 | MYO18A | XM_005257977 | 1 | 1 | 0 | 0 | 1 | 3 |
| hsa-miR-125b-2-3p | MIMAT0004603 | C11orf88 | NM_207430 | 0 | 1 | 1 | 0 | 1 | 3 |
| hsa-miR-125b-2-3p | MIMAT0004603 | RGS7BP | XM_005248503 | 0 | 1 | 0 | 1 | 1 | 3 |
| hsa-miR-125b-2-3p | MIMAT0004603 | CRIP3 | NM_206922 | 0 | 1 | 1 | 0 | 1 | 3 |
| hsa-miR-125b-2-3p | MIMAT0004603 | PTPLAD2 | XM_005251459 | 1 | 1 | 0 | 0 | 1 | 3 |
| hsa-miR-125b-2-3p | MIMAT0004603 | BCL2L15 | NM_001010922 | 1 | 1 | 0 | 0 | 1 | 3 |
| hsa-miR-125b-2-3p | MIMAT0004603 | ANKRD18B | NM_001244752 | 1 | 1 | 0 | 0 | 1 | 3 |
| hsa-miR-125b-2-3p | MIMAT0004603 | CT45A5 | NM_001172288 | 0 | 1 | 1 | 0 | 1 | 3 |
| hsa-miR-125b-2-3p | MIMAT0004603 | DUXA | NM_001012729 | 1 | 1 | 0 | 0 | 1 | 3 |
| hsa-miR-125b-2-3p | MIMAT0004603 | FAM110C | XM_005264697 | 1 | 1 | 0 | 0 | 1 | 3 |
| hsa-miR-125b-2-3p | MIMAT0004603 | MROH6 | NM_001100878 | 1 | 1 | 0 | 0 | 1 | 3 |
| hsa-miR-125b-2-3p | MIMAT0004603 | PPIAL4D | NM_001164261 | 1 | 1 | 0 | 0 | 1 | 3 |
| hsa-miR-125b-2-3p | MIMAT0004603 | LOC649201 | XM_005276118 | 1 | 1 | 0 | 0 | 1 | 3 |
| hsa-miR-125b-2-3p | MIMAT0004603 | ANXA8 | NM_001271702 | 0 | 1 | 0 | 1 | 1 | 3 |
| hsa-miR-125b-2-3p | MIMAT0004603 | ANXA8L1 | NM_001098845 | 0 | 1 | 0 | 1 | 1 | 3 |
| hsa-miR-125b-2-3p | MIMAT0004603 | CNTNAP3B | NM_001201380 | 1 | 1 | 0 | 0 | 1 | 3 |
| hsa-miR-125b-2-3p | MIMAT0004603 | PPIAL4F | NM_001164262 | 1 | 1 | 0 | 0 | 1 | 3 |
| hsa-miR-125b-2-3p | MIMAT0004603 | SFTPA2 | XM_005270123 | 0 | 1 | 0 | 1 | 1 | 3 |
| hsa-miR-125b-2-3p | MIMAT0004603 | FAM160A1 | XM_005263199 | 1 | 1 | 0 | 0 | 1 | 3 |
| hsa-miR-125b-2-3p | MIMAT0004603 | ISPD | NM_001101426 | 1 | 1 | 0 | 0 | 1 | 3 |
| hsa-miR-125b-2-3p | MIMAT0004603 | PPIAL4E | NM_001144032 | 1 | 1 | 0 | 0 | 1 | 3 |
| hsa-miR-125b-2-3p | MIMAT0004603 | ZNF737 | NM_001159293 | 0 | 1 | 1 | 0 | 1 | 3 |
| hsa-miR-125b-2-3p | MIMAT0004603 | ZNF316 | NM_001278559 | 1 | 1 | 0 | 0 | 1 | 3 |
| hsa-miR-125b-2-3p | MIMAT0004603 | ZNF705E | NM_001278713 | 1 | 1 | 0 | 0 | 1 | 3 |
| hsa-miR-125b-2-3p | MIMAT0004603 | TMEM178B | NM_001195278 | 1 | 1 | 0 | 0 | 1 | 3 |
| hsa-miR-125b-2-3p | MIMAT0004603 | RNF103-CHMP3 | NM_001198954 | 1 | 1 | 0 | 0 | 1 | 3 |
| hsa-miR-125b-2-3p | MIMAT0004603 | EPPIN-WFDC6 | NM_001198986 | 1 | 1 | 0 | 0 | 1 | 3 |
| hsa-miR-125b-2-3p | MIMAT0004603 | RPS10-NUDT3 | NM_001202470 | 1 | 1 | 0 | 0 | 1 | 3 |
| hsa-miR-125b-2-3p | MIMAT0004603 | LOC100996412 | XM_005275733 | 1 | 1 | 0 | 0 | 1 | 3 |
| hsa-miR-125b-2-3p | MIMAT0004603 | LOC100996725 | XM_003846490 | 1 | 1 | 0 | 0 | 1 | 3 |
| hsa-miR-125b-2-3p | MIMAT0004603 | LOC101927509 | XM_005247055 | 1 | 1 | 0 | 0 | 1 | 3 |
| hsa-miR-125b-2-3p | MIMAT0004603 | LOC101928218 | XM_005249509 | 1 | 1 | 0 | 0 | 1 | 3 |
| hsa-miR-125b-2-3p | MIMAT0004603 | LOC101928624 | XM_005275700 | 1 | 1 | 0 | 0 | 1 | 3 |
| hsa-miR-125b-2-3p | MIMAT0004603 | ADCY6 | NM_015270 | 1 | 0 | 0 | 1 | 1 | 3 |
| hsa-miR-125b-2-3p | MIMAT0004603 | ADCY7 | XM_005255783 | 1 | 0 | 0 | 1 | 1 | 3 |
| hsa-miR-125b-2-3p | MIMAT0004603 | ADH7 | NM_000673 | 1 | 0 | 0 | 1 | 1 | 3 |
| hsa-miR-125b-2-3p | MIMAT0004603 | ALDH3B2 | NM_000695 | 1 | 0 | 0 | 1 | 1 | 3 |
| hsa-miR-125b-2-3p | MIMAT0004603 | AMPD3 | NM_001025390 | 1 | 0 | 0 | 1 | 1 | 3 |
| hsa-miR-125b-2-3p | MIMAT0004603 | ANGPT2 | NM_001147 | 1 | 0 | 0 | 1 | 1 | 3 |
| hsa-miR-125b-2-3p | MIMAT0004603 | APLP2 | NM_001642 | 1 | 0 | 0 | 1 | 1 | 3 |
| hsa-miR-125b-2-3p | MIMAT0004603 | AQP2 | NM_000486 | 1 | 0 | 0 | 1 | 1 | 3 |
| hsa-miR-125b-2-3p | MIMAT0004603 | ARL4D | NM_001661 | 1 | 0 | 0 | 1 | 1 | 3 |
| hsa-miR-125b-2-3p | MIMAT0004603 | BAK1 | XM_005249253 | 1 | 0 | 0 | 1 | 1 | 3 |
| hsa-miR-125b-2-3p | MIMAT0004603 | DST | XM_005249310 | 1 | 0 | 0 | 1 | 1 | 3 |
| hsa-miR-125b-2-3p | MIMAT0004603 | TSPO | NM_001256530 | 1 | 0 | 0 | 1 | 1 | 3 |
| hsa-miR-125b-2-3p | MIMAT0004603 | CBL | NM_005188 | 1 | 0 | 0 | 1 | 1 | 3 |
| hsa-miR-125b-2-3p | MIMAT0004603 | KRIT1 | NM_194455 | 1 | 0 | 0 | 1 | 1 | 3 |
| hsa-miR-125b-2-3p | MIMAT0004603 | CD86 | XM_005247905 | 1 | 0 | 0 | 1 | 1 | 3 |
| hsa-miR-125b-2-3p | MIMAT0004603 | CDKN1A | NM_001220778 | 1 | 0 | 0 | 1 | 1 | 3 |
| hsa-miR-125b-2-3p | MIMAT0004603 | CHRNB2 | NM_000748 | 1 | 0 | 0 | 1 | 1 | 3 |
| hsa-miR-125b-2-3p | MIMAT0004603 | CLCN5 | NM_001127899 | 1 | 0 | 0 | 1 | 1 | 3 |
| hsa-miR-125b-2-3p | MIMAT0004603 | LTB4R | NM_181657 | 1 | 0 | 0 | 1 | 1 | 3 |
| hsa-miR-125b-2-3p | MIMAT0004603 | CNTF | NM_000614 | 1 | 0 | 0 | 1 | 1 | 3 |
| hsa-miR-125b-2-3p | MIMAT0004603 | COL8A2 | XM_005270477 | 1 | 0 | 0 | 1 | 1 | 3 |
| hsa-miR-125b-2-3p | MIMAT0004603 | CPN2 | NM_001080513 | 1 | 0 | 0 | 1 | 1 | 3 |
| hsa-miR-125b-2-3p | MIMAT0004603 | CRYAB | NM_001885 | 1 | 0 | 0 | 1 | 1 | 3 |
| hsa-miR-125b-2-3p | MIMAT0004603 | DHCR24 | NM_014762 | 1 | 0 | 0 | 1 | 1 | 3 |
| hsa-miR-125b-2-3p | MIMAT0004603 | DNA2 | NM_001080449 | 1 | 0 | 0 | 1 | 1 | 3 |
| hsa-miR-125b-2-3p | MIMAT0004603 | DUSP3 | XM_005257117 | 1 | 0 | 0 | 1 | 1 | 3 |
| hsa-miR-125b-2-3p | MIMAT0004603 | E2F2 | NM_004091 | 1 | 0 | 0 | 1 | 1 | 3 |
| hsa-miR-125b-2-3p | MIMAT0004603 | PHC1 | NM_004426 | 1 | 0 | 0 | 1 | 1 | 3 |
| hsa-miR-125b-2-3p | MIMAT0004603 | EIF2S1 | XM_005267390 | 1 | 0 | 0 | 1 | 1 | 3 |
| hsa-miR-125b-2-3p | MIMAT0004603 | STX2 | NM_194356 | 1 | 0 | 0 | 1 | 1 | 3 |
| hsa-miR-125b-2-3p | MIMAT0004603 | FANCF | NM_022725 | 1 | 0 | 0 | 1 | 1 | 3 |
| hsa-miR-125b-2-3p | MIMAT0004603 | FBN1 | NM_000138 | 1 | 0 | 0 | 1 | 1 | 3 |
| hsa-miR-125b-2-3p | MIMAT0004603 | FHL2 | NM_201555 | 1 | 0 | 0 | 1 | 1 | 3 |
| hsa-miR-125b-2-3p | MIMAT0004603 | FUT4 | NM_002033 | 1 | 0 | 0 | 1 | 1 | 3 |
| hsa-miR-125b-2-3p | MIMAT0004603 | GCK | NM_000162 | 1 | 0 | 0 | 1 | 1 | 3 |
| hsa-miR-125b-2-3p | MIMAT0004603 | GCNT2 | NM_001491 | 1 | 0 | 0 | 1 | 1 | 3 |
| hsa-miR-125b-2-3p | MIMAT0004603 | GJA3 | NM_021954 | 1 | 0 | 0 | 1 | 1 | 3 |
| hsa-miR-125b-2-3p | MIMAT0004603 | GJB1 | NM_000166 | 1 | 0 | 0 | 1 | 1 | 3 |
| hsa-miR-125b-2-3p | MIMAT0004603 | GLP1R | NM_002062 | 1 | 0 | 0 | 1 | 1 | 3 |
| hsa-miR-125b-2-3p | MIMAT0004603 | GNS | XM_005268800 | 1 | 0 | 0 | 1 | 1 | 3 |
| hsa-miR-125b-2-3p | MIMAT0004603 | GPR1 | NM_001261453 | 1 | 0 | 0 | 1 | 1 | 3 |
| hsa-miR-125b-2-3p | MIMAT0004603 | GPR17 | NM_001161415 | 1 | 0 | 0 | 1 | 1 | 3 |
| hsa-miR-125b-2-3p | MIMAT0004603 | GTF3C1 | NM_001520 | 1 | 0 | 0 | 1 | 1 | 3 |
| hsa-miR-125b-2-3p | MIMAT0004603 | HLA-B | XM_005249043 | 1 | 0 | 0 | 1 | 1 | 3 |
| hsa-miR-125b-2-3p | MIMAT0004603 | HMGB3 | XM_005274667 | 1 | 0 | 0 | 1 | 1 | 3 |
| hsa-miR-125b-2-3p | MIMAT0004603 | HOXA10 | NM_018951 | 1 | 0 | 0 | 1 | 1 | 3 |
| hsa-miR-125b-2-3p | MIMAT0004603 | ICA1 | XM_005249734 | 1 | 0 | 0 | 1 | 1 | 3 |
| hsa-miR-125b-2-3p | MIMAT0004603 | IGF1 | NM_001111283 | 1 | 0 | 0 | 1 | 1 | 3 |
| hsa-miR-125b-2-3p | MIMAT0004603 | IGFBP5 | NM_000599 | 1 | 0 | 0 | 1 | 1 | 3 |
| hsa-miR-125b-2-3p | MIMAT0004603 | ILF3 | XM_005259894 | 1 | 0 | 0 | 1 | 1 | 3 |
| hsa-miR-125b-2-3p | MIMAT0004603 | IRF5 | XM_005250317 | 1 | 0 | 0 | 1 | 1 | 3 |
| hsa-miR-125b-2-3p | MIMAT0004603 | JARID2 | NM_004973 | 1 | 0 | 0 | 1 | 1 | 3 |
| hsa-miR-125b-2-3p | MIMAT0004603 | KCNE1 | NM_000219 | 1 | 0 | 0 | 1 | 1 | 3 |
| hsa-miR-125b-2-3p | MIMAT0004603 | KLK2 | NM_001002231 | 1 | 0 | 0 | 1 | 1 | 3 |
| hsa-miR-125b-2-3p | MIMAT0004603 | KPNA4 | NM_002268 | 1 | 0 | 0 | 1 | 1 | 3 |
| hsa-miR-125b-2-3p | MIMAT0004603 | KRT85 | NM_002283 | 1 | 0 | 0 | 1 | 1 | 3 |
| hsa-miR-125b-2-3p | MIMAT0004603 | LGALS8 | NM_006499 | 1 | 0 | 0 | 1 | 1 | 3 |
| hsa-miR-125b-2-3p | MIMAT0004603 | LIF | NM_002309 | 1 | 0 | 0 | 1 | 1 | 3 |
| hsa-miR-125b-2-3p | MIMAT0004603 | LMX1B | NM_001174146 | 1 | 0 | 0 | 1 | 1 | 3 |
| hsa-miR-125b-2-3p | MIMAT0004603 | SMAD2 | NM_001003652 | 1 | 0 | 0 | 1 | 1 | 3 |
| hsa-miR-125b-2-3p | MIMAT0004603 | METTL1 | XM_005268873 | 1 | 0 | 0 | 1 | 1 | 3 |
| hsa-miR-125b-2-3p | MIMAT0004603 | MFNG | NM_002405 | 1 | 0 | 0 | 1 | 1 | 3 |
| hsa-miR-125b-2-3p | MIMAT0004603 | MGAT3 | NM_002409 | 1 | 0 | 0 | 1 | 1 | 3 |
| hsa-miR-125b-2-3p | MIMAT0004603 | MSN | XM_005262269 | 1 | 0 | 0 | 1 | 1 | 3 |
| hsa-miR-125b-2-3p | MIMAT0004603 | MYO9A | XM_005254404 | 1 | 0 | 0 | 1 | 1 | 3 |
| hsa-miR-125b-2-3p | MIMAT0004603 | NDUFA10 | NM_004544 | 1 | 0 | 0 | 1 | 1 | 3 |
| hsa-miR-125b-2-3p | MIMAT0004603 | NDUFC2 | NM_004549 | 1 | 0 | 0 | 1 | 1 | 3 |
| hsa-miR-125b-2-3p | MIMAT0004603 | NEDD9 | NM_001142393 | 1 | 0 | 0 | 1 | 1 | 3 |
| hsa-miR-125b-2-3p | MIMAT0004603 | NFIA | NM_001134673 | 1 | 0 | 0 | 1 | 1 | 3 |
| hsa-miR-125b-2-3p | MIMAT0004603 | NOTCH1 | NM_017617 | 1 | 0 | 0 | 1 | 1 | 3 |
| hsa-miR-125b-2-3p | MIMAT0004603 | NOTCH3 | NM_000435 | 1 | 0 | 0 | 1 | 1 | 3 |
| hsa-miR-125b-2-3p | MIMAT0004603 | P2RY1 | NM_002563 | 1 | 0 | 0 | 1 | 1 | 3 |
| hsa-miR-125b-2-3p | MIMAT0004603 | ENPP1 | NM_006208 | 1 | 0 | 0 | 1 | 1 | 3 |
| hsa-miR-125b-2-3p | MIMAT0004603 | PGR | NM_000926 | 1 | 0 | 0 | 1 | 1 | 3 |
| hsa-miR-125b-2-3p | MIMAT0004603 | PKNOX1 | NM_004571 | 1 | 0 | 0 | 1 | 1 | 3 |
| hsa-miR-125b-2-3p | MIMAT0004603 | POLE | NM_006231 | 1 | 0 | 0 | 1 | 1 | 3 |
| hsa-miR-125b-2-3p | MIMAT0004603 | POU2AF1 | XM_005271594 | 1 | 0 | 0 | 1 | 1 | 3 |
| hsa-miR-125b-2-3p | MIMAT0004603 | PPP1CB | NM_002709 | 1 | 0 | 0 | 1 | 1 | 3 |
| hsa-miR-125b-2-3p | MIMAT0004603 | PPP1R3A | NM_002711 | 1 | 0 | 0 | 1 | 1 | 3 |
| hsa-miR-125b-2-3p | MIMAT0004603 | PRKRIR | NM_004705 | 1 | 0 | 0 | 1 | 1 | 3 |
| hsa-miR-125b-2-3p | MIMAT0004603 | PSPH | XM_005271774 | 1 | 0 | 0 | 1 | 1 | 3 |
| hsa-miR-125b-2-3p | MIMAT0004603 | PTAFR | NM_001164723 | 1 | 0 | 0 | 1 | 1 | 3 |
| hsa-miR-125b-2-3p | MIMAT0004603 | PTCH1 | NM_001083602 | 1 | 0 | 0 | 1 | 1 | 3 |
| hsa-miR-125b-2-3p | MIMAT0004603 | PTPN11 | NM_002834 | 1 | 0 | 0 | 1 | 1 | 3 |
| hsa-miR-125b-2-3p | MIMAT0004603 | PEX5 | NM_001131025 | 1 | 0 | 0 | 1 | 1 | 3 |
| hsa-miR-125b-2-3p | MIMAT0004603 | PYGB | NM_002862 | 1 | 0 | 0 | 1 | 1 | 3 |
| hsa-miR-125b-2-3p | MIMAT0004603 | RAB27B | NM_004163 | 1 | 0 | 0 | 1 | 1 | 3 |
| hsa-miR-125b-2-3p | MIMAT0004603 | RAD52 | XM_005253720 | 1 | 0 | 0 | 1 | 1 | 3 |
| hsa-miR-125b-2-3p | MIMAT0004603 | RNASE2 | NM_002934 | 1 | 0 | 0 | 1 | 1 | 3 |
| hsa-miR-125b-2-3p | MIMAT0004603 | RNASE3 | NM_002935 | 1 | 0 | 0 | 1 | 1 | 3 |
| hsa-miR-125b-2-3p | MIMAT0004603 | RNASEL | NM_021133 | 1 | 0 | 0 | 1 | 1 | 3 |
| hsa-miR-125b-2-3p | MIMAT0004603 | MRPS12 | NM_033362 | 1 | 0 | 0 | 1 | 1 | 3 |
| hsa-miR-125b-2-3p | MIMAT0004603 | RPS23 | NM_001025 | 1 | 0 | 0 | 1 | 1 | 3 |
| hsa-miR-125b-2-3p | MIMAT0004603 | SALL2 | XM_005267983 | 1 | 0 | 0 | 1 | 1 | 3 |
| hsa-miR-125b-2-3p | MIMAT0004603 | SCN5A | NM_001099404 | 1 | 0 | 0 | 1 | 1 | 3 |
| hsa-miR-125b-2-3p | MIMAT0004603 | SFTPB | NM_000542 | 1 | 0 | 0 | 1 | 1 | 3 |
| hsa-miR-125b-2-3p | MIMAT0004603 | SIM2 | NM_005069 | 1 | 0 | 0 | 1 | 1 | 3 |
| hsa-miR-125b-2-3p | MIMAT0004603 | SLC6A4 | NM_001045 | 1 | 0 | 0 | 1 | 1 | 3 |
| hsa-miR-125b-2-3p | MIMAT0004603 | SOAT1 | NM_003101 | 1 | 0 | 0 | 1 | 1 | 3 |
| hsa-miR-125b-2-3p | MIMAT0004603 | TDGF1 | XM_005265418 | 1 | 0 | 0 | 1 | 1 | 3 |
| hsa-miR-125b-2-3p | MIMAT0004603 | TEP1 | NM_007110 | 1 | 0 | 0 | 1 | 1 | 3 |
| hsa-miR-125b-2-3p | MIMAT0004603 | TLE1 | NM_005077 | 1 | 0 | 0 | 1 | 1 | 3 |
| hsa-miR-125b-2-3p | MIMAT0004603 | TLL1 | XM_005263191 | 1 | 0 | 0 | 1 | 1 | 3 |
| hsa-miR-125b-2-3p | MIMAT0004603 | TLR5 | XM_005273242 | 1 | 0 | 0 | 1 | 1 | 3 |
| hsa-miR-125b-2-3p | MIMAT0004603 | TSPAN7 | XM_005272646 | 1 | 0 | 0 | 1 | 1 | 3 |
| hsa-miR-125b-2-3p | MIMAT0004603 | DNAJC7 | NM_003315 | 1 | 0 | 0 | 1 | 1 | 3 |
| hsa-miR-125b-2-3p | MIMAT0004603 | TNFSF4 | NM_003326 | 1 | 0 | 0 | 1 | 1 | 3 |
| hsa-miR-125b-2-3p | MIMAT0004603 | UBA52 | NM_001033930 | 1 | 0 | 0 | 1 | 1 | 3 |
| hsa-miR-125b-2-3p | MIMAT0004603 | UGP2 | XM_005264537 | 1 | 0 | 0 | 1 | 1 | 3 |
| hsa-miR-125b-2-3p | MIMAT0004603 | UGT2B4 | NM_021139 | 1 | 0 | 0 | 1 | 1 | 3 |
| hsa-miR-125b-2-3p | MIMAT0004603 | WNT11 | XM_005274231 | 1 | 0 | 0 | 1 | 1 | 3 |
| hsa-miR-125b-2-3p | MIMAT0004603 | ZNF16 | NM_001029976 | 1 | 0 | 0 | 1 | 1 | 3 |
| hsa-miR-125b-2-3p | MIMAT0004603 | ZNF37A | NM_001007094 | 1 | 0 | 0 | 1 | 1 | 3 |
| hsa-miR-125b-2-3p | MIMAT0004603 | ZNF148 | NM_021964 | 1 | 0 | 0 | 1 | 1 | 3 |
| hsa-miR-125b-2-3p | MIMAT0004603 | ZNF185 | XM_005274731 | 1 | 0 | 0 | 1 | 1 | 3 |
| hsa-miR-125b-2-3p | MIMAT0004603 | ZNF229 | NM_014518 | 1 | 0 | 0 | 1 | 1 | 3 |
| hsa-miR-125b-2-3p | MIMAT0004603 | ZXDA | NM_007156 | 1 | 0 | 0 | 1 | 1 | 3 |
| hsa-miR-125b-2-3p | MIMAT0004603 | USP7 | NM_003470 | 1 | 0 | 0 | 1 | 1 | 3 |
| hsa-miR-125b-2-3p | MIMAT0004603 | TKTL1 | NM_012253 | 1 | 0 | 0 | 1 | 1 | 3 |
| hsa-miR-125b-2-3p | MIMAT0004603 | AXIN2 | XM_005257717 | 1 | 0 | 0 | 1 | 1 | 3 |
| hsa-miR-125b-2-3p | MIMAT0004603 | MAD1L1 | NM_003550 | 1 | 0 | 0 | 1 | 1 | 3 |
| hsa-miR-125b-2-3p | MIMAT0004603 | PIP4K2B | NM_003559 | 1 | 0 | 0 | 1 | 1 | 3 |
| hsa-miR-125b-2-3p | MIMAT0004603 | STK24 | NM_001032296 | 1 | 0 | 0 | 1 | 1 | 3 |
| hsa-miR-125b-2-3p | MIMAT0004603 | GAS7 | NM_201433 | 1 | 0 | 0 | 1 | 1 | 3 |
| hsa-miR-125b-2-3p | MIMAT0004603 | TNFRSF14 | XM_005244813 | 1 | 0 | 0 | 1 | 1 | 3 |
| hsa-miR-125b-2-3p | MIMAT0004603 | MTMR1 | XM_005274765 | 1 | 0 | 0 | 1 | 1 | 3 |
| hsa-miR-125b-2-3p | MIMAT0004603 | TNFRSF10C | NM_003841 | 1 | 0 | 0 | 1 | 1 | 3 |
| hsa-miR-125b-2-3p | MIMAT0004603 | MTMR2 | NM_001243571 | 1 | 0 | 0 | 1 | 1 | 3 |
| hsa-miR-125b-2-3p | MIMAT0004603 | HAP1 | NM_177977 | 1 | 0 | 0 | 1 | 1 | 3 |
| hsa-miR-125b-2-3p | MIMAT0004603 | RABEP1 | NM_004703 | 1 | 0 | 0 | 1 | 1 | 3 |
| hsa-miR-125b-2-3p | MIMAT0004603 | SLC24A1 | NM_004727 | 1 | 0 | 0 | 1 | 1 | 3 |
| hsa-miR-125b-2-3p | MIMAT0004603 | VAPB | NM_004738 | 1 | 0 | 0 | 1 | 1 | 3 |
| hsa-miR-125b-2-3p | MIMAT0004603 | TRIP13 | XM_005248388 | 1 | 0 | 0 | 1 | 1 | 3 |
| hsa-miR-125b-2-3p | MIMAT0004603 | ADIPOQ | NM_001177800 | 1 | 0 | 0 | 1 | 1 | 3 |
| hsa-miR-125b-2-3p | MIMAT0004603 | CD101 | NM_004258 | 1 | 0 | 0 | 1 | 1 | 3 |
| hsa-miR-125b-2-3p | MIMAT0004603 | QKI | NM_206855 | 1 | 0 | 0 | 1 | 1 | 3 |
| hsa-miR-125b-2-3p | MIMAT0004603 | PIGL | NM_004278 | 1 | 0 | 0 | 1 | 1 | 3 |
| hsa-miR-125b-2-3p | MIMAT0004603 | ENTPD4 | NM_004901 | 1 | 0 | 0 | 1 | 1 | 3 |
| hsa-miR-125b-2-3p | MIMAT0004603 | PRDX6 | NM_004905 | 1 | 0 | 0 | 1 | 1 | 3 |
| hsa-miR-125b-2-3p | MIMAT0004603 | IKBKE | NM_014002 | 1 | 0 | 0 | 1 | 1 | 3 |
| hsa-miR-125b-2-3p | MIMAT0004603 | PPM1F | NM_014634 | 1 | 0 | 0 | 1 | 1 | 3 |
| hsa-miR-125b-2-3p | MIMAT0004603 | CEP104 | NM_014704 | 1 | 0 | 0 | 1 | 1 | 3 |
| hsa-miR-125b-2-3p | MIMAT0004603 | HDAC9 | XM_005249913 | 1 | 0 | 0 | 1 | 1 | 3 |
| hsa-miR-125b-2-3p | MIMAT0004603 | PCDHA9 | NM_014005 | 1 | 0 | 0 | 1 | 1 | 3 |
| hsa-miR-125b-2-3p | MIMAT0004603 | STARD8 | NM_001142503 | 1 | 0 | 0 | 1 | 1 | 3 |
| hsa-miR-125b-2-3p | MIMAT0004603 | TRIM66 | XM_005253270 | 1 | 0 | 0 | 1 | 1 | 3 |
| hsa-miR-125b-2-3p | MIMAT0004603 | ZBTB40 | NM_001083621 | 1 | 0 | 0 | 1 | 1 | 3 |
| hsa-miR-125b-2-3p | MIMAT0004603 | SLC17A4 | NM_005495 | 1 | 0 | 0 | 1 | 1 | 3 |
| hsa-miR-125b-2-3p | MIMAT0004603 | SUGP2 | XM_005259704 | 1 | 0 | 0 | 1 | 1 | 3 |
| hsa-miR-125b-2-3p | MIMAT0004603 | RBM5 | NM_005778 | 1 | 0 | 0 | 1 | 1 | 3 |
| hsa-miR-125b-2-3p | MIMAT0004603 | TRIM13 | NM_213590 | 1 | 0 | 0 | 1 | 1 | 3 |
| hsa-miR-125b-2-3p | MIMAT0004603 | TRIB1 | NM_025195 | 1 | 0 | 0 | 1 | 1 | 3 |
| hsa-miR-125b-2-3p | MIMAT0004603 | CRISP3 | NM_001190986 | 1 | 0 | 0 | 1 | 1 | 3 |
| hsa-miR-125b-2-3p | MIMAT0004603 | DLC1 | NM_182643 | 1 | 0 | 0 | 1 | 1 | 3 |
| hsa-miR-125b-2-3p | MIMAT0004603 | RAPGEF3 | NM_001098531 | 1 | 0 | 0 | 1 | 1 | 3 |
| hsa-miR-125b-2-3p | MIMAT0004603 | CD2BP2 | NM_001243646 | 1 | 0 | 0 | 1 | 1 | 3 |
| hsa-miR-125b-2-3p | MIMAT0004603 | PLAC1 | NM_021796 | 1 | 0 | 0 | 1 | 1 | 3 |
| hsa-miR-125b-2-3p | MIMAT0004603 | NUP50 | XM_005261312 | 1 | 0 | 0 | 1 | 1 | 3 |
| hsa-miR-125b-2-3p | MIMAT0004603 | ZNF274 | NM_001278734 | 1 | 0 | 0 | 1 | 1 | 3 |
| hsa-miR-125b-2-3p | MIMAT0004603 | TSPAN9 | NM_006675 | 1 | 0 | 0 | 1 | 1 | 3 |
| hsa-miR-125b-2-3p | MIMAT0004603 | PAPOLA | NM_001252007 | 1 | 0 | 0 | 1 | 1 | 3 |
| hsa-miR-125b-2-3p | MIMAT0004603 | CKAP4 | NM_006825 | 1 | 0 | 0 | 1 | 1 | 3 |
| hsa-miR-125b-2-3p | MIMAT0004603 | ILVBL | XM_005259717 | 1 | 0 | 0 | 1 | 1 | 3 |
| hsa-miR-125b-2-3p | MIMAT0004603 | KDELR3 | NM_006855 | 1 | 0 | 0 | 1 | 1 | 3 |
| hsa-miR-125b-2-3p | MIMAT0004603 | GTF2A1L | NM_006872 | 1 | 0 | 0 | 1 | 1 | 3 |
| hsa-miR-125b-2-3p | MIMAT0004603 | STON1 | NM_001198595 | 1 | 0 | 0 | 1 | 1 | 3 |
| hsa-miR-125b-2-3p | MIMAT0004603 | WDR5 | XM_005272163 | 1 | 0 | 0 | 1 | 1 | 3 |
| hsa-miR-125b-2-3p | MIMAT0004603 | HHLA3 | NM_001036645 | 1 | 0 | 0 | 1 | 1 | 3 |
| hsa-miR-125b-2-3p | MIMAT0004603 | BAZ2A | NM_013449 | 1 | 0 | 0 | 1 | 1 | 3 |
| hsa-miR-125b-2-3p | MIMAT0004603 | CA5B | NM_007220 | 1 | 0 | 0 | 1 | 1 | 3 |
| hsa-miR-125b-2-3p | MIMAT0004603 | GPN1 | NM_007266 | 1 | 0 | 0 | 1 | 1 | 3 |
| hsa-miR-125b-2-3p | MIMAT0004603 | SMC5 | NM_015110 | 1 | 0 | 0 | 1 | 1 | 3 |
| hsa-miR-125b-2-3p | MIMAT0004603 | N4BP3 | NM_015111 | 1 | 0 | 0 | 1 | 1 | 3 |
| hsa-miR-125b-2-3p | MIMAT0004603 | ZCCHC14 | NM_015144 | 1 | 0 | 0 | 1 | 1 | 3 |
| hsa-miR-125b-2-3p | MIMAT0004603 | RCOR1 | NM_015156 | 1 | 0 | 0 | 1 | 1 | 3 |
| hsa-miR-125b-2-3p | MIMAT0004603 | UBXN4 | NM_014607 | 1 | 0 | 0 | 1 | 1 | 3 |
| hsa-miR-125b-2-3p | MIMAT0004603 | OTUD3 | NM_015207 | 1 | 0 | 0 | 1 | 1 | 3 |
| hsa-miR-125b-2-3p | MIMAT0004603 | IQCE | NM_152558 | 1 | 0 | 0 | 1 | 1 | 3 |
| hsa-miR-125b-2-3p | MIMAT0004603 | TMEM194A | NM_001130963 | 1 | 0 | 0 | 1 | 1 | 3 |
| hsa-miR-125b-2-3p | MIMAT0004603 | USP22 | NM_015276 | 1 | 0 | 0 | 1 | 1 | 3 |
| hsa-miR-125b-2-3p | MIMAT0004603 | SPECC1L | NM_015330 | 1 | 0 | 0 | 1 | 1 | 3 |
| hsa-miR-125b-2-3p | MIMAT0004603 | ATP1B4 | NM_001142447 | 1 | 0 | 0 | 1 | 1 | 3 |
| hsa-miR-125b-2-3p | MIMAT0004603 | NPTXR | NM_014293 | 1 | 0 | 0 | 1 | 1 | 3 |
| hsa-miR-125b-2-3p | MIMAT0004603 | CBX5 | NM_001127322 | 1 | 0 | 0 | 1 | 1 | 3 |
| hsa-miR-125b-2-3p | MIMAT0004603 | LEPROTL1 | NM_015344 | 1 | 0 | 0 | 1 | 1 | 3 |
| hsa-miR-125b-2-3p | MIMAT0004603 | AMACR | NM_014324 | 1 | 0 | 0 | 1 | 1 | 3 |
| hsa-miR-125b-2-3p | MIMAT0004603 | NUP62 | NM_016553 | 1 | 0 | 0 | 1 | 1 | 3 |
| hsa-miR-125b-2-3p | MIMAT0004603 | RABGAP1 | NM_012197 | 1 | 0 | 0 | 1 | 1 | 3 |
| hsa-miR-125b-2-3p | MIMAT0004603 | SHPK | NM_013276 | 1 | 0 | 0 | 1 | 1 | 3 |
| hsa-miR-125b-2-3p | MIMAT0004603 | METTL21B | NM_206914 | 1 | 0 | 0 | 1 | 1 | 3 |
| hsa-miR-125b-2-3p | MIMAT0004603 | TIPARP | NM_001184717 | 1 | 0 | 0 | 1 | 1 | 3 |
| hsa-miR-125b-2-3p | MIMAT0004603 | GIGYF2 | NM_015575 | 1 | 0 | 0 | 1 | 1 | 3 |
| hsa-miR-125b-2-3p | MIMAT0004603 | PTPN18 | NM_014369 | 1 | 0 | 0 | 1 | 1 | 3 |
| hsa-miR-125b-2-3p | MIMAT0004603 | TIMM10B | NM_012192 | 1 | 0 | 0 | 1 | 1 | 3 |
| hsa-miR-125b-2-3p | MIMAT0004603 | SH3YL1 | NM_015677 | 1 | 0 | 0 | 1 | 1 | 3 |
| hsa-miR-125b-2-3p | MIMAT0004603 | TAF5L | NM_001025247 | 1 | 0 | 0 | 1 | 1 | 3 |
| hsa-miR-125b-2-3p | MIMAT0004603 | AFF4 | XM_005271963 | 1 | 0 | 0 | 1 | 1 | 3 |
| hsa-miR-125b-2-3p | MIMAT0004603 | ZNF544 | XM_005258756 | 1 | 0 | 0 | 1 | 1 | 3 |
| hsa-miR-125b-2-3p | MIMAT0004603 | KCNMB4 | NM_014505 | 1 | 0 | 0 | 1 | 1 | 3 |
| hsa-miR-125b-2-3p | MIMAT0004603 | OLA1 | NM_013341 | 1 | 0 | 0 | 1 | 1 | 3 |
| hsa-miR-125b-2-3p | MIMAT0004603 | UBN1 | XM_005255281 | 1 | 0 | 0 | 1 | 1 | 3 |
| hsa-miR-125b-2-3p | MIMAT0004603 | GPSM2 | XM_005270787 | 1 | 0 | 0 | 1 | 1 | 3 |
| hsa-miR-125b-2-3p | MIMAT0004603 | PYCR2 | XM_005273116 | 1 | 0 | 0 | 1 | 1 | 3 |
| hsa-miR-125b-2-3p | MIMAT0004603 | RAX | NM_013435 | 1 | 0 | 0 | 1 | 1 | 3 |
| hsa-miR-125b-2-3p | MIMAT0004603 | RRP15 | NM_016052 | 1 | 0 | 0 | 1 | 1 | 3 |
| hsa-miR-125b-2-3p | MIMAT0004603 | MRPS16 | NM_016065 | 1 | 0 | 0 | 1 | 1 | 3 |
| hsa-miR-125b-2-3p | MIMAT0004603 | WDPCP | NM_015910 | 1 | 0 | 0 | 1 | 1 | 3 |
| hsa-miR-125b-2-3p | MIMAT0004603 | BET1L | NM_016526 | 1 | 0 | 0 | 1 | 1 | 3 |
| hsa-miR-125b-2-3p | MIMAT0004603 | TMEM138 | NM_016464 | 1 | 0 | 0 | 1 | 1 | 3 |
| hsa-miR-125b-2-3p | MIMAT0004603 | FKBP7 | NM_181342 | 1 | 0 | 0 | 1 | 1 | 3 |
| hsa-miR-125b-2-3p | MIMAT0004603 | SUFU | NM_016169 | 1 | 0 | 0 | 1 | 1 | 3 |
| hsa-miR-125b-2-3p | MIMAT0004603 | PADI3 | NM_016233 | 1 | 0 | 0 | 1 | 1 | 3 |
| hsa-miR-125b-2-3p | MIMAT0004603 | CPSF2 | NM_017437 | 1 | 0 | 0 | 1 | 1 | 3 |
| hsa-miR-125b-2-3p | MIMAT0004603 | SSH1 | XM_005268984 | 1 | 0 | 0 | 1 | 1 | 3 |
| hsa-miR-125b-2-3p | MIMAT0004603 | TOLLIP | XM_005252994 | 1 | 0 | 0 | 1 | 1 | 3 |
| hsa-miR-125b-2-3p | MIMAT0004603 | TMX3 | NM_019022 | 1 | 0 | 0 | 1 | 1 | 3 |
| hsa-miR-125b-2-3p | MIMAT0004603 | NECAB2 | NM_019065 | 1 | 0 | 0 | 1 | 1 | 3 |
| hsa-miR-125b-2-3p | MIMAT0004603 | WDR5B | NM_019069 | 1 | 0 | 0 | 1 | 1 | 3 |
| hsa-miR-125b-2-3p | MIMAT0004603 | ZNHIT6 | NM_017953 | 1 | 0 | 0 | 1 | 1 | 3 |
| hsa-miR-125b-2-3p | MIMAT0004603 | XAF1 | XM_005256705 | 1 | 0 | 0 | 1 | 1 | 3 |
| hsa-miR-125b-2-3p | MIMAT0004603 | ZNF562 | NM_001130031 | 1 | 0 | 0 | 1 | 1 | 3 |
| hsa-miR-125b-2-3p | MIMAT0004603 | IMPAD1 | NM_017813 | 1 | 0 | 0 | 1 | 1 | 3 |
| hsa-miR-125b-2-3p | MIMAT0004603 | ZNF770 | NM_014106 | 1 | 0 | 0 | 1 | 1 | 3 |
| hsa-miR-125b-2-3p | MIMAT0004603 | UBR7 | NM_175748 | 1 | 0 | 0 | 1 | 1 | 3 |
| hsa-miR-125b-2-3p | MIMAT0004603 | GPALPP1 | XM_005266443 | 1 | 0 | 0 | 1 | 1 | 3 |
| hsa-miR-125b-2-3p | MIMAT0004603 | SMPD3 | XM_005256031 | 1 | 0 | 0 | 1 | 1 | 3 |
| hsa-miR-125b-2-3p | MIMAT0004603 | SLC48A1 | NM_017842 | 1 | 0 | 0 | 1 | 1 | 3 |
| hsa-miR-125b-2-3p | MIMAT0004603 | NPLOC4 | NM_017921 | 1 | 0 | 0 | 1 | 1 | 3 |
| hsa-miR-125b-2-3p | MIMAT0004603 | PCDHB4 | NM_018938 | 1 | 0 | 0 | 1 | 1 | 3 |
| hsa-miR-125b-2-3p | MIMAT0004603 | MTFR1L | NM_019557 | 1 | 0 | 0 | 1 | 1 | 3 |
| hsa-miR-125b-2-3p | MIMAT0004603 | LRRC8A | XM_005252094 | 1 | 0 | 0 | 1 | 1 | 3 |
| hsa-miR-125b-2-3p | MIMAT0004603 | DHX33 | NM_020162 | 1 | 0 | 0 | 1 | 1 | 3 |
| hsa-miR-125b-2-3p | MIMAT0004603 | KIAA1199 | XM_005254567 | 1 | 0 | 0 | 1 | 1 | 3 |
| hsa-miR-125b-2-3p | MIMAT0004603 | KIAA1244 | NM_020340 | 1 | 0 | 0 | 1 | 1 | 3 |
| hsa-miR-125b-2-3p | MIMAT0004603 | RAB22A | NM_020673 | 1 | 0 | 0 | 1 | 1 | 3 |
| hsa-miR-125b-2-3p | MIMAT0004603 | PPM1H | NM_020700 | 1 | 0 | 0 | 1 | 1 | 3 |
| hsa-miR-125b-2-3p | MIMAT0004603 | KIAA1377 | NM_020802 | 1 | 0 | 0 | 1 | 1 | 3 |
| hsa-miR-125b-2-3p | MIMAT0004603 | TRMT5 | XM_005267916 | 1 | 0 | 0 | 1 | 1 | 3 |
| hsa-miR-125b-2-3p | MIMAT0004603 | TMEM181 | NM_020823 | 1 | 0 | 0 | 1 | 1 | 3 |
| hsa-miR-125b-2-3p | MIMAT0004603 | SYT13 | NM_001247987 | 1 | 0 | 0 | 1 | 1 | 3 |
| hsa-miR-125b-2-3p | MIMAT0004603 | EP400 | XM_005253587 | 1 | 0 | 0 | 1 | 1 | 3 |
| hsa-miR-125b-2-3p | MIMAT0004603 | ZNF317 | NM_020933 | 1 | 0 | 0 | 1 | 1 | 3 |
| hsa-miR-125b-2-3p | MIMAT0004603 | ZNF529 | NM_001145649 | 1 | 0 | 0 | 1 | 1 | 3 |
| hsa-miR-125b-2-3p | MIMAT0004603 | ENOPH1 | NM_021204 | 1 | 0 | 0 | 1 | 1 | 3 |
| hsa-miR-125b-2-3p | MIMAT0004603 | XPNPEP3 | NM_022098 | 1 | 0 | 0 | 1 | 1 | 3 |
| hsa-miR-125b-2-3p | MIMAT0004603 | MOAP1 | NM_022151 | 1 | 0 | 0 | 1 | 1 | 3 |
| hsa-miR-125b-2-3p | MIMAT0004603 | TMBIM1 | XM_005246760 | 1 | 0 | 0 | 1 | 1 | 3 |
| hsa-miR-125b-2-3p | MIMAT0004603 | ZFYVE20 | NM_022340 | 1 | 0 | 0 | 1 | 1 | 3 |
| hsa-miR-125b-2-3p | MIMAT0004603 | COPS7B | NM_022730 | 1 | 0 | 0 | 1 | 1 | 3 |
| hsa-miR-125b-2-3p | MIMAT0004603 | C16orf58 | NM_022744 | 1 | 0 | 0 | 1 | 1 | 3 |
| hsa-miR-125b-2-3p | MIMAT0004603 | GNPNAT1 | NM_198066 | 1 | 0 | 0 | 1 | 1 | 3 |
| hsa-miR-125b-2-3p | MIMAT0004603 | VWA1 | NM_022834 | 1 | 0 | 0 | 1 | 1 | 3 |
| hsa-miR-125b-2-3p | MIMAT0004603 | WNK3 | NM_020922 | 1 | 0 | 0 | 1 | 1 | 3 |
| hsa-miR-125b-2-3p | MIMAT0004603 | FA2H | NM_024306 | 1 | 0 | 0 | 1 | 1 | 3 |
| hsa-miR-125b-2-3p | MIMAT0004603 | ZNF557 | NM_024341 | 1 | 0 | 0 | 1 | 1 | 3 |
| hsa-miR-125b-2-3p | MIMAT0004603 | TMEM231 | NM_001077416 | 1 | 0 | 0 | 1 | 1 | 3 |
| hsa-miR-125b-2-3p | MIMAT0004603 | NARG2 | NM_024611 | 1 | 0 | 0 | 1 | 1 | 3 |
| hsa-miR-125b-2-3p | MIMAT0004603 | GTDC1 | XM_005263783 | 1 | 0 | 0 | 1 | 1 | 3 |
| hsa-miR-125b-2-3p | MIMAT0004603 | TTC21B | NM_024753 | 1 | 0 | 0 | 1 | 1 | 3 |
| hsa-miR-125b-2-3p | MIMAT0004603 | FAM124B | NM_024785 | 1 | 0 | 0 | 1 | 1 | 3 |
| hsa-miR-125b-2-3p | MIMAT0004603 | ZNF671 | NM_024833 | 1 | 0 | 0 | 1 | 1 | 3 |
| hsa-miR-125b-2-3p | MIMAT0004603 | CCDC15 | NM_025004 | 1 | 0 | 0 | 1 | 1 | 3 |
| hsa-miR-125b-2-3p | MIMAT0004603 | AKNA | NM_030767 | 1 | 0 | 0 | 1 | 1 | 3 |
| hsa-miR-125b-2-3p | MIMAT0004603 | BHLHB9 | NM_001142524 | 1 | 0 | 0 | 1 | 1 | 3 |
| hsa-miR-125b-2-3p | MIMAT0004603 | TXNDC5 | NM_030810 | 1 | 0 | 0 | 1 | 1 | 3 |
| hsa-miR-125b-2-3p | MIMAT0004603 | CCDC130 | XM_005260085 | 1 | 0 | 0 | 1 | 1 | 3 |
| hsa-miR-125b-2-3p | MIMAT0004603 | ZNF93 | NM_031218 | 1 | 0 | 0 | 1 | 1 | 3 |
| hsa-miR-125b-2-3p | MIMAT0004603 | TM2D2 | NM_031940 | 1 | 0 | 0 | 1 | 1 | 3 |
| hsa-miR-125b-2-3p | MIMAT0004603 | KATNAL1 | XM_005266574 | 1 | 0 | 0 | 1 | 1 | 3 |
| hsa-miR-125b-2-3p | MIMAT0004603 | MND1 | XM_005263274 | 1 | 0 | 0 | 1 | 1 | 3 |
| hsa-miR-125b-2-3p | MIMAT0004603 | RBM48 | XM_005250636 | 1 | 0 | 0 | 1 | 1 | 3 |
| hsa-miR-125b-2-3p | MIMAT0004603 | ARID5B | NM_032199 | 1 | 0 | 0 | 1 | 1 | 3 |
| hsa-miR-125b-2-3p | MIMAT0004603 | SRRM4 | NM_194286 | 1 | 0 | 0 | 1 | 1 | 3 |
| hsa-miR-125b-2-3p | MIMAT0004603 | KBTBD8 | NM_032505 | 1 | 0 | 0 | 1 | 1 | 3 |
| hsa-miR-125b-2-3p | MIMAT0004603 | ST6GAL2 | NM_032528 | 1 | 0 | 0 | 1 | 1 | 3 |
| hsa-miR-125b-2-3p | MIMAT0004603 | SLC35B4 | NM_032826 | 1 | 0 | 0 | 1 | 1 | 3 |
| hsa-miR-125b-2-3p | MIMAT0004603 | LMLN | NM_001136049 | 1 | 0 | 0 | 1 | 1 | 3 |
| hsa-miR-125b-2-3p | MIMAT0004603 | FAM105B | NM_138348 | 1 | 0 | 0 | 1 | 1 | 3 |
| hsa-miR-125b-2-3p | MIMAT0004603 | MCFD2 | NM_001171508 | 1 | 0 | 0 | 1 | 1 | 3 |
| hsa-miR-125b-2-3p | MIMAT0004603 | HN1L | NM_144570 | 1 | 0 | 0 | 1 | 1 | 3 |
| hsa-miR-125b-2-3p | MIMAT0004603 | UAP1L1 | XM_005266118 | 1 | 0 | 0 | 1 | 1 | 3 |
| hsa-miR-125b-2-3p | MIMAT0004603 | RFT1 | NM_052859 | 1 | 0 | 0 | 1 | 1 | 3 |
| hsa-miR-125b-2-3p | MIMAT0004603 | SPSB4 | NM_080862 | 1 | 0 | 0 | 1 | 1 | 3 |
| hsa-miR-125b-2-3p | MIMAT0004603 | VPS26B | NM_052875 | 1 | 0 | 0 | 1 | 1 | 3 |
| hsa-miR-125b-2-3p | MIMAT0004603 | GLB1L3 | NM_001080407 | 1 | 0 | 0 | 1 | 1 | 3 |
| hsa-miR-125b-2-3p | MIMAT0004603 | CYP2U1 | XM_005262717 | 1 | 0 | 0 | 1 | 1 | 3 |
| hsa-miR-125b-2-3p | MIMAT0004603 | PIK3IP1 | XM_005261323 | 1 | 0 | 0 | 1 | 1 | 3 |
| hsa-miR-125b-2-3p | MIMAT0004603 | ERMAP | NM_001017922 | 1 | 0 | 0 | 1 | 1 | 3 |
| hsa-miR-125b-2-3p | MIMAT0004603 | GPRIN1 | NM_052899 | 1 | 0 | 0 | 1 | 1 | 3 |
| hsa-miR-125b-2-3p | MIMAT0004603 | ZNF526 | NM_133444 | 1 | 0 | 0 | 1 | 1 | 3 |
| hsa-miR-125b-2-3p | MIMAT0004603 | RAB39B | NM_171998 | 1 | 0 | 0 | 1 | 1 | 3 |
| hsa-miR-125b-2-3p | MIMAT0004603 | SH2D1B | NM_053282 | 1 | 0 | 0 | 1 | 1 | 3 |
| hsa-miR-125b-2-3p | MIMAT0004603 | MRGPRX2 | NM_054030 | 1 | 0 | 0 | 1 | 1 | 3 |
| hsa-miR-125b-2-3p | MIMAT0004603 | PIH1D2 | NM_001082619 | 1 | 0 | 0 | 1 | 1 | 3 |
| hsa-miR-125b-2-3p | MIMAT0004603 | SPATA33 | NM_001271909 | 1 | 0 | 0 | 1 | 1 | 3 |
| hsa-miR-125b-2-3p | MIMAT0004603 | AFMID | NM_001145526 | 1 | 0 | 0 | 1 | 1 | 3 |
| hsa-miR-125b-2-3p | MIMAT0004603 | MOB3A | XM_005259489 | 1 | 0 | 0 | 1 | 1 | 3 |
| hsa-miR-125b-2-3p | MIMAT0004603 | ZNF648 | NM_001009992 | 1 | 0 | 0 | 1 | 1 | 3 |
| hsa-miR-125b-2-3p | MIMAT0004603 | FBLN7 | NM_153214 | 1 | 0 | 0 | 1 | 1 | 3 |
| hsa-miR-125b-2-3p | MIMAT0004603 | MTERFD2 | NM_182501 | 1 | 0 | 0 | 1 | 1 | 3 |
| hsa-miR-125b-2-3p | MIMAT0004603 | LYZL4 | XM_005264865 | 1 | 0 | 0 | 1 | 1 | 3 |
| hsa-miR-125b-2-3p | MIMAT0004603 | UROC1 | NM_001165974 | 1 | 0 | 0 | 1 | 1 | 3 |
| hsa-miR-125b-2-3p | MIMAT0004603 | NUDT16 | NM_152395 | 1 | 0 | 0 | 1 | 1 | 3 |
| hsa-miR-125b-2-3p | MIMAT0004603 | C4orf33 | XM_005262734 | 1 | 0 | 0 | 1 | 1 | 3 |
| hsa-miR-125b-2-3p | MIMAT0004603 | NADK2 | NM_001085411 | 1 | 0 | 0 | 1 | 1 | 3 |
| hsa-miR-125b-2-3p | MIMAT0004603 | TRIM40 | XM_005248855 | 1 | 0 | 0 | 1 | 1 | 3 |
| hsa-miR-125b-2-3p | MIMAT0004603 | SRCRB4D | NM_080744 | 1 | 0 | 0 | 1 | 1 | 3 |
| hsa-miR-125b-2-3p | MIMAT0004603 | ISM1 | NM_080826 | 1 | 0 | 0 | 1 | 1 | 3 |
| hsa-miR-125b-2-3p | MIMAT0004603 | EIF5AL1 | NM_001099692 | 1 | 0 | 0 | 1 | 1 | 3 |
| hsa-miR-125b-2-3p | MIMAT0004603 | TMEM120B | NM_001080825 | 1 | 0 | 0 | 1 | 1 | 3 |
| hsa-miR-125b-2-3p | MIMAT0004603 | CEP128 | NM_152446 | 1 | 0 | 0 | 1 | 1 | 3 |
| hsa-miR-125b-2-3p | MIMAT0004603 | TOM1L2 | XM_005256461 | 1 | 0 | 0 | 1 | 1 | 3 |
| hsa-miR-125b-2-3p | MIMAT0004603 | TMC8 | NM_152468 | 1 | 0 | 0 | 1 | 1 | 3 |
| hsa-miR-125b-2-3p | MIMAT0004603 | ZNF418 | NM_133460 | 1 | 0 | 0 | 1 | 1 | 3 |
| hsa-miR-125b-2-3p | MIMAT0004603 | CLDN19 | NM_001123395 | 1 | 0 | 0 | 1 | 1 | 3 |
| hsa-miR-125b-2-3p | MIMAT0004603 | BTLA | NM_181780 | 1 | 0 | 0 | 1 | 1 | 3 |
| hsa-miR-125b-2-3p | MIMAT0004603 | FGD5 | XM_005264900 | 1 | 0 | 0 | 1 | 1 | 3 |
| hsa-miR-125b-2-3p | MIMAT0004603 | DAB2IP | XM_005251721 | 1 | 0 | 0 | 1 | 1 | 3 |
| hsa-miR-125b-2-3p | MIMAT0004603 | ABCA13 | NM_152701 | 1 | 0 | 0 | 1 | 1 | 3 |
| hsa-miR-125b-2-3p | MIMAT0004603 | AMZ1 | NM_133463 | 1 | 0 | 0 | 1 | 1 | 3 |
| hsa-miR-125b-2-3p | MIMAT0004603 | TTC39B | NM_152574 | 1 | 0 | 0 | 1 | 1 | 3 |
| hsa-miR-125b-2-3p | MIMAT0004603 | FAM134C | NM_178126 | 1 | 0 | 0 | 1 | 1 | 3 |
| hsa-miR-125b-2-3p | MIMAT0004603 | OR7D2 | NM_175883 | 1 | 0 | 0 | 1 | 1 | 3 |
| hsa-miR-125b-2-3p | MIMAT0004603 | DQX1 | NM_133637 | 1 | 0 | 0 | 1 | 1 | 3 |
| hsa-miR-125b-2-3p | MIMAT0004603 | RNF168 | NM_152617 | 1 | 0 | 0 | 1 | 1 | 3 |
| hsa-miR-125b-2-3p | MIMAT0004603 | GIMAP1 | NM_130759 | 1 | 0 | 0 | 1 | 1 | 3 |
| hsa-miR-125b-2-3p | MIMAT0004603 | C8orf74 | NM_001040032 | 1 | 0 | 0 | 1 | 1 | 3 |
| hsa-miR-125b-2-3p | MIMAT0004603 | NRK | NM_198465 | 1 | 0 | 0 | 1 | 1 | 3 |
| hsa-miR-125b-2-3p | MIMAT0004603 | UNC5B | NM_170744 | 1 | 0 | 0 | 1 | 1 | 3 |
| hsa-miR-125b-2-3p | MIMAT0004603 | BEND7 | NM_152751 | 1 | 0 | 0 | 1 | 1 | 3 |
| hsa-miR-125b-2-3p | MIMAT0004603 | LNX2 | NM_153371 | 1 | 0 | 0 | 1 | 1 | 3 |
| hsa-miR-125b-2-3p | MIMAT0004603 | LCE5A | NM_178438 | 1 | 0 | 0 | 1 | 1 | 3 |
| hsa-miR-125b-2-3p | MIMAT0004603 | SHPRH | NM_001042683 | 1 | 0 | 0 | 1 | 1 | 3 |
| hsa-miR-125b-2-3p | MIMAT0004603 | ANKRD52 | NM_173595 | 1 | 0 | 0 | 1 | 1 | 3 |
| hsa-miR-125b-2-3p | MIMAT0004603 | CES4A | XM_005255895 | 1 | 0 | 0 | 1 | 1 | 3 |
| hsa-miR-125b-2-3p | MIMAT0004603 | ZNF283 | XM_005258784 | 1 | 0 | 0 | 1 | 1 | 3 |
| hsa-miR-125b-2-3p | MIMAT0004603 | SH3PXD2B | NM_001017995 | 1 | 0 | 0 | 1 | 1 | 3 |
| hsa-miR-125b-2-3p | MIMAT0004603 | FAM78A | NM_033387 | 1 | 0 | 0 | 1 | 1 | 3 |
| hsa-miR-125b-2-3p | MIMAT0004603 | DDX51 | NM_175066 | 1 | 0 | 0 | 1 | 1 | 3 |
| hsa-miR-125b-2-3p | MIMAT0004603 | CLEC4D | NM_080387 | 1 | 0 | 0 | 1 | 1 | 3 |
| hsa-miR-125b-2-3p | MIMAT0004603 | VSX2 | NM_182894 | 1 | 0 | 0 | 1 | 1 | 3 |
| hsa-miR-125b-2-3p | MIMAT0004603 | C17orf51 | NM_001113434 | 1 | 0 | 0 | 1 | 1 | 3 |
| hsa-miR-125b-2-3p | MIMAT0004603 | ZNF260 | NM_001012756 | 1 | 0 | 0 | 1 | 1 | 3 |
| hsa-miR-125b-2-3p | MIMAT0004603 | CCDC36 | NM_178173 | 1 | 0 | 0 | 1 | 1 | 3 |
| hsa-miR-125b-2-3p | MIMAT0004603 | ARSI | NM_001012301 | 1 | 0 | 0 | 1 | 1 | 3 |
| hsa-miR-125b-2-3p | MIMAT0004603 | CEP85L | NM_001042475 | 1 | 0 | 0 | 1 | 1 | 3 |
| hsa-miR-125b-2-3p | MIMAT0004603 | TEX36 | NM_001128202 | 1 | 0 | 0 | 1 | 1 | 3 |
| hsa-miR-125b-2-3p | MIMAT0004603 | ZNF662 | NM_207404 | 1 | 0 | 0 | 1 | 1 | 3 |
| hsa-miR-125b-2-3p | MIMAT0004603 | TMPRSS11F | NM_207407 | 1 | 0 | 0 | 1 | 1 | 3 |
| hsa-miR-125b-2-3p | MIMAT0004603 | C6orf222 | NM_001010903 | 1 | 0 | 0 | 1 | 1 | 3 |
| hsa-miR-125b-2-3p | MIMAT0004603 | C3orf80 | NM_001168214 | 1 | 0 | 0 | 1 | 1 | 3 |
| hsa-miR-125b-2-3p | MIMAT0004603 | GOLGA8B | XM_005254389 | 1 | 0 | 0 | 1 | 1 | 3 |
| hsa-miR-125b-2-3p | MIMAT0004603 | CAPN14 | NM_001145122 | 1 | 0 | 0 | 1 | 1 | 3 |
| hsa-miR-125b-2-3p | MIMAT0004603 | HRCT1 | NM_001039792 | 1 | 0 | 0 | 1 | 1 | 3 |
| hsa-miR-125b-2-3p | MIMAT0004603 | SIGLEC14 | NM_001098612 | 1 | 0 | 0 | 1 | 1 | 3 |
| hsa-miR-125b-2-3p | MIMAT0004603 | GCH1 | NM_000161 | 1 | 0 | 0 | 1 | 1 | 3 |
| hsa-miR-125b-2-3p | MIMAT0004603 | RAP2A | NM_021033 | 1 | 0 | 0 | 1 | 1 | 3 |
| hsa-miR-125b-2-3p | MIMAT0004603 | RAP2B | NM_002886 | 1 | 0 | 0 | 1 | 1 | 3 |
| hsa-miR-125b-2-3p | MIMAT0004603 | RORB | XM_005252129 | 1 | 0 | 0 | 1 | 1 | 3 |
| hsa-miR-125b-2-3p | MIMAT0004603 | SNURF | NM_005678 | 1 | 0 | 0 | 1 | 1 | 3 |
| hsa-miR-125b-2-3p | MIMAT0004603 | ZDHHC13 | XM_005252995 | 1 | 0 | 0 | 1 | 1 | 3 |
| hsa-miR-125b-2-3p | MIMAT0004603 | GDAP2 | NM_017686 | 1 | 0 | 0 | 1 | 1 | 3 |
| hsa-miR-125b-2-3p | MIMAT0004603 | CXCL16 | NM_022059 | 1 | 0 | 0 | 1 | 1 | 3 |
| hsa-miR-125b-2-3p | MIMAT0004603 | NOL12 | NM_024313 | 1 | 0 | 0 | 1 | 1 | 3 |
| hsa-miR-125b-2-3p | MIMAT0004603 | KRTAP4-5 | NM_033188 | 1 | 0 | 0 | 1 | 1 | 3 |
| hsa-miR-125b-2-3p | MIMAT0004603 | REXO1L1 | XM_005251207 | 1 | 0 | 0 | 1 | 1 | 3 |
| hsa-miR-181a-5p | MIMAT0000256 | ATF1 | NM_005171 | 1 | 1 | 1 | 1 | 1 | 5 |
| hsa-miR-181a-5p | MIMAT0000256 | ATP1B1 | NM_001677 | 1 | 1 | 1 | 1 | 1 | 5 |
| hsa-miR-181a-5p | MIMAT0000256 | ATP2A2 | NM_170665 | 1 | 1 | 1 | 1 | 1 | 5 |
| hsa-miR-181a-5p | MIMAT0000256 | ZFP36L2 | NM_006887 | 1 | 1 | 1 | 1 | 1 | 5 |
| hsa-miR-181a-5p | MIMAT0000256 | CALB1 | NM_004929 | 1 | 1 | 1 | 1 | 1 | 5 |
| hsa-miR-181a-5p | MIMAT0000256 | CALCR | NM_001164737 | 1 | 1 | 1 | 1 | 1 | 5 |
| hsa-miR-181a-5p | MIMAT0000256 | CAMK2D | NM_001221 | 1 | 1 | 1 | 1 | 1 | 5 |
| hsa-miR-181a-5p | MIMAT0000256 | CEBPG | NM_001806 | 1 | 1 | 1 | 1 | 1 | 5 |
| hsa-miR-181a-5p | MIMAT0000256 | KLF6 | NM_001300 | 1 | 1 | 1 | 1 | 1 | 5 |
| hsa-miR-181a-5p | MIMAT0000256 | CPD | NM_001304 | 1 | 1 | 1 | 1 | 1 | 5 |
| hsa-miR-181a-5p | MIMAT0000256 | CPOX | NM_000097 | 1 | 1 | 1 | 1 | 1 | 5 |
| hsa-miR-181a-5p | MIMAT0000256 | DCN | NM_001920 | 1 | 1 | 1 | 1 | 1 | 5 |
| hsa-miR-181a-5p | MIMAT0000256 | DYNC1LI2 | NM_006141 | 1 | 1 | 1 | 1 | 1 | 5 |
| hsa-miR-181a-5p | MIMAT0000256 | MEGF9 | NM_001080497 | 1 | 1 | 1 | 1 | 1 | 5 |
| hsa-miR-181a-5p | MIMAT0000256 | EREG | NM_001432 | 1 | 1 | 1 | 1 | 1 | 5 |
| hsa-miR-181a-5p | MIMAT0000256 | ESR1 | NM_001122742 | 1 | 1 | 1 | 1 | 1 | 5 |
| hsa-miR-181a-5p | MIMAT0000256 | FBN2 | NM_001999 | 1 | 1 | 1 | 1 | 1 | 5 |
| hsa-miR-181a-5p | MIMAT0000256 | FKBP1A | NM_000801 | 1 | 1 | 1 | 1 | 1 | 5 |
| hsa-miR-181a-5p | MIMAT0000256 | AFF2 | NM_002025 | 1 | 1 | 1 | 1 | 1 | 5 |
| hsa-miR-181a-5p | MIMAT0000256 | NR6A1 | NM_033334 | 1 | 1 | 1 | 1 | 1 | 5 |
| hsa-miR-181a-5p | MIMAT0000256 | GFPT1 | NM_001244710 | 1 | 1 | 1 | 1 | 1 | 5 |
| hsa-miR-181a-5p | MIMAT0000256 | GLS | NM_014905 | 1 | 1 | 1 | 1 | 1 | 5 |
| hsa-miR-181a-5p | MIMAT0000256 | GLRX | NM_002064 | 1 | 1 | 1 | 1 | 1 | 5 |
| hsa-miR-181a-5p | MIMAT0000256 | GNA12 | NM_007353 | 1 | 1 | 1 | 1 | 1 | 5 |
| hsa-miR-181a-5p | MIMAT0000256 | NR3C1 | NM_001018077 | 1 | 1 | 1 | 1 | 1 | 5 |
| hsa-miR-181a-5p | MIMAT0000256 | HK2 | NM_000189 | 1 | 1 | 1 | 1 | 1 | 5 |
| hsa-miR-181a-5p | MIMAT0000256 | ITGA2 | NM_002203 | 1 | 1 | 1 | 1 | 1 | 5 |
| hsa-miR-181a-5p | MIMAT0000256 | KPNA4 | NM_002268 | 1 | 1 | 1 | 1 | 1 | 5 |
| hsa-miR-181a-5p | MIMAT0000256 | TNPO1 | NM_002270 | 1 | 1 | 1 | 1 | 1 | 5 |
| hsa-miR-181a-5p | MIMAT0000256 | KRAS | NM_033360 | 1 | 1 | 1 | 1 | 1 | 5 |
| hsa-miR-181a-5p | MIMAT0000256 | LAMP2 | NM_002294 | 1 | 1 | 1 | 1 | 1 | 5 |
| hsa-miR-181a-5p | MIMAT0000256 | LIFR | NM_001127671 | 1 | 1 | 1 | 1 | 1 | 5 |
| hsa-miR-181a-5p | MIMAT0000256 | LMAN1 | NM_005570 | 1 | 1 | 1 | 1 | 1 | 5 |
| hsa-miR-181a-5p | MIMAT0000256 | LOX | NM_002317 | 1 | 1 | 1 | 1 | 1 | 5 |
| hsa-miR-181a-5p | MIMAT0000256 | MAP1B | NM_005909 | 1 | 1 | 1 | 1 | 1 | 5 |
| hsa-miR-181a-5p | MIMAT0000256 | MDM4 | NM_002393 | 1 | 1 | 1 | 1 | 1 | 5 |
| hsa-miR-181a-5p | MIMAT0000256 | KITLG | NM_000899 | 1 | 1 | 1 | 1 | 1 | 5 |
| hsa-miR-181a-5p | MIMAT0000256 | MIP | NM_012064 | 1 | 1 | 1 | 1 | 1 | 5 |
| hsa-miR-181a-5p | MIMAT0000256 | ATXN3 | NM_004993 | 1 | 1 | 1 | 1 | 1 | 5 |
| hsa-miR-181a-5p | MIMAT0000256 | MKLN1 | NM_001145354 | 1 | 1 | 1 | 1 | 1 | 5 |
| hsa-miR-181a-5p | MIMAT0000256 | KMT2A | NM_001197104 | 1 | 1 | 1 | 1 | 1 | 5 |
| hsa-miR-181a-5p | MIMAT0000256 | MME | NM_007289 | 1 | 1 | 1 | 1 | 1 | 5 |
| hsa-miR-181a-5p | MIMAT0000256 | NAP1L1 | NM_004537 | 1 | 1 | 1 | 1 | 1 | 5 |
| hsa-miR-181a-5p | MIMAT0000256 | NFYB | NM_006166 | 1 | 1 | 1 | 1 | 1 | 5 |
| hsa-miR-181a-5p | MIMAT0000256 | NOTCH2 | NM_024408 | 1 | 1 | 1 | 1 | 1 | 5 |
| hsa-miR-181a-5p | MIMAT0000256 | NRAS | NM_002524 | 1 | 1 | 1 | 1 | 1 | 5 |
| hsa-miR-181a-5p | MIMAT0000256 | PAFAH1B2 | NM_002572 | 1 | 1 | 1 | 1 | 1 | 5 |
| hsa-miR-181a-5p | MIMAT0000256 | SERPINE1 | NM_000602 | 1 | 1 | 1 | 1 | 1 | 5 |
| hsa-miR-181a-5p | MIMAT0000256 | PARK2 | NM_004562 | 1 | 1 | 1 | 1 | 1 | 5 |
| hsa-miR-181a-5p | MIMAT0000256 | PAX6 | NM_000280 | 1 | 1 | 1 | 1 | 1 | 5 |
| hsa-miR-181a-5p | MIMAT0000256 | PBX3 | NM_006195 | 1 | 1 | 1 | 1 | 1 | 5 |
| hsa-miR-181a-5p | MIMAT0000256 | ENPP1 | NM_006208 | 1 | 1 | 1 | 1 | 1 | 5 |
| hsa-miR-181a-5p | MIMAT0000256 | SLC26A4 | NM_000441 | 1 | 1 | 1 | 1 | 1 | 5 |
| hsa-miR-181a-5p | MIMAT0000256 | PGR | NM_000926 | 1 | 1 | 1 | 1 | 1 | 5 |
| hsa-miR-181a-5p | MIMAT0000256 | PKNOX1 | NM_004571 | 1 | 1 | 1 | 1 | 1 | 5 |
| hsa-miR-181a-5p | MIMAT0000256 | PLAG1 | NM_002655 | 1 | 1 | 1 | 1 | 1 | 5 |
| hsa-miR-181a-5p | MIMAT0000256 | PLAU | NM_001145031 | 1 | 1 | 1 | 1 | 1 | 5 |
| hsa-miR-181a-5p | MIMAT0000256 | UBL3 | NM_007106 | 1 | 1 | 1 | 1 | 1 | 5 |
| hsa-miR-181a-5p | MIMAT0000256 | POMC | NM_001035256 | 1 | 1 | 1 | 1 | 1 | 5 |
| hsa-miR-181a-5p | MIMAT0000256 | POU2F1 | NM_001198783 | 1 | 1 | 1 | 1 | 1 | 5 |
| hsa-miR-181a-5p | MIMAT0000256 | PPARA | NM_005036 | 1 | 1 | 1 | 1 | 1 | 5 |
| hsa-miR-181a-5p | MIMAT0000256 | PPP1R3D | NM_006242 | 1 | 1 | 1 | 1 | 1 | 5 |
| hsa-miR-181a-5p | MIMAT0000256 | PPP3R1 | NM_000945 | 1 | 1 | 1 | 1 | 1 | 5 |
| hsa-miR-181a-5p | MIMAT0000256 | PRKG1 | NM_001098512 | 1 | 1 | 1 | 1 | 1 | 5 |
| hsa-miR-181a-5p | MIMAT0000256 | MAPK1 | NM_002745 | 1 | 1 | 1 | 1 | 1 | 5 |
| hsa-miR-181a-5p | MIMAT0000256 | PRLR | NM_000949 | 1 | 1 | 1 | 1 | 1 | 5 |
| hsa-miR-181a-5p | MIMAT0000256 | PTGER3 | NM_198715 | 1 | 1 | 1 | 1 | 1 | 5 |
| hsa-miR-181a-5p | MIMAT0000256 | PTPN9 | NM_002833 | 1 | 1 | 1 | 1 | 1 | 5 |
| hsa-miR-181a-5p | MIMAT0000256 | PTPRB | NM_001109754 | 1 | 1 | 1 | 1 | 1 | 5 |
| hsa-miR-181a-5p | MIMAT0000256 | RAB27B | NM_004163 | 1 | 1 | 1 | 1 | 1 | 5 |
| hsa-miR-181a-5p | MIMAT0000256 | RAD21 | NM_006265 | 1 | 1 | 1 | 1 | 1 | 5 |
| hsa-miR-181a-5p | MIMAT0000256 | RAP2A | NM_021033 | 1 | 1 | 1 | 1 | 1 | 5 |
| hsa-miR-181a-5p | MIMAT0000256 | KDM5A | NM_001042603 | 1 | 1 | 1 | 1 | 1 | 5 |
| hsa-miR-181a-5p | MIMAT0000256 | ROBO2 | NM_002942 | 1 | 1 | 1 | 1 | 1 | 5 |
| hsa-miR-181a-5p | MIMAT0000256 | RORA | NM_134260 | 1 | 1 | 1 | 1 | 1 | 5 |
| hsa-miR-181a-5p | MIMAT0000256 | ATXN1 | NM_000332 | 1 | 1 | 1 | 1 | 1 | 5 |
| hsa-miR-181a-5p | MIMAT0000256 | ATXN7 | NM_000333 | 1 | 1 | 1 | 1 | 1 | 5 |
| hsa-miR-181a-5p | MIMAT0000256 | SCD | NM_005063 | 1 | 1 | 1 | 1 | 1 | 5 |
| hsa-miR-181a-5p | MIMAT0000256 | SCN9A | NM_002977 | 1 | 1 | 1 | 1 | 1 | 5 |
| hsa-miR-181a-5p | MIMAT0000256 | SEL1L | NM_005065 | 1 | 1 | 1 | 1 | 1 | 5 |
| hsa-miR-181a-5p | MIMAT0000256 | SRSF7 | NM_001031684 | 1 | 1 | 1 | 1 | 1 | 5 |
| hsa-miR-181a-5p | MIMAT0000256 | SKP1 | NM_006930 | 1 | 1 | 1 | 1 | 1 | 5 |
| hsa-miR-181a-5p | MIMAT0000256 | SLC5A3 | NM_006933 | 1 | 1 | 1 | 1 | 1 | 5 |
| hsa-miR-181a-5p | MIMAT0000256 | SLC7A2 | NM_001008539 | 1 | 1 | 1 | 1 | 1 | 5 |
| hsa-miR-181a-5p | MIMAT0000256 | SMN1 | NM_000344 | 1 | 1 | 1 | 1 | 1 | 5 |
| hsa-miR-181a-5p | MIMAT0000256 | SOX5 | NM_152989 | 1 | 1 | 1 | 1 | 1 | 5 |
| hsa-miR-181a-5p | MIMAT0000256 | STC1 | NM_003155 | 1 | 1 | 1 | 1 | 1 | 5 |
| hsa-miR-181a-5p | MIMAT0000256 | TBL1X | NM_005647 | 1 | 1 | 1 | 1 | 1 | 5 |
| hsa-miR-181a-5p | MIMAT0000256 | TFRC | NM_003234 | 1 | 1 | 1 | 1 | 1 | 5 |
| hsa-miR-181a-5p | MIMAT0000256 | TGFBI | NM_000358 | 1 | 1 | 1 | 1 | 1 | 5 |
| hsa-miR-181a-5p | MIMAT0000256 | TGFBR1 | NM_004612 | 1 | 1 | 1 | 1 | 1 | 5 |
| hsa-miR-181a-5p | MIMAT0000256 | TIMP3 | NM_000362 | 1 | 1 | 1 | 1 | 1 | 5 |
| hsa-miR-181a-5p | MIMAT0000256 | TMF1 | NM_007114 | 1 | 1 | 1 | 1 | 1 | 5 |
| hsa-miR-181a-5p | MIMAT0000256 | VIP | NM_003381 | 1 | 1 | 1 | 1 | 1 | 5 |
| hsa-miR-181a-5p | MIMAT0000256 | YWHAG | NM_012479 | 1 | 1 | 1 | 1 | 1 | 5 |
| hsa-miR-181a-5p | MIMAT0000256 | ZIC2 | NM_007129 | 1 | 1 | 1 | 1 | 1 | 5 |
| hsa-miR-181a-5p | MIMAT0000256 | ZNF28 | NM_006969 | 1 | 1 | 1 | 1 | 1 | 5 |
| hsa-miR-181a-5p | MIMAT0000256 | ST8SIA4 | NM_005668 | 1 | 1 | 1 | 1 | 1 | 5 |
| hsa-miR-181a-5p | MIMAT0000256 | NR4A3 | NM_173200 | 1 | 1 | 1 | 1 | 1 | 5 |
| hsa-miR-181a-5p | MIMAT0000256 | CUL3 | NM_003590 | 1 | 1 | 1 | 1 | 1 | 5 |
| hsa-miR-181a-5p | MIMAT0000256 | ATRN | NM_139321 | 1 | 1 | 1 | 1 | 1 | 5 |
| hsa-miR-181a-5p | MIMAT0000256 | PIK3R3 | NM_003629 | 1 | 1 | 1 | 1 | 1 | 5 |
| hsa-miR-181a-5p | MIMAT0000256 | GAS7 | NM_201433 | 1 | 1 | 1 | 1 | 1 | 5 |
| hsa-miR-181a-5p | MIMAT0000256 | CBX4 | NM_003655 | 1 | 1 | 1 | 1 | 1 | 5 |
| hsa-miR-181a-5p | MIMAT0000256 | CDC14B | NM_033331 | 1 | 1 | 1 | 1 | 1 | 5 |
| hsa-miR-181a-5p | MIMAT0000256 | DDX3Y | NM_001122665 | 1 | 1 | 1 | 1 | 1 | 5 |
| hsa-miR-181a-5p | MIMAT0000256 | PDE5A | NM_001083 | 1 | 1 | 1 | 1 | 1 | 5 |
| hsa-miR-181a-5p | MIMAT0000256 | IRS2 | NM_003749 | 1 | 1 | 1 | 1 | 1 | 5 |
| hsa-miR-181a-5p | MIMAT0000256 | RNMT | NM_003799 | 1 | 1 | 1 | 1 | 1 | 5 |
| hsa-miR-181a-5p | MIMAT0000256 | RAB11A | NM_004663 | 1 | 1 | 1 | 1 | 1 | 5 |
| hsa-miR-181a-5p | MIMAT0000256 | ARHGEF7 | NM_001113511 | 1 | 1 | 1 | 1 | 1 | 5 |
| hsa-miR-181a-5p | MIMAT0000256 | RNF8 | NM_003958 | 1 | 1 | 1 | 1 | 1 | 5 |
| hsa-miR-181a-5p | MIMAT0000256 | COPS2 | NM_001143887 | 1 | 1 | 1 | 1 | 1 | 5 |
| hsa-miR-181a-5p | MIMAT0000256 | KIF3B | NM_004798 | 1 | 1 | 1 | 1 | 1 | 5 |
| hsa-miR-181a-5p | MIMAT0000256 | ATG5 | NM_004849 | 1 | 1 | 1 | 1 | 1 | 5 |
| hsa-miR-181a-5p | MIMAT0000256 | ONECUT2 | NM_004852 | 1 | 1 | 1 | 1 | 1 | 5 |
| hsa-miR-181a-5p | MIMAT0000256 | SLC4A8 | NM_001039960 | 1 | 1 | 1 | 1 | 1 | 5 |
| hsa-miR-181a-5p | MIMAT0000256 | CREB5 | NM_182898 | 1 | 1 | 1 | 1 | 1 | 5 |
| hsa-miR-181a-5p | MIMAT0000256 | GDA | NM_001242506 | 1 | 1 | 1 | 1 | 1 | 5 |
| hsa-miR-181a-5p | MIMAT0000256 | PHACTR2 | NM_001100164 | 1 | 1 | 1 | 1 | 1 | 5 |
| hsa-miR-181a-5p | MIMAT0000256 | TOX | NM_014729 | 1 | 1 | 1 | 1 | 1 | 5 |
| hsa-miR-181a-5p | MIMAT0000256 | KIAA0247 | NM_014734 | 1 | 1 | 1 | 1 | 1 | 5 |
| hsa-miR-181a-5p | MIMAT0000256 | KIAA0195 | NM_014738 | 1 | 1 | 1 | 1 | 1 | 5 |
| hsa-miR-181a-5p | MIMAT0000256 | PTBP3 | NM_001244898 | 1 | 1 | 1 | 1 | 1 | 5 |
| hsa-miR-181a-5p | MIMAT0000256 | CALCRL | NM_005795 | 1 | 1 | 1 | 1 | 1 | 5 |
| hsa-miR-181a-5p | MIMAT0000256 | CTDSPL | NM_001008392 | 1 | 1 | 1 | 1 | 1 | 5 |
| hsa-miR-181a-5p | MIMAT0000256 | SEMA3C | NM_006379 | 1 | 1 | 1 | 1 | 1 | 5 |
| hsa-miR-181a-5p | MIMAT0000256 | SLC19A2 | NM_006996 | 1 | 1 | 1 | 1 | 1 | 5 |
| hsa-miR-181a-5p | MIMAT0000256 | PRDX3 | NM_006793 | 1 | 1 | 1 | 1 | 1 | 5 |
| hsa-miR-181a-5p | MIMAT0000256 | METAP2 | NM_006838 | 1 | 1 | 1 | 1 | 1 | 5 |
| hsa-miR-181a-5p | MIMAT0000256 | NUDT21 | NM_007006 | 1 | 1 | 1 | 1 | 1 | 5 |
| hsa-miR-181a-5p | MIMAT0000256 | DDX52 | NM_007010 | 1 | 1 | 1 | 1 | 1 | 5 |
| hsa-miR-181a-5p | MIMAT0000256 | ADAMTS5 | NM_007038 | 1 | 1 | 1 | 1 | 1 | 5 |
| hsa-miR-181a-5p | MIMAT0000256 | NUDT4 | NM_199040 | 1 | 1 | 1 | 1 | 1 | 5 |
| hsa-miR-181a-5p | MIMAT0000256 | RASSF8 | NM_001164746 | 1 | 1 | 1 | 1 | 1 | 5 |
| hsa-miR-181a-5p | MIMAT0000256 | CA5B | NM_007220 | 1 | 1 | 1 | 1 | 1 | 5 |
| hsa-miR-181a-5p | MIMAT0000256 | PDAP1 | NM_014891 | 1 | 1 | 1 | 1 | 1 | 5 |
| hsa-miR-181a-5p | MIMAT0000256 | MTF2 | NM_007358 | 1 | 1 | 1 | 1 | 1 | 5 |
| hsa-miR-181a-5p | MIMAT0000256 | BTBD3 | NM_014962 | 1 | 1 | 1 | 1 | 1 | 5 |
| hsa-miR-181a-5p | MIMAT0000256 | WDFY3 | NM_014991 | 1 | 1 | 1 | 1 | 1 | 5 |
| hsa-miR-181a-5p | MIMAT0000256 | USP33 | NM_015017 | 1 | 1 | 1 | 1 | 1 | 5 |
| hsa-miR-181a-5p | MIMAT0000256 | ENDOD1 | NM_015036 | 1 | 1 | 1 | 1 | 1 | 5 |
| hsa-miR-181a-5p | MIMAT0000256 | RCOR1 | NM_015156 | 1 | 1 | 1 | 1 | 1 | 5 |
| hsa-miR-181a-5p | MIMAT0000256 | SIRT1 | NM_012238 | 1 | 1 | 1 | 1 | 1 | 5 |
| hsa-miR-181a-5p | MIMAT0000256 | NPTXR | NM_014293 | 1 | 1 | 1 | 1 | 1 | 5 |
| hsa-miR-181a-5p | MIMAT0000256 | CBX7 | NM_175709 | 1 | 1 | 1 | 1 | 1 | 5 |
| hsa-miR-181a-5p | MIMAT0000256 | SLC7A11 | NM_014331 | 1 | 1 | 1 | 1 | 1 | 5 |
| hsa-miR-181a-5p | MIMAT0000256 | KIAA1549L | NM_012194 | 1 | 1 | 1 | 1 | 1 | 5 |
| hsa-miR-181a-5p | MIMAT0000256 | AHCTF1 | NM_015446 | 1 | 1 | 1 | 1 | 1 | 5 |
| hsa-miR-181a-5p | MIMAT0000256 | CHMP2B | NM_014043 | 1 | 1 | 1 | 1 | 1 | 5 |
| hsa-miR-181a-5p | MIMAT0000256 | OSBPL3 | NM_015550 | 1 | 1 | 1 | 1 | 1 | 5 |
| hsa-miR-181a-5p | MIMAT0000256 | ARL5A | NM_012097 | 1 | 1 | 1 | 1 | 1 | 5 |
| hsa-miR-181a-5p | MIMAT0000256 | BLOC1S6 | NM_012388 | 1 | 1 | 1 | 1 | 1 | 5 |
| hsa-miR-181a-5p | MIMAT0000256 | NUFIP1 | NM_012345 | 1 | 1 | 1 | 1 | 1 | 5 |
| hsa-miR-181a-5p | MIMAT0000256 | GHITM | NM_014394 | 1 | 1 | 1 | 1 | 1 | 5 |
| hsa-miR-181a-5p | MIMAT0000256 | FOXP1 | NM_001244808 | 1 | 1 | 1 | 1 | 1 | 5 |
| hsa-miR-181a-5p | MIMAT0000256 | YPEL1 | NM_013313 | 1 | 1 | 1 | 1 | 1 | 5 |
| hsa-miR-181a-5p | MIMAT0000256 | RRP15 | NM_016052 | 1 | 1 | 1 | 1 | 1 | 5 |
| hsa-miR-181a-5p | MIMAT0000256 | KLF3 | NM_016531 | 1 | 1 | 1 | 1 | 1 | 5 |
| hsa-miR-181a-5p | MIMAT0000256 | HOOK1 | NM_015888 | 1 | 1 | 1 | 1 | 1 | 5 |
| hsa-miR-181a-5p | MIMAT0000256 | SELT | NM_016275 | 1 | 1 | 1 | 1 | 1 | 5 |
| hsa-miR-181a-5p | MIMAT0000256 | BRWD1 | NM_033656 | 1 | 1 | 1 | 1 | 1 | 5 |
| hsa-miR-181a-5p | MIMAT0000256 | SIAE | NM_001199922 | 1 | 1 | 1 | 1 | 1 | 5 |
| hsa-miR-181a-5p | MIMAT0000256 | CNNM2 | NM_017649 | 1 | 1 | 1 | 1 | 1 | 5 |
| hsa-miR-181a-5p | MIMAT0000256 | LPCAT2 | NM_017839 | 1 | 1 | 1 | 1 | 1 | 5 |
| hsa-miR-181a-5p | MIMAT0000256 | TMEM255A | NM_017938 | 1 | 1 | 1 | 1 | 1 | 5 |
| hsa-miR-181a-5p | MIMAT0000256 | LRRC8D | NM_001134479 | 1 | 1 | 1 | 1 | 1 | 5 |
| hsa-miR-181a-5p | MIMAT0000256 | CCDC25 | NM_018246 | 1 | 1 | 1 | 1 | 1 | 5 |
| hsa-miR-181a-5p | MIMAT0000256 | PI4K2B | NM_018323 | 1 | 1 | 1 | 1 | 1 | 5 |
| hsa-miR-181a-5p | MIMAT0000256 | SOX6 | NM_017508 | 1 | 1 | 1 | 1 | 1 | 5 |
| hsa-miR-181a-5p | MIMAT0000256 | NDC1 | NM_018087 | 1 | 1 | 1 | 1 | 1 | 5 |
| hsa-miR-181a-5p | MIMAT0000256 | EXOC2 | NM_018303 | 1 | 1 | 1 | 1 | 1 | 5 |
| hsa-miR-181a-5p | MIMAT0000256 | LMO3 | NM_018640 | 1 | 1 | 1 | 1 | 1 | 5 |
| hsa-miR-181a-5p | MIMAT0000256 | KMT2E | NM_182931 | 1 | 1 | 1 | 1 | 1 | 5 |
| hsa-miR-181a-5p | MIMAT0000256 | PCDHA6 | NM_018909 | 1 | 1 | 1 | 1 | 1 | 5 |
| hsa-miR-181a-5p | MIMAT0000256 | TM9SF3 | NM_020123 | 1 | 1 | 1 | 1 | 1 | 5 |
| hsa-miR-181a-5p | MIMAT0000256 | PHTF2 | NM_001127358 | 1 | 1 | 1 | 1 | 1 | 5 |
| hsa-miR-181a-5p | MIMAT0000256 | KIAA1244 | NM_020340 | 1 | 1 | 1 | 1 | 1 | 5 |
| hsa-miR-181a-5p | MIMAT0000256 | NLN | NM_020726 | 1 | 1 | 1 | 1 | 1 | 5 |
| hsa-miR-181a-5p | MIMAT0000256 | KIAA1239 | NM_001144990 | 1 | 1 | 1 | 1 | 1 | 5 |
| hsa-miR-181a-5p | MIMAT0000256 | NUFIP2 | NM_020772 | 1 | 1 | 1 | 1 | 1 | 5 |
| hsa-miR-181a-5p | MIMAT0000256 | KLHL42 | NM_020782 | 1 | 1 | 1 | 1 | 1 | 5 |
| hsa-miR-181a-5p | MIMAT0000256 | ZBTB4 | NM_020899 | 1 | 1 | 1 | 1 | 1 | 5 |
| hsa-miR-181a-5p | MIMAT0000256 | BEND3 | NM_001080450 | 1 | 1 | 1 | 1 | 1 | 5 |
| hsa-miR-181a-5p | MIMAT0000256 | PAPD5 | NM_001040284 | 1 | 1 | 1 | 1 | 1 | 5 |
| hsa-miR-181a-5p | MIMAT0000256 | NUCKS1 | NM_022731 | 1 | 1 | 1 | 1 | 1 | 5 |
| hsa-miR-181a-5p | MIMAT0000256 | TNS3 | NM_022748 | 1 | 1 | 1 | 1 | 1 | 5 |
| hsa-miR-181a-5p | MIMAT0000256 | FNDC3B | NM_022763 | 1 | 1 | 1 | 1 | 1 | 5 |
| hsa-miR-181a-5p | MIMAT0000256 | NDRG4 | NM_001130487 | 1 | 1 | 1 | 1 | 1 | 5 |
| hsa-miR-181a-5p | MIMAT0000256 | GPBP1 | NM_022913 | 1 | 1 | 1 | 1 | 1 | 5 |
| hsa-miR-181a-5p | MIMAT0000256 | PLEKHA3 | NM_019091 | 1 | 1 | 1 | 1 | 1 | 5 |
| hsa-miR-181a-5p | MIMAT0000256 | HAUS3 | NM_024511 | 1 | 1 | 1 | 1 | 1 | 5 |
| hsa-miR-181a-5p | MIMAT0000256 | PPP1R3B | NM_001201329 | 1 | 1 | 1 | 1 | 1 | 5 |
| hsa-miR-181a-5p | MIMAT0000256 | NARG2 | NM_024611 | 1 | 1 | 1 | 1 | 1 | 5 |
| hsa-miR-181a-5p | MIMAT0000256 | QSER1 | NM_001076786 | 1 | 1 | 1 | 1 | 1 | 5 |
| hsa-miR-181a-5p | MIMAT0000256 | RNF34 | NM_194271 | 1 | 1 | 1 | 1 | 1 | 5 |
| hsa-miR-181a-5p | MIMAT0000256 | PROSER1 | NM_025138 | 1 | 1 | 1 | 1 | 1 | 5 |
| hsa-miR-181a-5p | MIMAT0000256 | CPEB4 | NM_030627 | 1 | 1 | 1 | 1 | 1 | 5 |
| hsa-miR-181a-5p | MIMAT0000256 | VANGL1 | NM_138959 | 1 | 1 | 1 | 1 | 1 | 5 |
| hsa-miR-181a-5p | MIMAT0000256 | PCDH11Y | NM_032973 | 1 | 1 | 1 | 1 | 1 | 5 |
| hsa-miR-181a-5p | MIMAT0000256 | THAP2 | NM_031435 | 1 | 1 | 1 | 1 | 1 | 5 |
| hsa-miR-181a-5p | MIMAT0000256 | LCOR | NM_032440 | 1 | 1 | 1 | 1 | 1 | 5 |
| hsa-miR-181a-5p | MIMAT0000256 | ADO | NM_032804 | 1 | 1 | 1 | 1 | 1 | 5 |
| hsa-miR-181a-5p | MIMAT0000256 | TMEM87B | NM_032824 | 1 | 1 | 1 | 1 | 1 | 5 |
| hsa-miR-181a-5p | MIMAT0000256 | FAM105B | NM_138348 | 1 | 1 | 1 | 1 | 1 | 5 |
| hsa-miR-181a-5p | MIMAT0000256 | ERI1 | NM_153332 | 1 | 1 | 1 | 1 | 1 | 5 |
| hsa-miR-181a-5p | MIMAT0000256 | ELMSAN1 | NM_194278 | 1 | 1 | 1 | 1 | 1 | 5 |
| hsa-miR-181a-5p | MIMAT0000256 | ARRDC4 | NM_183376 | 1 | 1 | 1 | 1 | 1 | 5 |
| hsa-miR-181a-5p | MIMAT0000256 | ADAMTSL1 | NM_001040272 | 1 | 1 | 1 | 1 | 1 | 5 |
| hsa-miR-181a-5p | MIMAT0000256 | ZFAND4 | NM_174890 | 1 | 1 | 1 | 1 | 1 | 5 |
| hsa-miR-181a-5p | MIMAT0000256 | MED12L | NM_053002 | 1 | 1 | 1 | 1 | 1 | 5 |
| hsa-miR-181a-5p | MIMAT0000256 | ANTXR2 | NM_058172 | 1 | 1 | 1 | 1 | 1 | 5 |
| hsa-miR-181a-5p | MIMAT0000256 | SOCS4 | NM_199421 | 1 | 1 | 1 | 1 | 1 | 5 |
| hsa-miR-181a-5p | MIMAT0000256 | WHAMM | NM_001080435 | 1 | 1 | 1 | 1 | 1 | 5 |
| hsa-miR-181a-5p | MIMAT0000256 | SHE | NM_001010846 | 1 | 1 | 1 | 1 | 1 | 5 |
| hsa-miR-181a-5p | MIMAT0000256 | C5orf47 | NM_001144954 | 1 | 1 | 1 | 1 | 1 | 5 |
| hsa-miR-181a-5p | MIMAT0000256 | PRRC1 | NM_130809 | 1 | 1 | 1 | 1 | 1 | 5 |
| hsa-miR-181a-5p | MIMAT0000256 | SOWAHA | NM_175873 | 1 | 1 | 1 | 1 | 1 | 5 |
| hsa-miR-181a-5p | MIMAT0000256 | NEK7 | NM_133494 | 1 | 1 | 1 | 1 | 1 | 5 |
| hsa-miR-181a-5p | MIMAT0000256 | VTI1A | NM_145206 | 1 | 1 | 1 | 1 | 1 | 5 |
| hsa-miR-181a-5p | MIMAT0000256 | E2F7 | NM_203394 | 1 | 1 | 1 | 1 | 1 | 5 |
| hsa-miR-181a-5p | MIMAT0000256 | CCT8L2 | NM_014406 | 1 | 1 | 1 | 1 | 1 | 5 |
| hsa-miR-181a-5p | MIMAT0000256 | ZNF780B | NM_001005851 | 1 | 1 | 1 | 1 | 1 | 5 |
| hsa-miR-181a-5p | MIMAT0000256 | TOR1AIP2 | NM_001199260 | 1 | 1 | 1 | 1 | 1 | 5 |
| hsa-miR-181a-5p | MIMAT0000256 | MIER3 | NM_152622 | 1 | 1 | 1 | 1 | 1 | 5 |
| hsa-miR-181a-5p | MIMAT0000256 | TMEM64 | NM_001008495 | 1 | 1 | 1 | 1 | 1 | 5 |
| hsa-miR-181a-5p | MIMAT0000256 | ADAMTS18 | NM_199355 | 1 | 1 | 1 | 1 | 1 | 5 |
| hsa-miR-181a-5p | MIMAT0000256 | AGO4 | NM_017629 | 1 | 1 | 1 | 1 | 1 | 5 |
| hsa-miR-181a-5p | MIMAT0000256 | C2orf69 | NM_153689 | 1 | 1 | 1 | 1 | 1 | 5 |
| hsa-miR-181a-5p | MIMAT0000256 | JAZF1 | NM_175061 | 1 | 1 | 1 | 1 | 1 | 5 |
| hsa-miR-181a-5p | MIMAT0000256 | FOXK1 | NM_001037165 | 1 | 1 | 1 | 1 | 1 | 5 |
| hsa-miR-181a-5p | MIMAT0000256 | KIAA1324L | NM_001142749 | 1 | 1 | 1 | 1 | 1 | 5 |
| hsa-miR-181a-5p | MIMAT0000256 | TMEM196 | NM_152774 | 1 | 1 | 1 | 1 | 1 | 5 |
| hsa-miR-181a-5p | MIMAT0000256 | CXorf23 | NM_198279 | 1 | 1 | 1 | 1 | 1 | 5 |
| hsa-miR-181a-5p | MIMAT0000256 | PRTG | NM_173814 | 1 | 1 | 1 | 1 | 1 | 5 |
| hsa-miR-181a-5p | MIMAT0000256 | MICU3 | NM_181723 | 1 | 1 | 1 | 1 | 1 | 5 |
| hsa-miR-181a-5p | MIMAT0000256 | ATP11C | NM_173694 | 1 | 1 | 1 | 1 | 1 | 5 |
| hsa-miR-181a-5p | MIMAT0000256 | HEPHL1 | NM_001098672 | 1 | 1 | 1 | 1 | 1 | 5 |
| hsa-miR-181a-5p | MIMAT0000256 | GLDN | NM_181789 | 1 | 1 | 1 | 1 | 1 | 5 |
| hsa-miR-181a-5p | MIMAT0000256 | ZC3H6 | NM_198581 | 1 | 1 | 1 | 1 | 1 | 5 |
| hsa-miR-181a-5p | MIMAT0000256 | CCDC88C | NM_001080414 | 1 | 1 | 1 | 1 | 1 | 5 |
| hsa-miR-181a-5p | MIMAT0000256 | CLN8 | NM_018941 | 1 | 1 | 1 | 1 | 1 | 5 |
| hsa-miR-181a-5p | MIMAT0000256 | FUT1 | NM_000148 | 1 | 1 | 1 | 1 | 1 | 5 |
| hsa-miR-181a-5p | MIMAT0000256 | GRIN2A | NM_001134407 | 1 | 1 | 1 | 1 | 1 | 5 |
| hsa-miR-181a-5p | MIMAT0000256 | CXCL1 | NM_001511 | 1 | 1 | 1 | 1 | 1 | 5 |
| hsa-miR-181a-5p | MIMAT0000256 | PSG3 | NM_021016 | 1 | 1 | 1 | 1 | 1 | 5 |
| hsa-miR-181a-5p | MIMAT0000256 | PSG11 | NM_002785 | 1 | 1 | 1 | 1 | 1 | 5 |
| hsa-miR-181a-5p | MIMAT0000256 | RFC1 | NM_001204747 | 1 | 1 | 1 | 1 | 1 | 5 |
| hsa-miR-181a-5p | MIMAT0000256 | TBXA2R | NM_001060 | 1 | 1 | 1 | 1 | 1 | 5 |
| hsa-miR-181a-5p | MIMAT0000256 | ZNF37A | NM_001007094 | 1 | 1 | 1 | 1 | 1 | 5 |
| hsa-miR-181a-5p | MIMAT0000256 | ZNF74 | NM_003426 | 1 | 1 | 1 | 1 | 1 | 5 |
| hsa-miR-181a-5p | MIMAT0000256 | CD302 | NM_014880 | 1 | 1 | 1 | 1 | 1 | 5 |
| hsa-miR-181a-5p | MIMAT0000256 | PROCR | NM_006404 | 1 | 1 | 1 | 1 | 1 | 5 |
| hsa-miR-181a-5p | MIMAT0000256 | KIF3A | NM_007054 | 1 | 1 | 1 | 1 | 1 | 5 |
| hsa-miR-181a-5p | MIMAT0000256 | CARD8 | NM_001184902 | 1 | 1 | 1 | 1 | 1 | 5 |
| hsa-miR-181a-5p | MIMAT0000256 | FAN1 | NM_014967 | 1 | 1 | 1 | 1 | 1 | 5 |
| hsa-miR-181a-5p | MIMAT0000256 | ZFYVE26 | NM_015346 | 1 | 1 | 1 | 1 | 1 | 5 |
| hsa-miR-181a-5p | MIMAT0000256 | EPC2 | NM_015630 | 1 | 1 | 1 | 1 | 1 | 5 |
| hsa-miR-181a-5p | MIMAT0000256 | TAF5L | NM_001025247 | 1 | 1 | 1 | 1 | 1 | 5 |
| hsa-miR-181a-5p | MIMAT0000256 | IFT81 | NM_014055 | 1 | 1 | 1 | 1 | 1 | 5 |
| hsa-miR-181a-5p | MIMAT0000256 | DMGDH | NM_013391 | 1 | 1 | 1 | 1 | 1 | 5 |
| hsa-miR-181a-5p | MIMAT0000256 | MBTPS2 | NM_015884 | 1 | 1 | 1 | 1 | 1 | 5 |
| hsa-miR-181a-5p | MIMAT0000256 | SLC22A15 | NM_018420 | 1 | 1 | 1 | 1 | 1 | 5 |
| hsa-miR-181a-5p | MIMAT0000256 | PPP1R9A | NM_001166160 | 1 | 1 | 1 | 1 | 1 | 5 |
| hsa-miR-181a-5p | MIMAT0000256 | PCDHAC2 | NM_018899 | 1 | 1 | 1 | 1 | 1 | 5 |
| hsa-miR-181a-5p | MIMAT0000256 | PCDHAC1 | NM_018898 | 1 | 1 | 1 | 1 | 1 | 5 |
| hsa-miR-181a-5p | MIMAT0000256 | PCDHA13 | NM_018904 | 1 | 1 | 1 | 1 | 1 | 5 |
| hsa-miR-181a-5p | MIMAT0000256 | PCDHA12 | NM_018903 | 1 | 1 | 1 | 1 | 1 | 5 |
| hsa-miR-181a-5p | MIMAT0000256 | PCDHA10 | NM_018901 | 1 | 1 | 1 | 1 | 1 | 5 |
| hsa-miR-181a-5p | MIMAT0000256 | PCDHA8 | NM_018911 | 1 | 1 | 1 | 1 | 1 | 5 |
| hsa-miR-181a-5p | MIMAT0000256 | PCDHA7 | NM_018910 | 1 | 1 | 1 | 1 | 1 | 5 |
| hsa-miR-181a-5p | MIMAT0000256 | PCDHA5 | NM_018908 | 1 | 1 | 1 | 1 | 1 | 5 |
| hsa-miR-181a-5p | MIMAT0000256 | PCDHA4 | NM_018907 | 1 | 1 | 1 | 1 | 1 | 5 |
| hsa-miR-181a-5p | MIMAT0000256 | PCDHA3 | NM_018906 | 1 | 1 | 1 | 1 | 1 | 5 |
| hsa-miR-181a-5p | MIMAT0000256 | PCDHA2 | NM_018905 | 1 | 1 | 1 | 1 | 1 | 5 |
| hsa-miR-181a-5p | MIMAT0000256 | PCDHA1 | NM_018900 | 1 | 1 | 1 | 1 | 1 | 5 |
| hsa-miR-181a-5p | MIMAT0000256 | TMEM181 | NM_020823 | 1 | 1 | 1 | 1 | 1 | 5 |
| hsa-miR-181a-5p | MIMAT0000256 | AASDHPPT | NM_015423 | 1 | 1 | 1 | 1 | 1 | 5 |
| hsa-miR-181a-5p | MIMAT0000256 | SCOC | NM_001153585 | 1 | 1 | 1 | 1 | 1 | 5 |
| hsa-miR-181a-5p | MIMAT0000256 | GID4 | NM_024052 | 1 | 1 | 1 | 1 | 1 | 5 |
| hsa-miR-181a-5p | MIMAT0000256 | STEAP4 | NM_001205315 | 1 | 1 | 1 | 1 | 1 | 5 |
| hsa-miR-181a-5p | MIMAT0000256 | ZFP91 | NM_053023 | 1 | 1 | 1 | 1 | 1 | 5 |
| hsa-miR-181a-5p | MIMAT0000256 | CCSAP | NM_145257 | 1 | 1 | 1 | 1 | 1 | 5 |
| hsa-miR-181a-5p | MIMAT0000256 | CCDC42 | NM_144681 | 1 | 1 | 1 | 1 | 1 | 5 |
| hsa-miR-181a-5p | MIMAT0000256 | ZNF555 | NM_152791 | 1 | 1 | 1 | 1 | 1 | 5 |
| hsa-miR-181a-5p | MIMAT0000256 | ESCO2 | NM_001017420 | 1 | 1 | 1 | 1 | 1 | 5 |
| hsa-miR-181a-5p | MIMAT0000256 | EXD1 | NM_152596 | 1 | 1 | 1 | 1 | 1 | 5 |
| hsa-miR-181a-5p | MIMAT0000256 | TMEM26 | NM_178505 | 1 | 1 | 1 | 1 | 1 | 5 |
| hsa-miR-181a-5p | MIMAT0000256 | TWISTNB | NM_001002926 | 1 | 1 | 1 | 1 | 1 | 5 |
| hsa-miR-181a-5p | MIMAT0000256 | CYB561D1 | NM_001134404 | 1 | 1 | 1 | 1 | 1 | 5 |
| hsa-miR-181a-5p | MIMAT0000256 | C11orf87 | NM_207645 | 1 | 1 | 1 | 1 | 1 | 5 |
| hsa-miR-181a-5p | MIMAT0000256 | ABL2 | NM_007314 | 1 | 1 | 0 | 1 | 1 | 4 |
| hsa-miR-181a-5p | MIMAT0000256 | ACVR2A | NM_001278579 | 1 | 1 | 0 | 1 | 1 | 4 |
| hsa-miR-181a-5p | MIMAT0000256 | ADARB1 | NM_015833 | 0 | 1 | 1 | 1 | 1 | 4 |
| hsa-miR-181a-5p | MIMAT0000256 | ADCY1 | NM_021116 | 1 | 1 | 0 | 1 | 1 | 4 |
| hsa-miR-181a-5p | MIMAT0000256 | ADCY2 | NM_020546 | 1 | 1 | 0 | 1 | 1 | 4 |
| hsa-miR-181a-5p | MIMAT0000256 | ADRBK1 | NM_001619 | 0 | 1 | 1 | 1 | 1 | 4 |
| hsa-miR-181a-5p | MIMAT0000256 | AP1G1 | XM_005255823 | 1 | 1 | 0 | 1 | 1 | 4 |
| hsa-miR-181a-5p | MIMAT0000256 | AHR | NM_001621 | 1 | 1 | 0 | 1 | 1 | 4 |
| hsa-miR-181a-5p | MIMAT0000256 | XIAP | NM_001167 | 1 | 1 | 0 | 1 | 1 | 4 |
| hsa-miR-181a-5p | MIMAT0000256 | AR | NM_000044 | 1 | 1 | 0 | 1 | 1 | 4 |
| hsa-miR-181a-5p | MIMAT0000256 | TRIM23 | NM_001656 | 1 | 1 | 0 | 1 | 1 | 4 |
| hsa-miR-181a-5p | MIMAT0000256 | ARL1 | XM_005268869 | 1 | 1 | 0 | 1 | 1 | 4 |
| hsa-miR-181a-5p | MIMAT0000256 | STS | XM_005274512 | 1 | 1 | 0 | 1 | 1 | 4 |
| hsa-miR-181a-5p | MIMAT0000256 | ASPH | XM_005251235 | 1 | 1 | 0 | 1 | 1 | 4 |
| hsa-miR-181a-5p | MIMAT0000256 | RERE | NM_012102 | 1 | 1 | 0 | 1 | 1 | 4 |
| hsa-miR-181a-5p | MIMAT0000256 | ATP1B2 | NM_001678 | 1 | 1 | 1 | 0 | 1 | 4 |
| hsa-miR-181a-5p | MIMAT0000256 | ATP2B1 | XM_005268919 | 1 | 1 | 0 | 1 | 1 | 4 |
| hsa-miR-181a-5p | MIMAT0000256 | ATP2B3 | XM_005274692 | 1 | 1 | 0 | 1 | 1 | 4 |
| hsa-miR-181a-5p | MIMAT0000256 | HCN2 | NM_001194 | 1 | 1 | 0 | 1 | 1 | 4 |
| hsa-miR-181a-5p | MIMAT0000256 | BMPR2 | NM_001204 | 1 | 1 | 0 | 1 | 1 | 4 |
| hsa-miR-181a-5p | MIMAT0000256 | BRAF | XM_005250045 | 1 | 1 | 0 | 1 | 1 | 4 |
| hsa-miR-181a-5p | MIMAT0000256 | ZFP36L1 | NM_004926 | 1 | 1 | 1 | 0 | 1 | 4 |
| hsa-miR-181a-5p | MIMAT0000256 | LDLRAD4 | NM_001003674 | 1 | 1 | 0 | 1 | 1 | 4 |
| hsa-miR-181a-5p | MIMAT0000256 | CA3 | NM_005181 | 1 | 1 | 0 | 1 | 1 | 4 |
| hsa-miR-181a-5p | MIMAT0000256 | CACNB2 | NM_000724 | 1 | 1 | 0 | 1 | 1 | 4 |
| hsa-miR-181a-5p | MIMAT0000256 | CAMK2G | XM_005270195 | 1 | 1 | 0 | 1 | 1 | 4 |
| hsa-miR-181a-5p | MIMAT0000256 | RUNX1T1 | NM_001198625 | 1 | 1 | 0 | 1 | 1 | 4 |
| hsa-miR-181a-5p | MIMAT0000256 | CBLB | XM_005247854 | 1 | 1 | 0 | 1 | 1 | 4 |
| hsa-miR-181a-5p | MIMAT0000256 | KRIT1 | NM_194455 | 1 | 1 | 1 | 0 | 1 | 4 |
| hsa-miR-181a-5p | MIMAT0000256 | CCNG1 | NM_004060 | 0 | 1 | 1 | 1 | 1 | 4 |
| hsa-miR-181a-5p | MIMAT0000256 | CD4 | NM_001195014 | 0 | 1 | 1 | 1 | 1 | 4 |
| hsa-miR-181a-5p | MIMAT0000256 | CHD2 | XM_005254836 | 1 | 1 | 0 | 1 | 1 | 4 |
| hsa-miR-181a-5p | MIMAT0000256 | CLCN5 | NM_001127899 | 1 | 1 | 0 | 1 | 1 | 4 |
| hsa-miR-181a-5p | MIMAT0000256 | TPP1 | NM_000391 | 1 | 1 | 0 | 1 | 1 | 4 |
| hsa-miR-181a-5p | MIMAT0000256 | CNR1 | XM_005248651 | 1 | 1 | 0 | 1 | 1 | 4 |
| hsa-miR-181a-5p | MIMAT0000256 | COL4A1 | NM_001845 | 1 | 1 | 0 | 1 | 1 | 4 |
| hsa-miR-181a-5p | MIMAT0000256 | COL5A1 | NM_000093 | 1 | 1 | 0 | 1 | 1 | 4 |
| hsa-miR-181a-5p | MIMAT0000256 | COX11 | NM_004375 | 1 | 1 | 0 | 1 | 1 | 4 |
| hsa-miR-181a-5p | MIMAT0000256 | CREB1 | NM_134442 | 1 | 1 | 1 | 0 | 1 | 4 |
| hsa-miR-181a-5p | MIMAT0000256 | ATF2 | NM_001256090 | 1 | 1 | 0 | 1 | 1 | 4 |
| hsa-miR-181a-5p | MIMAT0000256 | CREBL2 | NM_001310 | 1 | 1 | 0 | 1 | 1 | 4 |
| hsa-miR-181a-5p | MIMAT0000256 | HAPLN1 | NM_001884 | 1 | 1 | 0 | 1 | 1 | 4 |
| hsa-miR-181a-5p | MIMAT0000256 | CSF2RB | XM_005261340 | 1 | 1 | 0 | 1 | 1 | 4 |
| hsa-miR-181a-5p | MIMAT0000256 | VCAN | NM_004385 | 1 | 1 | 1 | 0 | 1 | 4 |
| hsa-miR-181a-5p | MIMAT0000256 | CTNNA1 | NM_001903 | 1 | 1 | 1 | 0 | 1 | 4 |
| hsa-miR-181a-5p | MIMAT0000256 | CXADR | NM_001338 | 1 | 1 | 0 | 1 | 1 | 4 |
| hsa-miR-181a-5p | MIMAT0000256 | CYP7A1 | NM_000780 | 1 | 1 | 0 | 1 | 1 | 4 |
| hsa-miR-181a-5p | MIMAT0000256 | DGKB | XM_005249628 | 1 | 1 | 0 | 1 | 1 | 4 |
| hsa-miR-181a-5p | MIMAT0000256 | DDX3X | XM_005272592 | 1 | 1 | 0 | 1 | 1 | 4 |
| hsa-miR-181a-5p | MIMAT0000256 | TRDMT1 | NM_004412 | 1 | 1 | 1 | 0 | 1 | 4 |
| hsa-miR-181a-5p | MIMAT0000256 | DSC3 | NM_024423 | 1 | 1 | 1 | 0 | 1 | 4 |
| hsa-miR-181a-5p | MIMAT0000256 | DTNA | NM_032975 | 1 | 1 | 0 | 1 | 1 | 4 |
| hsa-miR-181a-5p | MIMAT0000256 | E2F5 | NM_001951 | 0 | 1 | 1 | 1 | 1 | 4 |
| hsa-miR-181a-5p | MIMAT0000256 | S1PR1 | NM_001400 | 1 | 1 | 1 | 0 | 1 | 4 |
| hsa-miR-181a-5p | MIMAT0000256 | EDNRA | NM_001957 | 1 | 1 | 0 | 1 | 1 | 4 |
| hsa-miR-181a-5p | MIMAT0000256 | EDNRB | NM_001201397 | 1 | 1 | 0 | 1 | 1 | 4 |
| hsa-miR-181a-5p | MIMAT0000256 | EIF4A2 | NM_001967 | 1 | 1 | 1 | 0 | 1 | 4 |
| hsa-miR-181a-5p | MIMAT0000256 | ELAVL4 | XM_005270581 | 1 | 1 | 0 | 1 | 1 | 4 |
| hsa-miR-181a-5p | MIMAT0000256 | EMP2 | NM_001424 | 1 | 1 | 0 | 1 | 1 | 4 |
| hsa-miR-181a-5p | MIMAT0000256 | EN2 | NM_001427 | 1 | 1 | 1 | 0 | 1 | 4 |
| hsa-miR-181a-5p | MIMAT0000256 | EPB41 | XM_005245753 | 1 | 1 | 0 | 1 | 1 | 4 |
| hsa-miR-181a-5p | MIMAT0000256 | EPHA4 | XM_005246374 | 1 | 1 | 0 | 1 | 1 | 4 |
| hsa-miR-181a-5p | MIMAT0000256 | EPHA5 | NM_004439 | 1 | 1 | 0 | 1 | 1 | 4 |
| hsa-miR-181a-5p | MIMAT0000256 | EPS8 | NM_004447 | 1 | 1 | 0 | 1 | 1 | 4 |
| hsa-miR-181a-5p | MIMAT0000256 | ETF1 | XM_005271922 | 1 | 1 | 0 | 1 | 1 | 4 |
| hsa-miR-181a-5p | MIMAT0000256 | ETS1 | NM_005238 | 1 | 1 | 0 | 1 | 1 | 4 |
| hsa-miR-181a-5p | MIMAT0000256 | F2R | NM_001992 | 0 | 1 | 1 | 1 | 1 | 4 |
| hsa-miR-181a-5p | MIMAT0000256 | KDSR | NM_002035 | 1 | 1 | 0 | 1 | 1 | 4 |
| hsa-miR-181a-5p | MIMAT0000256 | GABRA1 | NM_001127644 | 1 | 1 | 1 | 0 | 1 | 4 |
| hsa-miR-181a-5p | MIMAT0000256 | GALNT3 | XM_005246449 | 1 | 1 | 0 | 1 | 1 | 4 |
| hsa-miR-181a-5p | MIMAT0000256 | GATM | NM_001482 | 1 | 1 | 1 | 0 | 1 | 4 |
| hsa-miR-181a-5p | MIMAT0000256 | GFRA1 | NM_005264 | 1 | 1 | 0 | 1 | 1 | 4 |
| hsa-miR-181a-5p | MIMAT0000256 | B4GALT1 | NM_001497 | 0 | 1 | 1 | 1 | 1 | 4 |
| hsa-miR-181a-5p | MIMAT0000256 | GNS | XM_005268800 | 1 | 1 | 0 | 1 | 1 | 4 |
| hsa-miR-181a-5p | MIMAT0000256 | GOLGA1 | NM_002077 | 1 | 1 | 0 | 1 | 1 | 4 |
| hsa-miR-181a-5p | MIMAT0000256 | GOT2 | NM_002080 | 1 | 1 | 0 | 1 | 1 | 4 |
| hsa-miR-181a-5p | MIMAT0000256 | GP5 | NM_004488 | 1 | 1 | 1 | 0 | 1 | 4 |
| hsa-miR-181a-5p | MIMAT0000256 | GPR26 | NM_153442 | 1 | 1 | 1 | 0 | 1 | 4 |
| hsa-miR-181a-5p | MIMAT0000256 | GRB10 | XM_005271760 | 1 | 1 | 0 | 1 | 1 | 4 |
| hsa-miR-181a-5p | MIMAT0000256 | GRIK2 | NM_001166247 | 1 | 1 | 0 | 1 | 1 | 4 |
| hsa-miR-181a-5p | MIMAT0000256 | GRM5 | XM_005273956 | 1 | 1 | 0 | 1 | 1 | 4 |
| hsa-miR-181a-5p | MIMAT0000256 | GSK3B | NM_002093 | 1 | 1 | 0 | 1 | 1 | 4 |
| hsa-miR-181a-5p | MIMAT0000256 | GTF2H1 | NM_001142307 | 1 | 1 | 0 | 1 | 1 | 4 |
| hsa-miR-181a-5p | MIMAT0000256 | GYPA | NM_002099 | 1 | 1 | 1 | 0 | 1 | 4 |
| hsa-miR-181a-5p | MIMAT0000256 | UBE2K | NM_005339 | 1 | 1 | 0 | 1 | 1 | 4 |
| hsa-miR-181a-5p | MIMAT0000256 | HLF | NM_002126 | 1 | 1 | 1 | 0 | 1 | 4 |
| hsa-miR-181a-5p | MIMAT0000256 | HMGB2 | NM_002129 | 1 | 1 | 1 | 0 | 1 | 4 |
| hsa-miR-181a-5p | MIMAT0000256 | HOXA1 | NM_005522 | 0 | 1 | 1 | 1 | 1 | 4 |
| hsa-miR-181a-5p | MIMAT0000256 | HOXB5 | NM_002147 | 1 | 1 | 0 | 1 | 1 | 4 |
| hsa-miR-181a-5p | MIMAT0000256 | DNAJB1 | NM_006145 | 1 | 1 | 0 | 1 | 1 | 4 |
| hsa-miR-181a-5p | MIMAT0000256 | IDH1 | XM_005246521 | 1 | 1 | 0 | 1 | 1 | 4 |
| hsa-miR-181a-5p | MIMAT0000256 | IFNA4 | NM_021068 | 1 | 1 | 1 | 0 | 1 | 4 |
| hsa-miR-181a-5p | MIMAT0000256 | CYR61 | NM_001554 | 1 | 1 | 1 | 0 | 1 | 4 |
| hsa-miR-181a-5p | MIMAT0000256 | IL1A | NM_000575 | 1 | 1 | 1 | 0 | 1 | 4 |
| hsa-miR-181a-5p | MIMAT0000256 | IL1RAP | NM_001167928 | 1 | 1 | 0 | 1 | 1 | 4 |
| hsa-miR-181a-5p | MIMAT0000256 | IL2 | NM_000586 | 1 | 1 | 1 | 0 | 1 | 4 |
| hsa-miR-181a-5p | MIMAT0000256 | IL15 | NM_172175 | 1 | 1 | 0 | 1 | 1 | 4 |
| hsa-miR-181a-5p | MIMAT0000256 | INPP4A | NM_001134224 | 1 | 1 | 1 | 0 | 1 | 4 |
| hsa-miR-181a-5p | MIMAT0000256 | ITGB8 | NM_002214 | 1 | 1 | 1 | 0 | 1 | 4 |
| hsa-miR-181a-5p | MIMAT0000256 | ITPR2 | NM_002223 | 1 | 1 | 0 | 1 | 1 | 4 |
| hsa-miR-181a-5p | MIMAT0000256 | KCNA1 | NM_000217 | 1 | 1 | 0 | 1 | 1 | 4 |
| hsa-miR-181a-5p | MIMAT0000256 | KCNA4 | NM_002233 | 1 | 1 | 1 | 0 | 1 | 4 |
| hsa-miR-181a-5p | MIMAT0000256 | KCNA6 | XM_005253686 | 1 | 1 | 0 | 1 | 1 | 4 |
| hsa-miR-181a-5p | MIMAT0000256 | KCNJ2 | XM_005257332 | 1 | 1 | 0 | 1 | 1 | 4 |
| hsa-miR-181a-5p | MIMAT0000256 | KIF5A | XM_005268858 | 1 | 1 | 0 | 1 | 1 | 4 |
| hsa-miR-181a-5p | MIMAT0000256 | IPO5 | XM_005254051 | 1 | 1 | 0 | 1 | 1 | 4 |
| hsa-miR-181a-5p | MIMAT0000256 | ABLIM1 | XM_005269818 | 1 | 1 | 0 | 1 | 1 | 4 |
| hsa-miR-181a-5p | MIMAT0000256 | LPP | XM_005247445 | 1 | 1 | 0 | 1 | 1 | 4 |
| hsa-miR-181a-5p | MIMAT0000256 | LRP4 | NM_002334 | 1 | 1 | 0 | 1 | 1 | 4 |
| hsa-miR-181a-5p | MIMAT0000256 | LRP6 | NM_002336 | 1 | 1 | 0 | 1 | 1 | 4 |
| hsa-miR-181a-5p | MIMAT0000256 | SMAD7 | NM_005904 | 1 | 1 | 0 | 1 | 1 | 4 |
| hsa-miR-181a-5p | MIMAT0000256 | MAT2A | NM_005911 | 0 | 1 | 1 | 1 | 1 | 4 |
| hsa-miR-181a-5p | MIMAT0000256 | MATN3 | NM_002381 | 0 | 1 | 1 | 1 | 1 | 4 |
| hsa-miR-181a-5p | MIMAT0000256 | MBNL1 | NM_021038 | 0 | 1 | 1 | 1 | 1 | 4 |
| hsa-miR-181a-5p | MIMAT0000256 | ADAM11 | XM_005257373 | 1 | 1 | 0 | 1 | 1 | 4 |
| hsa-miR-181a-5p | MIMAT0000256 | MECP2 | XM_005274683 | 1 | 1 | 0 | 1 | 1 | 4 |
| hsa-miR-181a-5p | MIMAT0000256 | MAP3K3 | XM_005257376 | 1 | 1 | 0 | 1 | 1 | 4 |
| hsa-miR-181a-5p | MIMAT0000256 | MEST | NM_002402 | 1 | 1 | 0 | 1 | 1 | 4 |
| hsa-miR-181a-5p | MIMAT0000256 | MET | XM_005250353 | 1 | 1 | 0 | 1 | 1 | 4 |
| hsa-miR-181a-5p | MIMAT0000256 | MLF1 | NM_001195432 | 1 | 1 | 0 | 1 | 1 | 4 |
| hsa-miR-181a-5p | MIMAT0000256 | MAP3K10 | NM_002446 | 1 | 1 | 0 | 1 | 1 | 4 |
| hsa-miR-181a-5p | MIMAT0000256 | MMP14 | NM_004995 | 1 | 1 | 0 | 1 | 1 | 4 |
| hsa-miR-181a-5p | MIMAT0000256 | MUC7 | NM_001145006 | 1 | 1 | 1 | 0 | 1 | 4 |
| hsa-miR-181a-5p | MIMAT0000256 | MYBL1 | NM_001080416 | 1 | 1 | 0 | 1 | 1 | 4 |
| hsa-miR-181a-5p | MIMAT0000256 | MYH10 | XM_005256651 | 1 | 1 | 0 | 1 | 1 | 4 |
| hsa-miR-181a-5p | MIMAT0000256 | MYO1E | NM_004998 | 1 | 1 | 1 | 0 | 1 | 4 |
| hsa-miR-181a-5p | MIMAT0000256 | MYO9A | XM_005254404 | 1 | 1 | 0 | 1 | 1 | 4 |
| hsa-miR-181a-5p | MIMAT0000256 | PPP1R12B | XM_005245201 | 1 | 1 | 0 | 1 | 1 | 4 |
| hsa-miR-181a-5p | MIMAT0000256 | NEO1 | XM_005254408 | 1 | 1 | 0 | 1 | 1 | 4 |
| hsa-miR-181a-5p | MIMAT0000256 | NF1 | NM_001042492 | 1 | 1 | 0 | 1 | 1 | 4 |
| hsa-miR-181a-5p | MIMAT0000256 | NFATC2 | NM_012340 | 1 | 1 | 0 | 1 | 1 | 4 |
| hsa-miR-181a-5p | MIMAT0000256 | NFIB | XM_005251467 | 1 | 1 | 0 | 1 | 1 | 4 |
| hsa-miR-181a-5p | MIMAT0000256 | NOVA1 | XM_005267707 | 1 | 1 | 0 | 1 | 1 | 4 |
| hsa-miR-181a-5p | MIMAT0000256 | NRCAM | XM_005250373 | 1 | 1 | 0 | 1 | 1 | 4 |
| hsa-miR-181a-5p | MIMAT0000256 | NRF1 | XM_005250387 | 1 | 1 | 0 | 1 | 1 | 4 |
| hsa-miR-181a-5p | MIMAT0000256 | NTS | NM_006183 | 1 | 1 | 1 | 0 | 1 | 4 |
| hsa-miR-181a-5p | MIMAT0000256 | TNFRSF11B | NM_002546 | 1 | 1 | 1 | 0 | 1 | 4 |
| hsa-miR-181a-5p | MIMAT0000256 | PAWR | NM_002583 | 1 | 1 | 1 | 0 | 1 | 4 |
| hsa-miR-181a-5p | MIMAT0000256 | PAX9 | NM_006194 | 1 | 1 | 1 | 0 | 1 | 4 |
| hsa-miR-181a-5p | MIMAT0000256 | PBX1 | XM_005245228 | 1 | 1 | 0 | 1 | 1 | 4 |
| hsa-miR-181a-5p | MIMAT0000256 | PCSK1 | NM_000439 | 1 | 1 | 1 | 0 | 1 | 4 |
| hsa-miR-181a-5p | MIMAT0000256 | PDE1A | NM_005019 | 1 | 1 | 0 | 1 | 1 | 4 |
| hsa-miR-181a-5p | MIMAT0000256 | PDE1C | XM_005249767 | 1 | 1 | 0 | 1 | 1 | 4 |
| hsa-miR-181a-5p | MIMAT0000256 | PDE3A | XM_005253389 | 1 | 1 | 0 | 1 | 1 | 4 |
| hsa-miR-181a-5p | MIMAT0000256 | PDGFRA | NM_006206 | 1 | 1 | 1 | 0 | 1 | 4 |
| hsa-miR-181a-5p | MIMAT0000256 | PDK4 | NM_002612 | 1 | 1 | 0 | 1 | 1 | 4 |
| hsa-miR-181a-5p | MIMAT0000256 | PDPK1 | XM_005255356 | 1 | 1 | 0 | 1 | 1 | 4 |
| hsa-miR-181a-5p | MIMAT0000256 | PEG3 | NM_006210 | 1 | 1 | 0 | 1 | 1 | 4 |
| hsa-miR-181a-5p | MIMAT0000256 | PEX13 | NM_002618 | 1 | 1 | 1 | 0 | 1 | 4 |
| hsa-miR-181a-5p | MIMAT0000256 | ATP8B1 | NM_005603 | 1 | 1 | 0 | 1 | 1 | 4 |
| hsa-miR-181a-5p | MIMAT0000256 | PHKA1 | NM_002637 | 1 | 1 | 0 | 1 | 1 | 4 |
| hsa-miR-181a-5p | MIMAT0000256 | PIK3C2A | XM_005252978 | 1 | 1 | 0 | 1 | 1 | 4 |
| hsa-miR-181a-5p | MIMAT0000256 | PKD2 | NM_000297 | 1 | 1 | 0 | 1 | 1 | 4 |
| hsa-miR-181a-5p | MIMAT0000256 | PKP2 | NM_004572 | 1 | 1 | 1 | 0 | 1 | 4 |
| hsa-miR-181a-5p | MIMAT0000256 | PLA2G4A | NM_024420 | 1 | 1 | 0 | 1 | 1 | 4 |
| hsa-miR-181a-5p | MIMAT0000256 | PLD1 | NM_002662 | 1 | 1 | 1 | 0 | 1 | 4 |
| hsa-miR-181a-5p | MIMAT0000256 | PLEK | NM_002664 | 1 | 1 | 0 | 1 | 1 | 4 |
| hsa-miR-181a-5p | MIMAT0000256 | PLS1 | NM_001172312 | 1 | 1 | 0 | 1 | 1 | 4 |
| hsa-miR-181a-5p | MIMAT0000256 | PMAIP1 | XM_005266710 | 1 | 1 | 0 | 1 | 1 | 4 |
| hsa-miR-181a-5p | MIMAT0000256 | PML | NM_033238 | 1 | 1 | 0 | 1 | 1 | 4 |
| hsa-miR-181a-5p | MIMAT0000256 | POLA1 | XM_005274552 | 1 | 1 | 0 | 1 | 1 | 4 |
| hsa-miR-181a-5p | MIMAT0000256 | PPP1CB | NM_002709 | 1 | 1 | 0 | 1 | 1 | 4 |
| hsa-miR-181a-5p | MIMAT0000256 | PPP1R2 | NM_006241 | 1 | 1 | 0 | 1 | 1 | 4 |
| hsa-miR-181a-5p | MIMAT0000256 | PPP1R3C | NM_005398 | 1 | 1 | 1 | 0 | 1 | 4 |
| hsa-miR-181a-5p | MIMAT0000256 | PPP6C | NM_001123355 | 1 | 1 | 0 | 1 | 1 | 4 |
| hsa-miR-181a-5p | MIMAT0000256 | SRGN | NM_002727 | 1 | 1 | 0 | 1 | 1 | 4 |
| hsa-miR-181a-5p | MIMAT0000256 | PRKAA2 | NM_006252 | 1 | 1 | 0 | 1 | 1 | 4 |
| hsa-miR-181a-5p | MIMAT0000256 | PRKCD | NM_006254 | 1 | 1 | 1 | 0 | 1 | 4 |
| hsa-miR-181a-5p | MIMAT0000256 | PRKCE | XM_005264429 | 1 | 1 | 0 | 1 | 1 | 4 |
| hsa-miR-181a-5p | MIMAT0000256 | MAP2K1 | NM_002755 | 1 | 1 | 1 | 0 | 1 | 4 |
| hsa-miR-181a-5p | MIMAT0000256 | PRKX | NM_005044 | 1 | 1 | 0 | 1 | 1 | 4 |
| hsa-miR-181a-5p | MIMAT0000256 | PROX1 | NM_001270616 | 1 | 1 | 0 | 1 | 1 | 4 |
| hsa-miR-181a-5p | MIMAT0000256 | PRRG1 | NM_001173489 | 1 | 1 | 0 | 1 | 1 | 4 |
| hsa-miR-181a-5p | MIMAT0000256 | PSAP | NM_001042465 | 1 | 1 | 0 | 1 | 1 | 4 |
| hsa-miR-181a-5p | MIMAT0000256 | PTGS2 | NM_000963 | 1 | 1 | 1 | 0 | 1 | 4 |
| hsa-miR-181a-5p | MIMAT0000256 | PTPN4 | NM_002830 | 1 | 1 | 0 | 1 | 1 | 4 |
| hsa-miR-181a-5p | MIMAT0000256 | PTPRE | XM_005252692 | 1 | 1 | 0 | 1 | 1 | 4 |
| hsa-miR-181a-5p | MIMAT0000256 | PURB | NM_033224 | 1 | 1 | 0 | 1 | 1 | 4 |
| hsa-miR-181a-5p | MIMAT0000256 | RAD23B | NM_002874 | 1 | 1 | 0 | 1 | 1 | 4 |
| hsa-miR-181a-5p | MIMAT0000256 | RALA | NM_005402 | 1 | 1 | 0 | 1 | 1 | 4 |
| hsa-miR-181a-5p | MIMAT0000256 | RBBP7 | XM_005274572 | 1 | 1 | 0 | 1 | 1 | 4 |
| hsa-miR-181a-5p | MIMAT0000256 | RBMS1 | XM_005246738 | 1 | 1 | 0 | 1 | 1 | 4 |
| hsa-miR-181a-5p | MIMAT0000256 | RCN2 | NM_001271837 | 1 | 1 | 0 | 1 | 1 | 4 |
| hsa-miR-181a-5p | MIMAT0000256 | REST | NM_005612 | 1 | 1 | 0 | 1 | 1 | 4 |
| hsa-miR-181a-5p | MIMAT0000256 | RFX2 | NM_000635 | 1 | 1 | 0 | 1 | 1 | 4 |
| hsa-miR-181a-5p | MIMAT0000256 | RLF | NM_012421 | 1 | 1 | 1 | 0 | 1 | 4 |
| hsa-miR-181a-5p | MIMAT0000256 | RPE65 | NM_000329 | 1 | 1 | 1 | 0 | 1 | 4 |
| hsa-miR-181a-5p | MIMAT0000256 | RPS6KA3 | XM_005274574 | 1 | 1 | 0 | 1 | 1 | 4 |
| hsa-miR-181a-5p | MIMAT0000256 | RPS6KB1 | NM_001272044 | 1 | 1 | 0 | 1 | 1 | 4 |
| hsa-miR-181a-5p | MIMAT0000256 | CLIP1 | XM_005253593 | 1 | 1 | 0 | 1 | 1 | 4 |
| hsa-miR-181a-5p | MIMAT0000256 | RYR3 | NM_001036 | 1 | 1 | 1 | 0 | 1 | 4 |
| hsa-miR-181a-5p | MIMAT0000256 | SELE | NM_000450 | 1 | 1 | 0 | 1 | 1 | 4 |
| hsa-miR-181a-5p | MIMAT0000256 | SFPQ | XM_005271111 | 1 | 1 | 0 | 1 | 1 | 4 |
| hsa-miR-181a-5p | MIMAT0000256 | ITSN1 | NM_003024 | 1 | 1 | 0 | 1 | 1 | 4 |
| hsa-miR-181a-5p | MIMAT0000256 | ST3GAL1 | XM_005251023 | 1 | 1 | 0 | 1 | 1 | 4 |
| hsa-miR-181a-5p | MIMAT0000256 | SIM1 | XM_005267100 | 1 | 1 | 0 | 1 | 1 | 4 |
| hsa-miR-181a-5p | MIMAT0000256 | SLA | NM_001045556 | 1 | 1 | 1 | 0 | 1 | 4 |
| hsa-miR-181a-5p | MIMAT0000256 | SLC2A1 | NM_006516 | 1 | 1 | 1 | 0 | 1 | 4 |
| hsa-miR-181a-5p | MIMAT0000256 | SLC18A2 | NM_003054 | 1 | 1 | 0 | 1 | 1 | 4 |
| hsa-miR-181a-5p | MIMAT0000256 | SLIT1 | NM_003061 | 1 | 1 | 0 | 1 | 1 | 4 |
| hsa-miR-181a-5p | MIMAT0000256 | HLTF | NM_003071 | 1 | 1 | 0 | 1 | 1 | 4 |
| hsa-miR-181a-5p | MIMAT0000256 | SMN2 | XM_005248578 | 1 | 1 | 0 | 1 | 1 | 4 |
| hsa-miR-181a-5p | MIMAT0000256 | SNAP25 | XM_005260809 | 1 | 1 | 0 | 1 | 1 | 4 |
| hsa-miR-181a-5p | MIMAT0000256 | SNX1 | NM_003099 | 1 | 1 | 0 | 1 | 1 | 4 |
| hsa-miR-181a-5p | MIMAT0000256 | SNTB2 | NM_006750 | 1 | 1 | 0 | 1 | 1 | 4 |
| hsa-miR-181a-5p | MIMAT0000256 | SOAT1 | NM_003101 | 1 | 1 | 0 | 1 | 1 | 4 |
| hsa-miR-181a-5p | MIMAT0000256 | SOS1 | XM_005264515 | 1 | 1 | 0 | 1 | 1 | 4 |
| hsa-miR-181a-5p | MIMAT0000256 | SP1 | NM_138473 | 1 | 1 | 0 | 1 | 1 | 4 |
| hsa-miR-181a-5p | MIMAT0000256 | SPOCK1 | NM_004598 | 1 | 1 | 1 | 0 | 1 | 4 |
| hsa-miR-181a-5p | MIMAT0000256 | SPP1 | NM_001251830 | 1 | 1 | 1 | 0 | 1 | 4 |
| hsa-miR-181a-5p | MIMAT0000256 | SPTBN1 | NM_003128 | 1 | 1 | 0 | 1 | 1 | 4 |
| hsa-miR-181a-5p | MIMAT0000256 | SSR1 | NM_003144 | 1 | 1 | 0 | 1 | 1 | 4 |
| hsa-miR-181a-5p | MIMAT0000256 | STAT3 | XM_005257614 | 1 | 1 | 0 | 1 | 1 | 4 |
| hsa-miR-181a-5p | MIMAT0000256 | HSPA13 | NM_006948 | 1 | 1 | 0 | 1 | 1 | 4 |
| hsa-miR-181a-5p | MIMAT0000256 | STK4 | XM_005260533 | 1 | 1 | 0 | 1 | 1 | 4 |
| hsa-miR-181a-5p | MIMAT0000256 | STRN | NM_003162 | 1 | 1 | 1 | 0 | 1 | 4 |
| hsa-miR-181a-5p | MIMAT0000256 | SULT1C2 | NM_176825 | 1 | 1 | 1 | 0 | 1 | 4 |
| hsa-miR-181a-5p | MIMAT0000256 | TEAD1 | NM_021961 | 1 | 1 | 0 | 1 | 1 | 4 |
| hsa-miR-181a-5p | MIMAT0000256 | TFAM | NM_003201 | 1 | 1 | 0 | 1 | 1 | 4 |
| hsa-miR-181a-5p | MIMAT0000256 | TGFBR3 | NM_003243 | 1 | 1 | 0 | 1 | 1 | 4 |
| hsa-miR-181a-5p | MIMAT0000256 | THRB | NM_001252634 | 1 | 1 | 0 | 1 | 1 | 4 |
| hsa-miR-181a-5p | MIMAT0000256 | SEC62 | NM_003262 | 1 | 1 | 0 | 1 | 1 | 4 |
| hsa-miR-181a-5p | MIMAT0000256 | TSPAN8 | NM_004616 | 1 | 1 | 1 | 0 | 1 | 4 |
| hsa-miR-181a-5p | MIMAT0000256 | TMPRSS2 | XM_005261041 | 1 | 1 | 0 | 1 | 1 | 4 |
| hsa-miR-181a-5p | MIMAT0000256 | TNF | NM_000594 | 1 | 1 | 1 | 0 | 1 | 4 |
| hsa-miR-181a-5p | MIMAT0000256 | TNS1 | XM_005246822 | 1 | 1 | 0 | 1 | 1 | 4 |
| hsa-miR-181a-5p | MIMAT0000256 | TPD52 | NM_001025252 | 1 | 1 | 0 | 1 | 1 | 4 |
| hsa-miR-181a-5p | MIMAT0000256 | TUB | NM_003320 | 1 | 1 | 0 | 1 | 1 | 4 |
| hsa-miR-181a-5p | MIMAT0000256 | UBE2A | NM_003336 | 1 | 1 | 1 | 0 | 1 | 4 |
| hsa-miR-181a-5p | MIMAT0000256 | UBE2B | NM_003337 | 1 | 1 | 1 | 0 | 1 | 4 |
| hsa-miR-181a-5p | MIMAT0000256 | UBP1 | NM_014517 | 1 | 1 | 1 | 0 | 1 | 4 |
| hsa-miR-181a-5p | MIMAT0000256 | UFD1L | XM_005261276 | 1 | 1 | 0 | 1 | 1 | 4 |
| hsa-miR-181a-5p | MIMAT0000256 | VBP1 | XM_005274729 | 1 | 1 | 0 | 1 | 1 | 4 |
| hsa-miR-181a-5p | MIMAT0000256 | VIPR2 | NM_003382 | 1 | 1 | 0 | 1 | 1 | 4 |
| hsa-miR-181a-5p | MIMAT0000256 | WHSC1 | XM_005248004 | 1 | 1 | 0 | 1 | 1 | 4 |
| hsa-miR-181a-5p | MIMAT0000256 | NELFA | NM_005663 | 1 | 1 | 1 | 0 | 1 | 4 |
| hsa-miR-181a-5p | MIMAT0000256 | ZFX | NM_001178085 | 1 | 1 | 0 | 1 | 1 | 4 |
| hsa-miR-181a-5p | MIMAT0000256 | ZIC3 | NM_003413 | 1 | 1 | 1 | 0 | 1 | 4 |
| hsa-miR-181a-5p | MIMAT0000256 | ZNF14 | NM_021030 | 1 | 1 | 1 | 0 | 1 | 4 |
| hsa-miR-181a-5p | MIMAT0000256 | ZNF33B | XM_005271825 | 1 | 1 | 0 | 1 | 1 | 4 |
| hsa-miR-181a-5p | MIMAT0000256 | ZNF148 | NM_021964 | 1 | 1 | 0 | 1 | 1 | 4 |
| hsa-miR-181a-5p | MIMAT0000256 | ZNF154 | NM_001085384 | 1 | 1 | 0 | 1 | 1 | 4 |
| hsa-miR-181a-5p | MIMAT0000256 | ZMYM2 | NM_003453 | 1 | 1 | 0 | 1 | 1 | 4 |
| hsa-miR-181a-5p | MIMAT0000256 | ZNF200 | NM_003454 | 1 | 1 | 0 | 1 | 1 | 4 |
| hsa-miR-181a-5p | MIMAT0000256 | ZNF207 | XM_005258028 | 1 | 1 | 0 | 1 | 1 | 4 |
| hsa-miR-181a-5p | MIMAT0000256 | ZNF229 | NM_014518 | 1 | 1 | 0 | 1 | 1 | 4 |
| hsa-miR-181a-5p | MIMAT0000256 | EVI5 | NM_005665 | 1 | 1 | 0 | 1 | 1 | 4 |
| hsa-miR-181a-5p | MIMAT0000256 | SLMAP | XM_005265458 | 1 | 1 | 0 | 1 | 1 | 4 |
| hsa-miR-181a-5p | MIMAT0000256 | REEP5 | NM_005669 | 1 | 1 | 0 | 1 | 1 | 4 |
| hsa-miR-181a-5p | MIMAT0000256 | DEK | NM_003472 | 1 | 1 | 0 | 1 | 1 | 4 |
| hsa-miR-181a-5p | MIMAT0000256 | FZD3 | NM_017412 | 1 | 1 | 1 | 0 | 1 | 4 |
| hsa-miR-181a-5p | MIMAT0000256 | TUSC3 | NM_006765 | 1 | 1 | 0 | 1 | 1 | 4 |
| hsa-miR-181a-5p | MIMAT0000256 | LHX3 | XM_005263410 | 1 | 1 | 0 | 1 | 1 | 4 |
| hsa-miR-181a-5p | MIMAT0000256 | MLLT10 | NM_004641 | 1 | 1 | 1 | 0 | 1 | 4 |
| hsa-miR-181a-5p | MIMAT0000256 | CCDC6 | NM_005436 | 1 | 1 | 0 | 1 | 1 | 4 |
| hsa-miR-181a-5p | MIMAT0000256 | ADAM12 | XM_005270188 | 1 | 1 | 0 | 1 | 1 | 4 |
| hsa-miR-181a-5p | MIMAT0000256 | HMGA2 | NM_003483 | 1 | 1 | 0 | 1 | 1 | 4 |
| hsa-miR-181a-5p | MIMAT0000256 | HDHD1 | NM_001135565 | 0 | 1 | 1 | 1 | 1 | 4 |
| hsa-miR-181a-5p | MIMAT0000256 | USP9Y | NM_004654 | 1 | 1 | 1 | 0 | 1 | 4 |
| hsa-miR-181a-5p | MIMAT0000256 | SNN | NM_003498 | 1 | 1 | 0 | 1 | 1 | 4 |
| hsa-miR-181a-5p | MIMAT0000256 | STX7 | NM_003569 | 1 | 1 | 1 | 0 | 1 | 4 |
| hsa-miR-181a-5p | MIMAT0000256 | RGS5 | NM_001254749 | 1 | 1 | 0 | 1 | 1 | 4 |
| hsa-miR-181a-5p | MIMAT0000256 | BHLHE40 | NM_003670 | 0 | 1 | 1 | 1 | 1 | 4 |
| hsa-miR-181a-5p | MIMAT0000256 | PRPF18 | NM_003675 | 1 | 1 | 0 | 1 | 1 | 4 |
| hsa-miR-181a-5p | MIMAT0000256 | PDXK | NM_003681 | 1 | 1 | 0 | 1 | 1 | 4 |
| hsa-miR-181a-5p | MIMAT0000256 | CASK | XM_005272685 | 1 | 1 | 0 | 1 | 1 | 4 |
| hsa-miR-181a-5p | MIMAT0000256 | TNFSF11 | NM_033012 | 1 | 1 | 0 | 1 | 1 | 4 |
| hsa-miR-181a-5p | MIMAT0000256 | KLF7 | NM_003709 | 1 | 1 | 0 | 1 | 1 | 4 |
| hsa-miR-181a-5p | MIMAT0000256 | STC2 | NM_003714 | 0 | 1 | 1 | 1 | 1 | 4 |
| hsa-miR-181a-5p | MIMAT0000256 | KCNK5 | NM_003740 | 1 | 1 | 0 | 1 | 1 | 4 |
| hsa-miR-181a-5p | MIMAT0000256 | TNKS | XM_005272399 | 1 | 1 | 0 | 1 | 1 | 4 |
| hsa-miR-181a-5p | MIMAT0000256 | CDS2 | NM_003818 | 1 | 1 | 0 | 1 | 1 | 4 |
| hsa-miR-181a-5p | MIMAT0000256 | TNFRSF11A | NM_003839 | 1 | 1 | 0 | 1 | 1 | 4 |
| hsa-miR-181a-5p | MIMAT0000256 | PER3 | XM_005263525 | 1 | 1 | 0 | 1 | 1 | 4 |
| hsa-miR-181a-5p | MIMAT0000256 | PER2 | NM_022817 | 1 | 1 | 0 | 1 | 1 | 4 |
| hsa-miR-181a-5p | MIMAT0000256 | PHOX2B | NM_003924 | 1 | 1 | 0 | 1 | 1 | 4 |
| hsa-miR-181a-5p | MIMAT0000256 | BTRC | NM_033637 | 1 | 1 | 0 | 1 | 1 | 4 |
| hsa-miR-181a-5p | MIMAT0000256 | WASL | NM_003941 | 1 | 1 | 1 | 0 | 1 | 4 |
| hsa-miR-181a-5p | MIMAT0000256 | SPAG9 | NM_001130528 | 1 | 1 | 0 | 1 | 1 | 4 |
| hsa-miR-181a-5p | MIMAT0000256 | SLC7A6 | NM_001076785 | 1 | 1 | 0 | 1 | 1 | 4 |
| hsa-miR-181a-5p | MIMAT0000256 | MTMR6 | NM_004685 | 1 | 1 | 0 | 1 | 1 | 4 |
| hsa-miR-181a-5p | MIMAT0000256 | LATS1 | NM_004690 | 1 | 1 | 0 | 1 | 1 | 4 |
| hsa-miR-181a-5p | MIMAT0000256 | SLC16A6 | NM_001174166 | 1 | 1 | 1 | 0 | 1 | 4 |
| hsa-miR-181a-5p | MIMAT0000256 | CBFA2T2 | NM_005093 | 1 | 1 | 0 | 1 | 1 | 4 |
| hsa-miR-181a-5p | MIMAT0000256 | MAP3K13 | NM_004721 | 1 | 1 | 0 | 1 | 1 | 4 |
| hsa-miR-181a-5p | MIMAT0000256 | REPS2 | NM_004726 | 1 | 1 | 1 | 0 | 1 | 4 |
| hsa-miR-181a-5p | MIMAT0000256 | SLC24A1 | NM_004727 | 0 | 1 | 1 | 1 | 1 | 4 |
| hsa-miR-181a-5p | MIMAT0000256 | DCLK1 | NM_004734 | 1 | 1 | 1 | 0 | 1 | 4 |
| hsa-miR-181a-5p | MIMAT0000256 | LRRFIP1 | XM_005246113 | 1 | 1 | 0 | 1 | 1 | 4 |
| hsa-miR-181a-5p | MIMAT0000256 | VAPB | NM_004738 | 1 | 1 | 0 | 1 | 1 | 4 |
| hsa-miR-181a-5p | MIMAT0000256 | LRAT | NM_004744 | 1 | 1 | 0 | 1 | 1 | 4 |
| hsa-miR-181a-5p | MIMAT0000256 | DLGAP2 | XM_005266038 | 1 | 1 | 0 | 1 | 1 | 4 |
| hsa-miR-181a-5p | MIMAT0000256 | DLGAP1 | XM_005258171 | 1 | 1 | 0 | 1 | 1 | 4 |
| hsa-miR-181a-5p | MIMAT0000256 | PNMA1 | NM_006029 | 0 | 1 | 1 | 1 | 1 | 4 |
| hsa-miR-181a-5p | MIMAT0000256 | B4GALT6 | XM_005258387 | 1 | 1 | 0 | 1 | 1 | 4 |
| hsa-miR-181a-5p | MIMAT0000256 | NRXN1 | NM_001135659 | 1 | 1 | 1 | 0 | 1 | 4 |
| hsa-miR-181a-5p | MIMAT0000256 | NMT2 | NM_004808 | 1 | 1 | 1 | 0 | 1 | 4 |
| hsa-miR-181a-5p | MIMAT0000256 | CDYL | NM_004824 | 0 | 1 | 1 | 1 | 1 | 4 |
| hsa-miR-181a-5p | MIMAT0000256 | RASAL2 | NM_170692 | 1 | 1 | 0 | 1 | 1 | 4 |
| hsa-miR-181a-5p | MIMAT0000256 | HAND2 | NM_021973 | 1 | 1 | 0 | 1 | 1 | 4 |
| hsa-miR-181a-5p | MIMAT0000256 | AKAP7 | NM_016377 | 1 | 1 | 1 | 0 | 1 | 4 |
| hsa-miR-181a-5p | MIMAT0000256 | AKAP6 | NM_004274 | 1 | 1 | 1 | 0 | 1 | 4 |
| hsa-miR-181a-5p | MIMAT0000256 | BAG4 | NM_004874 | 1 | 1 | 1 | 0 | 1 | 4 |
| hsa-miR-181a-5p | MIMAT0000256 | CLOCK | NM_001267843 | 1 | 1 | 0 | 1 | 1 | 4 |
| hsa-miR-181a-5p | MIMAT0000256 | ZNF592 | NM_014630 | 0 | 1 | 1 | 1 | 1 | 4 |
| hsa-miR-181a-5p | MIMAT0000256 | GCC2 | NM_181453 | 0 | 1 | 1 | 1 | 1 | 4 |
| hsa-miR-181a-5p | MIMAT0000256 | HS2ST1 | NM_012262 | 1 | 1 | 0 | 1 | 1 | 4 |
| hsa-miR-181a-5p | MIMAT0000256 | NOS1AP | NM_001126060 | 1 | 1 | 0 | 1 | 1 | 4 |
| hsa-miR-181a-5p | MIMAT0000256 | DOCK4 | XM_005250724 | 1 | 1 | 0 | 1 | 1 | 4 |
| hsa-miR-181a-5p | MIMAT0000256 | ARHGAP32 | NM_001142685 | 1 | 1 | 0 | 1 | 1 | 4 |
| hsa-miR-181a-5p | MIMAT0000256 | PCDHA9 | NM_014005 | 1 | 1 | 0 | 1 | 1 | 4 |
| hsa-miR-181a-5p | MIMAT0000256 | MLEC | NM_014730 | 1 | 1 | 0 | 1 | 1 | 4 |
| hsa-miR-181a-5p | MIMAT0000256 | RASSF2 | XM_005260895 | 1 | 1 | 0 | 1 | 1 | 4 |
| hsa-miR-181a-5p | MIMAT0000256 | BCLAF1 | XM_005267237 | 1 | 1 | 0 | 1 | 1 | 4 |
| hsa-miR-181a-5p | MIMAT0000256 | KIAA0232 | NM_014743 | 1 | 1 | 0 | 1 | 1 | 4 |
| hsa-miR-181a-5p | MIMAT0000256 | SERTAD2 | XM_005264669 | 1 | 1 | 0 | 1 | 1 | 4 |
| hsa-miR-181a-5p | MIMAT0000256 | GIT2 | NM_057169 | 1 | 1 | 0 | 1 | 1 | 4 |
| hsa-miR-181a-5p | MIMAT0000256 | MFAP3L | XM_005263365 | 1 | 1 | 0 | 1 | 1 | 4 |
| hsa-miR-181a-5p | MIMAT0000256 | PJA2 | NM_014819 | 1 | 1 | 0 | 1 | 1 | 4 |
| hsa-miR-181a-5p | MIMAT0000256 | TLK1 | NM_012290 | 1 | 1 | 0 | 1 | 1 | 4 |
| hsa-miR-181a-5p | MIMAT0000256 | ZC3H11A | XM_005245645 | 1 | 1 | 0 | 1 | 1 | 4 |
| hsa-miR-181a-5p | MIMAT0000256 | TBC1D4 | XM_005266602 | 1 | 1 | 0 | 1 | 1 | 4 |
| hsa-miR-181a-5p | MIMAT0000256 | ARNT2 | XM_005254811 | 1 | 1 | 0 | 1 | 1 | 4 |
| hsa-miR-181a-5p | MIMAT0000256 | KBTBD11 | NM_014867 | 1 | 1 | 0 | 1 | 1 | 4 |
| hsa-miR-181a-5p | MIMAT0000256 | LPGAT1 | XM_005273364 | 1 | 1 | 0 | 1 | 1 | 4 |
| hsa-miR-181a-5p | MIMAT0000256 | SLC23A2 | NM_203327 | 1 | 1 | 0 | 1 | 1 | 4 |
| hsa-miR-181a-5p | MIMAT0000256 | NR1D2 | NM_005126 | 1 | 1 | 1 | 0 | 1 | 4 |
| hsa-miR-181a-5p | MIMAT0000256 | AKT3 | XM_005272995 | 1 | 1 | 0 | 1 | 1 | 4 |
| hsa-miR-181a-5p | MIMAT0000256 | KCNE3 | NM_005472 | 1 | 1 | 0 | 1 | 1 | 4 |
| hsa-miR-181a-5p | MIMAT0000256 | ZBTB33 | NM_001184742 | 1 | 1 | 0 | 1 | 1 | 4 |
| hsa-miR-181a-5p | MIMAT0000256 | SUGP2 | XM_005259704 | 1 | 1 | 0 | 1 | 1 | 4 |
| hsa-miR-181a-5p | MIMAT0000256 | GPR64 | NM_001079858 | 1 | 1 | 0 | 1 | 1 | 4 |
| hsa-miR-181a-5p | MIMAT0000256 | DHRS9 | NM_005771 | 1 | 1 | 0 | 1 | 1 | 4 |
| hsa-miR-181a-5p | MIMAT0000256 | TRIM13 | NM_213590 | 1 | 1 | 0 | 1 | 1 | 4 |
| hsa-miR-181a-5p | MIMAT0000256 | INADL | NM_176877 | 1 | 1 | 0 | 1 | 1 | 4 |
| hsa-miR-181a-5p | MIMAT0000256 | AP3S2 | NM_005829 | 1 | 1 | 0 | 1 | 1 | 4 |
| hsa-miR-181a-5p | MIMAT0000256 | KCNMB2 | NM_181361 | 1 | 1 | 1 | 0 | 1 | 4 |
| hsa-miR-181a-5p | MIMAT0000256 | NET1 | NM_001047160 | 1 | 1 | 0 | 1 | 1 | 4 |
| hsa-miR-181a-5p | MIMAT0000256 | ATP8A1 | NM_006095 | 1 | 1 | 0 | 1 | 1 | 4 |
| hsa-miR-181a-5p | MIMAT0000256 | FAM3C | NM_014888 | 1 | 1 | 1 | 0 | 1 | 4 |
| hsa-miR-181a-5p | MIMAT0000256 | MERTK | NM_006343 | 1 | 1 | 0 | 1 | 1 | 4 |
| hsa-miR-181a-5p | MIMAT0000256 | SLC9A6 | NM_001042537 | 0 | 1 | 1 | 1 | 1 | 4 |
| hsa-miR-181a-5p | MIMAT0000256 | SYNCRIP | NM_001159677 | 1 | 1 | 0 | 1 | 1 | 4 |
| hsa-miR-181a-5p | MIMAT0000256 | ENOX2 | NM_182314 | 1 | 1 | 0 | 1 | 1 | 4 |
| hsa-miR-181a-5p | MIMAT0000256 | NEBL | NM_006393 | 1 | 1 | 0 | 1 | 1 | 4 |
| hsa-miR-181a-5p | MIMAT0000256 | ARFGEF2 | XM_005260252 | 1 | 1 | 0 | 1 | 1 | 4 |
| hsa-miR-181a-5p | MIMAT0000256 | PDLIM5 | XM_005262693 | 1 | 1 | 0 | 1 | 1 | 4 |
| hsa-miR-181a-5p | MIMAT0000256 | POLR3G | XM_005248405 | 1 | 1 | 0 | 1 | 1 | 4 |
| hsa-miR-181a-5p | MIMAT0000256 | EXOC5 | NM_006544 | 1 | 1 | 1 | 0 | 1 | 4 |
| hsa-miR-181a-5p | MIMAT0000256 | NFAT5 | XM_005255777 | 1 | 1 | 0 | 1 | 1 | 4 |
| hsa-miR-181a-5p | MIMAT0000256 | MAP3K2 | NM_006609 | 1 | 1 | 0 | 1 | 1 | 4 |
| hsa-miR-181a-5p | MIMAT0000256 | NUP50 | XM_005261312 | 1 | 1 | 0 | 1 | 1 | 4 |
| hsa-miR-181a-5p | MIMAT0000256 | ZMYND11 | NM_006624 | 1 | 1 | 1 | 0 | 1 | 4 |
| hsa-miR-181a-5p | MIMAT0000256 | ZNF266 | NM_006631 | 0 | 1 | 1 | 1 | 1 | 4 |
| hsa-miR-181a-5p | MIMAT0000256 | SEC24A | NM_021982 | 1 | 1 | 1 | 0 | 1 | 4 |
| hsa-miR-181a-5p | MIMAT0000256 | GPR83 | NM_016540 | 1 | 1 | 1 | 0 | 1 | 4 |
| hsa-miR-181a-5p | MIMAT0000256 | MALT1 | NM_006785 | 1 | 1 | 0 | 1 | 1 | 4 |
| hsa-miR-181a-5p | MIMAT0000256 | SPIN1 | XM_005251669 | 1 | 1 | 0 | 1 | 1 | 4 |
| hsa-miR-181a-5p | MIMAT0000256 | CPSF6 | XM_005268588 | 1 | 1 | 0 | 1 | 1 | 4 |
| hsa-miR-181a-5p | MIMAT0000256 | RAPGEF4 | NM_007023 | 1 | 1 | 1 | 0 | 1 | 4 |
| hsa-miR-181a-5p | MIMAT0000256 | PRDM5 | XM_005262706 | 1 | 1 | 0 | 1 | 1 | 4 |
| hsa-miR-181a-5p | MIMAT0000256 | PRDM4 | NM_012406 | 0 | 1 | 1 | 1 | 1 | 4 |
| hsa-miR-181a-5p | MIMAT0000256 | PTPRT | NM_133170 | 1 | 1 | 0 | 1 | 1 | 4 |
| hsa-miR-181a-5p | MIMAT0000256 | ERLIN2 | XM_005273391 | 1 | 1 | 0 | 1 | 1 | 4 |
| hsa-miR-181a-5p | MIMAT0000256 | ADAMTS6 | NM_197941 | 1 | 1 | 0 | 1 | 1 | 4 |
| hsa-miR-181a-5p | MIMAT0000256 | ZNF277 | NM_021994 | 1 | 1 | 1 | 0 | 1 | 4 |
| hsa-miR-181a-5p | MIMAT0000256 | WIF1 | NM_007191 | 1 | 1 | 1 | 0 | 1 | 4 |
| hsa-miR-181a-5p | MIMAT0000256 | RNF24 | NM_007219 | 1 | 1 | 0 | 1 | 1 | 4 |
| hsa-miR-181a-5p | MIMAT0000256 | SYNRG | XM_005256980 | 1 | 1 | 0 | 1 | 1 | 4 |
| hsa-miR-181a-5p | MIMAT0000256 | AAK1 | NM_014911 | 1 | 1 | 1 | 0 | 1 | 4 |
| hsa-miR-181a-5p | MIMAT0000256 | LMTK2 | NM_014916 | 1 | 1 | 0 | 1 | 1 | 4 |
| hsa-miR-181a-5p | MIMAT0000256 | FNDC3A | NM_001278438 | 1 | 1 | 0 | 1 | 1 | 4 |
| hsa-miR-181a-5p | MIMAT0000256 | DIS3 | NM_014953 | 1 | 1 | 0 | 1 | 1 | 4 |
| hsa-miR-181a-5p | MIMAT0000256 | DIP2C | XM_005252428 | 1 | 1 | 0 | 1 | 1 | 4 |
| hsa-miR-181a-5p | MIMAT0000256 | PCNX | NM_014982 | 1 | 1 | 0 | 1 | 1 | 4 |
| hsa-miR-181a-5p | MIMAT0000256 | SAMD4A | XM_005267432 | 1 | 1 | 0 | 1 | 1 | 4 |
| hsa-miR-181a-5p | MIMAT0000256 | PHLPP2 | XM_005255852 | 1 | 1 | 0 | 1 | 1 | 4 |
| hsa-miR-181a-5p | MIMAT0000256 | XPO7 | NM_015024 | 0 | 1 | 1 | 1 | 1 | 4 |
| hsa-miR-181a-5p | MIMAT0000256 | SETX | XM_005272173 | 1 | 1 | 0 | 1 | 1 | 4 |
| hsa-miR-181a-5p | MIMAT0000256 | TRIM35 | NM_171982 | 1 | 1 | 0 | 1 | 1 | 4 |
| hsa-miR-181a-5p | MIMAT0000256 | ARHGAP26 | XM_005268398 | 1 | 1 | 0 | 1 | 1 | 4 |
| hsa-miR-181a-5p | MIMAT0000256 | ZBTB43 | XM_005251835 | 1 | 1 | 0 | 1 | 1 | 4 |
| hsa-miR-181a-5p | MIMAT0000256 | MRPS27 | NM_015084 | 1 | 1 | 0 | 1 | 1 | 4 |
| hsa-miR-181a-5p | MIMAT0000256 | TNRC6B | XM_005261393 | 1 | 1 | 0 | 1 | 1 | 4 |
| hsa-miR-181a-5p | MIMAT0000256 | HIC2 | XM_005261395 | 1 | 1 | 0 | 1 | 1 | 4 |
| hsa-miR-181a-5p | MIMAT0000256 | TBC1D9 | NM_015130 | 1 | 1 | 0 | 1 | 1 | 4 |
| hsa-miR-181a-5p | MIMAT0000256 | RTF1 | NM_015138 | 1 | 1 | 0 | 1 | 1 | 4 |
| hsa-miR-181a-5p | MIMAT0000256 | METAP1 | XM_005262868 | 1 | 1 | 0 | 1 | 1 | 4 |
| hsa-miR-181a-5p | MIMAT0000256 | GSE1 | XM_005255859 | 1 | 1 | 0 | 1 | 1 | 4 |
| hsa-miR-181a-5p | MIMAT0000256 | TBC1D1 | XM_005262646 | 1 | 1 | 0 | 1 | 1 | 4 |
| hsa-miR-181a-5p | MIMAT0000256 | FBXO28 | NM_015176 | 1 | 1 | 0 | 1 | 1 | 4 |
| hsa-miR-181a-5p | MIMAT0000256 | DTX4 | NM_015177 | 1 | 1 | 0 | 1 | 1 | 4 |
| hsa-miR-181a-5p | MIMAT0000256 | ATP11A | XM_005268305 | 1 | 1 | 0 | 1 | 1 | 4 |
| hsa-miR-181a-5p | MIMAT0000256 | PPIP5K2 | NM_001281471 | 1 | 1 | 0 | 1 | 1 | 4 |
| hsa-miR-181a-5p | MIMAT0000256 | TSPYL4 | NM_021648 | 0 | 1 | 1 | 1 | 1 | 4 |
| hsa-miR-181a-5p | MIMAT0000256 | WSCD1 | XM_005256572 | 1 | 1 | 0 | 1 | 1 | 4 |
| hsa-miR-181a-5p | MIMAT0000256 | TMEM194A | NM_001130963 | 1 | 1 | 1 | 0 | 1 | 4 |
| hsa-miR-181a-5p | MIMAT0000256 | DMXL2 | XM_005254255 | 1 | 1 | 0 | 1 | 1 | 4 |
| hsa-miR-181a-5p | MIMAT0000256 | SLC9A8 | NM_001260491 | 1 | 1 | 0 | 1 | 1 | 4 |
| hsa-miR-181a-5p | MIMAT0000256 | TRIM2 | NM_001130067 | 1 | 1 | 1 | 0 | 1 | 4 |
| hsa-miR-181a-5p | MIMAT0000256 | SASH1 | XM_005266876 | 1 | 1 | 0 | 1 | 1 | 4 |
| hsa-miR-181a-5p | MIMAT0000256 | TBC1D30 | NM_015279 | 1 | 1 | 0 | 1 | 1 | 4 |
| hsa-miR-181a-5p | MIMAT0000256 | CLASP1 | NM_015282 | 0 | 1 | 1 | 1 | 1 | 4 |
| hsa-miR-181a-5p | MIMAT0000256 | DPY19L1 | NM_015283 | 0 | 1 | 1 | 1 | 1 | 4 |
| hsa-miR-181a-5p | MIMAT0000256 | PHF15 | XM_005271948 | 1 | 1 | 0 | 1 | 1 | 4 |
| hsa-miR-181a-5p | MIMAT0000256 | PSD3 | NM_015310 | 1 | 1 | 0 | 1 | 1 | 4 |
| hsa-miR-181a-5p | MIMAT0000256 | EXOSC2 | NM_014285 | 1 | 1 | 0 | 1 | 1 | 4 |
| hsa-miR-181a-5p | MIMAT0000256 | TARDBP | NM_007375 | 1 | 1 | 1 | 0 | 1 | 4 |
| hsa-miR-181a-5p | MIMAT0000256 | ICMT | NM_012405 | 1 | 1 | 0 | 1 | 1 | 4 |
| hsa-miR-181a-5p | MIMAT0000256 | CBX5 | NM_001127322 | 1 | 1 | 0 | 1 | 1 | 4 |
| hsa-miR-181a-5p | MIMAT0000256 | POFUT1 | NM_015352 | 1 | 1 | 0 | 1 | 1 | 4 |
| hsa-miR-181a-5p | MIMAT0000256 | SLC39A14 | XM_005273463 | 1 | 1 | 0 | 1 | 1 | 4 |
| hsa-miR-181a-5p | MIMAT0000256 | CORO1C | NM_001276471 | 1 | 1 | 0 | 1 | 1 | 4 |
| hsa-miR-181a-5p | MIMAT0000256 | STX12 | NM_177424 | 1 | 1 | 0 | 1 | 1 | 4 |
| hsa-miR-181a-5p | MIMAT0000256 | RAB38 | XM_005273860 | 1 | 1 | 0 | 1 | 1 | 4 |
| hsa-miR-181a-5p | MIMAT0000256 | PITPNB | NM_012399 | 0 | 1 | 1 | 1 | 1 | 4 |
| hsa-miR-181a-5p | MIMAT0000256 | BRD1 | XM_005261470 | 1 | 1 | 0 | 1 | 1 | 4 |
| hsa-miR-181a-5p | MIMAT0000256 | ARIH1 | NM_005744 | 1 | 1 | 0 | 1 | 1 | 4 |
| hsa-miR-181a-5p | MIMAT0000256 | ARMC8 | NM_015396 | 1 | 1 | 0 | 1 | 1 | 4 |
| hsa-miR-181a-5p | MIMAT0000256 | RNF19A | NM_183419 | 0 | 1 | 1 | 1 | 1 | 4 |
| hsa-miR-181a-5p | MIMAT0000256 | C1orf43 | NM_001098616 | 1 | 1 | 0 | 1 | 1 | 4 |
| hsa-miR-181a-5p | MIMAT0000256 | C2CD2 | NM_015500 | 1 | 1 | 0 | 1 | 1 | 4 |
| hsa-miR-181a-5p | MIMAT0000256 | ZZZ3 | XM_005270725 | 1 | 1 | 0 | 1 | 1 | 4 |
| hsa-miR-181a-5p | MIMAT0000256 | SUSD5 | NM_015551 | 1 | 1 | 0 | 1 | 1 | 4 |
| hsa-miR-181a-5p | MIMAT0000256 | ZNF451 | NM_001257273 | 1 | 1 | 0 | 1 | 1 | 4 |
| hsa-miR-181a-5p | MIMAT0000256 | SIPA1L1 | XM_005267516 | 1 | 1 | 0 | 1 | 1 | 4 |
| hsa-miR-181a-5p | MIMAT0000256 | SS18L1 | XM_005260391 | 1 | 1 | 0 | 1 | 1 | 4 |
| hsa-miR-181a-5p | MIMAT0000256 | AUTS2 | NM_015570 | 1 | 1 | 0 | 1 | 1 | 4 |
| hsa-miR-181a-5p | MIMAT0000256 | RAI14 | NM_001145525 | 1 | 1 | 0 | 1 | 1 | 4 |
| hsa-miR-181a-5p | MIMAT0000256 | WSB1 | XM_005257962 | 1 | 1 | 0 | 1 | 1 | 4 |
| hsa-miR-181a-5p | MIMAT0000256 | TCTN3 | NM_015631 | 1 | 1 | 0 | 1 | 1 | 4 |
| hsa-miR-181a-5p | MIMAT0000256 | GAPVD1 | XM_005251899 | 1 | 1 | 0 | 1 | 1 | 4 |
| hsa-miR-181a-5p | MIMAT0000256 | RGS22 | NM_015668 | 1 | 1 | 0 | 1 | 1 | 4 |
| hsa-miR-181a-5p | MIMAT0000256 | FBXO5 | NM_001142522 | 1 | 1 | 0 | 1 | 1 | 4 |
| hsa-miR-181a-5p | MIMAT0000256 | LHX6 | NM_001242335 | 1 | 1 | 0 | 1 | 1 | 4 |
| hsa-miR-181a-5p | MIMAT0000256 | GREM1 | XM_005254301 | 1 | 1 | 0 | 1 | 1 | 4 |
| hsa-miR-181a-5p | MIMAT0000256 | NPTN | XM_005254304 | 1 | 1 | 0 | 1 | 1 | 4 |
| hsa-miR-181a-5p | MIMAT0000256 | BBS9 | NM_198428 | 0 | 1 | 1 | 1 | 1 | 4 |
| hsa-miR-181a-5p | MIMAT0000256 | ZNF544 | XM_005258756 | 1 | 1 | 0 | 1 | 1 | 4 |
| hsa-miR-181a-5p | MIMAT0000256 | RBMS3 | XM_005265060 | 1 | 1 | 0 | 1 | 1 | 4 |
| hsa-miR-181a-5p | MIMAT0000256 | PCDH11X | NM_032968 | 1 | 1 | 0 | 1 | 1 | 4 |
| hsa-miR-181a-5p | MIMAT0000256 | RABGEF1 | XM_005250280 | 1 | 1 | 0 | 1 | 1 | 4 |
| hsa-miR-181a-5p | MIMAT0000256 | OSTM1 | NM_014028 | 1 | 1 | 0 | 1 | 1 | 4 |
| hsa-miR-181a-5p | MIMAT0000256 | FLVCR1 | NM_014053 | 1 | 1 | 1 | 0 | 1 | 4 |
| hsa-miR-181a-5p | MIMAT0000256 | HIPK2 | NM_022740 | 1 | 1 | 0 | 1 | 1 | 4 |
| hsa-miR-181a-5p | MIMAT0000256 | MRPL13 | NM_014078 | 1 | 1 | 1 | 0 | 1 | 4 |
| hsa-miR-181a-5p | MIMAT0000256 | NDUFAF4 | NM_014165 | 0 | 1 | 1 | 1 | 1 | 4 |
| hsa-miR-181a-5p | MIMAT0000256 | TFCP2L1 | NM_014553 | 1 | 1 | 0 | 1 | 1 | 4 |
| hsa-miR-181a-5p | MIMAT0000256 | SENP1 | NM_001267595 | 1 | 1 | 0 | 1 | 1 | 4 |
| hsa-miR-181a-5p | MIMAT0000256 | SLC25A24 | NM_013386 | 1 | 1 | 1 | 0 | 1 | 4 |
| hsa-miR-181a-5p | MIMAT0000256 | EMR2 | NM_013447 | 1 | 1 | 0 | 1 | 1 | 4 |
| hsa-miR-181a-5p | MIMAT0000256 | SCAPER | XM_005254418 | 1 | 1 | 0 | 1 | 1 | 4 |
| hsa-miR-181a-5p | MIMAT0000256 | CHST11 | NM_018413 | 1 | 1 | 0 | 1 | 1 | 4 |
| hsa-miR-181a-5p | MIMAT0000256 | F11R | NM_016946 | 1 | 1 | 0 | 1 | 1 | 4 |
| hsa-miR-181a-5p | MIMAT0000256 | SPOCK3 | NM_001204352 | 1 | 1 | 0 | 1 | 1 | 4 |
| hsa-miR-181a-5p | MIMAT0000256 | ST8SIA3 | NM_015879 | 1 | 1 | 0 | 1 | 1 | 4 |
| hsa-miR-181a-5p | MIMAT0000256 | FAM135B | NM_015912 | 1 | 1 | 0 | 1 | 1 | 4 |
| hsa-miR-181a-5p | MIMAT0000256 | KLHL5 | NM_015990 | 1 | 1 | 1 | 0 | 1 | 4 |
| hsa-miR-181a-5p | MIMAT0000256 | PHF20L1 | NM_016018 | 1 | 1 | 1 | 0 | 1 | 4 |
| hsa-miR-181a-5p | MIMAT0000256 | MYO15A | NM_016239 | 1 | 1 | 0 | 1 | 1 | 4 |
| hsa-miR-181a-5p | MIMAT0000256 | CRIM1 | NM_016441 | 1 | 1 | 0 | 1 | 1 | 4 |
| hsa-miR-181a-5p | MIMAT0000256 | MEX3C | NM_016626 | 1 | 1 | 0 | 1 | 1 | 4 |
| hsa-miR-181a-5p | MIMAT0000256 | SPG21 | XM_005254436 | 1 | 1 | 0 | 1 | 1 | 4 |
| hsa-miR-181a-5p | MIMAT0000256 | CRLF3 | NM_015986 | 1 | 1 | 0 | 1 | 1 | 4 |
| hsa-miR-181a-5p | MIMAT0000256 | PRKAG2 | XM_005250002 | 1 | 1 | 0 | 1 | 1 | 4 |
| hsa-miR-181a-5p | MIMAT0000256 | PCYOX1 | NM_016297 | 1 | 1 | 0 | 1 | 1 | 4 |
| hsa-miR-181a-5p | MIMAT0000256 | ANKFY1 | NM_001257999 | 1 | 1 | 0 | 1 | 1 | 4 |
| hsa-miR-181a-5p | MIMAT0000256 | GSKIP | NM_001271904 | 1 | 1 | 0 | 1 | 1 | 4 |
| hsa-miR-181a-5p | MIMAT0000256 | TAF9B | XM_005262142 | 1 | 1 | 0 | 1 | 1 | 4 |
| hsa-miR-181a-5p | MIMAT0000256 | ZFR | NM_016107 | 1 | 1 | 0 | 1 | 1 | 4 |
| hsa-miR-181a-5p | MIMAT0000256 | HECA | NM_016217 | 1 | 1 | 1 | 0 | 1 | 4 |
| hsa-miR-181a-5p | MIMAT0000256 | RSF1 | XM_005274051 | 1 | 1 | 0 | 1 | 1 | 4 |
| hsa-miR-181a-5p | MIMAT0000256 | SIX4 | NM_017420 | 1 | 1 | 0 | 1 | 1 | 4 |
| hsa-miR-181a-5p | MIMAT0000256 | ADAM22 | XM_005250444 | 1 | 1 | 0 | 1 | 1 | 4 |
| hsa-miR-181a-5p | MIMAT0000256 | GPR88 | NM_022049 | 0 | 1 | 1 | 1 | 1 | 4 |
| hsa-miR-181a-5p | MIMAT0000256 | KCNK10 | NM_138317 | 1 | 1 | 1 | 0 | 1 | 4 |
| hsa-miR-181a-5p | MIMAT0000256 | SLC38A2 | NM_018976 | 0 | 1 | 1 | 1 | 1 | 4 |
| hsa-miR-181a-5p | MIMAT0000256 | CCSER2 | NM_018999 | 1 | 1 | 0 | 1 | 1 | 4 |
| hsa-miR-181a-5p | MIMAT0000256 | XRN1 | NM_019001 | 0 | 1 | 1 | 1 | 1 | 4 |
| hsa-miR-181a-5p | MIMAT0000256 | MIEF1 | XM_005261651 | 1 | 1 | 0 | 1 | 1 | 4 |
| hsa-miR-181a-5p | MIMAT0000256 | RBM47 | NM_001098634 | 1 | 1 | 0 | 1 | 1 | 4 |
| hsa-miR-181a-5p | MIMAT0000256 | DDIT4 | NM_019058 | 0 | 1 | 1 | 1 | 1 | 4 |
| hsa-miR-181a-5p | MIMAT0000256 | MTMR12 | XM_005248313 | 1 | 1 | 0 | 1 | 1 | 4 |
| hsa-miR-181a-5p | MIMAT0000256 | EGLN1 | NM_022051 | 1 | 1 | 0 | 1 | 1 | 4 |
| hsa-miR-181a-5p | MIMAT0000256 | CCNJ | NM_001134375 | 1 | 1 | 1 | 0 | 1 | 4 |
| hsa-miR-181a-5p | MIMAT0000256 | TMEM106B | NM_018374 | 1 | 1 | 1 | 0 | 1 | 4 |
| hsa-miR-181a-5p | MIMAT0000256 | OTUD4 | XM_005263079 | 1 | 1 | 0 | 1 | 1 | 4 |
| hsa-miR-181a-5p | MIMAT0000256 | ZNF586 | NM_001204814 | 0 | 1 | 1 | 1 | 1 | 4 |
| hsa-miR-181a-5p | MIMAT0000256 | ZNF280D | XM_005254480 | 1 | 1 | 0 | 1 | 1 | 4 |
| hsa-miR-181a-5p | MIMAT0000256 | FAM46C | XM_005270960 | 1 | 1 | 0 | 1 | 1 | 4 |
| hsa-miR-181a-5p | MIMAT0000256 | DCAF16 | NM_017741 | 1 | 1 | 0 | 1 | 1 | 4 |
| hsa-miR-181a-5p | MIMAT0000256 | INO80D | XM_005246658 | 1 | 1 | 0 | 1 | 1 | 4 |
| hsa-miR-181a-5p | MIMAT0000256 | SEMA4C | XM_005263973 | 1 | 1 | 0 | 1 | 1 | 4 |
| hsa-miR-181a-5p | MIMAT0000256 | RNF125 | NM_017831 | 1 | 1 | 0 | 1 | 1 | 4 |
| hsa-miR-181a-5p | MIMAT0000256 | BANK1 | NM_017935 | 1 | 1 | 0 | 1 | 1 | 4 |
| hsa-miR-181a-5p | MIMAT0000256 | AIM1L | NM_001039775 | 1 | 1 | 1 | 0 | 1 | 4 |
| hsa-miR-181a-5p | MIMAT0000256 | SPATA6L | NM_001039395 | 1 | 1 | 0 | 1 | 1 | 4 |
| hsa-miR-181a-5p | MIMAT0000256 | WDYHV1 | NM_018024 | 0 | 1 | 1 | 1 | 1 | 4 |
| hsa-miR-181a-5p | MIMAT0000256 | MAGOHB | NM_018048 | 1 | 1 | 1 | 0 | 1 | 4 |
| hsa-miR-181a-5p | MIMAT0000256 | ARMC1 | XM_005251263 | 1 | 1 | 0 | 1 | 1 | 4 |
| hsa-miR-181a-5p | MIMAT0000256 | SHQ1 | NM_018130 | 1 | 1 | 1 | 0 | 1 | 4 |
| hsa-miR-181a-5p | MIMAT0000256 | MSL2 | NM_018133 | 1 | 1 | 0 | 1 | 1 | 4 |
| hsa-miR-181a-5p | MIMAT0000256 | SBNO1 | XM_005253573 | 1 | 1 | 0 | 1 | 1 | 4 |
| hsa-miR-181a-5p | MIMAT0000256 | UBA6 | NM_018227 | 1 | 1 | 0 | 1 | 1 | 4 |
| hsa-miR-181a-5p | MIMAT0000256 | ZNF654 | XM_005264762 | 1 | 1 | 0 | 1 | 1 | 4 |
| hsa-miR-181a-5p | MIMAT0000256 | UBE2W | NM_001001481 | 1 | 1 | 0 | 1 | 1 | 4 |
| hsa-miR-181a-5p | MIMAT0000256 | RFK | NM_018339 | 1 | 1 | 0 | 1 | 1 | 4 |
| hsa-miR-181a-5p | MIMAT0000256 | STRBP | NM_018387 | 1 | 1 | 0 | 1 | 1 | 4 |
| hsa-miR-181a-5p | MIMAT0000256 | YOD1 | NM_001276320 | 1 | 1 | 0 | 1 | 1 | 4 |
| hsa-miR-181a-5p | MIMAT0000256 | DOCK10 | NM_014689 | 1 | 1 | 1 | 0 | 1 | 4 |
| hsa-miR-181a-5p | MIMAT0000256 | ZDHHC7 | NM_001145548 | 1 | 1 | 0 | 1 | 1 | 4 |
| hsa-miR-181a-5p | MIMAT0000256 | RUFY2 | NM_017987 | 1 | 1 | 1 | 0 | 1 | 4 |
| hsa-miR-181a-5p | MIMAT0000256 | FAM178A | NM_018121 | 0 | 1 | 1 | 1 | 1 | 4 |
| hsa-miR-181a-5p | MIMAT0000256 | AGK | NM_018238 | 1 | 1 | 1 | 0 | 1 | 4 |
| hsa-miR-181a-5p | MIMAT0000256 | 11-Sep | NM_018243 | 1 | 1 | 0 | 1 | 1 | 4 |
| hsa-miR-181a-5p | MIMAT0000256 | TMEM30A | NM_018247 | 1 | 1 | 0 | 1 | 1 | 4 |
| hsa-miR-181a-5p | MIMAT0000256 | ZNF701 | NM_001172655 | 1 | 1 | 0 | 1 | 1 | 4 |
| hsa-miR-181a-5p | MIMAT0000256 | MCTP2 | XM_005254956 | 1 | 1 | 0 | 1 | 1 | 4 |
| hsa-miR-181a-5p | MIMAT0000256 | MBNL3 | XM_005262434 | 1 | 1 | 0 | 1 | 1 | 4 |
| hsa-miR-181a-5p | MIMAT0000256 | PAG1 | NM_018440 | 1 | 1 | 1 | 0 | 1 | 4 |
| hsa-miR-181a-5p | MIMAT0000256 | MYNN | NM_001185118 | 1 | 1 | 0 | 1 | 1 | 4 |
| hsa-miR-181a-5p | MIMAT0000256 | CTTNBP2NL | XM_005271034 | 1 | 1 | 0 | 1 | 1 | 4 |
| hsa-miR-181a-5p | MIMAT0000256 | 3-Sep | NM_019106 | 1 | 1 | 0 | 1 | 1 | 4 |
| hsa-miR-181a-5p | MIMAT0000256 | PCDHB3 | NM_018937 | 1 | 1 | 0 | 1 | 1 | 4 |
| hsa-miR-181a-5p | MIMAT0000256 | ANKH | NM_054027 | 1 | 1 | 0 | 1 | 1 | 4 |
| hsa-miR-181a-5p | MIMAT0000256 | KCNQ5 | NM_001160133 | 1 | 1 | 1 | 0 | 1 | 4 |
| hsa-miR-181a-5p | MIMAT0000256 | CYP26B1 | NM_019885 | 1 | 1 | 0 | 1 | 1 | 4 |
| hsa-miR-181a-5p | MIMAT0000256 | AGPAT3 | XM_005261159 | 1 | 1 | 0 | 1 | 1 | 4 |
| hsa-miR-181a-5p | MIMAT0000256 | C11orf30 | XM_005274106 | 1 | 1 | 0 | 1 | 1 | 4 |
| hsa-miR-181a-5p | MIMAT0000256 | STOX2 | XM_005263142 | 1 | 1 | 0 | 1 | 1 | 4 |
| hsa-miR-181a-5p | MIMAT0000256 | BBX | NM_001142568 | 1 | 1 | 0 | 1 | 1 | 4 |
| hsa-miR-181a-5p | MIMAT0000256 | ADCK3 | XM_005273202 | 1 | 1 | 0 | 1 | 1 | 4 |
| hsa-miR-181a-5p | MIMAT0000256 | PCNP | NM_020357 | 1 | 1 | 1 | 0 | 1 | 4 |
| hsa-miR-181a-5p | MIMAT0000256 | PARP11 | NM_020367 | 0 | 1 | 1 | 1 | 1 | 4 |
| hsa-miR-181a-5p | MIMAT0000256 | CHMP1B | NM_020412 | 1 | 1 | 1 | 0 | 1 | 4 |
| hsa-miR-181a-5p | MIMAT0000256 | PAK7 | XM_005260764 | 1 | 1 | 0 | 1 | 1 | 4 |
| hsa-miR-181a-5p | MIMAT0000256 | RALGAPB | NM_020336 | 1 | 1 | 1 | 0 | 1 | 4 |
| hsa-miR-181a-5p | MIMAT0000256 | SLC39A10 | XM_005246691 | 1 | 1 | 0 | 1 | 1 | 4 |
| hsa-miR-181a-5p | MIMAT0000256 | ZNF286A | XM_005256740 | 1 | 1 | 0 | 1 | 1 | 4 |
| hsa-miR-181a-5p | MIMAT0000256 | BIRC6 | XM_005264449 | 1 | 1 | 0 | 1 | 1 | 4 |
| hsa-miR-181a-5p | MIMAT0000256 | GALNT16 | NM_001168368 | 1 | 1 | 0 | 1 | 1 | 4 |
| hsa-miR-181a-5p | MIMAT0000256 | CNOT6 | XM_005265953 | 1 | 1 | 0 | 1 | 1 | 4 |
| hsa-miR-181a-5p | MIMAT0000256 | USP31 | NM_020718 | 0 | 1 | 1 | 1 | 1 | 4 |
| hsa-miR-181a-5p | MIMAT0000256 | HEG1 | XM_005247666 | 1 | 1 | 0 | 1 | 1 | 4 |
| hsa-miR-181a-5p | MIMAT0000256 | PCDH19 | NM_001184880 | 1 | 1 | 0 | 1 | 1 | 4 |
| hsa-miR-181a-5p | MIMAT0000256 | TBC1D14 | XM_005247985 | 1 | 1 | 0 | 1 | 1 | 4 |
| hsa-miR-181a-5p | MIMAT0000256 | ALPK3 | NM_020778 | 1 | 1 | 0 | 1 | 1 | 4 |
| hsa-miR-181a-5p | MIMAT0000256 | ZNF398 | NM_020781 | 1 | 1 | 0 | 1 | 1 | 4 |
| hsa-miR-181a-5p | MIMAT0000256 | WDFY1 | NM_020830 | 1 | 1 | 0 | 1 | 1 | 4 |
| hsa-miR-181a-5p | MIMAT0000256 | SLAIN2 | XM_005248121 | 1 | 1 | 0 | 1 | 1 | 4 |
| hsa-miR-181a-5p | MIMAT0000256 | DIP2B | NM_173602 | 0 | 1 | 1 | 1 | 1 | 4 |
| hsa-miR-181a-5p | MIMAT0000256 | SHROOM3 | NM_020859 | 1 | 1 | 0 | 1 | 1 | 4 |
| hsa-miR-181a-5p | MIMAT0000256 | LRRN1 | XM_005265351 | 1 | 1 | 0 | 1 | 1 | 4 |
| hsa-miR-181a-5p | MIMAT0000256 | KIAA1549 | NM_001164665 | 1 | 1 | 0 | 1 | 1 | 4 |
| hsa-miR-181a-5p | MIMAT0000256 | ZFP14 | NM_020917 | 1 | 1 | 1 | 0 | 1 | 4 |
| hsa-miR-181a-5p | MIMAT0000256 | GPAM | XM_005269998 | 1 | 1 | 0 | 1 | 1 | 4 |
| hsa-miR-181a-5p | MIMAT0000256 | DENND1A | XM_005252110 | 1 | 1 | 0 | 1 | 1 | 4 |
| hsa-miR-181a-5p | MIMAT0000256 | CREBZF | NM_001039618 | 1 | 1 | 1 | 0 | 1 | 4 |
| hsa-miR-181a-5p | MIMAT0000256 | DMRT3 | NM_021240 | 1 | 1 | 0 | 1 | 1 | 4 |
| hsa-miR-181a-5p | MIMAT0000256 | ENPP5 | XM_005249259 | 1 | 1 | 0 | 1 | 1 | 4 |
| hsa-miR-181a-5p | MIMAT0000256 | PLEKHA1 | XM_005270017 | 1 | 1 | 0 | 1 | 1 | 4 |
| hsa-miR-181a-5p | MIMAT0000256 | GNB4 | NM_021629 | 1 | 1 | 1 | 0 | 1 | 4 |
| hsa-miR-181a-5p | MIMAT0000256 | RXFP1 | NM_001253732 | 1 | 1 | 0 | 1 | 1 | 4 |
| hsa-miR-181a-5p | MIMAT0000256 | TGIF2 | NM_001199514 | 1 | 1 | 0 | 1 | 1 | 4 |
| hsa-miR-181a-5p | MIMAT0000256 | BACH2 | NM_021813 | 1 | 1 | 0 | 1 | 1 | 4 |
| hsa-miR-181a-5p | MIMAT0000256 | C2orf43 | NM_021925 | 1 | 1 | 0 | 1 | 1 | 4 |
| hsa-miR-181a-5p | MIMAT0000256 | PKNOX2 | NM_022062 | 0 | 1 | 1 | 1 | 1 | 4 |
| hsa-miR-181a-5p | MIMAT0000256 | MRPS14 | NM_022100 | 1 | 1 | 0 | 1 | 1 | 4 |
| hsa-miR-181a-5p | MIMAT0000256 | ZNF667 | NM_022103 | 1 | 1 | 0 | 1 | 1 | 4 |
| hsa-miR-181a-5p | MIMAT0000256 | SLC39A8 | NM_001135147 | 1 | 1 | 0 | 1 | 1 | 4 |
| hsa-miR-181a-5p | MIMAT0000256 | ZFYVE20 | NM_022340 | 1 | 1 | 0 | 1 | 1 | 4 |
| hsa-miR-181a-5p | MIMAT0000256 | IRF2BPL | NM_024496 | 1 | 1 | 0 | 1 | 1 | 4 |
| hsa-miR-181a-5p | MIMAT0000256 | RGS18 | NM_130782 | 1 | 1 | 1 | 0 | 1 | 4 |
| hsa-miR-181a-5p | MIMAT0000256 | SUDS3 | NM_022491 | 0 | 1 | 1 | 1 | 1 | 4 |
| hsa-miR-181a-5p | MIMAT0000256 | MRPS25 | XM_005265401 | 1 | 1 | 0 | 1 | 1 | 4 |
| hsa-miR-181a-5p | MIMAT0000256 | CCDC14 | NM_022757 | 1 | 1 | 1 | 0 | 1 | 4 |
| hsa-miR-181a-5p | MIMAT0000256 | RMND5A | NM_022780 | 1 | 1 | 0 | 1 | 1 | 4 |
| hsa-miR-181a-5p | MIMAT0000256 | DEPTOR | NM_022783 | 1 | 1 | 1 | 0 | 1 | 4 |
| hsa-miR-181a-5p | MIMAT0000256 | YTHDC2 | NM_022828 | 0 | 1 | 1 | 1 | 1 | 4 |
| hsa-miR-181a-5p | MIMAT0000256 | REEP1 | XM_005264502 | 1 | 1 | 0 | 1 | 1 | 4 |
| hsa-miR-181a-5p | MIMAT0000256 | TMEM237 | NM_152388 | 1 | 1 | 0 | 1 | 1 | 4 |
| hsa-miR-181a-5p | MIMAT0000256 | TMEM135 | NM_022918 | 1 | 1 | 1 | 0 | 1 | 4 |
| hsa-miR-181a-5p | MIMAT0000256 | PHACTR4 | NM_001048183 | 0 | 1 | 1 | 1 | 1 | 4 |
| hsa-miR-181a-5p | MIMAT0000256 | CAPRIN2 | NM_032156 | 0 | 1 | 1 | 1 | 1 | 4 |
| hsa-miR-181a-5p | MIMAT0000256 | TRAK2 | NM_015049 | 1 | 1 | 0 | 1 | 1 | 4 |
| hsa-miR-181a-5p | MIMAT0000256 | MTMR9 | NM_015458 | 0 | 1 | 1 | 1 | 1 | 4 |
| hsa-miR-181a-5p | MIMAT0000256 | SECISBP2 | NM_024077 | 1 | 1 | 0 | 1 | 1 | 4 |
| hsa-miR-181a-5p | MIMAT0000256 | DERL1 | NM_024295 | 0 | 1 | 1 | 1 | 1 | 4 |
| hsa-miR-181a-5p | MIMAT0000256 | CENPO | NM_001199803 | 1 | 1 | 0 | 1 | 1 | 4 |
| hsa-miR-181a-5p | MIMAT0000256 | TTPAL | XM_005260549 | 1 | 1 | 0 | 1 | 1 | 4 |
| hsa-miR-181a-5p | MIMAT0000256 | HMBOX1 | XM_005273634 | 1 | 1 | 0 | 1 | 1 | 4 |
| hsa-miR-181a-5p | MIMAT0000256 | PARP8 | NM_001178055 | 1 | 1 | 0 | 1 | 1 | 4 |
| hsa-miR-181a-5p | MIMAT0000256 | ZNF329 | NM_024620 | 0 | 1 | 1 | 1 | 1 | 4 |
| hsa-miR-181a-5p | MIMAT0000256 | SMC6 | XM_005262627 | 1 | 1 | 0 | 1 | 1 | 4 |
| hsa-miR-181a-5p | MIMAT0000256 | NSUN7 | NM_024677 | 1 | 1 | 1 | 0 | 1 | 4 |
| hsa-miR-181a-5p | MIMAT0000256 | KATNBL1 | NM_024713 | 1 | 1 | 1 | 0 | 1 | 4 |
| hsa-miR-181a-5p | MIMAT0000256 | TXNDC15 | NM_024715 | 1 | 1 | 1 | 0 | 1 | 4 |
| hsa-miR-181a-5p | MIMAT0000256 | ZFHX4 | NM_024721 | 1 | 1 | 0 | 1 | 1 | 4 |
| hsa-miR-181a-5p | MIMAT0000256 | ALG9 | NM_001077691 | 1 | 1 | 1 | 0 | 1 | 4 |
| hsa-miR-181a-5p | MIMAT0000256 | GSTCD | NM_001031720 | 1 | 1 | 0 | 1 | 1 | 4 |
| hsa-miR-181a-5p | MIMAT0000256 | PEAK1 | XM_005254672 | 1 | 1 | 0 | 1 | 1 | 4 |
| hsa-miR-181a-5p | MIMAT0000256 | CSRNP3 | NM_001172173 | 1 | 1 | 0 | 1 | 1 | 4 |
| hsa-miR-181a-5p | MIMAT0000256 | VCPIP1 | NM_025054 | 1 | 1 | 0 | 1 | 1 | 4 |
| hsa-miR-181a-5p | MIMAT0000256 | ASRGL1 | NM_001083926 | 1 | 1 | 0 | 1 | 1 | 4 |
| hsa-miR-181a-5p | MIMAT0000256 | COQ10B | NM_025147 | 1 | 1 | 0 | 1 | 1 | 4 |
| hsa-miR-181a-5p | MIMAT0000256 | WDR26 | NM_025160 | 1 | 1 | 0 | 1 | 1 | 4 |
| hsa-miR-181a-5p | MIMAT0000256 | KLHL15 | NM_030624 | 1 | 1 | 0 | 1 | 1 | 4 |
| hsa-miR-181a-5p | MIMAT0000256 | DNAJC5 | NM_025219 | 1 | 1 | 0 | 1 | 1 | 4 |
| hsa-miR-181a-5p | MIMAT0000256 | DCAF11 | NM_025230 | 1 | 1 | 0 | 1 | 1 | 4 |
| hsa-miR-181a-5p | MIMAT0000256 | SRCIN1 | NM_025248 | 1 | 1 | 0 | 1 | 1 | 4 |
| hsa-miR-181a-5p | MIMAT0000256 | THSD7B | XM_005263802 | 1 | 1 | 0 | 1 | 1 | 4 |
| hsa-miR-181a-5p | MIMAT0000256 | ZNF436 | NM_001077195 | 1 | 1 | 0 | 1 | 1 | 4 |
| hsa-miR-181a-5p | MIMAT0000256 | OR51E2 | NM_030774 | 1 | 1 | 0 | 1 | 1 | 4 |
| hsa-miR-181a-5p | MIMAT0000256 | ANKRD13C | NM_030816 | 1 | 1 | 1 | 0 | 1 | 4 |
| hsa-miR-181a-5p | MIMAT0000256 | PLA2G12A | NM_030821 | 1 | 1 | 0 | 1 | 1 | 4 |
| hsa-miR-181a-5p | MIMAT0000256 | ADAMTS12 | XM_005248381 | 1 | 1 | 0 | 1 | 1 | 4 |
| hsa-miR-181a-5p | MIMAT0000256 | APH1B | NM_031301 | 1 | 1 | 0 | 1 | 1 | 4 |
| hsa-miR-181a-5p | MIMAT0000256 | NUDT12 | NM_031438 | 0 | 1 | 1 | 1 | 1 | 4 |
| hsa-miR-181a-5p | MIMAT0000256 | SOX7 | NM_031439 | 1 | 1 | 0 | 1 | 1 | 4 |
| hsa-miR-181a-5p | MIMAT0000256 | C19orf12 | NM_031448 | 1 | 1 | 1 | 0 | 1 | 4 |
| hsa-miR-181a-5p | MIMAT0000256 | FRMD8 | NM_031904 | 1 | 1 | 0 | 1 | 1 | 4 |
| hsa-miR-181a-5p | MIMAT0000256 | FSD1L | XM_005252254 | 1 | 1 | 0 | 1 | 1 | 4 |
| hsa-miR-181a-5p | MIMAT0000256 | TMTC1 | XM_005253498 | 1 | 1 | 0 | 1 | 1 | 4 |
| hsa-miR-181a-5p | MIMAT0000256 | NCALD | XM_005251079 | 1 | 1 | 0 | 1 | 1 | 4 |
| hsa-miR-181a-5p | MIMAT0000256 | B3GNT5 | XM_005247823 | 1 | 1 | 0 | 1 | 1 | 4 |
| hsa-miR-181a-5p | MIMAT0000256 | SLC10A7 | XM_005263276 | 1 | 1 | 0 | 1 | 1 | 4 |
| hsa-miR-181a-5p | MIMAT0000256 | RHBDD1 | NM_001167608 | 1 | 1 | 0 | 1 | 1 | 4 |
| hsa-miR-181a-5p | MIMAT0000256 | ING5 | NM_032329 | 1 | 1 | 0 | 1 | 1 | 4 |
| hsa-miR-181a-5p | MIMAT0000256 | UTP23 | NM_032334 | 1 | 1 | 0 | 1 | 1 | 4 |
| hsa-miR-181a-5p | MIMAT0000256 | C15orf48 | NM_032413 | 1 | 1 | 1 | 0 | 1 | 4 |
| hsa-miR-181a-5p | MIMAT0000256 | CARD11 | NM_032415 | 1 | 1 | 0 | 1 | 1 | 4 |
| hsa-miR-181a-5p | MIMAT0000256 | MEGF10 | XM_005272114 | 1 | 1 | 0 | 1 | 1 | 4 |
| hsa-miR-181a-5p | MIMAT0000256 | PARD6B | NM_032521 | 1 | 1 | 0 | 1 | 1 | 4 |
| hsa-miR-181a-5p | MIMAT0000256 | ZNF594 | XM_005256827 | 1 | 1 | 0 | 1 | 1 | 4 |
| hsa-miR-181a-5p | MIMAT0000256 | TTBK1 | NM_032538 | 1 | 1 | 0 | 1 | 1 | 4 |
| hsa-miR-181a-5p | MIMAT0000256 | SLITRK2 | XM_005262343 | 1 | 1 | 0 | 1 | 1 | 4 |
| hsa-miR-181a-5p | MIMAT0000256 | NFATC2IP | NM_032815 | 0 | 1 | 1 | 1 | 1 | 4 |
| hsa-miR-181a-5p | MIMAT0000256 | LRP11 | NM_032832 | 1 | 1 | 0 | 1 | 1 | 4 |
| hsa-miR-181a-5p | MIMAT0000256 | ZNF566 | NM_001145345 | 1 | 1 | 0 | 1 | 1 | 4 |
| hsa-miR-181a-5p | MIMAT0000256 | ABHD13 | NM_032859 | 1 | 1 | 1 | 0 | 1 | 4 |
| hsa-miR-181a-5p | MIMAT0000256 | TMEM241 | NM_032933 | 1 | 1 | 0 | 1 | 1 | 4 |
| hsa-miR-181a-5p | MIMAT0000256 | KIAA1644 | XM_005261790 | 1 | 1 | 0 | 1 | 1 | 4 |
| hsa-miR-181a-5p | MIMAT0000256 | UBE3D | NM_198920 | 1 | 1 | 0 | 1 | 1 | 4 |
| hsa-miR-181a-5p | MIMAT0000256 | ZNF468 | NM_001277120 | 1 | 1 | 0 | 1 | 1 | 4 |
| hsa-miR-181a-5p | MIMAT0000256 | TMEM41A | NM_080652 | 1 | 1 | 0 | 1 | 1 | 4 |
| hsa-miR-181a-5p | MIMAT0000256 | CCDC126 | NM_138771 | 1 | 1 | 0 | 1 | 1 | 4 |
| hsa-miR-181a-5p | MIMAT0000256 | ZNF697 | XM_005271315 | 1 | 1 | 0 | 1 | 1 | 4 |
| hsa-miR-181a-5p | MIMAT0000256 | SESTD1 | NM_178123 | 1 | 1 | 0 | 1 | 1 | 4 |
| hsa-miR-181a-5p | MIMAT0000256 | ZNF765 | NM_001040185 | 1 | 1 | 0 | 1 | 1 | 4 |
| hsa-miR-181a-5p | MIMAT0000256 | LONRF1 | NM_152271 | 0 | 1 | 1 | 1 | 1 | 4 |
| hsa-miR-181a-5p | MIMAT0000256 | YTHDC1 | NM_001031732 | 1 | 1 | 1 | 0 | 1 | 4 |
| hsa-miR-181a-5p | MIMAT0000256 | DSEL | NM_032160 | 1 | 1 | 0 | 1 | 1 | 4 |
| hsa-miR-181a-5p | MIMAT0000256 | TIFA | NM_052864 | 1 | 1 | 1 | 0 | 1 | 4 |
| hsa-miR-181a-5p | MIMAT0000256 | MAPK1IP1L | NM_144578 | 1 | 1 | 1 | 0 | 1 | 4 |
| hsa-miR-181a-5p | MIMAT0000256 | FOXP2 | NM_148898 | 1 | 1 | 0 | 1 | 1 | 4 |
| hsa-miR-181a-5p | MIMAT0000256 | ARHGAP12 | NM_018287 | 1 | 1 | 0 | 1 | 1 | 4 |
| hsa-miR-181a-5p | MIMAT0000256 | H2AFV | NM_138635 | 1 | 1 | 1 | 0 | 1 | 4 |
| hsa-miR-181a-5p | MIMAT0000256 | CEP41 | NM_018718 | 1 | 1 | 0 | 1 | 1 | 4 |
| hsa-miR-181a-5p | MIMAT0000256 | SLC46A1 | NM_080669 | 1 | 1 | 0 | 1 | 1 | 4 |
| hsa-miR-181a-5p | MIMAT0000256 | SLC2A13 | XM_005268615 | 1 | 1 | 0 | 1 | 1 | 4 |
| hsa-miR-181a-5p | MIMAT0000256 | PALM2 | NM_053016 | 1 | 1 | 1 | 0 | 1 | 4 |
| hsa-miR-181a-5p | MIMAT0000256 | SLITRK1 | NM_052910 | 0 | 1 | 1 | 1 | 1 | 4 |
| hsa-miR-181a-5p | MIMAT0000256 | KLHL29 | NM_052920 | 1 | 1 | 1 | 0 | 1 | 4 |
| hsa-miR-181a-5p | MIMAT0000256 | OSBPL1A | NM_080597 | 1 | 1 | 0 | 1 | 1 | 4 |
| hsa-miR-181a-5p | MIMAT0000256 | OSBPL6 | NM_001201480 | 1 | 1 | 0 | 1 | 1 | 4 |
| hsa-miR-181a-5p | MIMAT0000256 | OSBPL8 | XM_005268621 | 1 | 1 | 0 | 1 | 1 | 4 |
| hsa-miR-181a-5p | MIMAT0000256 | FBXO32 | NM_058229 | 1 | 1 | 0 | 1 | 1 | 4 |
| hsa-miR-181a-5p | MIMAT0000256 | KCTD12 | NM_138444 | 1 | 1 | 0 | 1 | 1 | 4 |
| hsa-miR-181a-5p | MIMAT0000256 | C12orf56 | NM_001170633 | 1 | 1 | 0 | 1 | 1 | 4 |
| hsa-miR-181a-5p | MIMAT0000256 | DIS3L | XM_005254144 | 1 | 1 | 0 | 1 | 1 | 4 |
| hsa-miR-181a-5p | MIMAT0000256 | LRRC58 | NM_001099678 | 1 | 1 | 0 | 1 | 1 | 4 |
| hsa-miR-181a-5p | MIMAT0000256 | TADA1 | NM_053053 | 1 | 1 | 1 | 0 | 1 | 4 |
| hsa-miR-181a-5p | MIMAT0000256 | MIA2 | NM_054024 | 1 | 1 | 1 | 0 | 1 | 4 |
| hsa-miR-181a-5p | MIMAT0000256 | RAB3IP | NM_175623 | 1 | 1 | 1 | 0 | 1 | 4 |
| hsa-miR-181a-5p | MIMAT0000256 | GALNT15 | NM_054110 | 1 | 1 | 0 | 1 | 1 | 4 |
| hsa-miR-181a-5p | MIMAT0000256 | PARD3B | XM_005246273 | 1 | 1 | 0 | 1 | 1 | 4 |
| hsa-miR-181a-5p | MIMAT0000256 | ANAPC16 | NM_001242546 | 1 | 1 | 1 | 0 | 1 | 4 |
| hsa-miR-181a-5p | MIMAT0000256 | FAT3 | XM_005273759 | 1 | 1 | 0 | 1 | 1 | 4 |
| hsa-miR-181a-5p | MIMAT0000256 | NIPA1 | NM_144599 | 1 | 1 | 0 | 1 | 1 | 4 |
| hsa-miR-181a-5p | MIMAT0000256 | MSI2 | XM_005257014 | 1 | 1 | 0 | 1 | 1 | 4 |
| hsa-miR-181a-5p | MIMAT0000256 | ZNF441 | NM_152355 | 1 | 1 | 1 | 0 | 1 | 4 |
| hsa-miR-181a-5p | MIMAT0000256 | MAB21L3 | NM_152367 | 1 | 1 | 1 | 0 | 1 | 4 |
| hsa-miR-181a-5p | MIMAT0000256 | TYW5 | NM_001039693 | 1 | 1 | 0 | 1 | 1 | 4 |
| hsa-miR-181a-5p | MIMAT0000256 | MBOAT2 | NM_138799 | 1 | 1 | 1 | 0 | 1 | 4 |
| hsa-miR-181a-5p | MIMAT0000256 | KCNH8 | NM_144633 | 1 | 1 | 1 | 0 | 1 | 4 |
| hsa-miR-181a-5p | MIMAT0000256 | FAM43A | NM_153690 | 1 | 1 | 0 | 1 | 1 | 4 |
| hsa-miR-181a-5p | MIMAT0000256 | SYNPR | NM_144642 | 0 | 1 | 1 | 1 | 1 | 4 |
| hsa-miR-181a-5p | MIMAT0000256 | C4orf33 | XM_005262734 | 1 | 1 | 0 | 1 | 1 | 4 |
| hsa-miR-181a-5p | MIMAT0000256 | SPATA18 | NM_145263 | 1 | 1 | 0 | 1 | 1 | 4 |
| hsa-miR-181a-5p | MIMAT0000256 | PPARGC1B | NM_133263 | 1 | 1 | 0 | 1 | 1 | 4 |
| hsa-miR-181a-5p | MIMAT0000256 | HINT3 | NM_138571 | 1 | 1 | 0 | 1 | 1 | 4 |
| hsa-miR-181a-5p | MIMAT0000256 | CD109 | NM_133493 | 1 | 1 | 0 | 1 | 1 | 4 |
| hsa-miR-181a-5p | MIMAT0000256 | FAM199X | NM_207318 | 1 | 1 | 0 | 1 | 1 | 4 |
| hsa-miR-181a-5p | MIMAT0000256 | NANP | NM_152667 | 1 | 1 | 0 | 1 | 1 | 4 |
| hsa-miR-181a-5p | MIMAT0000256 | SAMD8 | NM_144660 | 1 | 1 | 0 | 1 | 1 | 4 |
| hsa-miR-181a-5p | MIMAT0000256 | MPP7 | NM_173496 | 1 | 1 | 1 | 0 | 1 | 4 |
| hsa-miR-181a-5p | MIMAT0000256 | TMEM86A | NM_153347 | 1 | 1 | 0 | 1 | 1 | 4 |
| hsa-miR-181a-5p | MIMAT0000256 | FAM216B | NM_182508 | 1 | 1 | 0 | 1 | 1 | 4 |
| hsa-miR-181a-5p | MIMAT0000256 | ZNF558 | XM_005259755 | 1 | 1 | 0 | 1 | 1 | 4 |
| hsa-miR-181a-5p | MIMAT0000256 | ZNF569 | XM_005258563 | 1 | 1 | 0 | 1 | 1 | 4 |
| hsa-miR-181a-5p | MIMAT0000256 | CCDC117 | NM_173510 | 1 | 1 | 1 | 0 | 1 | 4 |
| hsa-miR-181a-5p | MIMAT0000256 | ZSWIM2 | NM_182521 | 1 | 1 | 0 | 1 | 1 | 4 |
| hsa-miR-181a-5p | MIMAT0000256 | GPR155 | NM_001033045 | 1 | 1 | 0 | 1 | 1 | 4 |
| hsa-miR-181a-5p | MIMAT0000256 | RNF38 | NM_194328 | 1 | 1 | 0 | 1 | 1 | 4 |
| hsa-miR-181a-5p | MIMAT0000256 | IGSF11 | NM_152538 | 0 | 1 | 1 | 1 | 1 | 4 |
| hsa-miR-181a-5p | MIMAT0000256 | CREBRF | NM_153607 | 1 | 1 | 1 | 0 | 1 | 4 |
| hsa-miR-181a-5p | MIMAT0000256 | MBLAC2 | NM_203406 | 0 | 1 | 1 | 1 | 1 | 4 |
| hsa-miR-181a-5p | MIMAT0000256 | RNF145 | NM_001199383 | 0 | 1 | 1 | 1 | 1 | 4 |
| hsa-miR-181a-5p | MIMAT0000256 | CNKSR3 | NM_173515 | 0 | 1 | 1 | 1 | 1 | 4 |
| hsa-miR-181a-5p | MIMAT0000256 | CLVS1 | XM_005251175 | 1 | 1 | 0 | 1 | 1 | 4 |
| hsa-miR-181a-5p | MIMAT0000256 | ZXDB | NM_007157 | 1 | 1 | 0 | 1 | 1 | 4 |
| hsa-miR-181a-5p | MIMAT0000256 | SLC35G1 | XM_005269584 | 1 | 1 | 0 | 1 | 1 | 4 |
| hsa-miR-181a-5p | MIMAT0000256 | DENND5B | NM_144973 | 1 | 1 | 0 | 1 | 1 | 4 |
| hsa-miR-181a-5p | MIMAT0000256 | CCDC60 | NM_178499 | 1 | 1 | 0 | 1 | 1 | 4 |
| hsa-miR-181a-5p | MIMAT0000256 | ITPRIPL2 | NM_001034841 | 1 | 1 | 0 | 1 | 1 | 4 |
| hsa-miR-181a-5p | MIMAT0000256 | RHBDL3 | NM_138328 | 1 | 1 | 0 | 1 | 1 | 4 |
| hsa-miR-181a-5p | MIMAT0000256 | C18orf54 | XM_005258201 | 1 | 1 | 0 | 1 | 1 | 4 |
| hsa-miR-181a-5p | MIMAT0000256 | LPPR5 | NM_001037317 | 1 | 1 | 0 | 1 | 1 | 4 |
| hsa-miR-181a-5p | MIMAT0000256 | LONRF2 | NM_198461 | 0 | 1 | 1 | 1 | 1 | 4 |
| hsa-miR-181a-5p | MIMAT0000256 | PRICKLE2 | NM_198859 | 1 | 1 | 0 | 1 | 1 | 4 |
| hsa-miR-181a-5p | MIMAT0000256 | RBM46 | NM_001277171 | 1 | 1 | 0 | 1 | 1 | 4 |
| hsa-miR-181a-5p | MIMAT0000256 | SLC30A8 | NM_001172813 | 1 | 1 | 0 | 1 | 1 | 4 |
| hsa-miR-181a-5p | MIMAT0000256 | TMEM252 | NM_153237 | 1 | 1 | 0 | 1 | 1 | 4 |
| hsa-miR-181a-5p | MIMAT0000256 | GLIS3 | NM_001042413 | 1 | 1 | 0 | 1 | 1 | 4 |
| hsa-miR-181a-5p | MIMAT0000256 | ARID2 | NM_152641 | 1 | 1 | 0 | 1 | 1 | 4 |
| hsa-miR-181a-5p | MIMAT0000256 | TXLNA | NM_175852 | 1 | 1 | 0 | 1 | 1 | 4 |
| hsa-miR-181a-5p | MIMAT0000256 | CREG2 | NM_153836 | 1 | 1 | 0 | 1 | 1 | 4 |
| hsa-miR-181a-5p | MIMAT0000256 | FBXO45 | NM_001105573 | 1 | 1 | 1 | 0 | 1 | 4 |
| hsa-miR-181a-5p | MIMAT0000256 | TAPT1 | NM_153365 | 1 | 1 | 0 | 1 | 1 | 4 |
| hsa-miR-181a-5p | MIMAT0000256 | DNAJC18 | XM_005271917 | 1 | 1 | 0 | 1 | 1 | 4 |
| hsa-miR-181a-5p | MIMAT0000256 | GAPT | NM_152687 | 1 | 1 | 1 | 0 | 1 | 4 |
| hsa-miR-181a-5p | MIMAT0000256 | CERS3 | NM_178842 | 1 | 1 | 1 | 0 | 1 | 4 |
| hsa-miR-181a-5p | MIMAT0000256 | RTKN2 | NM_145307 | 1 | 1 | 0 | 1 | 1 | 4 |
| hsa-miR-181a-5p | MIMAT0000256 | PLAC1L | NM_173801 | 1 | 1 | 1 | 0 | 1 | 4 |
| hsa-miR-181a-5p | MIMAT0000256 | FAM124A | NM_145019 | 1 | 1 | 0 | 1 | 1 | 4 |
| hsa-miR-181a-5p | MIMAT0000256 | TDRD6 | NM_001010870 | 1 | 1 | 1 | 0 | 1 | 4 |
| hsa-miR-181a-5p | MIMAT0000256 | RNF182 | NM_001165032 | 1 | 1 | 1 | 0 | 1 | 4 |
| hsa-miR-181a-5p | MIMAT0000256 | SLC35F1 | NM_001029858 | 1 | 1 | 0 | 1 | 1 | 4 |
| hsa-miR-181a-5p | MIMAT0000256 | ZNRF2 | NM_147128 | 0 | 1 | 1 | 1 | 1 | 4 |
| hsa-miR-181a-5p | MIMAT0000256 | CNOT6L | NM_144571 | 1 | 1 | 0 | 1 | 1 | 4 |
| hsa-miR-181a-5p | MIMAT0000256 | ZNF396 | NM_145756 | 1 | 1 | 1 | 0 | 1 | 4 |
| hsa-miR-181a-5p | MIMAT0000256 | STXBP4 | XM_005257185 | 1 | 1 | 0 | 1 | 1 | 4 |
| hsa-miR-181a-5p | MIMAT0000256 | IPMK | NM_152230 | 1 | 1 | 1 | 0 | 1 | 4 |
| hsa-miR-181a-5p | MIMAT0000256 | GPATCH11 | NM_174931 | 1 | 1 | 1 | 0 | 1 | 4 |
| hsa-miR-181a-5p | MIMAT0000256 | BRWD3 | NM_153252 | 1 | 1 | 0 | 1 | 1 | 4 |
| hsa-miR-181a-5p | MIMAT0000256 | FBXO33 | NM_203301 | 1 | 1 | 1 | 0 | 1 | 4 |
| hsa-miR-181a-5p | MIMAT0000256 | RNF169 | NM_001098638 | 1 | 1 | 0 | 1 | 1 | 4 |
| hsa-miR-181a-5p | MIMAT0000256 | LCORL | XM_005248145 | 1 | 1 | 0 | 1 | 1 | 4 |
| hsa-miR-181a-5p | MIMAT0000256 | NAALADL2 | NM_207015 | 1 | 1 | 1 | 0 | 1 | 4 |
| hsa-miR-181a-5p | MIMAT0000256 | RNF144B | XM_005248985 | 1 | 1 | 0 | 1 | 1 | 4 |
| hsa-miR-181a-5p | MIMAT0000256 | ST6GALNAC3 | NM_152996 | 1 | 1 | 1 | 0 | 1 | 4 |
| hsa-miR-181a-5p | MIMAT0000256 | MFSD8 | NM_152778 | 1 | 1 | 0 | 1 | 1 | 4 |
| hsa-miR-181a-5p | MIMAT0000256 | GXYLT1 | NM_173601 | 1 | 1 | 0 | 1 | 1 | 4 |
| hsa-miR-181a-5p | MIMAT0000256 | NWD1 | NM_001007525 | 1 | 1 | 0 | 1 | 1 | 4 |
| hsa-miR-181a-5p | MIMAT0000256 | FAM102B | XM_005270772 | 1 | 1 | 0 | 1 | 1 | 4 |
| hsa-miR-181a-5p | MIMAT0000256 | SYPL2 | NM_001040709 | 1 | 1 | 0 | 1 | 1 | 4 |
| hsa-miR-181a-5p | MIMAT0000256 | CCDC141 | NM_173648 | 1 | 1 | 0 | 1 | 1 | 4 |
| hsa-miR-181a-5p | MIMAT0000256 | UNC80 | XM_005246476 | 1 | 1 | 0 | 1 | 1 | 4 |
| hsa-miR-181a-5p | MIMAT0000256 | RABL3 | NM_173825 | 1 | 1 | 0 | 1 | 1 | 4 |
| hsa-miR-181a-5p | MIMAT0000256 | GPRIN3 | XM_005262936 | 1 | 1 | 0 | 1 | 1 | 4 |
| hsa-miR-181a-5p | MIMAT0000256 | RSPO2 | NM_178565 | 1 | 1 | 0 | 1 | 1 | 4 |
| hsa-miR-181a-5p | MIMAT0000256 | ZDHHC21 | NM_178566 | 1 | 1 | 1 | 0 | 1 | 4 |
| hsa-miR-181a-5p | MIMAT0000256 | KIAA2022 | NM_001008537 | 0 | 1 | 1 | 1 | 1 | 4 |
| hsa-miR-181a-5p | MIMAT0000256 | ZC3H12B | XM_005262260 | 1 | 1 | 0 | 1 | 1 | 4 |
| hsa-miR-181a-5p | MIMAT0000256 | FREM2 | NM_207361 | 1 | 1 | 0 | 1 | 1 | 4 |
| hsa-miR-181a-5p | MIMAT0000256 | ZNF677 | NM_182609 | 0 | 1 | 1 | 1 | 1 | 4 |
| hsa-miR-181a-5p | MIMAT0000256 | PLCXD3 | NM_001005473 | 1 | 1 | 0 | 1 | 1 | 4 |
| hsa-miR-181a-5p | MIMAT0000256 | MACC1 | NM_182762 | 1 | 1 | 0 | 1 | 1 | 4 |
| hsa-miR-181a-5p | MIMAT0000256 | ZNF81 | NM_007137 | 1 | 1 | 0 | 1 | 1 | 4 |
| hsa-miR-181a-5p | MIMAT0000256 | TICAM2 | NM_021649 | 1 | 1 | 0 | 1 | 1 | 4 |
| hsa-miR-181a-5p | MIMAT0000256 | LHFPL3 | NM_199000 | 1 | 1 | 1 | 0 | 1 | 4 |
| hsa-miR-181a-5p | MIMAT0000256 | CEP85L | NM_001042475 | 1 | 1 | 0 | 1 | 1 | 4 |
| hsa-miR-181a-5p | MIMAT0000256 | CC2D2B | XM_005269814 | 1 | 1 | 0 | 1 | 1 | 4 |
| hsa-miR-181a-5p | MIMAT0000256 | YPEL2 | NM_001005404 | 1 | 1 | 0 | 1 | 1 | 4 |
| hsa-miR-181a-5p | MIMAT0000256 | TRNP1 | NM_001013642 | 0 | 1 | 1 | 1 | 1 | 4 |
| hsa-miR-181a-5p | MIMAT0000256 | VGLL3 | NM_016206 | 1 | 1 | 0 | 1 | 1 | 4 |
| hsa-miR-181a-5p | MIMAT0000256 | BEND4 | NM_207406 | 1 | 1 | 0 | 1 | 1 | 4 |
| hsa-miR-181a-5p | MIMAT0000256 | LIN28B | NM_001004317 | 0 | 1 | 1 | 1 | 1 | 4 |
| hsa-miR-181a-5p | MIMAT0000256 | IYD | NM_001164694 | 1 | 1 | 0 | 1 | 1 | 4 |
| hsa-miR-181a-5p | MIMAT0000256 | ANKRD34C | NM_001146341 | 1 | 1 | 1 | 0 | 1 | 4 |
| hsa-miR-181a-5p | MIMAT0000256 | FAM102A | NM_001035254 | 1 | 1 | 0 | 1 | 1 | 4 |
| hsa-miR-181a-5p | MIMAT0000256 | SERTM1 | NM_203451 | 1 | 1 | 0 | 1 | 1 | 4 |
| hsa-miR-181a-5p | MIMAT0000256 | C1orf226 | NM_001085375 | 1 | 1 | 0 | 1 | 1 | 4 |
| hsa-miR-181a-5p | MIMAT0000256 | RGPD1 | NM_001024457 | 0 | 1 | 1 | 1 | 1 | 4 |
| hsa-miR-181a-5p | MIMAT0000256 | SNX30 | NM_001012994 | 1 | 1 | 0 | 1 | 1 | 4 |
| hsa-miR-181a-5p | MIMAT0000256 | ZBTB34 | XM_005251989 | 1 | 1 | 0 | 1 | 1 | 4 |
| hsa-miR-181a-5p | MIMAT0000256 | PEF1 | NM_012392 | 1 | 1 | 0 | 1 | 1 | 4 |
| hsa-miR-181a-5p | MIMAT0000256 | ZNF704 | XM_005251279 | 1 | 1 | 0 | 1 | 1 | 4 |
| hsa-miR-181a-5p | MIMAT0000256 | ZFP62 | NM_152283 | 1 | 1 | 1 | 0 | 1 | 4 |
| hsa-miR-181a-5p | MIMAT0000256 | SLC35E2B | NM_001110781 | 1 | 1 | 0 | 1 | 1 | 4 |
| hsa-miR-181a-5p | MIMAT0000256 | ZNF814 | NM_001144989 | 1 | 1 | 1 | 0 | 1 | 4 |
| hsa-miR-181a-5p | MIMAT0000256 | TMED7-TICAM2 | NM_001164469 | 1 | 1 | 0 | 1 | 1 | 4 |
| hsa-miR-181a-5p | MIMAT0000256 | OCLN | NM_002538 | 1 | 1 | 0 | 1 | 1 | 4 |
| hsa-miR-181a-5p | MIMAT0000256 | SMLR1 | NM_001195597 | 1 | 1 | 1 | 0 | 1 | 4 |
| hsa-miR-181a-5p | MIMAT0000256 | ACP1 | NM_001040649 | 1 | 1 | 1 | 0 | 1 | 4 |
| hsa-miR-181a-5p | MIMAT0000256 | ACVR2B | NM_001106 | 1 | 1 | 1 | 0 | 1 | 4 |
| hsa-miR-181a-5p | MIMAT0000256 | ACYP1 | NM_001107 | 1 | 1 | 1 | 0 | 1 | 4 |
| hsa-miR-181a-5p | MIMAT0000256 | ADARB2 | NM_018702 | 1 | 1 | 1 | 0 | 1 | 4 |
| hsa-miR-181a-5p | MIMAT0000256 | ALDH9A1 | XM_005244965 | 1 | 1 | 0 | 1 | 1 | 4 |
| hsa-miR-181a-5p | MIMAT0000256 | BIRC5 | NM_001012271 | 1 | 1 | 0 | 1 | 1 | 4 |
| hsa-miR-181a-5p | MIMAT0000256 | AQP9 | NM_020980 | 1 | 1 | 0 | 1 | 1 | 4 |
| hsa-miR-181a-5p | MIMAT0000256 | ARF6 | NM_001663 | 1 | 1 | 1 | 0 | 1 | 4 |
| hsa-miR-181a-5p | MIMAT0000256 | ARHGAP5 | XM_005267636 | 1 | 1 | 0 | 1 | 1 | 4 |
| hsa-miR-181a-5p | MIMAT0000256 | ATM | NM_000051 | 1 | 1 | 1 | 0 | 1 | 4 |
| hsa-miR-181a-5p | MIMAT0000256 | ATP2B2 | NM_001683 | 1 | 1 | 1 | 0 | 1 | 4 |
| hsa-miR-181a-5p | MIMAT0000256 | BAI3 | NM_001704 | 1 | 1 | 1 | 0 | 1 | 4 |
| hsa-miR-181a-5p | MIMAT0000256 | BCHE | NM_000055 | 1 | 1 | 1 | 0 | 1 | 4 |
| hsa-miR-181a-5p | MIMAT0000256 | MPPED2 | NM_001145399 | 1 | 1 | 0 | 1 | 1 | 4 |
| hsa-miR-181a-5p | MIMAT0000256 | CACNA1E | XM_005245477 | 1 | 1 | 0 | 1 | 1 | 4 |
| hsa-miR-181a-5p | MIMAT0000256 | CBL | NM_005188 | 1 | 1 | 0 | 1 | 1 | 4 |
| hsa-miR-181a-5p | MIMAT0000256 | CD1E | NM_030893 | 0 | 1 | 1 | 1 | 1 | 4 |
| hsa-miR-181a-5p | MIMAT0000256 | CD48 | NM_001778 | 1 | 1 | 1 | 0 | 1 | 4 |
| hsa-miR-181a-5p | MIMAT0000256 | CDKN1B | NM_004064 | 1 | 1 | 0 | 1 | 1 | 4 |
| hsa-miR-181a-5p | MIMAT0000256 | CETN3 | NM_004365 | 1 | 1 | 1 | 0 | 1 | 4 |
| hsa-miR-181a-5p | MIMAT0000256 | RCBTB2 | NM_001268 | 1 | 1 | 0 | 1 | 1 | 4 |
| hsa-miR-181a-5p | MIMAT0000256 | CHEK1 | NM_001114122 | 1 | 1 | 0 | 1 | 1 | 4 |
| hsa-miR-181a-5p | MIMAT0000256 | CHRNB2 | NM_000748 | 1 | 1 | 0 | 1 | 1 | 4 |
| hsa-miR-181a-5p | MIMAT0000256 | CLCN6 | NM_001286 | 1 | 1 | 0 | 1 | 1 | 4 |
| hsa-miR-181a-5p | MIMAT0000256 | COL16A1 | NM_001856 | 1 | 1 | 0 | 1 | 1 | 4 |
| hsa-miR-181a-5p | MIMAT0000256 | COL19A1 | NM_001858 | 1 | 1 | 1 | 0 | 1 | 4 |
| hsa-miR-181a-5p | MIMAT0000256 | DNAH8 | NM_001206927 | 1 | 1 | 1 | 0 | 1 | 4 |
| hsa-miR-181a-5p | MIMAT0000256 | ELAVL2 | NM_001171195 | 1 | 1 | 1 | 0 | 1 | 4 |
| hsa-miR-181a-5p | MIMAT0000256 | STX2 | NM_194356 | 1 | 1 | 0 | 1 | 1 | 4 |
| hsa-miR-181a-5p | MIMAT0000256 | ETV6 | NM_001987 | 1 | 1 | 1 | 0 | 1 | 4 |
| hsa-miR-181a-5p | MIMAT0000256 | F5 | NM_000130 | 1 | 1 | 1 | 0 | 1 | 4 |
| hsa-miR-181a-5p | MIMAT0000256 | ACSL1 | NM_001995 | 1 | 1 | 1 | 0 | 1 | 4 |
| hsa-miR-181a-5p | MIMAT0000256 | FKTN | NM_001079802 | 1 | 1 | 0 | 1 | 1 | 4 |
| hsa-miR-181a-5p | MIMAT0000256 | FPR2 | NM_001462 | 1 | 1 | 0 | 1 | 1 | 4 |
| hsa-miR-181a-5p | MIMAT0000256 | GAB1 | NM_002039 | 1 | 1 | 1 | 0 | 1 | 4 |
| hsa-miR-181a-5p | MIMAT0000256 | HLA-E | NM_005516 | 1 | 1 | 0 | 1 | 1 | 4 |
| hsa-miR-181a-5p | MIMAT0000256 | HMGB3 | XM_005274667 | 1 | 1 | 0 | 1 | 1 | 4 |
| hsa-miR-181a-5p | MIMAT0000256 | IL2RB | NM_000878 | 1 | 1 | 0 | 1 | 1 | 4 |
| hsa-miR-181a-5p | MIMAT0000256 | IL7R | NM_002185 | 1 | 1 | 0 | 1 | 1 | 4 |
| hsa-miR-181a-5p | MIMAT0000256 | INCENP | NM_001040694 | 1 | 1 | 0 | 1 | 1 | 4 |
| hsa-miR-181a-5p | MIMAT0000256 | IRF5 | XM_005250317 | 1 | 1 | 0 | 1 | 1 | 4 |
| hsa-miR-181a-5p | MIMAT0000256 | KCNH1 | NM_172362 | 1 | 1 | 1 | 0 | 1 | 4 |
| hsa-miR-181a-5p | MIMAT0000256 | KCNN3 | NM_001204087 | 1 | 1 | 1 | 0 | 1 | 4 |
| hsa-miR-181a-5p | MIMAT0000256 | KPNB1 | NM_002265 | 1 | 1 | 1 | 0 | 1 | 4 |
| hsa-miR-181a-5p | MIMAT0000256 | CYP4F3 | NM_000896 | 1 | 1 | 0 | 1 | 1 | 4 |
| hsa-miR-181a-5p | MIMAT0000256 | LTBP2 | NM_000428 | 1 | 1 | 0 | 1 | 1 | 4 |
| hsa-miR-181a-5p | MIMAT0000256 | CAPRIN1 | NM_005898 | 1 | 1 | 1 | 0 | 1 | 4 |
| hsa-miR-181a-5p | MIMAT0000256 | SMAD3 | XM_005254383 | 1 | 1 | 0 | 1 | 1 | 4 |
| hsa-miR-181a-5p | MIMAT0000256 | MAGEA4 | XM_005274677 | 1 | 1 | 0 | 1 | 1 | 4 |
| hsa-miR-181a-5p | MIMAT0000256 | MDM2 | NM_002392 | 1 | 1 | 0 | 1 | 1 | 4 |
| hsa-miR-181a-5p | MIMAT0000256 | MGAT5 | XM_005263666 | 1 | 1 | 0 | 1 | 1 | 4 |
| hsa-miR-181a-5p | MIMAT0000256 | MTAP | NM_002451 | 1 | 1 | 0 | 1 | 1 | 4 |
| hsa-miR-181a-5p | MIMAT0000256 | NBN | NM_002485 | 1 | 1 | 0 | 1 | 1 | 4 |
| hsa-miR-181a-5p | MIMAT0000256 | NEK2 | NM_001204182 | 1 | 1 | 1 | 0 | 1 | 4 |
| hsa-miR-181a-5p | MIMAT0000256 | NFATC4 | NM_001136022 | 1 | 1 | 0 | 1 | 1 | 4 |
| hsa-miR-181a-5p | MIMAT0000256 | NKTR | XM_005265177 | 1 | 1 | 0 | 1 | 1 | 4 |
| hsa-miR-181a-5p | MIMAT0000256 | CNOT2 | NM_001199303 | 1 | 1 | 1 | 0 | 1 | 4 |
| hsa-miR-181a-5p | MIMAT0000256 | NOTCH4 | NM_004557 | 1 | 1 | 1 | 0 | 1 | 4 |
| hsa-miR-181a-5p | MIMAT0000256 | NSF | NM_006178 | 1 | 1 | 0 | 1 | 1 | 4 |
| hsa-miR-181a-5p | MIMAT0000256 | DDR2 | XM_005245221 | 1 | 1 | 0 | 1 | 1 | 4 |
| hsa-miR-181a-5p | MIMAT0000256 | OPRK1 | XM_005251252 | 1 | 1 | 0 | 1 | 1 | 4 |
| hsa-miR-181a-5p | MIMAT0000256 | PEBP1 | NM_002567 | 1 | 1 | 0 | 1 | 1 | 4 |
| hsa-miR-181a-5p | MIMAT0000256 | PAM | NM_000919 | 1 | 1 | 1 | 0 | 1 | 4 |
| hsa-miR-181a-5p | MIMAT0000256 | PDK1 | NM_002610 | 1 | 1 | 1 | 0 | 1 | 4 |
| hsa-miR-181a-5p | MIMAT0000256 | PFAS | NM_012393 | 1 | 1 | 0 | 1 | 1 | 4 |
| hsa-miR-181a-5p | MIMAT0000256 | SERPINA1 | NM_001127701 | 1 | 1 | 1 | 0 | 1 | 4 |
| hsa-miR-181a-5p | MIMAT0000256 | SERPINB9 | NM_004155 | 1 | 1 | 0 | 1 | 1 | 4 |
| hsa-miR-181a-5p | MIMAT0000256 | PLG | NM_000301 | 1 | 1 | 1 | 0 | 1 | 4 |
| hsa-miR-181a-5p | MIMAT0000256 | POLR2K | NM_005034 | 1 | 1 | 0 | 1 | 1 | 4 |
| hsa-miR-181a-5p | MIMAT0000256 | PPP2R5E | NM_006246 | 1 | 1 | 1 | 0 | 1 | 4 |
| hsa-miR-181a-5p | MIMAT0000256 | PRH2 | NM_005042 | 1 | 1 | 1 | 0 | 1 | 4 |
| hsa-miR-181a-5p | MIMAT0000256 | PRKCA | NM_002737 | 1 | 1 | 0 | 1 | 1 | 4 |
| hsa-miR-181a-5p | MIMAT0000256 | MASP1 | NM_001031849 | 1 | 1 | 1 | 0 | 1 | 4 |
| hsa-miR-181a-5p | MIMAT0000256 | PSG5 | NM_002781 | 1 | 1 | 1 | 0 | 1 | 4 |
| hsa-miR-181a-5p | MIMAT0000256 | PSG9 | NM_002784 | 1 | 1 | 1 | 0 | 1 | 4 |
| hsa-miR-181a-5p | MIMAT0000256 | PWP2 | NM_005049 | 1 | 1 | 0 | 1 | 1 | 4 |
| hsa-miR-181a-5p | MIMAT0000256 | PEX19 | NM_002857 | 1 | 1 | 0 | 1 | 1 | 4 |
| hsa-miR-181a-5p | MIMAT0000256 | RANGAP1 | NM_001278651 | 1 | 1 | 0 | 1 | 1 | 4 |
| hsa-miR-181a-5p | MIMAT0000256 | RAP1B | NM_001251922 | 1 | 1 | 1 | 0 | 1 | 4 |
| hsa-miR-181a-5p | MIMAT0000256 | PRPH2 | NM_000322 | 1 | 1 | 0 | 1 | 1 | 4 |
| hsa-miR-181a-5p | MIMAT0000256 | RECQL | NM_002907 | 1 | 1 | 0 | 1 | 1 | 4 |
| hsa-miR-181a-5p | MIMAT0000256 | RFC3 | NM_002915 | 1 | 1 | 0 | 1 | 1 | 4 |
| hsa-miR-181a-5p | MIMAT0000256 | RGS16 | NM_002928 | 1 | 1 | 0 | 1 | 1 | 4 |
| hsa-miR-181a-5p | MIMAT0000256 | RNF6 | NM_183043 | 1 | 1 | 1 | 0 | 1 | 4 |
| hsa-miR-181a-5p | MIMAT0000256 | RPL28 | NM_001136134 | 1 | 1 | 0 | 1 | 1 | 4 |
| hsa-miR-181a-5p | MIMAT0000256 | RPS27A | NM_002954 | 1 | 1 | 0 | 1 | 1 | 4 |
| hsa-miR-181a-5p | MIMAT0000256 | CCL22 | NM_002990 | 1 | 1 | 0 | 1 | 1 | 4 |
| hsa-miR-181a-5p | MIMAT0000256 | CXCL5 | NM_002994 | 1 | 1 | 0 | 1 | 1 | 4 |
| hsa-miR-181a-5p | MIMAT0000256 | MAP2K4 | NM_001281435 | 1 | 1 | 0 | 1 | 1 | 4 |
| hsa-miR-181a-5p | MIMAT0000256 | SH3BP2 | NM_001145855 | 1 | 1 | 0 | 1 | 1 | 4 |
| hsa-miR-181a-5p | MIMAT0000256 | SLC3A1 | NM_000341 | 1 | 1 | 1 | 0 | 1 | 4 |
| hsa-miR-181a-5p | MIMAT0000256 | SLC6A2 | NM_001172504 | 1 | 1 | 1 | 0 | 1 | 4 |
| hsa-miR-181a-5p | MIMAT0000256 | SLC6A4 | NM_001045 | 1 | 1 | 0 | 1 | 1 | 4 |
| hsa-miR-181a-5p | MIMAT0000256 | SLC11A1 | NM_000578 | 1 | 1 | 1 | 0 | 1 | 4 |
| hsa-miR-181a-5p | MIMAT0000256 | SUMO3 | XM_005261165 | 1 | 1 | 0 | 1 | 1 | 4 |
| hsa-miR-181a-5p | MIMAT0000256 | SIGLEC1 | XM_005260807 | 1 | 1 | 0 | 1 | 1 | 4 |
| hsa-miR-181a-5p | MIMAT0000256 | SNAPC3 | NM_001039697 | 1 | 1 | 1 | 0 | 1 | 4 |
| hsa-miR-181a-5p | MIMAT0000256 | SORD | NM_003104 | 1 | 1 | 1 | 0 | 1 | 4 |
| hsa-miR-181a-5p | MIMAT0000256 | SRPK2 | NM_182692 | 1 | 1 | 1 | 0 | 1 | 4 |
| hsa-miR-181a-5p | MIMAT0000256 | SSB | NM_003142 | 1 | 1 | 1 | 0 | 1 | 4 |
| hsa-miR-181a-5p | MIMAT0000256 | TAP2 | NM_000544 | 1 | 1 | 1 | 0 | 1 | 4 |
| hsa-miR-181a-5p | MIMAT0000256 | TBCD | XM_005256395 | 1 | 1 | 0 | 1 | 1 | 4 |
| hsa-miR-181a-5p | MIMAT0000256 | TCEB1 | NM_001204858 | 1 | 1 | 0 | 1 | 1 | 4 |
| hsa-miR-181a-5p | MIMAT0000256 | HNF1B | NM_000458 | 1 | 1 | 0 | 1 | 1 | 4 |
| hsa-miR-181a-5p | MIMAT0000256 | TCF7L2 | NM_001198530 | 1 | 1 | 1 | 0 | 1 | 4 |
| hsa-miR-181a-5p | MIMAT0000256 | TFPI | XM_005246818 | 1 | 1 | 0 | 1 | 1 | 4 |
| hsa-miR-181a-5p | MIMAT0000256 | TGFA | XM_005264522 | 1 | 1 | 0 | 1 | 1 | 4 |
| hsa-miR-181a-5p | MIMAT0000256 | TIA1 | NM_022037 | 1 | 1 | 1 | 0 | 1 | 4 |
| hsa-miR-181a-5p | MIMAT0000256 | TK2 | NM_001271934 | 1 | 1 | 0 | 1 | 1 | 4 |
| hsa-miR-181a-5p | MIMAT0000256 | TLL1 | NM_012464 | 1 | 1 | 1 | 0 | 1 | 4 |
| hsa-miR-181a-5p | MIMAT0000256 | TLR4 | NM_138554 | 1 | 1 | 1 | 0 | 1 | 4 |
| hsa-miR-181a-5p | MIMAT0000256 | TNFAIP1 | NM_021137 | 1 | 1 | 1 | 0 | 1 | 4 |
| hsa-miR-181a-5p | MIMAT0000256 | TNFAIP6 | NM_007115 | 1 | 1 | 1 | 0 | 1 | 4 |
| hsa-miR-181a-5p | MIMAT0000256 | NR2C2 | NM_003298 | 1 | 1 | 1 | 0 | 1 | 4 |
| hsa-miR-181a-5p | MIMAT0000256 | HSP90B1 | NM_003299 | 1 | 1 | 1 | 0 | 1 | 4 |
| hsa-miR-181a-5p | MIMAT0000256 | TRPC6 | NM_004621 | 1 | 1 | 0 | 1 | 1 | 4 |
| hsa-miR-181a-5p | MIMAT0000256 | TSG101 | XM_005253108 | 1 | 1 | 0 | 1 | 1 | 4 |
| hsa-miR-181a-5p | MIMAT0000256 | TUFM | NM_003321 | 1 | 1 | 0 | 1 | 1 | 4 |
| hsa-miR-181a-5p | MIMAT0000256 | TNFSF4 | NM_003326 | 1 | 1 | 1 | 0 | 1 | 4 |
| hsa-miR-181a-5p | MIMAT0000256 | UBE2D1 | NM_003338 | 1 | 1 | 1 | 0 | 1 | 4 |
| hsa-miR-181a-5p | MIMAT0000256 | VHL | NM_000551 | 1 | 1 | 1 | 0 | 1 | 4 |
| hsa-miR-181a-5p | MIMAT0000256 | WFS1 | NM_006005 | 1 | 1 | 0 | 1 | 1 | 4 |
| hsa-miR-181a-5p | MIMAT0000256 | XG | XM_005274587 | 1 | 1 | 0 | 1 | 1 | 4 |
| hsa-miR-181a-5p | MIMAT0000256 | ZNF20 | NM_021143 | 1 | 1 | 1 | 0 | 1 | 4 |
| hsa-miR-181a-5p | MIMAT0000256 | ZNF84 | XM_005266185 | 1 | 1 | 0 | 1 | 1 | 4 |
| hsa-miR-181a-5p | MIMAT0000256 | ZNF124 | NM_003431 | 1 | 1 | 1 | 0 | 1 | 4 |
| hsa-miR-181a-5p | MIMAT0000256 | ZNF217 | NM_006526 | 1 | 1 | 1 | 0 | 1 | 4 |
| hsa-miR-181a-5p | MIMAT0000256 | MOGS | NM_006302 | 1 | 1 | 0 | 1 | 1 | 4 |
| hsa-miR-181a-5p | MIMAT0000256 | ZNF212 | NM_012256 | 1 | 1 | 0 | 1 | 1 | 4 |
| hsa-miR-181a-5p | MIMAT0000256 | TAF15 | NM_139215 | 1 | 1 | 0 | 1 | 1 | 4 |
| hsa-miR-181a-5p | MIMAT0000256 | SMC1A | NM_001281463 | 1 | 1 | 0 | 1 | 1 | 4 |
| hsa-miR-181a-5p | MIMAT0000256 | BRAP | NM_006768 | 1 | 1 | 1 | 0 | 1 | 4 |
| hsa-miR-181a-5p | MIMAT0000256 | ULK1 | NM_003565 | 1 | 1 | 0 | 1 | 1 | 4 |
| hsa-miR-181a-5p | MIMAT0000256 | RECK | NM_021111 | 1 | 1 | 1 | 0 | 1 | 4 |
| hsa-miR-181a-5p | MIMAT0000256 | CYP4F2 | NM_001082 | 1 | 1 | 0 | 1 | 1 | 4 |
| hsa-miR-181a-5p | MIMAT0000256 | KMO | NM_003679 | 1 | 1 | 0 | 1 | 1 | 4 |
| hsa-miR-181a-5p | MIMAT0000256 | USO1 | NM_003715 | 1 | 1 | 1 | 0 | 1 | 4 |
| hsa-miR-181a-5p | MIMAT0000256 | TP63 | NM_001114981 | 1 | 1 | 1 | 0 | 1 | 4 |
| hsa-miR-181a-5p | MIMAT0000256 | TNFSF14 | XM_005259670 | 1 | 1 | 0 | 1 | 1 | 4 |
| hsa-miR-181a-5p | MIMAT0000256 | SUCLG2 | NM_003848 | 1 | 1 | 1 | 0 | 1 | 4 |
| hsa-miR-181a-5p | MIMAT0000256 | IL18R1 | NM_003855 | 1 | 1 | 0 | 1 | 1 | 4 |
| hsa-miR-181a-5p | MIMAT0000256 | KAT2B | NM_003884 | 1 | 1 | 1 | 0 | 1 | 4 |
| hsa-miR-181a-5p | MIMAT0000256 | MTMR3 | NM_021090 | 1 | 1 | 0 | 1 | 1 | 4 |
| hsa-miR-181a-5p | MIMAT0000256 | PRPF4B | NM_003913 | 1 | 1 | 0 | 1 | 1 | 4 |
| hsa-miR-181a-5p | MIMAT0000256 | AP3D1 | NM_001261826 | 1 | 1 | 0 | 1 | 1 | 4 |
| hsa-miR-181a-5p | MIMAT0000256 | TMSB4Y | NM_004202 | 1 | 1 | 0 | 1 | 1 | 4 |
| hsa-miR-181a-5p | MIMAT0000256 | NREP | NM_001142483 | 1 | 1 | 0 | 1 | 1 | 4 |
| hsa-miR-181a-5p | MIMAT0000256 | KL | NM_004795 | 1 | 1 | 0 | 1 | 1 | 4 |
| hsa-miR-181a-5p | MIMAT0000256 | ADIPOQ | NM_001177800 | 1 | 1 | 0 | 1 | 1 | 4 |
| hsa-miR-181a-5p | MIMAT0000256 | QKI | NM_206855 | 1 | 1 | 0 | 1 | 1 | 4 |
| hsa-miR-181a-5p | MIMAT0000256 | AKAP5 | NM_004857 | 1 | 1 | 1 | 0 | 1 | 4 |
| hsa-miR-181a-5p | MIMAT0000256 | EEF1E1 | XM_005249486 | 1 | 1 | 0 | 1 | 1 | 4 |
| hsa-miR-181a-5p | MIMAT0000256 | BAG2 | NM_004282 | 0 | 1 | 1 | 1 | 1 | 4 |
| hsa-miR-181a-5p | MIMAT0000256 | MTL5 | NM_001039656 | 1 | 1 | 0 | 1 | 1 | 4 |
| hsa-miR-181a-5p | MIMAT0000256 | ZFYVE16 | NM_001105251 | 1 | 1 | 1 | 0 | 1 | 4 |
| hsa-miR-181a-5p | MIMAT0000256 | RAPGEF5 | XM_005249914 | 1 | 1 | 0 | 1 | 1 | 4 |
| hsa-miR-181a-5p | MIMAT0000256 | GAB2 | NM_080491 | 1 | 1 | 0 | 1 | 1 | 4 |
| hsa-miR-181a-5p | MIMAT0000256 | C2CD5 | NM_014802 | 1 | 1 | 1 | 0 | 1 | 4 |
| hsa-miR-181a-5p | MIMAT0000256 | OSBPL2 | XM_005260624 | 1 | 1 | 0 | 1 | 1 | 4 |
| hsa-miR-181a-5p | MIMAT0000256 | HS3ST2 | NM_006043 | 1 | 1 | 0 | 1 | 1 | 4 |
| hsa-miR-181a-5p | MIMAT0000256 | DGCR2 | NM_005137 | 1 | 1 | 0 | 1 | 1 | 4 |
| hsa-miR-181a-5p | MIMAT0000256 | TOM1L1 | NM_005486 | 1 | 1 | 1 | 0 | 1 | 4 |
| hsa-miR-181a-5p | MIMAT0000256 | ARPC1B | XM_005250097 | 1 | 1 | 0 | 1 | 1 | 4 |
| hsa-miR-181a-5p | MIMAT0000256 | PDIA6 | NM_005742 | 1 | 1 | 0 | 1 | 1 | 4 |
| hsa-miR-181a-5p | MIMAT0000256 | FAM13A | NM_014883 | 1 | 1 | 0 | 1 | 1 | 4 |
| hsa-miR-181a-5p | MIMAT0000256 | RASA4 | NM_006989 | 1 | 1 | 0 | 1 | 1 | 4 |
| hsa-miR-181a-5p | MIMAT0000256 | PRSS16 | NM_005865 | 1 | 1 | 1 | 0 | 1 | 4 |
| hsa-miR-181a-5p | MIMAT0000256 | PCGF3 | XM_005272253 | 1 | 1 | 0 | 1 | 1 | 4 |
| hsa-miR-181a-5p | MIMAT0000256 | TRDN | NM_001251987 | 1 | 1 | 1 | 0 | 1 | 4 |
| hsa-miR-181a-5p | MIMAT0000256 | TIMM17A | NM_006335 | 0 | 1 | 1 | 1 | 1 | 4 |
| hsa-miR-181a-5p | MIMAT0000256 | POLR3F | NM_006466 | 1 | 1 | 0 | 1 | 1 | 4 |
| hsa-miR-181a-5p | MIMAT0000256 | FUT9 | NM_006581 | 1 | 1 | 1 | 0 | 1 | 4 |
| hsa-miR-181a-5p | MIMAT0000256 | TCERG1 | NM_001040006 | 1 | 1 | 1 | 0 | 1 | 4 |
| hsa-miR-181a-5p | MIMAT0000256 | RAB40B | NM_006822 | 1 | 1 | 1 | 0 | 1 | 4 |
| hsa-miR-181a-5p | MIMAT0000256 | LILRB3 | XM_005277289 | 1 | 1 | 0 | 1 | 1 | 4 |
| hsa-miR-181a-5p | MIMAT0000256 | CIT | NM_001206999 | 1 | 1 | 0 | 1 | 1 | 4 |
| hsa-miR-181a-5p | MIMAT0000256 | POLI | NM_007195 | 1 | 1 | 1 | 0 | 1 | 4 |
| hsa-miR-181a-5p | MIMAT0000256 | GPR176 | NM_007223 | 1 | 1 | 0 | 1 | 1 | 4 |
| hsa-miR-181a-5p | MIMAT0000256 | TFEC | NM_001244583 | 1 | 1 | 1 | 0 | 1 | 4 |
| hsa-miR-181a-5p | MIMAT0000256 | RAB3GAP1 | NM_001172435 | 0 | 1 | 1 | 1 | 1 | 4 |
| hsa-miR-181a-5p | MIMAT0000256 | GOLGA8A | XM_005254239 | 1 | 1 | 0 | 1 | 1 | 4 |
| hsa-miR-181a-5p | MIMAT0000256 | EMC1 | NM_015047 | 1 | 1 | 0 | 1 | 1 | 4 |
| hsa-miR-181a-5p | MIMAT0000256 | RRP1B | NM_015056 | 1 | 1 | 0 | 1 | 1 | 4 |
| hsa-miR-181a-5p | MIMAT0000256 | FAM179B | NM_015091 | 1 | 1 | 1 | 0 | 1 | 4 |
| hsa-miR-181a-5p | MIMAT0000256 | N4BP3 | NM_015111 | 1 | 1 | 0 | 1 | 1 | 4 |
| hsa-miR-181a-5p | MIMAT0000256 | PRRC2C | NM_015172 | 1 | 1 | 1 | 0 | 1 | 4 |
| hsa-miR-181a-5p | MIMAT0000256 | TTC28 | NM_001145418 | 1 | 1 | 0 | 1 | 1 | 4 |
| hsa-miR-181a-5p | MIMAT0000256 | WDR7 | NM_015285 | 1 | 1 | 0 | 1 | 1 | 4 |
| hsa-miR-181a-5p | MIMAT0000256 | SYNE1 | NM_182961 | 1 | 1 | 1 | 0 | 1 | 4 |
| hsa-miR-181a-5p | MIMAT0000256 | KIAA1045 | NM_015297 | 1 | 1 | 0 | 1 | 1 | 4 |
| hsa-miR-181a-5p | MIMAT0000256 | ARHGEF12 | NM_015313 | 1 | 1 | 0 | 1 | 1 | 4 |
| hsa-miR-181a-5p | MIMAT0000256 | NCS1 | NM_014286 | 1 | 1 | 0 | 1 | 1 | 4 |
| hsa-miR-181a-5p | MIMAT0000256 | HEY2 | NM_012259 | 1 | 1 | 1 | 0 | 1 | 4 |
| hsa-miR-181a-5p | MIMAT0000256 | ARL2BP | NM_012106 | 0 | 1 | 1 | 1 | 1 | 4 |
| hsa-miR-181a-5p | MIMAT0000256 | NPAP1 | NM_018958 | 1 | 1 | 1 | 0 | 1 | 4 |
| hsa-miR-181a-5p | MIMAT0000256 | DAK | NM_015533 | 1 | 1 | 0 | 1 | 1 | 4 |
| hsa-miR-181a-5p | MIMAT0000256 | FAM169A | XM_005248479 | 1 | 1 | 0 | 1 | 1 | 4 |
| hsa-miR-181a-5p | MIMAT0000256 | DCAF4 | NM_015604 | 1 | 1 | 0 | 1 | 1 | 4 |
| hsa-miR-181a-5p | MIMAT0000256 | FBXL3 | NM_012158 | 1 | 1 | 1 | 0 | 1 | 4 |
| hsa-miR-181a-5p | MIMAT0000256 | EHF | NM_001206615 | 1 | 1 | 1 | 0 | 1 | 4 |
| hsa-miR-181a-5p | MIMAT0000256 | LATS2 | XM_005266342 | 1 | 1 | 0 | 1 | 1 | 4 |
| hsa-miR-181a-5p | MIMAT0000256 | PPP2R3B | XM_005274428 | 1 | 1 | 0 | 1 | 1 | 4 |
| hsa-miR-181a-5p | MIMAT0000256 | MCTS1 | NM_014060 | 1 | 1 | 1 | 0 | 1 | 4 |
| hsa-miR-181a-5p | MIMAT0000256 | ZBTB44 | NM_014155 | 1 | 1 | 1 | 0 | 1 | 4 |
| hsa-miR-181a-5p | MIMAT0000256 | ARHGEF3 | NM_001128616 | 1 | 1 | 1 | 0 | 1 | 4 |
| hsa-miR-181a-5p | MIMAT0000256 | PDE11A | NM_016953 | 1 | 1 | 0 | 1 | 1 | 4 |
| hsa-miR-181a-5p | MIMAT0000256 | SLC35C2 | NM_001281460 | 1 | 1 | 0 | 1 | 1 | 4 |
| hsa-miR-181a-5p | MIMAT0000256 | MRPS16 | NM_016065 | 0 | 1 | 1 | 1 | 1 | 4 |
| hsa-miR-181a-5p | MIMAT0000256 | RMDN1 | NM_016033 | 0 | 1 | 1 | 1 | 1 | 4 |
| hsa-miR-181a-5p | MIMAT0000256 | ZDHHC3 | NM_016598 | 1 | 1 | 1 | 0 | 1 | 4 |
| hsa-miR-181a-5p | MIMAT0000256 | FAM13B | NM_001101801 | 1 | 1 | 1 | 0 | 1 | 4 |
| hsa-miR-181a-5p | MIMAT0000256 | KLRF1 | NM_016523 | 1 | 1 | 0 | 1 | 1 | 4 |
| hsa-miR-181a-5p | MIMAT0000256 | WNT16 | NM_057168 | 1 | 1 | 1 | 0 | 1 | 4 |
| hsa-miR-181a-5p | MIMAT0000256 | ZAK | NM_016653 | 1 | 1 | 1 | 0 | 1 | 4 |
| hsa-miR-181a-5p | MIMAT0000256 | BCL11A | NM_022893 | 0 | 1 | 1 | 1 | 1 | 4 |
| hsa-miR-181a-5p | MIMAT0000256 | SHC3 | NM_016848 | 1 | 1 | 1 | 0 | 1 | 4 |
| hsa-miR-181a-5p | MIMAT0000256 | NDFIP2 | NM_019080 | 1 | 1 | 0 | 1 | 1 | 4 |
| hsa-miR-181a-5p | MIMAT0000256 | INO80 | NM_017553 | 0 | 1 | 1 | 1 | 1 | 4 |
| hsa-miR-181a-5p | MIMAT0000256 | EPDR1 | NM_017549 | 1 | 1 | 0 | 1 | 1 | 4 |
| hsa-miR-181a-5p | MIMAT0000256 | ZRANB1 | NM_017580 | 1 | 1 | 1 | 0 | 1 | 4 |
| hsa-miR-181a-5p | MIMAT0000256 | TET2 | NM_017628 | 1 | 1 | 1 | 0 | 1 | 4 |
| hsa-miR-181a-5p | MIMAT0000256 | VPS13C | NM_001018088 | 1 | 1 | 1 | 0 | 1 | 4 |
| hsa-miR-181a-5p | MIMAT0000256 | ARHGEF38 | NM_001242729 | 0 | 1 | 1 | 1 | 1 | 4 |
| hsa-miR-181a-5p | MIMAT0000256 | TOR4A | NM_017723 | 1 | 1 | 0 | 1 | 1 | 4 |
| hsa-miR-181a-5p | MIMAT0000256 | ZCCHC2 | NM_017742 | 1 | 1 | 0 | 1 | 1 | 4 |
| hsa-miR-181a-5p | MIMAT0000256 | MTMR10 | NM_017762 | 1 | 1 | 0 | 1 | 1 | 4 |
| hsa-miR-181a-5p | MIMAT0000256 | OCIAD1 | NM_001079839 | 1 | 1 | 1 | 0 | 1 | 4 |
| hsa-miR-181a-5p | MIMAT0000256 | TRNAU1AP | NM_017846 | 1 | 1 | 0 | 1 | 1 | 4 |
| hsa-miR-181a-5p | MIMAT0000256 | 2-Mar | NM_017898 | 1 | 1 | 1 | 0 | 1 | 4 |
| hsa-miR-181a-5p | MIMAT0000256 | PTCD3 | NM_017952 | 1 | 1 | 0 | 1 | 1 | 4 |
| hsa-miR-181a-5p | MIMAT0000256 | SOBP | NM_018013 | 1 | 1 | 1 | 0 | 1 | 4 |
| hsa-miR-181a-5p | MIMAT0000256 | FIGN | NM_018086 | 1 | 1 | 1 | 0 | 1 | 4 |
| hsa-miR-181a-5p | MIMAT0000256 | PBRM1 | XM_005265276 | 1 | 1 | 0 | 1 | 1 | 4 |
| hsa-miR-181a-5p | MIMAT0000256 | TMEM40 | NM_018306 | 1 | 1 | 0 | 1 | 1 | 4 |
| hsa-miR-181a-5p | MIMAT0000256 | SPTLC3 | NM_018327 | 1 | 1 | 0 | 1 | 1 | 4 |
| hsa-miR-181a-5p | MIMAT0000256 | AP5S1 | NM_001204446 | 0 | 1 | 1 | 1 | 1 | 4 |
| hsa-miR-181a-5p | MIMAT0000256 | MCM10 | NM_182751 | 1 | 1 | 0 | 1 | 1 | 4 |
| hsa-miR-181a-5p | MIMAT0000256 | BMP2K | NM_017593 | 1 | 1 | 1 | 0 | 1 | 4 |
| hsa-miR-181a-5p | MIMAT0000256 | CDKN2AIP | XM_005263118 | 1 | 1 | 0 | 1 | 1 | 4 |
| hsa-miR-181a-5p | MIMAT0000256 | KRBOX4 | NM_001129899 | 0 | 1 | 1 | 1 | 1 | 4 |
| hsa-miR-181a-5p | MIMAT0000256 | CHD7 | NM_017780 | 1 | 1 | 0 | 1 | 1 | 4 |
| hsa-miR-181a-5p | MIMAT0000256 | CCAR1 | NM_018237 | 1 | 1 | 1 | 0 | 1 | 4 |
| hsa-miR-181a-5p | MIMAT0000256 | TXLNG | NM_018360 | 1 | 1 | 0 | 1 | 1 | 4 |
| hsa-miR-181a-5p | MIMAT0000256 | LRP2BP | NM_018409 | 1 | 1 | 0 | 1 | 1 | 4 |
| hsa-miR-181a-5p | MIMAT0000256 | TMEM165 | NM_018475 | 0 | 1 | 1 | 1 | 1 | 4 |
| hsa-miR-181a-5p | MIMAT0000256 | PCDHA11 | XM_005268489 | 1 | 1 | 0 | 1 | 1 | 4 |
| hsa-miR-181a-5p | MIMAT0000256 | GPCPD1 | NM_019593 | 1 | 1 | 1 | 0 | 1 | 4 |
| hsa-miR-181a-5p | MIMAT0000256 | WDR45B | NM_019613 | 1 | 1 | 0 | 1 | 1 | 4 |
| hsa-miR-181a-5p | MIMAT0000256 | UGGT1 | NM_020120 | 1 | 1 | 0 | 1 | 1 | 4 |
| hsa-miR-181a-5p | MIMAT0000256 | C1GALT1 | XM_005249812 | 1 | 1 | 0 | 1 | 1 | 4 |
| hsa-miR-181a-5p | MIMAT0000256 | DHX33 | NM_020162 | 1 | 1 | 0 | 1 | 1 | 4 |
| hsa-miR-181a-5p | MIMAT0000256 | SLC4A10 | NM_001178016 | 0 | 1 | 1 | 1 | 1 | 4 |
| hsa-miR-181a-5p | MIMAT0000256 | IFT80 | XM_005247675 | 1 | 1 | 0 | 1 | 1 | 4 |
| hsa-miR-181a-5p | MIMAT0000256 | FAM135A | NM_020819 | 1 | 1 | 1 | 0 | 1 | 4 |
| hsa-miR-181a-5p | MIMAT0000256 | USP36 | XM_005257543 | 1 | 1 | 0 | 1 | 1 | 4 |
| hsa-miR-181a-5p | MIMAT0000256 | USP37 | XM_005246720 | 1 | 1 | 0 | 1 | 1 | 4 |
| hsa-miR-181a-5p | MIMAT0000256 | SEMA4G | NM_017893 | 1 | 1 | 1 | 0 | 1 | 4 |
| hsa-miR-181a-5p | MIMAT0000256 | PAPPA2 | XM_005245422 | 1 | 1 | 0 | 1 | 1 | 4 |
| hsa-miR-181a-5p | MIMAT0000256 | SH2D4A | NM_022071 | 1 | 1 | 1 | 0 | 1 | 4 |
| hsa-miR-181a-5p | MIMAT0000256 | PRDM15 | NM_001040424 | 1 | 1 | 0 | 1 | 1 | 4 |
| hsa-miR-181a-5p | MIMAT0000256 | RBM26 | NM_022118 | 1 | 1 | 1 | 0 | 1 | 4 |
| hsa-miR-181a-5p | MIMAT0000256 | PCNXL4 | NM_022495 | 1 | 1 | 1 | 0 | 1 | 4 |
| hsa-miR-181a-5p | MIMAT0000256 | NOM1 | NM_138400 | 1 | 1 | 0 | 1 | 1 | 4 |
| hsa-miR-181a-5p | MIMAT0000256 | CREB3L2 | NM_194071 | 1 | 1 | 0 | 1 | 1 | 4 |
| hsa-miR-181a-5p | MIMAT0000256 | TTC23 | XM_005254969 | 1 | 1 | 0 | 1 | 1 | 4 |
| hsa-miR-181a-5p | MIMAT0000256 | PCDH15 | NM_001142765 | 1 | 1 | 1 | 0 | 1 | 4 |
| hsa-miR-181a-5p | MIMAT0000256 | TMEM108 | XM_005247728 | 1 | 1 | 0 | 1 | 1 | 4 |
| hsa-miR-181a-5p | MIMAT0000256 | PRRG4 | NM_024081 | 1 | 1 | 1 | 0 | 1 | 4 |
| hsa-miR-181a-5p | MIMAT0000256 | C1orf116 | NM_023938 | 1 | 1 | 0 | 1 | 1 | 4 |
| hsa-miR-181a-5p | MIMAT0000256 | CHCHD7 | NM_001011667 | 1 | 1 | 1 | 0 | 1 | 4 |
| hsa-miR-181a-5p | MIMAT0000256 | MUL1 | XM_005246001 | 1 | 1 | 0 | 1 | 1 | 4 |
| hsa-miR-181a-5p | MIMAT0000256 | RIC3 | NM_001206671 | 1 | 1 | 0 | 1 | 1 | 4 |
| hsa-miR-181a-5p | MIMAT0000256 | SH3TC2 | NM_024577 | 1 | 1 | 1 | 0 | 1 | 4 |
| hsa-miR-181a-5p | MIMAT0000256 | ARSJ | NM_024590 | 1 | 1 | 1 | 0 | 1 | 4 |
| hsa-miR-181a-5p | MIMAT0000256 | ZNF750 | NM_024702 | 1 | 1 | 0 | 1 | 1 | 4 |
| hsa-miR-181a-5p | MIMAT0000256 | KLHL36 | XM_005256149 | 1 | 1 | 0 | 1 | 1 | 4 |
| hsa-miR-181a-5p | MIMAT0000256 | CLMN | NM_024734 | 1 | 1 | 1 | 0 | 1 | 4 |
| hsa-miR-181a-5p | MIMAT0000256 | RPAP2 | NM_024813 | 1 | 1 | 1 | 0 | 1 | 4 |
| hsa-miR-181a-5p | MIMAT0000256 | CBLL1 | NM_024814 | 1 | 1 | 1 | 0 | 1 | 4 |
| hsa-miR-181a-5p | MIMAT0000256 | PHC3 | NM_024947 | 1 | 1 | 1 | 0 | 1 | 4 |
| hsa-miR-181a-5p | MIMAT0000256 | WWC2 | NM_024949 | 1 | 1 | 0 | 1 | 1 | 4 |
| hsa-miR-181a-5p | MIMAT0000256 | PGAP1 | NM_024989 | 1 | 1 | 1 | 0 | 1 | 4 |
| hsa-miR-181a-5p | MIMAT0000256 | ZNF606 | NM_025027 | 0 | 1 | 1 | 1 | 1 | 4 |
| hsa-miR-181a-5p | MIMAT0000256 | IFT74 | NM_001099222 | 1 | 1 | 1 | 0 | 1 | 4 |
| hsa-miR-181a-5p | MIMAT0000256 | ZBP1 | NM_030776 | 1 | 1 | 0 | 1 | 1 | 4 |
| hsa-miR-181a-5p | MIMAT0000256 | GFOD2 | NM_001243650 | 1 | 1 | 0 | 1 | 1 | 4 |
| hsa-miR-181a-5p | MIMAT0000256 | RNF170 | NM_001160223 | 1 | 1 | 0 | 1 | 1 | 4 |
| hsa-miR-181a-5p | MIMAT0000256 | SYT15 | NM_031912 | 1 | 1 | 1 | 0 | 1 | 4 |
| hsa-miR-181a-5p | MIMAT0000256 | MRO | NM_031939 | 1 | 1 | 0 | 1 | 1 | 4 |
| hsa-miR-181a-5p | MIMAT0000256 | KCTD10 | XM_005253945 | 1 | 1 | 0 | 1 | 1 | 4 |
| hsa-miR-181a-5p | MIMAT0000256 | FAM160A2 | NM_032127 | 1 | 1 | 1 | 0 | 1 | 4 |
| hsa-miR-181a-5p | MIMAT0000256 | SLC37A3 | XM_005250058 | 1 | 1 | 0 | 1 | 1 | 4 |
| hsa-miR-181a-5p | MIMAT0000256 | DCUN1D5 | NM_032299 | 1 | 1 | 1 | 0 | 1 | 4 |
| hsa-miR-181a-5p | MIMAT0000256 | EFCAB2 | NM_001143943 | 1 | 1 | 0 | 1 | 1 | 4 |
| hsa-miR-181a-5p | MIMAT0000256 | HOOK3 | NM_032410 | 1 | 1 | 1 | 0 | 1 | 4 |
| hsa-miR-181a-5p | MIMAT0000256 | ZNF527 | NM_032453 | 1 | 1 | 1 | 0 | 1 | 4 |
| hsa-miR-181a-5p | MIMAT0000256 | CCDC62 | NM_201435 | 0 | 1 | 1 | 1 | 1 | 4 |
| hsa-miR-181a-5p | MIMAT0000256 | PLEKHA8 | NM_001197026 | 1 | 1 | 1 | 0 | 1 | 4 |
| hsa-miR-181a-5p | MIMAT0000256 | MINA | NM_001042533 | 1 | 1 | 1 | 0 | 1 | 4 |
| hsa-miR-181a-5p | MIMAT0000256 | FIBCD1 | NM_032843 | 1 | 1 | 0 | 1 | 1 | 4 |
| hsa-miR-181a-5p | MIMAT0000256 | DOCK7 | NM_033407 | 1 | 1 | 1 | 0 | 1 | 4 |
| hsa-miR-181a-5p | MIMAT0000256 | SSH2 | XM_005258058 | 1 | 1 | 0 | 1 | 1 | 4 |
| hsa-miR-181a-5p | MIMAT0000256 | FRMD7 | NM_194277 | 1 | 1 | 1 | 0 | 1 | 4 |
| hsa-miR-181a-5p | MIMAT0000256 | BTF3L4 | NM_152265 | 1 | 1 | 0 | 1 | 1 | 4 |
| hsa-miR-181a-5p | MIMAT0000256 | ANKRD44 | NM_001195144 | 1 | 1 | 1 | 0 | 1 | 4 |
| hsa-miR-181a-5p | MIMAT0000256 | CABLES1 | NM_001100619 | 1 | 1 | 0 | 1 | 1 | 4 |
| hsa-miR-181a-5p | MIMAT0000256 | TMEM132C | NM_001136103 | 1 | 1 | 0 | 1 | 1 | 4 |
| hsa-miR-181a-5p | MIMAT0000256 | C17orf72 | NM_001164257 | 1 | 1 | 0 | 1 | 1 | 4 |
| hsa-miR-181a-5p | MIMAT0000256 | TADA2B | NM_152293 | 1 | 1 | 1 | 0 | 1 | 4 |
| hsa-miR-181a-5p | MIMAT0000256 | PAXBP1 | NM_016631 | 0 | 1 | 1 | 1 | 1 | 4 |
| hsa-miR-181a-5p | MIMAT0000256 | LARP4 | NM_001170803 | 1 | 1 | 1 | 0 | 1 | 4 |
| hsa-miR-181a-5p | MIMAT0000256 | ERMAP | NM_001017922 | 1 | 1 | 1 | 0 | 1 | 4 |
| hsa-miR-181a-5p | MIMAT0000256 | FMNL2 | NM_052905 | 1 | 1 | 1 | 0 | 1 | 4 |
| hsa-miR-181a-5p | MIMAT0000256 | MYSM1 | NM_001085487 | 1 | 1 | 1 | 0 | 1 | 4 |
| hsa-miR-181a-5p | MIMAT0000256 | OMA1 | XM_005270422 | 1 | 1 | 0 | 1 | 1 | 4 |
| hsa-miR-181a-5p | MIMAT0000256 | ARL11 | NM_138450 | 1 | 1 | 0 | 1 | 1 | 4 |
| hsa-miR-181a-5p | MIMAT0000256 | SH2D1B | NM_053282 | 1 | 1 | 0 | 1 | 1 | 4 |
| hsa-miR-181a-5p | MIMAT0000256 | PRR20A | NM_198441 | 1 | 1 | 1 | 0 | 1 | 4 |
| hsa-miR-181a-5p | MIMAT0000256 | ZNF440 | NM_152357 | 0 | 1 | 1 | 1 | 1 | 4 |
| hsa-miR-181a-5p | MIMAT0000256 | C22orf39 | NM_173793 | 1 | 1 | 0 | 1 | 1 | 4 |
| hsa-miR-181a-5p | MIMAT0000256 | MDH1B | NM_001039845 | 0 | 1 | 1 | 1 | 1 | 4 |
| hsa-miR-181a-5p | MIMAT0000256 | TMEM207 | NM_207316 | 0 | 1 | 1 | 1 | 1 | 4 |
| hsa-miR-181a-5p | MIMAT0000256 | WDR36 | NM_139281 | 0 | 1 | 1 | 1 | 1 | 4 |
| hsa-miR-181a-5p | MIMAT0000256 | AGPAT6 | XM_005273402 | 1 | 1 | 0 | 1 | 1 | 4 |
| hsa-miR-181a-5p | MIMAT0000256 | PABPC5 | NM_080832 | 1 | 1 | 1 | 0 | 1 | 4 |
| hsa-miR-181a-5p | MIMAT0000256 | XRRA1 | XM_005273768 | 1 | 1 | 0 | 1 | 1 | 4 |
| hsa-miR-181a-5p | MIMAT0000256 | PGPEP1L | NM_001167902 | 1 | 1 | 1 | 0 | 1 | 4 |
| hsa-miR-181a-5p | MIMAT0000256 | SLFN13 | NM_144682 | 1 | 1 | 0 | 1 | 1 | 4 |
| hsa-miR-181a-5p | MIMAT0000256 | ZNF418 | NM_133460 | 1 | 1 | 0 | 1 | 1 | 4 |
| hsa-miR-181a-5p | MIMAT0000256 | CEP120 | NM_001166226 | 1 | 1 | 1 | 0 | 1 | 4 |
| hsa-miR-181a-5p | MIMAT0000256 | VPS13B | XM_005250800 | 1 | 1 | 0 | 1 | 1 | 4 |
| hsa-miR-181a-5p | MIMAT0000256 | DGKH | NM_001204506 | 1 | 1 | 1 | 0 | 1 | 4 |
| hsa-miR-181a-5p | MIMAT0000256 | TMEM92 | NM_001168215 | 1 | 1 | 0 | 1 | 1 | 4 |
| hsa-miR-181a-5p | MIMAT0000256 | QSOX2 | NM_181701 | 1 | 1 | 0 | 1 | 1 | 4 |
| hsa-miR-181a-5p | MIMAT0000256 | TCEANC | XM_005274455 | 1 | 1 | 0 | 1 | 1 | 4 |
| hsa-miR-181a-5p | MIMAT0000256 | GIMAP1 | NM_130759 | 1 | 1 | 1 | 0 | 1 | 4 |
| hsa-miR-181a-5p | MIMAT0000256 | SYNPO2 | NM_133477 | 1 | 1 | 1 | 0 | 1 | 4 |
| hsa-miR-181a-5p | MIMAT0000256 | FAM9C | XM_005274463 | 1 | 1 | 0 | 1 | 1 | 4 |
| hsa-miR-181a-5p | MIMAT0000256 | HIGD2A | NM_138820 | 1 | 1 | 0 | 1 | 1 | 4 |
| hsa-miR-181a-5p | MIMAT0000256 | ACSF3 | NM_001243279 | 1 | 1 | 0 | 1 | 1 | 4 |
| hsa-miR-181a-5p | MIMAT0000256 | ZNF626 | NM_001076675 | 0 | 1 | 1 | 1 | 1 | 4 |
| hsa-miR-181a-5p | MIMAT0000256 | CCDC125 | XM_005276761 | 1 | 1 | 0 | 1 | 1 | 4 |
| hsa-miR-181a-5p | MIMAT0000256 | STK32A | XM_005268385 | 1 | 1 | 0 | 1 | 1 | 4 |
| hsa-miR-181a-5p | MIMAT0000256 | UNC5B | NM_170744 | 1 | 1 | 0 | 1 | 1 | 4 |
| hsa-miR-181a-5p | MIMAT0000256 | ARL5B | NM_178815 | 1 | 1 | 1 | 0 | 1 | 4 |
| hsa-miR-181a-5p | MIMAT0000256 | AK9 | NM_145025 | 1 | 1 | 1 | 0 | 1 | 4 |
| hsa-miR-181a-5p | MIMAT0000256 | FAM26D | NM_001256887 | 1 | 1 | 0 | 1 | 1 | 4 |
| hsa-miR-181a-5p | MIMAT0000256 | C6orf89 | NM_152734 | 1 | 1 | 1 | 0 | 1 | 4 |
| hsa-miR-181a-5p | MIMAT0000256 | ZSCAN23 | NM_001012455 | 1 | 1 | 1 | 0 | 1 | 4 |
| hsa-miR-181a-5p | MIMAT0000256 | ATP6V1C2 | NM_001039362 | 1 | 1 | 1 | 0 | 1 | 4 |
| hsa-miR-181a-5p | MIMAT0000256 | RICTOR | XM_005248277 | 1 | 1 | 0 | 1 | 1 | 4 |
| hsa-miR-181a-5p | MIMAT0000256 | BCL6B | NM_181844 | 1 | 1 | 0 | 1 | 1 | 4 |
| hsa-miR-181a-5p | MIMAT0000256 | C10orf67 | NM_153714 | 1 | 1 | 0 | 1 | 1 | 4 |
| hsa-miR-181a-5p | MIMAT0000256 | GPR137C | NM_001099652 | 1 | 1 | 1 | 0 | 1 | 4 |
| hsa-miR-181a-5p | MIMAT0000256 | ZNF615 | NM_001199324 | 1 | 1 | 1 | 0 | 1 | 4 |
| hsa-miR-181a-5p | MIMAT0000256 | RIMKLA | NM_173642 | 1 | 1 | 1 | 0 | 1 | 4 |
| hsa-miR-181a-5p | MIMAT0000256 | C9orf47 | NM_001001938 | 1 | 1 | 0 | 1 | 1 | 4 |
| hsa-miR-181a-5p | MIMAT0000256 | FFAR4 | NM_181745 | 0 | 1 | 1 | 1 | 1 | 4 |
| hsa-miR-181a-5p | MIMAT0000256 | CCDC144NL | NM_001004306 | 1 | 1 | 1 | 0 | 1 | 4 |
| hsa-miR-181a-5p | MIMAT0000256 | C17orf51 | NM_001113434 | 1 | 1 | 0 | 1 | 1 | 4 |
| hsa-miR-181a-5p | MIMAT0000256 | GADL1 | NM_207359 | 1 | 1 | 0 | 1 | 1 | 4 |
| hsa-miR-181a-5p | MIMAT0000256 | MYLK4 | NM_001012418 | 1 | 1 | 0 | 1 | 1 | 4 |
| hsa-miR-181a-5p | MIMAT0000256 | CD300E | NM_181449 | 1 | 1 | 0 | 1 | 1 | 4 |
| hsa-miR-181a-5p | MIMAT0000256 | MTX3 | NM_001010891 | 1 | 1 | 1 | 0 | 1 | 4 |
| hsa-miR-181a-5p | MIMAT0000256 | EYS | NM_001142801 | 1 | 1 | 1 | 0 | 1 | 4 |
| hsa-miR-181a-5p | MIMAT0000256 | NIPAL4 | NM_001099287 | 0 | 1 | 1 | 1 | 1 | 4 |
| hsa-miR-181a-5p | MIMAT0000256 | GJB7 | NM_198568 | 1 | 1 | 0 | 1 | 1 | 4 |
| hsa-miR-181a-5p | MIMAT0000256 | SOGA3 | NM_001012279 | 1 | 1 | 0 | 1 | 1 | 4 |
| hsa-miR-181a-5p | MIMAT0000256 | PLSCR5 | NM_001085420 | 1 | 1 | 1 | 0 | 1 | 4 |
| hsa-miR-181a-5p | MIMAT0000256 | C3orf80 | NM_001168214 | 1 | 1 | 1 | 0 | 1 | 4 |
| hsa-miR-181a-5p | MIMAT0000256 | C8orf59 | NM_001099670 | 1 | 1 | 1 | 0 | 1 | 4 |
| hsa-miR-181a-5p | MIMAT0000256 | GTF2H5 | NM_207118 | 1 | 1 | 1 | 0 | 1 | 4 |
| hsa-miR-181a-5p | MIMAT0000256 | ALG11 | NM_001004127 | 0 | 1 | 1 | 1 | 1 | 4 |
| hsa-miR-181a-5p | MIMAT0000256 | GOLGA8B | XM_005254389 | 1 | 1 | 0 | 1 | 1 | 4 |
| hsa-miR-181a-5p | MIMAT0000256 | GOLGA6L9 | XM_005254396 | 1 | 1 | 0 | 1 | 1 | 4 |
| hsa-miR-181a-5p | MIMAT0000256 | TMEM151B | NM_001137560 | 1 | 1 | 0 | 1 | 1 | 4 |
| hsa-miR-181a-5p | MIMAT0000256 | ANKRD20A3 | NM_001012419 | 1 | 1 | 0 | 1 | 1 | 4 |
| hsa-miR-181a-5p | MIMAT0000256 | GPX8 | NM_001008397 | 1 | 1 | 1 | 0 | 1 | 4 |
| hsa-miR-181a-5p | MIMAT0000256 | C15orf56 | NM_001039905 | 1 | 1 | 0 | 1 | 1 | 4 |
| hsa-miR-181a-5p | MIMAT0000256 | CCNI2 | NM_001039780 | 1 | 1 | 0 | 1 | 1 | 4 |
| hsa-miR-181a-5p | MIMAT0000256 | GOLGA6L10 | NM_001164465 | 1 | 1 | 1 | 0 | 1 | 4 |
| hsa-miR-181a-5p | MIMAT0000256 | PRR20B | NM_001130404 | 1 | 1 | 1 | 0 | 1 | 4 |
| hsa-miR-181a-5p | MIMAT0000256 | PRR20C | NM_001130405 | 1 | 1 | 1 | 0 | 1 | 4 |
| hsa-miR-181a-5p | MIMAT0000256 | PRR20D | NM_001130406 | 1 | 1 | 1 | 0 | 1 | 4 |
| hsa-miR-181a-5p | MIMAT0000256 | PRR20E | NM_001130407 | 1 | 1 | 1 | 0 | 1 | 4 |
| hsa-miR-181a-5p | MIMAT0000256 | LY75-CD302 | NM_001198759 | 1 | 1 | 1 | 0 | 1 | 4 |
| hsa-miR-181a-5p | MIMAT0000256 | LOC100653247 | XM_003403788 | 1 | 1 | 1 | 0 | 1 | 4 |
| hsa-miR-181a-5p | MIMAT0000256 | ABCA1 | NM_005502 | 1 | 0 | 0 | 1 | 1 | 3 |
| hsa-miR-181a-5p | MIMAT0000256 | ACTA2 | NM_001141945 | 1 | 1 | 0 | 0 | 1 | 3 |
| hsa-miR-181a-5p | MIMAT0000256 | ADM | NM_001124 | 0 | 1 | 1 | 0 | 1 | 3 |
| hsa-miR-181a-5p | MIMAT0000256 | ACAN | NM_013227 | 0 | 1 | 0 | 1 | 1 | 3 |
| hsa-miR-181a-5p | MIMAT0000256 | AGTR1 | NM_031850 | 0 | 1 | 1 | 0 | 1 | 3 |
| hsa-miR-181a-5p | MIMAT0000256 | AK2 | XM_005270603 | 1 | 1 | 0 | 0 | 1 | 3 |
| hsa-miR-181a-5p | MIMAT0000256 | ALDH3A2 | NM_001031806 | 1 | 0 | 0 | 1 | 1 | 3 |
| hsa-miR-181a-5p | MIMAT0000256 | ANGPT2 | NM_001147 | 1 | 1 | 0 | 0 | 1 | 3 |
| hsa-miR-181a-5p | MIMAT0000256 | SLC25A4 | NM_001151 | 0 | 1 | 1 | 0 | 1 | 3 |
| hsa-miR-181a-5p | MIMAT0000256 | ANXA11 | NM_001278408 | 0 | 1 | 0 | 1 | 1 | 3 |
| hsa-miR-181a-5p | MIMAT0000256 | AQP4 | XM_005258257 | 1 | 0 | 0 | 1 | 1 | 3 |
| hsa-miR-181a-5p | MIMAT0000256 | ASTN1 | NM_004319 | 1 | 1 | 0 | 0 | 1 | 3 |
| hsa-miR-181a-5p | MIMAT0000256 | ZFHX3 | NM_006885 | 1 | 1 | 0 | 0 | 1 | 3 |
| hsa-miR-181a-5p | MIMAT0000256 | BAAT | NM_001701 | 1 | 1 | 0 | 0 | 1 | 3 |
| hsa-miR-181a-5p | MIMAT0000256 | NKX3-2 | NM_001189 | 1 | 1 | 0 | 0 | 1 | 3 |
| hsa-miR-181a-5p | MIMAT0000256 | BCL2 | NM_000633 | 1 | 1 | 0 | 0 | 1 | 3 |
| hsa-miR-181a-5p | MIMAT0000256 | BCL6 | XM_005247694 | 1 | 0 | 0 | 1 | 1 | 3 |
| hsa-miR-181a-5p | MIMAT0000256 | PRDM1 | XM_005267094 | 1 | 1 | 0 | 0 | 1 | 3 |
| hsa-miR-181a-5p | MIMAT0000256 | BMP3 | NM_001201 | 1 | 1 | 0 | 0 | 1 | 3 |
| hsa-miR-181a-5p | MIMAT0000256 | CA8 | NM_004056 | 1 | 1 | 0 | 0 | 1 | 3 |
| hsa-miR-181a-5p | MIMAT0000256 | CACNA2D1 | XM_005250569 | 0 | 1 | 0 | 1 | 1 | 3 |
| hsa-miR-181a-5p | MIMAT0000256 | CACNB4 | NM_001005747 | 1 | 1 | 0 | 0 | 1 | 3 |
| hsa-miR-181a-5p | MIMAT0000256 | CALM1 | XM_005268091 | 0 | 1 | 0 | 1 | 1 | 3 |
| hsa-miR-181a-5p | MIMAT0000256 | CALU | NM_001199671 | 1 | 1 | 0 | 0 | 1 | 3 |
| hsa-miR-181a-5p | MIMAT0000256 | CAMK4 | XM_005272094 | 1 | 1 | 0 | 0 | 1 | 3 |
| hsa-miR-181a-5p | MIMAT0000256 | CAPZA2 | NM_006136 | 1 | 1 | 0 | 0 | 1 | 3 |
| hsa-miR-181a-5p | MIMAT0000256 | CBFA2T3 | NM_005187 | 0 | 1 | 0 | 1 | 1 | 3 |
| hsa-miR-181a-5p | MIMAT0000256 | CCNB1 | NM_031966 | 0 | 1 | 1 | 0 | 1 | 3 |
| hsa-miR-181a-5p | MIMAT0000256 | CCNG2 | NM_004354 | 0 | 1 | 0 | 1 | 1 | 3 |
| hsa-miR-181a-5p | MIMAT0000256 | ENTPD6 | XM_005260881 | 0 | 1 | 0 | 1 | 1 | 3 |
| hsa-miR-181a-5p | MIMAT0000256 | CD59 | NM_001127223 | 1 | 1 | 0 | 0 | 1 | 3 |
| hsa-miR-181a-5p | MIMAT0000256 | CD69 | NM_001781 | 0 | 1 | 1 | 0 | 1 | 3 |
| hsa-miR-181a-5p | MIMAT0000256 | CDK1 | XM_005270303 | 1 | 1 | 0 | 0 | 1 | 3 |
| hsa-miR-181a-5p | MIMAT0000256 | CDH8 | NM_001796 | 1 | 1 | 0 | 0 | 1 | 3 |
| hsa-miR-181a-5p | MIMAT0000256 | CDK6 | NM_001145306 | 1 | 0 | 0 | 1 | 1 | 3 |
| hsa-miR-181a-5p | MIMAT0000256 | CDS1 | NM_001263 | 0 | 1 | 0 | 1 | 1 | 3 |
| hsa-miR-181a-5p | MIMAT0000256 | CDX2 | NM_001265 | 1 | 1 | 0 | 0 | 1 | 3 |
| hsa-miR-181a-5p | MIMAT0000256 | CFL2 | NM_138638 | 1 | 1 | 0 | 0 | 1 | 3 |
| hsa-miR-181a-5p | MIMAT0000256 | CHRM3 | XM_005273031 | 1 | 1 | 0 | 0 | 1 | 3 |
| hsa-miR-181a-5p | MIMAT0000256 | COL6A3 | NM_004369 | 1 | 1 | 0 | 0 | 1 | 3 |
| hsa-miR-181a-5p | MIMAT0000256 | MAP3K8 | NM_005204 | 0 | 1 | 1 | 0 | 1 | 3 |
| hsa-miR-181a-5p | MIMAT0000256 | CPT1A | NM_001876 | 0 | 1 | 0 | 1 | 1 | 3 |
| hsa-miR-181a-5p | MIMAT0000256 | CRY2 | NM_021117 | 1 | 1 | 0 | 0 | 1 | 3 |
| hsa-miR-181a-5p | MIMAT0000256 | CSNK1A1 | XM_005268376 | 1 | 1 | 0 | 0 | 1 | 3 |
| hsa-miR-181a-5p | MIMAT0000256 | CTGF | NM_001901 | 1 | 0 | 0 | 1 | 1 | 3 |
| hsa-miR-181a-5p | MIMAT0000256 | CTNND1 | NM_001085458 | 0 | 1 | 0 | 1 | 1 | 3 |
| hsa-miR-181a-5p | MIMAT0000256 | CYLD | XM_005255811 | 1 | 0 | 0 | 1 | 1 | 3 |
| hsa-miR-181a-5p | MIMAT0000256 | CYP19A1 | NM_031226 | 0 | 1 | 0 | 1 | 1 | 3 |
| hsa-miR-181a-5p | MIMAT0000256 | DDX5 | NM_004396 | 1 | 1 | 0 | 0 | 1 | 3 |
| hsa-miR-181a-5p | MIMAT0000256 | DLG2 | NM_001142699 | 1 | 0 | 0 | 1 | 1 | 3 |
| hsa-miR-181a-5p | MIMAT0000256 | DPP6 | NM_001039350 | 0 | 1 | 0 | 1 | 1 | 3 |
| hsa-miR-181a-5p | MIMAT0000256 | DPYSL2 | NM_001197293 | 0 | 1 | 1 | 0 | 1 | 3 |
| hsa-miR-181a-5p | MIMAT0000256 | DR1 | NM_001938 | 1 | 1 | 0 | 0 | 1 | 3 |
| hsa-miR-181a-5p | MIMAT0000256 | LPAR1 | XM_005251781 | 1 | 0 | 0 | 1 | 1 | 3 |
| hsa-miR-181a-5p | MIMAT0000256 | EDN3 | NM_207032 | 1 | 0 | 0 | 1 | 1 | 3 |
| hsa-miR-181a-5p | MIMAT0000256 | EGR1 | NM_001964 | 1 | 0 | 0 | 1 | 1 | 3 |
| hsa-miR-181a-5p | MIMAT0000256 | EIF2S3 | NM_001415 | 1 | 1 | 0 | 0 | 1 | 3 |
| hsa-miR-181a-5p | MIMAT0000256 | ELK4 | NM_001973 | 1 | 1 | 0 | 0 | 1 | 3 |
| hsa-miR-181a-5p | MIMAT0000256 | EN1 | NM_001426 | 0 | 1 | 1 | 0 | 1 | 3 |
| hsa-miR-181a-5p | MIMAT0000256 | STOM | NM_004099 | 0 | 1 | 0 | 1 | 1 | 3 |
| hsa-miR-181a-5p | MIMAT0000256 | EPHA7 | NM_004440 | 1 | 0 | 0 | 1 | 1 | 3 |
| hsa-miR-181a-5p | MIMAT0000256 | EPHB1 | NM_004441 | 1 | 0 | 0 | 1 | 1 | 3 |
| hsa-miR-181a-5p | MIMAT0000256 | EPS15 | XM_005270617 | 0 | 1 | 0 | 1 | 1 | 3 |
| hsa-miR-181a-5p | MIMAT0000256 | ERBB4 | XM_005246375 | 0 | 1 | 0 | 1 | 1 | 3 |
| hsa-miR-181a-5p | MIMAT0000256 | ERG | NM_001243428 | 1 | 1 | 0 | 0 | 1 | 3 |
| hsa-miR-181a-5p | MIMAT0000256 | EVI2A | NM_001003927 | 0 | 1 | 1 | 0 | 1 | 3 |
| hsa-miR-181a-5p | MIMAT0000256 | ACSL4 | NM_022977 | 1 | 1 | 0 | 0 | 1 | 3 |
| hsa-miR-181a-5p | MIMAT0000256 | FDX1 | NM_004109 | 0 | 1 | 0 | 1 | 1 | 3 |
| hsa-miR-181a-5p | MIMAT0000256 | FGF7 | NM_002009 | 1 | 1 | 0 | 0 | 1 | 3 |
| hsa-miR-181a-5p | MIMAT0000256 | FGF12 | NM_021032 | 1 | 1 | 0 | 0 | 1 | 3 |
| hsa-miR-181a-5p | MIMAT0000256 | FLT1 | NM_002019 | 0 | 1 | 1 | 0 | 1 | 3 |
| hsa-miR-181a-5p | MIMAT0000256 | FOS | NM_005252 | 1 | 1 | 0 | 0 | 1 | 3 |
| hsa-miR-181a-5p | MIMAT0000256 | GABRA4 | NM_000809 | 0 | 1 | 1 | 0 | 1 | 3 |
| hsa-miR-181a-5p | MIMAT0000256 | GABRG1 | NM_173536 | 0 | 1 | 0 | 1 | 1 | 3 |
| hsa-miR-181a-5p | MIMAT0000256 | GALNT2 | NM_004481 | 1 | 0 | 0 | 1 | 1 | 3 |
| hsa-miR-181a-5p | MIMAT0000256 | GBAS | NM_001483 | 1 | 0 | 0 | 1 | 1 | 3 |
| hsa-miR-181a-5p | MIMAT0000256 | GCNT2 | NM_001491 | 1 | 1 | 0 | 0 | 1 | 3 |
| hsa-miR-181a-5p | MIMAT0000256 | GJA3 | NM_021954 | 1 | 0 | 0 | 1 | 1 | 3 |
| hsa-miR-181a-5p | MIMAT0000256 | GK | NM_001205019 | 1 | 1 | 0 | 0 | 1 | 3 |
| hsa-miR-181a-5p | MIMAT0000256 | GNAQ | NM_002072 | 1 | 1 | 0 | 0 | 1 | 3 |
| hsa-miR-181a-5p | MIMAT0000256 | GPD2 | NM_001083112 | 0 | 1 | 1 | 0 | 1 | 3 |
| hsa-miR-181a-5p | MIMAT0000256 | GRIA2 | NM_000826 | 1 | 1 | 0 | 0 | 1 | 3 |
| hsa-miR-181a-5p | MIMAT0000256 | GRIK3 | NM_000831 | 0 | 1 | 0 | 1 | 1 | 3 |
| hsa-miR-181a-5p | MIMAT0000256 | GRIK4 | NM_014619 | 1 | 1 | 0 | 0 | 1 | 3 |
| hsa-miR-181a-5p | MIMAT0000256 | ARHGAP35 | NM_004491 | 1 | 1 | 0 | 0 | 1 | 3 |
| hsa-miR-181a-5p | MIMAT0000256 | GRM1 | XM_005266950 | 0 | 1 | 0 | 1 | 1 | 3 |
| hsa-miR-181a-5p | MIMAT0000256 | CXCL2 | NM_002089 | 0 | 1 | 1 | 0 | 1 | 3 |
| hsa-miR-181a-5p | MIMAT0000256 | GUCY1A2 | NM_001256424 | 1 | 1 | 0 | 0 | 1 | 3 |
| hsa-miR-181a-5p | MIMAT0000256 | HTT | NM_002111 | 0 | 1 | 0 | 1 | 1 | 3 |
| hsa-miR-181a-5p | MIMAT0000256 | HMGCL | XM_005245857 | 1 | 1 | 0 | 0 | 1 | 3 |
| hsa-miR-181a-5p | MIMAT0000256 | HOXA11 | NM_005523 | 0 | 1 | 1 | 0 | 1 | 3 |
| hsa-miR-181a-5p | MIMAT0000256 | HOXB4 | NM_024015 | 0 | 1 | 1 | 0 | 1 | 3 |
| hsa-miR-181a-5p | MIMAT0000256 | HOXC8 | NM_022658 | 0 | 1 | 1 | 0 | 1 | 3 |
| hsa-miR-181a-5p | MIMAT0000256 | HOXD1 | NM_024501 | 0 | 1 | 1 | 0 | 1 | 3 |
| hsa-miR-181a-5p | MIMAT0000256 | AGFG1 | XM_005246516 | 1 | 1 | 0 | 0 | 1 | 3 |
| hsa-miR-181a-5p | MIMAT0000256 | AGFG2 | NM_006076 | 0 | 1 | 0 | 1 | 1 | 3 |
| hsa-miR-181a-5p | MIMAT0000256 | HSF2 | NM_004506 | 1 | 1 | 0 | 0 | 1 | 3 |
| hsa-miR-181a-5p | MIMAT0000256 | ID4 | XM_005249076 | 1 | 1 | 0 | 0 | 1 | 3 |
| hsa-miR-181a-5p | MIMAT0000256 | IDS | NM_000202 | 1 | 0 | 0 | 1 | 1 | 3 |
| hsa-miR-181a-5p | MIMAT0000256 | IFNA17 | NM_021268 | 1 | 1 | 0 | 0 | 1 | 3 |
| hsa-miR-181a-5p | MIMAT0000256 | IGFBP4 | NM_001552 | 0 | 1 | 0 | 1 | 1 | 3 |
| hsa-miR-181a-5p | MIMAT0000256 | RBPJ | NM_203284 | 1 | 0 | 0 | 1 | 1 | 3 |
| hsa-miR-181a-5p | MIMAT0000256 | IL1R1 | XM_005263933 | 1 | 1 | 0 | 0 | 1 | 3 |
| hsa-miR-181a-5p | MIMAT0000256 | ILF3 | XM_005259894 | 0 | 1 | 0 | 1 | 1 | 3 |
| hsa-miR-181a-5p | MIMAT0000256 | IMPG1 | NM_001563 | 0 | 1 | 0 | 1 | 1 | 3 |
| hsa-miR-181a-5p | MIMAT0000256 | INPP5A | XM_005252681 | 0 | 1 | 0 | 1 | 1 | 3 |
| hsa-miR-181a-5p | MIMAT0000256 | INSR | NM_000208 | 0 | 1 | 0 | 1 | 1 | 3 |
| hsa-miR-181a-5p | MIMAT0000256 | IRS1 | NM_005544 | 1 | 1 | 0 | 0 | 1 | 3 |
| hsa-miR-181a-5p | MIMAT0000256 | ITGA3 | NM_002204 | 0 | 1 | 0 | 1 | 1 | 3 |
| hsa-miR-181a-5p | MIMAT0000256 | JARID2 | NM_004973 | 0 | 1 | 0 | 1 | 1 | 3 |
| hsa-miR-181a-5p | MIMAT0000256 | KCNA3 | XM_005270844 | 1 | 1 | 0 | 0 | 1 | 3 |
| hsa-miR-181a-5p | MIMAT0000256 | KCNC2 | NM_001260497 | 1 | 1 | 0 | 0 | 1 | 3 |
| hsa-miR-181a-5p | MIMAT0000256 | KCNJ10 | NM_002241 | 1 | 1 | 0 | 0 | 1 | 3 |
| hsa-miR-181a-5p | MIMAT0000256 | KIF5C | NM_004522 | 1 | 0 | 0 | 1 | 1 | 3 |
| hsa-miR-181a-5p | MIMAT0000256 | KPNA1 | XM_005247439 | 0 | 1 | 0 | 1 | 1 | 3 |
| hsa-miR-181a-5p | MIMAT0000256 | LAMC1 | NM_002293 | 1 | 1 | 0 | 0 | 1 | 3 |
| hsa-miR-181a-5p | MIMAT0000256 | LBR | NM_002296 | 0 | 1 | 1 | 0 | 1 | 3 |
| hsa-miR-181a-5p | MIMAT0000256 | LIF | NM_002309 | 0 | 1 | 0 | 1 | 1 | 3 |
| hsa-miR-181a-5p | MIMAT0000256 | LMO1 | NM_002315 | 1 | 1 | 0 | 0 | 1 | 3 |
| hsa-miR-181a-5p | MIMAT0000256 | MARCKS | NM_002356 | 1 | 0 | 0 | 1 | 1 | 3 |
| hsa-miR-181a-5p | MIMAT0000256 | MAOA | NM_001270458 | 1 | 1 | 0 | 0 | 1 | 3 |
| hsa-miR-181a-5p | MIMAT0000256 | MAP1A | XM_005254385 | 0 | 1 | 0 | 1 | 1 | 3 |
| hsa-miR-181a-5p | MIMAT0000256 | MARK1 | NM_018650 | 0 | 1 | 1 | 0 | 1 | 3 |
| hsa-miR-181a-5p | MIMAT0000256 | MCC | NM_001085377 | 0 | 1 | 1 | 0 | 1 | 3 |
| hsa-miR-181a-5p | MIMAT0000256 | MCM6 | NM_005915 | 0 | 1 | 0 | 1 | 1 | 3 |
| hsa-miR-181a-5p | MIMAT0000256 | ME2 | NM_002396 | 1 | 1 | 0 | 0 | 1 | 3 |
| hsa-miR-181a-5p | MIMAT0000256 | MEF2A | NM_001171894 | 1 | 1 | 0 | 0 | 1 | 3 |
| hsa-miR-181a-5p | MIMAT0000256 | MGAT3 | NM_002409 | 1 | 1 | 0 | 0 | 1 | 3 |
| hsa-miR-181a-5p | MIMAT0000256 | MITF | NM_198159 | 1 | 0 | 0 | 1 | 1 | 3 |
| hsa-miR-181a-5p | MIMAT0000256 | MAP3K9 | NM_033141 | 1 | 1 | 0 | 0 | 1 | 3 |
| hsa-miR-181a-5p | MIMAT0000256 | AFF1 | XM_005263009 | 1 | 1 | 0 | 0 | 1 | 3 |
| hsa-miR-181a-5p | MIMAT0000256 | MLLT3 | XM_005251460 | 1 | 1 | 0 | 0 | 1 | 3 |
| hsa-miR-181a-5p | MIMAT0000256 | MMP7 | NM_002423 | 1 | 1 | 0 | 0 | 1 | 3 |
| hsa-miR-181a-5p | MIMAT0000256 | MMP10 | NM_002425 | 1 | 0 | 0 | 1 | 1 | 3 |
| hsa-miR-181a-5p | MIMAT0000256 | MSI1 | NM_002442 | 1 | 1 | 0 | 0 | 1 | 3 |
| hsa-miR-181a-5p | MIMAT0000256 | MSR1 | NM_138715 | 1 | 1 | 0 | 0 | 1 | 3 |
| hsa-miR-181a-5p | MIMAT0000256 | MYO9B | NM_001130065 | 1 | 1 | 0 | 0 | 1 | 3 |
| hsa-miR-181a-5p | MIMAT0000256 | 2-Sep | NM_001008491 | 1 | 1 | 0 | 0 | 1 | 3 |
| hsa-miR-181a-5p | MIMAT0000256 | NEFH | NM_021076 | 1 | 1 | 0 | 0 | 1 | 3 |
| hsa-miR-181a-5p | MIMAT0000256 | NPAT | NM_002519 | 1 | 1 | 0 | 0 | 1 | 3 |
| hsa-miR-181a-5p | MIMAT0000256 | NTRK2 | XM_005252001 | 1 | 0 | 0 | 1 | 1 | 3 |
| hsa-miR-181a-5p | MIMAT0000256 | OGN | NM_033014 | 1 | 1 | 0 | 0 | 1 | 3 |
| hsa-miR-181a-5p | MIMAT0000256 | P2RY2 | NM_176072 | 1 | 1 | 0 | 0 | 1 | 3 |
| hsa-miR-181a-5p | MIMAT0000256 | PARN | NM_002582 | 1 | 1 | 0 | 0 | 1 | 3 |
| hsa-miR-181a-5p | MIMAT0000256 | PAX5 | NM_016734 | 1 | 1 | 0 | 0 | 1 | 3 |
| hsa-miR-181a-5p | MIMAT0000256 | PCCB | XM_005247510 | 1 | 0 | 0 | 1 | 1 | 3 |
| hsa-miR-181a-5p | MIMAT0000256 | PDE3B | NM_000922 | 1 | 1 | 0 | 0 | 1 | 3 |
| hsa-miR-181a-5p | MIMAT0000256 | PDE7A | NM_001242318 | 1 | 1 | 0 | 0 | 1 | 3 |
| hsa-miR-181a-5p | MIMAT0000256 | PFKFB2 | NM_006212 | 1 | 0 | 0 | 1 | 1 | 3 |
| hsa-miR-181a-5p | MIMAT0000256 | PIGA | NM_002641 | 1 | 0 | 0 | 1 | 1 | 3 |
| hsa-miR-181a-5p | MIMAT0000256 | PIK3CB | XM_005247530 | 1 | 1 | 0 | 0 | 1 | 3 |
| hsa-miR-181a-5p | MIMAT0000256 | PLRG1 | NM_002669 | 1 | 1 | 0 | 0 | 1 | 3 |
| hsa-miR-181a-5p | MIMAT0000256 | PODXL | NM_001018111 | 1 | 1 | 0 | 0 | 1 | 3 |
| hsa-miR-181a-5p | MIMAT0000256 | POU3F2 | NM_005604 | 1 | 1 | 0 | 0 | 1 | 3 |
| hsa-miR-181a-5p | MIMAT0000256 | PPM1A | XM_005267777 | 1 | 0 | 0 | 1 | 1 | 3 |
| hsa-miR-181a-5p | MIMAT0000256 | PPM1B | NM_177968 | 1 | 1 | 0 | 0 | 1 | 3 |
| hsa-miR-181a-5p | MIMAT0000256 | PPP2R2C | XM_005247976 | 1 | 1 | 0 | 0 | 1 | 3 |
| hsa-miR-181a-5p | MIMAT0000256 | PPP2R3A | NM_002718 | 1 | 1 | 0 | 0 | 1 | 3 |
| hsa-miR-181a-5p | MIMAT0000256 | PRKAR2A | XM_005265314 | 1 | 1 | 0 | 0 | 1 | 3 |
| hsa-miR-181a-5p | MIMAT0000256 | PRKCI | NM_002740 | 1 | 0 | 0 | 1 | 1 | 3 |
| hsa-miR-181a-5p | MIMAT0000256 | MAPK10 | XM_005263133 | 1 | 0 | 0 | 1 | 1 | 3 |
| hsa-miR-181a-5p | MIMAT0000256 | PSMB2 | NM_002794 | 1 | 1 | 0 | 0 | 1 | 3 |
| hsa-miR-181a-5p | MIMAT0000256 | PTAFR | NM_001164723 | 1 | 1 | 0 | 0 | 1 | 3 |
| hsa-miR-181a-5p | MIMAT0000256 | PTEN | NM_000314 | 1 | 1 | 0 | 0 | 1 | 3 |
| hsa-miR-181a-5p | MIMAT0000256 | TWF1 | NM_001242397 | 1 | 1 | 0 | 0 | 1 | 3 |
| hsa-miR-181a-5p | MIMAT0000256 | PTPN14 | NM_005401 | 1 | 1 | 0 | 0 | 1 | 3 |
| hsa-miR-181a-5p | MIMAT0000256 | QDPR | NM_000320 | 1 | 1 | 0 | 0 | 1 | 3 |
| hsa-miR-181a-5p | MIMAT0000256 | RAB3B | NM_002867 | 1 | 0 | 0 | 1 | 1 | 3 |
| hsa-miR-181a-5p | MIMAT0000256 | RAN | XM_005253592 | 1 | 1 | 0 | 0 | 1 | 3 |
| hsa-miR-181a-5p | MIMAT0000256 | RCN1 | NM_002901 | 1 | 1 | 0 | 0 | 1 | 3 |
| hsa-miR-181a-5p | MIMAT0000256 | RFX3 | NM_134428 | 1 | 0 | 0 | 1 | 1 | 3 |
| hsa-miR-181a-5p | MIMAT0000256 | RP2 | NM_006915 | 1 | 1 | 0 | 0 | 1 | 3 |
| hsa-miR-181a-5p | MIMAT0000256 | RPN2 | XM_005260491 | 1 | 1 | 0 | 0 | 1 | 3 |
| hsa-miR-181a-5p | MIMAT0000256 | SCML1 | NM_001037540 | 1 | 0 | 0 | 1 | 1 | 3 |
| hsa-miR-181a-5p | MIMAT0000256 | CCL8 | NM_005623 | 1 | 1 | 0 | 0 | 1 | 3 |
| hsa-miR-181a-5p | MIMAT0000256 | CCL13 | NM_005408 | 1 | 1 | 0 | 0 | 1 | 3 |
| hsa-miR-181a-5p | MIMAT0000256 | SDC2 | NM_002998 | 1 | 1 | 0 | 0 | 1 | 3 |
| hsa-miR-181a-5p | MIMAT0000256 | SFRP4 | NM_003014 | 1 | 1 | 0 | 0 | 1 | 3 |
| hsa-miR-181a-5p | MIMAT0000256 | SRSF5 | XM_005268001 | 1 | 1 | 0 | 0 | 1 | 3 |
| hsa-miR-181a-5p | MIMAT0000256 | SGCD | NM_000337 | 1 | 0 | 0 | 1 | 1 | 3 |
| hsa-miR-181a-5p | MIMAT0000256 | SLC2A3 | NM_006931 | 1 | 1 | 0 | 0 | 1 | 3 |
| hsa-miR-181a-5p | MIMAT0000256 | SLC22A2 | NM_003058 | 1 | 1 | 0 | 0 | 1 | 3 |
| hsa-miR-181a-5p | MIMAT0000256 | SMARCC2 | NM_003075 | 1 | 1 | 0 | 0 | 1 | 3 |
| hsa-miR-181a-5p | MIMAT0000256 | SORL1 | NM_003105 | 1 | 0 | 0 | 1 | 1 | 3 |
| hsa-miR-181a-5p | MIMAT0000256 | SRI | NM_003130 | 1 | 1 | 0 | 0 | 1 | 3 |
| hsa-miR-181a-5p | MIMAT0000256 | SSR3 | NM_007107 | 1 | 0 | 0 | 1 | 1 | 3 |
| hsa-miR-181a-5p | MIMAT0000256 | VAMP1 | NM_199245 | 1 | 1 | 0 | 0 | 1 | 3 |
| hsa-miR-181a-5p | MIMAT0000256 | SYT4 | NM_020783 | 1 | 0 | 0 | 1 | 1 | 3 |
| hsa-miR-181a-5p | MIMAT0000256 | TAF4 | NM_003185 | 1 | 1 | 0 | 0 | 1 | 3 |
| hsa-miR-181a-5p | MIMAT0000256 | MAP3K7 | NM_145331 | 1 | 1 | 0 | 0 | 1 | 3 |
| hsa-miR-181a-5p | MIMAT0000256 | TGFBR2 | NM_001024847 | 1 | 1 | 0 | 0 | 1 | 3 |
| hsa-miR-181a-5p | MIMAT0000256 | THBS4 | NM_003248 | 1 | 1 | 0 | 0 | 1 | 3 |
| hsa-miR-181a-5p | MIMAT0000256 | TRPC1 | NM_001251845 | 1 | 1 | 0 | 0 | 1 | 3 |
| hsa-miR-181a-5p | MIMAT0000256 | DNAJC7 | NM_003315 | 1 | 1 | 0 | 0 | 1 | 3 |
| hsa-miR-181a-5p | MIMAT0000256 | UBE2D3 | NM_181892 | 1 | 1 | 0 | 0 | 1 | 3 |
| hsa-miR-181a-5p | MIMAT0000256 | UBTF | NM_014233 | 1 | 0 | 0 | 1 | 1 | 3 |
| hsa-miR-181a-5p | MIMAT0000256 | VCAM1 | NM_001078 | 1 | 1 | 0 | 0 | 1 | 3 |
| hsa-miR-181a-5p | MIMAT0000256 | YWHAB | NM_003404 | 1 | 1 | 0 | 0 | 1 | 3 |
| hsa-miR-181a-5p | MIMAT0000256 | SF1 | XM_005274236 | 1 | 1 | 0 | 0 | 1 | 3 |
| hsa-miR-181a-5p | MIMAT0000256 | ZKSCAN1 | XM_005250564 | 1 | 1 | 0 | 0 | 1 | 3 |
| hsa-miR-181a-5p | MIMAT0000256 | ZNF136 | NM_003437 | 0 | 1 | 1 | 0 | 1 | 3 |
| hsa-miR-181a-5p | MIMAT0000256 | ZNF140 | NM_003440 | 0 | 1 | 1 | 0 | 1 | 3 |
| hsa-miR-181a-5p | MIMAT0000256 | ZNF175 | NM_007147 | 1 | 1 | 0 | 0 | 1 | 3 |
| hsa-miR-181a-5p | MIMAT0000256 | ZFAND5 | NM_001102420 | 1 | 0 | 0 | 1 | 1 | 3 |
| hsa-miR-181a-5p | MIMAT0000256 | ZNF236 | NM_007345 | 1 | 1 | 0 | 0 | 1 | 3 |
| hsa-miR-181a-5p | MIMAT0000256 | LUZP1 | XM_005245991 | 1 | 0 | 0 | 1 | 1 | 3 |
| hsa-miR-181a-5p | MIMAT0000256 | LRP8 | NM_004631 | 1 | 1 | 0 | 0 | 1 | 3 |
| hsa-miR-181a-5p | MIMAT0000256 | SHOC2 | NM_007373 | 0 | 1 | 0 | 1 | 1 | 3 |
| hsa-miR-181a-5p | MIMAT0000256 | CSRP3 | NM_003476 | 1 | 1 | 0 | 0 | 1 | 3 |
| hsa-miR-181a-5p | MIMAT0000256 | ANP32A | NM_006305 | 1 | 1 | 0 | 0 | 1 | 3 |
| hsa-miR-181a-5p | MIMAT0000256 | NCOA3 | NM_181659 | 0 | 1 | 0 | 1 | 1 | 3 |
| hsa-miR-181a-5p | MIMAT0000256 | USP9X | NM_001039590 | 1 | 1 | 0 | 0 | 1 | 3 |
| hsa-miR-181a-5p | MIMAT0000256 | TRRAP | NM_001244580 | 0 | 1 | 0 | 1 | 1 | 3 |
| hsa-miR-181a-5p | MIMAT0000256 | PIP4K2B | NM_003559 | 1 | 1 | 0 | 0 | 1 | 3 |
| hsa-miR-181a-5p | MIMAT0000256 | DYRK2 | NM_006482 | 1 | 1 | 0 | 0 | 1 | 3 |
| hsa-miR-181a-5p | MIMAT0000256 | SORBS2 | XM_005263296 | 1 | 1 | 0 | 0 | 1 | 3 |
| hsa-miR-181a-5p | MIMAT0000256 | CDC42BPA | XM_005273321 | 0 | 1 | 0 | 1 | 1 | 3 |
| hsa-miR-181a-5p | MIMAT0000256 | PPFIA1 | NM_003626 | 0 | 1 | 1 | 0 | 1 | 3 |
| hsa-miR-181a-5p | MIMAT0000256 | DGKE | NM_003647 | 1 | 0 | 0 | 1 | 1 | 3 |
| hsa-miR-181a-5p | MIMAT0000256 | API5 | NM_001142930 | 1 | 1 | 0 | 0 | 1 | 3 |
| hsa-miR-181a-5p | MIMAT0000256 | CGGBP1 | NM_001008390 | 1 | 1 | 0 | 0 | 1 | 3 |
| hsa-miR-181a-5p | MIMAT0000256 | CDC14A | NM_003672 | 1 | 1 | 0 | 0 | 1 | 3 |
| hsa-miR-181a-5p | MIMAT0000256 | PLA2G4C | NM_001159323 | 0 | 1 | 1 | 0 | 1 | 3 |
| hsa-miR-181a-5p | MIMAT0000256 | VAMP4 | NM_003762 | 1 | 1 | 0 | 0 | 1 | 3 |
| hsa-miR-181a-5p | MIMAT0000256 | B3GALT1 | NM_020981 | 1 | 1 | 0 | 0 | 1 | 3 |
| hsa-miR-181a-5p | MIMAT0000256 | NOL4 | NM_003787 | 1 | 1 | 0 | 0 | 1 | 3 |
| hsa-miR-181a-5p | MIMAT0000256 | EED | NM_152991 | 0 | 1 | 1 | 0 | 1 | 3 |
| hsa-miR-181a-5p | MIMAT0000256 | DCAF5 | NM_003861 | 0 | 1 | 0 | 1 | 1 | 3 |
| hsa-miR-181a-5p | MIMAT0000256 | INPP4B | NM_003866 | 0 | 1 | 0 | 1 | 1 | 3 |
| hsa-miR-181a-5p | MIMAT0000256 | NRP1 | NM_003873 | 1 | 0 | 0 | 1 | 1 | 3 |
| hsa-miR-181a-5p | MIMAT0000256 | VNN2 | NM_004665 | 0 | 1 | 0 | 1 | 1 | 3 |
| hsa-miR-181a-5p | MIMAT0000256 | TIMELESS | NM_003920 | 0 | 1 | 1 | 0 | 1 | 3 |
| hsa-miR-181a-5p | MIMAT0000256 | WASF1 | NM_003931 | 1 | 1 | 0 | 0 | 1 | 3 |
| hsa-miR-181a-5p | MIMAT0000256 | USP13 | NM_003940 | 0 | 1 | 0 | 1 | 1 | 3 |
| hsa-miR-181a-5p | MIMAT0000256 | TBX18 | NM_001080508 | 1 | 1 | 0 | 0 | 1 | 3 |
| hsa-miR-181a-5p | MIMAT0000256 | USP8 | NM_001128610 | 1 | 0 | 0 | 1 | 1 | 3 |
| hsa-miR-181a-5p | MIMAT0000256 | DGKI | NM_004717 | 1 | 1 | 0 | 0 | 1 | 3 |
| hsa-miR-181a-5p | MIMAT0000256 | BUB3 | NM_004725 | 1 | 1 | 0 | 0 | 1 | 3 |
| hsa-miR-181a-5p | MIMAT0000256 | NRXN3 | XM_005268218 | 1 | 1 | 0 | 0 | 1 | 3 |
| hsa-miR-181a-5p | MIMAT0000256 | HS6ST1 | NM_004807 | 1 | 1 | 0 | 0 | 1 | 3 |
| hsa-miR-181a-5p | MIMAT0000256 | ZRANB2 | NM_005455 | 1 | 1 | 0 | 0 | 1 | 3 |
| hsa-miR-181a-5p | MIMAT0000256 | TMPRSS11D | NM_004262 | 1 | 1 | 0 | 0 | 1 | 3 |
| hsa-miR-181a-5p | MIMAT0000256 | ZNF264 | NM_003417 | 1 | 1 | 0 | 0 | 1 | 3 |
| hsa-miR-181a-5p | MIMAT0000256 | ROCK2 | XM_005246190 | 1 | 1 | 0 | 0 | 1 | 3 |
| hsa-miR-181a-5p | MIMAT0000256 | ADAMTS1 | NM_006988 | 1 | 1 | 0 | 0 | 1 | 3 |
| hsa-miR-181a-5p | MIMAT0000256 | IGDCC3 | NM_004884 | 0 | 1 | 0 | 1 | 1 | 3 |
| hsa-miR-181a-5p | MIMAT0000256 | WSCD2 | XM_005269240 | 0 | 1 | 0 | 1 | 1 | 3 |
| hsa-miR-181a-5p | MIMAT0000256 | UBE3C | NM_014671 | 1 | 1 | 0 | 0 | 1 | 3 |
| hsa-miR-181a-5p | MIMAT0000256 | KIAA0226 | NM_001145642 | 0 | 1 | 0 | 1 | 1 | 3 |
| hsa-miR-181a-5p | MIMAT0000256 | SEC14L5 | NM_014692 | 0 | 1 | 0 | 1 | 1 | 3 |
| hsa-miR-181a-5p | MIMAT0000256 | KIAA0408 | NM_014702 | 1 | 1 | 0 | 0 | 1 | 3 |
| hsa-miR-181a-5p | MIMAT0000256 | HDAC9 | XM_005249913 | 1 | 0 | 0 | 1 | 1 | 3 |
| hsa-miR-181a-5p | MIMAT0000256 | CCP110 | XM_005255720 | 0 | 1 | 0 | 1 | 1 | 3 |
| hsa-miR-181a-5p | MIMAT0000256 | SLK | XM_005270301 | 1 | 1 | 0 | 0 | 1 | 3 |
| hsa-miR-181a-5p | MIMAT0000256 | DAZAP2 | NM_014764 | 0 | 1 | 1 | 0 | 1 | 3 |
| hsa-miR-181a-5p | MIMAT0000256 | CTIF | NM_001142397 | 0 | 1 | 0 | 1 | 1 | 3 |
| hsa-miR-181a-5p | MIMAT0000256 | TSC22D2 | XM_005247920 | 0 | 1 | 0 | 1 | 1 | 3 |
| hsa-miR-181a-5p | MIMAT0000256 | MELK | XM_005251633 | 0 | 1 | 0 | 1 | 1 | 3 |
| hsa-miR-181a-5p | MIMAT0000256 | ZEB2 | NM_014795 | 0 | 1 | 1 | 0 | 1 | 3 |
| hsa-miR-181a-5p | MIMAT0000256 | ZBTB24 | NM_014797 | 1 | 0 | 0 | 1 | 1 | 3 |
| hsa-miR-181a-5p | MIMAT0000256 | CEP350 | XM_005245635 | 0 | 1 | 0 | 1 | 1 | 3 |
| hsa-miR-181a-5p | MIMAT0000256 | TRANK1 | NM_014831 | 1 | 0 | 0 | 1 | 1 | 3 |
| hsa-miR-181a-5p | MIMAT0000256 | SMG7 | XM_005245647 | 1 | 0 | 0 | 1 | 1 | 3 |
| hsa-miR-181a-5p | MIMAT0000256 | KIAA0196 | NM_014846 | 0 | 1 | 1 | 0 | 1 | 3 |
| hsa-miR-181a-5p | MIMAT0000256 | G3BP2 | XM_005263384 | 0 | 1 | 0 | 1 | 1 | 3 |
| hsa-miR-181a-5p | MIMAT0000256 | HS3ST3A1 | NM_006042 | 1 | 1 | 0 | 0 | 1 | 3 |
| hsa-miR-181a-5p | MIMAT0000256 | ABI1 | NM_005470 | 0 | 1 | 1 | 0 | 1 | 3 |
| hsa-miR-181a-5p | MIMAT0000256 | PDCD6IP | NM_001162429 | 0 | 1 | 1 | 0 | 1 | 3 |
| hsa-miR-181a-5p | MIMAT0000256 | BCL2L11 | NM_001204108 | 0 | 1 | 0 | 1 | 1 | 3 |
| hsa-miR-181a-5p | MIMAT0000256 | HMGXB4 | NM_001003681 | 1 | 1 | 0 | 0 | 1 | 3 |
| hsa-miR-181a-5p | MIMAT0000256 | CTDSP2 | NM_005730 | 0 | 1 | 0 | 1 | 1 | 3 |
| hsa-miR-181a-5p | MIMAT0000256 | CLEC3A | NM_005752 | 0 | 1 | 1 | 0 | 1 | 3 |
| hsa-miR-181a-5p | MIMAT0000256 | MBNL2 | XM_005254018 | 1 | 1 | 0 | 0 | 1 | 3 |
| hsa-miR-181a-5p | MIMAT0000256 | ABI2 | XM_005246217 | 0 | 1 | 0 | 1 | 1 | 3 |
| hsa-miR-181a-5p | MIMAT0000256 | PLXNC1 | NM_005761 | 1 | 1 | 0 | 0 | 1 | 3 |
| hsa-miR-181a-5p | MIMAT0000256 | ZNF197 | XM_005264783 | 1 | 0 | 0 | 1 | 1 | 3 |
| hsa-miR-181a-5p | MIMAT0000256 | ZNF443 | XM_005259705 | 1 | 1 | 0 | 0 | 1 | 3 |
| hsa-miR-181a-5p | MIMAT0000256 | STAG1 | XM_005247064 | 0 | 1 | 0 | 1 | 1 | 3 |
| hsa-miR-181a-5p | MIMAT0000256 | DNAJA2 | NM_005880 | 1 | 0 | 0 | 1 | 1 | 3 |
| hsa-miR-181a-5p | MIMAT0000256 | 6-Mar | NM_005885 | 1 | 1 | 0 | 0 | 1 | 3 |
| hsa-miR-181a-5p | MIMAT0000256 | PIAS3 | XM_005277332 | 1 | 1 | 0 | 0 | 1 | 3 |
| hsa-miR-181a-5p | MIMAT0000256 | YAP1 | XM_005271378 | 1 | 0 | 0 | 1 | 1 | 3 |
| hsa-miR-181a-5p | MIMAT0000256 | PGRMC2 | NM_006320 | 0 | 1 | 0 | 1 | 1 | 3 |
| hsa-miR-181a-5p | MIMAT0000256 | COG5 | NM_001161520 | 1 | 1 | 0 | 0 | 1 | 3 |
| hsa-miR-181a-5p | MIMAT0000256 | CARM1 | NM_199141 | 0 | 1 | 0 | 1 | 1 | 3 |
| hsa-miR-181a-5p | MIMAT0000256 | SEMA4F | NM_004263 | 0 | 1 | 0 | 1 | 1 | 3 |
| hsa-miR-181a-5p | MIMAT0000256 | IPO8 | NM_006390 | 0 | 1 | 1 | 0 | 1 | 3 |
| hsa-miR-181a-5p | MIMAT0000256 | IPO7 | NM_006391 | 0 | 1 | 1 | 0 | 1 | 3 |
| hsa-miR-181a-5p | MIMAT0000256 | ARL6IP5 | NM_006407 | 0 | 1 | 1 | 0 | 1 | 3 |
| hsa-miR-181a-5p | MIMAT0000256 | SPTLC1 | NM_006415 | 1 | 1 | 0 | 0 | 1 | 3 |
| hsa-miR-181a-5p | MIMAT0000256 | MAB21L2 | NM_006439 | 1 | 1 | 0 | 0 | 1 | 3 |
| hsa-miR-181a-5p | MIMAT0000256 | TRIM3 | XM_005252751 | 0 | 1 | 0 | 1 | 1 | 3 |
| hsa-miR-181a-5p | MIMAT0000256 | IGF2BP2 | NM_006548 | 0 | 1 | 1 | 0 | 1 | 3 |
| hsa-miR-181a-5p | MIMAT0000256 | MTX2 | NM_006554 | 1 | 1 | 0 | 0 | 1 | 3 |
| hsa-miR-181a-5p | MIMAT0000256 | PNMA2 | XM_005273377 | 0 | 1 | 0 | 1 | 1 | 3 |
| hsa-miR-181a-5p | MIMAT0000256 | POLQ | NM_199420 | 0 | 1 | 1 | 0 | 1 | 3 |
| hsa-miR-181a-5p | MIMAT0000256 | SIX2 | XM_005264100 | 1 | 1 | 0 | 0 | 1 | 3 |
| hsa-miR-181a-5p | MIMAT0000256 | ZNF268 | NM_152943 | 0 | 1 | 1 | 0 | 1 | 3 |
| hsa-miR-181a-5p | MIMAT0000256 | CYSLTR1 | NM_006639 | 1 | 1 | 0 | 0 | 1 | 3 |
| hsa-miR-181a-5p | MIMAT0000256 | FRS2 | NM_001278351 | 0 | 1 | 0 | 1 | 1 | 3 |
| hsa-miR-181a-5p | MIMAT0000256 | PPP1R17 | NM_006658 | 0 | 1 | 0 | 1 | 1 | 3 |
| hsa-miR-181a-5p | MIMAT0000256 | PDE10A | NM_001130690 | 1 | 1 | 0 | 0 | 1 | 3 |
| hsa-miR-181a-5p | MIMAT0000256 | ADAM28 | NM_014265 | 0 | 1 | 1 | 0 | 1 | 3 |
| hsa-miR-181a-5p | MIMAT0000256 | EDAR | NM_022336 | 1 | 0 | 0 | 1 | 1 | 3 |
| hsa-miR-181a-5p | MIMAT0000256 | AFG3L2 | NM_006796 | 0 | 1 | 1 | 0 | 1 | 3 |
| hsa-miR-181a-5p | MIMAT0000256 | AP3M2 | NM_001134296 | 0 | 1 | 0 | 1 | 1 | 3 |
| hsa-miR-181a-5p | MIMAT0000256 | PNRC1 | NM_006813 | 0 | 1 | 0 | 1 | 1 | 3 |
| hsa-miR-181a-5p | MIMAT0000256 | CKAP4 | NM_006825 | 0 | 1 | 0 | 1 | 1 | 3 |
| hsa-miR-181a-5p | MIMAT0000256 | TMED10 | NM_006827 | 1 | 0 | 0 | 1 | 1 | 3 |
| hsa-miR-181a-5p | MIMAT0000256 | KIF2C | XM_005270394 | 1 | 1 | 0 | 0 | 1 | 3 |
| hsa-miR-181a-5p | MIMAT0000256 | TLK2 | XM_005256970 | 1 | 1 | 0 | 0 | 1 | 3 |
| hsa-miR-181a-5p | MIMAT0000256 | KDELR2 | NM_006854 | 1 | 1 | 0 | 0 | 1 | 3 |
| hsa-miR-181a-5p | MIMAT0000256 | KDELR3 | NM_006855 | 1 | 1 | 0 | 0 | 1 | 3 |
| hsa-miR-181a-5p | MIMAT0000256 | VAX1 | NM_199131 | 1 | 1 | 0 | 0 | 1 | 3 |
| hsa-miR-181a-5p | MIMAT0000256 | PAPD7 | XM_005248234 | 0 | 1 | 0 | 1 | 1 | 3 |
| hsa-miR-181a-5p | MIMAT0000256 | ESM1 | NM_007036 | 0 | 1 | 1 | 0 | 1 | 3 |
| hsa-miR-181a-5p | MIMAT0000256 | ATE1 | XM_005269456 | 0 | 1 | 0 | 1 | 1 | 3 |
| hsa-miR-181a-5p | MIMAT0000256 | BVES | NM_001199563 | 1 | 0 | 0 | 1 | 1 | 3 |
| hsa-miR-181a-5p | MIMAT0000256 | FAM107A | NM_007177 | 0 | 1 | 0 | 1 | 1 | 3 |
| hsa-miR-181a-5p | MIMAT0000256 | BAZ2A | NM_013449 | 1 | 1 | 0 | 0 | 1 | 3 |
| hsa-miR-181a-5p | MIMAT0000256 | DDX20 | NM_007204 | 0 | 1 | 1 | 0 | 1 | 3 |
| hsa-miR-181a-5p | MIMAT0000256 | KLHL2 | NM_007246 | 0 | 1 | 1 | 0 | 1 | 3 |
| hsa-miR-181a-5p | MIMAT0000256 | KLF12 | XM_005266251 | 1 | 1 | 0 | 0 | 1 | 3 |
| hsa-miR-181a-5p | MIMAT0000256 | RNF13 | XM_005247090 | 1 | 1 | 0 | 0 | 1 | 3 |
| hsa-miR-181a-5p | MIMAT0000256 | NLGN4Y | NM_014893 | 1 | 0 | 0 | 1 | 1 | 3 |
| hsa-miR-181a-5p | MIMAT0000256 | SLITRK3 | XM_005247230 | 1 | 1 | 0 | 0 | 1 | 3 |
| hsa-miR-181a-5p | MIMAT0000256 | CNKSR2 | NM_014927 | 0 | 1 | 0 | 1 | 1 | 3 |
| hsa-miR-181a-5p | MIMAT0000256 | MON1B | NM_014940 | 1 | 1 | 0 | 0 | 1 | 3 |
| hsa-miR-181a-5p | MIMAT0000256 | WDR37 | XM_005252417 | 0 | 1 | 0 | 1 | 1 | 3 |
| hsa-miR-181a-5p | MIMAT0000256 | EPN2 | XM_005256538 | 0 | 1 | 0 | 1 | 1 | 3 |
| hsa-miR-181a-5p | MIMAT0000256 | RAB18 | NM_001256410 | 0 | 1 | 0 | 1 | 1 | 3 |
| hsa-miR-181a-5p | MIMAT0000256 | DAAM1 | XM_005267431 | 0 | 1 | 0 | 1 | 1 | 3 |
| hsa-miR-181a-5p | MIMAT0000256 | FBXO21 | XM_005253857 | 1 | 1 | 0 | 0 | 1 | 3 |
| hsa-miR-181a-5p | MIMAT0000256 | PALLD | XM_005262863 | 0 | 1 | 0 | 1 | 1 | 3 |
| hsa-miR-181a-5p | MIMAT0000256 | ZNF292 | XM_005248697 | 1 | 1 | 0 | 0 | 1 | 3 |
| hsa-miR-181a-5p | MIMAT0000256 | PDXDC1 | XM_005255174 | 1 | 1 | 0 | 0 | 1 | 3 |
| hsa-miR-181a-5p | MIMAT0000256 | CLUAP1 | NM_015041 | 1 | 1 | 0 | 0 | 1 | 3 |
| hsa-miR-181a-5p | MIMAT0000256 | AVL9 | XM_005249668 | 1 | 1 | 0 | 0 | 1 | 3 |
| hsa-miR-181a-5p | MIMAT0000256 | TBC1D2B | NM_144572 | 1 | 0 | 0 | 1 | 1 | 3 |
| hsa-miR-181a-5p | MIMAT0000256 | TAB2 | NM_015093 | 0 | 1 | 0 | 1 | 1 | 3 |
| hsa-miR-181a-5p | MIMAT0000256 | CAMTA2 | XM_005256548 | 0 | 1 | 0 | 1 | 1 | 3 |
| hsa-miR-181a-5p | MIMAT0000256 | EPB41L3 | NM_012307 | 0 | 1 | 0 | 1 | 1 | 3 |
| hsa-miR-181a-5p | MIMAT0000256 | GPD1L | NM_015141 | 0 | 1 | 1 | 0 | 1 | 3 |
| hsa-miR-181a-5p | MIMAT0000256 | ZCCHC14 | NM_015144 | 1 | 0 | 0 | 1 | 1 | 3 |
| hsa-miR-181a-5p | MIMAT0000256 | LARP4B | NM_015155 | 1 | 0 | 0 | 1 | 1 | 3 |
| hsa-miR-181a-5p | MIMAT0000256 | KANK1 | XM_005251410 | 0 | 1 | 0 | 1 | 1 | 3 |
| hsa-miR-181a-5p | MIMAT0000256 | ATP11B | XM_005247242 | 1 | 1 | 0 | 0 | 1 | 3 |
| hsa-miR-181a-5p | MIMAT0000256 | JMJD6 | NM_001081461 | 0 | 1 | 0 | 1 | 1 | 3 |
| hsa-miR-181a-5p | MIMAT0000256 | PLCL2 | NM_001144382 | 0 | 1 | 1 | 0 | 1 | 3 |
| hsa-miR-181a-5p | MIMAT0000256 | VPS13A | NM_033305 | 0 | 1 | 1 | 0 | 1 | 3 |
| hsa-miR-181a-5p | MIMAT0000256 | COBL | XM_005271749 | 0 | 1 | 0 | 1 | 1 | 3 |
| hsa-miR-181a-5p | MIMAT0000256 | KIAA1024 | NM_015206 | 1 | 1 | 0 | 0 | 1 | 3 |
| hsa-miR-181a-5p | MIMAT0000256 | OTUD3 | NM_015207 | 0 | 1 | 0 | 1 | 1 | 3 |
| hsa-miR-181a-5p | MIMAT0000256 | ANKRD12 | NM_001204056 | 1 | 1 | 0 | 0 | 1 | 3 |
| hsa-miR-181a-5p | MIMAT0000256 | CAMSAP2 | XM_005245040 | 1 | 1 | 0 | 0 | 1 | 3 |
| hsa-miR-181a-5p | MIMAT0000256 | ATMIN | NM_015251 | 0 | 1 | 0 | 1 | 1 | 3 |
| hsa-miR-181a-5p | MIMAT0000256 | FNBP4 | XM_005252833 | 0 | 1 | 0 | 1 | 1 | 3 |
| hsa-miR-181a-5p | MIMAT0000256 | SIK3 | XM_005271481 | 1 | 1 | 0 | 0 | 1 | 3 |
| hsa-miR-181a-5p | MIMAT0000256 | ZDHHC17 | NM_015336 | 1 | 0 | 0 | 1 | 1 | 3 |
| hsa-miR-181a-5p | MIMAT0000256 | SLC35A3 | NM_012243 | 0 | 1 | 0 | 1 | 1 | 3 |
| hsa-miR-181a-5p | MIMAT0000256 | HEY1 | NM_001040708 | 0 | 1 | 0 | 1 | 1 | 3 |
| hsa-miR-181a-5p | MIMAT0000256 | TMEM131 | XM_005263909 | 0 | 1 | 0 | 1 | 1 | 3 |
| hsa-miR-181a-5p | MIMAT0000256 | KCTD2 | NM_015353 | 0 | 1 | 0 | 1 | 1 | 3 |
| hsa-miR-181a-5p | MIMAT0000256 | MORC3 | NM_015358 | 0 | 1 | 1 | 0 | 1 | 3 |
| hsa-miR-181a-5p | MIMAT0000256 | CCNDBP1 | NM_012142 | 0 | 1 | 1 | 0 | 1 | 3 |
| hsa-miR-181a-5p | MIMAT0000256 | LEMD3 | NM_014319 | 0 | 1 | 0 | 1 | 1 | 3 |
| hsa-miR-181a-5p | MIMAT0000256 | CD2AP | NM_012120 | 1 | 1 | 0 | 0 | 1 | 3 |
| hsa-miR-181a-5p | MIMAT0000256 | MKRN1 | NM_013446 | 0 | 1 | 0 | 1 | 1 | 3 |
| hsa-miR-181a-5p | MIMAT0000256 | PRND | NM_012409 | 0 | 1 | 0 | 1 | 1 | 3 |
| hsa-miR-181a-5p | MIMAT0000256 | SGK3 | NM_001033578 | 1 | 1 | 0 | 0 | 1 | 3 |
| hsa-miR-181a-5p | MIMAT0000256 | FLRT2 | XM_005267490 | 1 | 0 | 0 | 1 | 1 | 3 |
| hsa-miR-181a-5p | MIMAT0000256 | RAD54B | NM_001205262 | 1 | 1 | 0 | 0 | 1 | 3 |
| hsa-miR-181a-5p | MIMAT0000256 | NIPBL | NM_015384 | 1 | 1 | 0 | 0 | 1 | 3 |
| hsa-miR-181a-5p | MIMAT0000256 | ABTB2 | NM_145804 | 0 | 1 | 0 | 1 | 1 | 3 |
| hsa-miR-181a-5p | MIMAT0000256 | MOB4 | NM_199482 | 1 | 1 | 0 | 0 | 1 | 3 |
| hsa-miR-181a-5p | MIMAT0000256 | PARM1 | NM_015393 | 0 | 1 | 1 | 0 | 1 | 3 |
| hsa-miR-181a-5p | MIMAT0000256 | ZNF345 | NM_003419 | 1 | 1 | 0 | 0 | 1 | 3 |
| hsa-miR-181a-5p | MIMAT0000256 | IFFO1 | NM_001193457 | 0 | 1 | 0 | 1 | 1 | 3 |
| hsa-miR-181a-5p | MIMAT0000256 | ATL3 | XM_005273890 | 1 | 1 | 0 | 0 | 1 | 3 |
| hsa-miR-181a-5p | MIMAT0000256 | PNISR | XM_005266915 | 0 | 1 | 0 | 1 | 1 | 3 |
| hsa-miR-181a-5p | MIMAT0000256 | IPCEF1 | NM_001130699 | 1 | 0 | 0 | 1 | 1 | 3 |
| hsa-miR-181a-5p | MIMAT0000256 | LTN1 | NM_015565 | 0 | 1 | 0 | 1 | 1 | 3 |
| hsa-miR-181a-5p | MIMAT0000256 | GIGYF2 | NM_015575 | 1 | 0 | 0 | 1 | 1 | 3 |
| hsa-miR-181a-5p | MIMAT0000256 | ERC2 | XM_005265036 | 0 | 1 | 0 | 1 | 1 | 3 |
| hsa-miR-181a-5p | MIMAT0000256 | GPSM1 | NM_001145638 | 0 | 1 | 1 | 0 | 1 | 3 |
| hsa-miR-181a-5p | MIMAT0000256 | KLHL3 | NM_001257194 | 0 | 1 | 0 | 1 | 1 | 3 |
| hsa-miR-181a-5p | MIMAT0000256 | RPS6KC1 | XM_005273096 | 1 | 1 | 0 | 0 | 1 | 3 |
| hsa-miR-181a-5p | MIMAT0000256 | NBEA | NM_015678 | 1 | 0 | 0 | 1 | 1 | 3 |
| hsa-miR-181a-5p | MIMAT0000256 | RNF11 | NM_014372 | 1 | 0 | 0 | 1 | 1 | 3 |
| hsa-miR-181a-5p | MIMAT0000256 | AFF4 | XM_005271963 | 1 | 1 | 0 | 0 | 1 | 3 |
| hsa-miR-181a-5p | MIMAT0000256 | SNX5 | XM_005260706 | 0 | 1 | 0 | 1 | 1 | 3 |
| hsa-miR-181a-5p | MIMAT0000256 | ARFIP1 | NM_001025595 | 1 | 1 | 0 | 0 | 1 | 3 |
| hsa-miR-181a-5p | MIMAT0000256 | RNF115 | NM_014455 | 1 | 1 | 0 | 0 | 1 | 3 |
| hsa-miR-181a-5p | MIMAT0000256 | RAB30 | XM_005273911 | 1 | 1 | 0 | 0 | 1 | 3 |
| hsa-miR-181a-5p | MIMAT0000256 | CECR2 | NM_031413 | 0 | 1 | 0 | 1 | 1 | 3 |
| hsa-miR-181a-5p | MIMAT0000256 | KLF15 | NM_014079 | 0 | 1 | 1 | 0 | 1 | 3 |
| hsa-miR-181a-5p | MIMAT0000256 | LGALSL | NM_014181 | 0 | 1 | 1 | 0 | 1 | 3 |
| hsa-miR-181a-5p | MIMAT0000256 | DSE | XM_005266951 | 1 | 1 | 0 | 0 | 1 | 3 |
| hsa-miR-181a-5p | MIMAT0000256 | LRP12 | NM_013437 | 0 | 1 | 1 | 0 | 1 | 3 |
| hsa-miR-181a-5p | MIMAT0000256 | ERO1L | NM_014584 | 1 | 1 | 0 | 0 | 1 | 3 |
| hsa-miR-181a-5p | MIMAT0000256 | ZBTB21 | XM_005261122 | 1 | 0 | 0 | 1 | 1 | 3 |
| hsa-miR-181a-5p | MIMAT0000256 | RRM2B | NM_015713 | 1 | 0 | 0 | 1 | 1 | 3 |
| hsa-miR-181a-5p | MIMAT0000256 | MINK1 | XM_005256664 | 0 | 1 | 0 | 1 | 1 | 3 |
| hsa-miR-181a-5p | MIMAT0000256 | ITSN2 | XM_005264337 | 1 | 1 | 0 | 0 | 1 | 3 |
| hsa-miR-181a-5p | MIMAT0000256 | AK3 | NM_016282 | 1 | 1 | 0 | 0 | 1 | 3 |
| hsa-miR-181a-5p | MIMAT0000256 | RNF141 | NM_016422 | 1 | 1 | 0 | 0 | 1 | 3 |
| hsa-miR-181a-5p | MIMAT0000256 | CDON | XM_005271584 | 0 | 1 | 0 | 1 | 1 | 3 |
| hsa-miR-181a-5p | MIMAT0000256 | TMED5 | NM_001167830 | 0 | 1 | 0 | 1 | 1 | 3 |
| hsa-miR-181a-5p | MIMAT0000256 | VPS36 | NM_016075 | 1 | 1 | 0 | 0 | 1 | 3 |
| hsa-miR-181a-5p | MIMAT0000256 | DESI2 | NM_016076 | 1 | 1 | 0 | 0 | 1 | 3 |
| hsa-miR-181a-5p | MIMAT0000256 | TXNDC12 | NM_015913 | 0 | 1 | 0 | 1 | 1 | 3 |
| hsa-miR-181a-5p | MIMAT0000256 | GOLGA7 | NM_001002296 | 1 | 1 | 0 | 0 | 1 | 3 |
| hsa-miR-181a-5p | MIMAT0000256 | SAR1B | NM_001033503 | 0 | 1 | 0 | 1 | 1 | 3 |
| hsa-miR-181a-5p | MIMAT0000256 | RLIM | NM_183353 | 1 | 1 | 0 | 0 | 1 | 3 |
| hsa-miR-181a-5p | MIMAT0000256 | KCTD3 | XM_005273158 | 0 | 1 | 0 | 1 | 1 | 3 |
| hsa-miR-181a-5p | MIMAT0000256 | CCDC41 | NM_016122 | 1 | 1 | 0 | 0 | 1 | 3 |
| hsa-miR-181a-5p | MIMAT0000256 | SS18L2 | XM_005265203 | 0 | 1 | 0 | 1 | 1 | 3 |
| hsa-miR-181a-5p | MIMAT0000256 | CLDN18 | NM_016369 | 1 | 1 | 0 | 0 | 1 | 3 |
| hsa-miR-181a-5p | MIMAT0000256 | ZNF571 | XM_005258977 | 0 | 1 | 0 | 1 | 1 | 3 |
| hsa-miR-181a-5p | MIMAT0000256 | FKBP11 | XM_005268950 | 1 | 1 | 0 | 0 | 1 | 3 |
| hsa-miR-181a-5p | MIMAT0000256 | SLC25A37 | XM_005273526 | 1 | 1 | 0 | 0 | 1 | 3 |
| hsa-miR-181a-5p | MIMAT0000256 | HEMK1 | NM_016173 | 0 | 1 | 0 | 1 | 1 | 3 |
| hsa-miR-181a-5p | MIMAT0000256 | YTHDF2 | NM_016258 | 1 | 1 | 0 | 0 | 1 | 3 |
| hsa-miR-181a-5p | MIMAT0000256 | SFMBT1 | XM_005265220 | 1 | 1 | 0 | 0 | 1 | 3 |
| hsa-miR-181a-5p | MIMAT0000256 | UBE2J1 | NM_016021 | 1 | 1 | 0 | 0 | 1 | 3 |
| hsa-miR-181a-5p | MIMAT0000256 | MRPS23 | NM_016070 | 1 | 1 | 0 | 0 | 1 | 3 |
| hsa-miR-181a-5p | MIMAT0000256 | CHMP3 | NM_016079 | 0 | 1 | 0 | 1 | 1 | 3 |
| hsa-miR-181a-5p | MIMAT0000256 | SUFU | NM_016169 | 0 | 1 | 0 | 1 | 1 | 3 |
| hsa-miR-181a-5p | MIMAT0000256 | ZNF44 | NM_001164276 | 0 | 1 | 1 | 0 | 1 | 3 |
| hsa-miR-181a-5p | MIMAT0000256 | ATP8A2 | XM_005266419 | 1 | 1 | 0 | 0 | 1 | 3 |
| hsa-miR-181a-5p | MIMAT0000256 | RAB8B | NM_016530 | 0 | 1 | 0 | 1 | 1 | 3 |
| hsa-miR-181a-5p | MIMAT0000256 | SLC37A1 | XM_005261140 | 1 | 1 | 0 | 0 | 1 | 3 |
| hsa-miR-181a-5p | MIMAT0000256 | DCUN1D1 | XM_005247539 | 1 | 1 | 0 | 0 | 1 | 3 |
| hsa-miR-181a-5p | MIMAT0000256 | GDAP1 | NM_018972 | 1 | 1 | 0 | 0 | 1 | 3 |
| hsa-miR-181a-5p | MIMAT0000256 | HAO1 | NM_017545 | 0 | 1 | 1 | 0 | 1 | 3 |
| hsa-miR-181a-5p | MIMAT0000256 | RBM27 | XM_005268466 | 1 | 1 | 0 | 0 | 1 | 3 |
| hsa-miR-181a-5p | MIMAT0000256 | ZFAND6 | NM_019006 | 0 | 1 | 1 | 0 | 1 | 3 |
| hsa-miR-181a-5p | MIMAT0000256 | NEURL1B | NM_001142651 | 0 | 1 | 0 | 1 | 1 | 3 |
| hsa-miR-181a-5p | MIMAT0000256 | TMX3 | NM_019022 | 1 | 1 | 0 | 0 | 1 | 3 |
| hsa-miR-181a-5p | MIMAT0000256 | DHX29 | NM_019030 | 0 | 1 | 1 | 0 | 1 | 3 |
| hsa-miR-181a-5p | MIMAT0000256 | RSBN1 | NM_018364 | 1 | 1 | 0 | 0 | 1 | 3 |
| hsa-miR-181a-5p | MIMAT0000256 | LEPROT | XM_005270951 | 1 | 1 | 0 | 0 | 1 | 3 |
| hsa-miR-181a-5p | MIMAT0000256 | TRIM44 | NM_017583 | 1 | 1 | 0 | 0 | 1 | 3 |
| hsa-miR-181a-5p | MIMAT0000256 | ZNF562 | NM_001130031 | 0 | 1 | 1 | 0 | 1 | 3 |
| hsa-miR-181a-5p | MIMAT0000256 | AFTPH | NM_203437 | 0 | 1 | 1 | 0 | 1 | 3 |
| hsa-miR-181a-5p | MIMAT0000256 | MFSD6 | NM_017694 | 0 | 1 | 1 | 0 | 1 | 3 |
| hsa-miR-181a-5p | MIMAT0000256 | UHRF1BP1 | NM_017754 | 1 | 0 | 0 | 1 | 1 | 3 |
| hsa-miR-181a-5p | MIMAT0000256 | RNF43 | NM_017763 | 0 | 1 | 0 | 1 | 1 | 3 |
| hsa-miR-181a-5p | MIMAT0000256 | ELOVL2 | NM_017770 | 1 | 0 | 0 | 1 | 1 | 3 |
| hsa-miR-181a-5p | MIMAT0000256 | CMTM6 | NM_017801 | 0 | 1 | 0 | 1 | 1 | 3 |
| hsa-miR-181a-5p | MIMAT0000256 | IMPAD1 | NM_017813 | 1 | 0 | 0 | 1 | 1 | 3 |
| hsa-miR-181a-5p | MIMAT0000256 | FAM120C | NM_017848 | 0 | 1 | 0 | 1 | 1 | 3 |
| hsa-miR-181a-5p | MIMAT0000256 | PHIP | NM_017934 | 1 | 1 | 0 | 0 | 1 | 3 |
| hsa-miR-181a-5p | MIMAT0000256 | FBXO34 | NM_017943 | 0 | 1 | 1 | 0 | 1 | 3 |
| hsa-miR-181a-5p | MIMAT0000256 | USP47 | XM_005252997 | 0 | 1 | 0 | 1 | 1 | 3 |
| hsa-miR-181a-5p | MIMAT0000256 | SLC38A4 | NM_018018 | 1 | 1 | 0 | 0 | 1 | 3 |
| hsa-miR-181a-5p | MIMAT0000256 | ATG2B | NM_018036 | 1 | 1 | 0 | 0 | 1 | 3 |
| hsa-miR-181a-5p | MIMAT0000256 | ANO1 | NM_018043 | 0 | 1 | 1 | 0 | 1 | 3 |
| hsa-miR-181a-5p | MIMAT0000256 | AGGF1 | NM_018046 | 0 | 1 | 0 | 1 | 1 | 3 |
| hsa-miR-181a-5p | MIMAT0000256 | PLEKHJ1 | XM_005259590 | 0 | 1 | 0 | 1 | 1 | 3 |
| hsa-miR-181a-5p | MIMAT0000256 | TMEM33 | NM_018126 | 0 | 1 | 1 | 0 | 1 | 3 |
| hsa-miR-181a-5p | MIMAT0000256 | SLC25A36 | XM_005247575 | 1 | 1 | 0 | 0 | 1 | 3 |
| hsa-miR-181a-5p | MIMAT0000256 | KIAA1551 | NM_018169 | 0 | 1 | 0 | 1 | 1 | 3 |
| hsa-miR-181a-5p | MIMAT0000256 | RPRD1A | XM_005258294 | 0 | 1 | 0 | 1 | 1 | 3 |
| hsa-miR-181a-5p | MIMAT0000256 | APPL2 | NM_001251905 | 0 | 1 | 0 | 1 | 1 | 3 |
| hsa-miR-181a-5p | MIMAT0000256 | LGI2 | NM_018176 | 0 | 1 | 0 | 1 | 1 | 3 |
| hsa-miR-181a-5p | MIMAT0000256 | TMEM19 | NM_018279 | 1 | 0 | 0 | 1 | 1 | 3 |
| hsa-miR-181a-5p | MIMAT0000256 | TMEM144 | NM_018342 | 0 | 1 | 0 | 1 | 1 | 3 |
| hsa-miR-181a-5p | MIMAT0000256 | TMA16 | NM_018352 | 1 | 1 | 0 | 0 | 1 | 3 |
| hsa-miR-181a-5p | MIMAT0000256 | LIN7C | NM_018362 | 1 | 1 | 0 | 0 | 1 | 3 |
| hsa-miR-181a-5p | MIMAT0000256 | ACER3 | NM_018367 | 1 | 1 | 0 | 0 | 1 | 3 |
| hsa-miR-181a-5p | MIMAT0000256 | DRAM1 | NM_018370 | 0 | 1 | 0 | 1 | 1 | 3 |
| hsa-miR-181a-5p | MIMAT0000256 | LAPTM4B | NM_018407 | 0 | 1 | 1 | 0 | 1 | 3 |
| hsa-miR-181a-5p | MIMAT0000256 | PI4K2A | NM_018425 | 0 | 1 | 0 | 1 | 1 | 3 |
| hsa-miR-181a-5p | MIMAT0000256 | DNAJA4 | NM_018602 | 0 | 1 | 1 | 0 | 1 | 3 |
| hsa-miR-181a-5p | MIMAT0000256 | ETNK1 | NM_018638 | 1 | 1 | 0 | 0 | 1 | 3 |
| hsa-miR-181a-5p | MIMAT0000256 | CNOT11 | NM_017546 | 1 | 1 | 0 | 0 | 1 | 3 |
| hsa-miR-181a-5p | MIMAT0000256 | FAM46A | XM_005248731 | 0 | 1 | 0 | 1 | 1 | 3 |
| hsa-miR-181a-5p | MIMAT0000256 | THUMPD1 | NM_017736 | 1 | 0 | 0 | 1 | 1 | 3 |
| hsa-miR-181a-5p | MIMAT0000256 | PNRC2 | NM_017761 | 0 | 1 | 1 | 0 | 1 | 3 |
| hsa-miR-181a-5p | MIMAT0000256 | TMEM127 | NM_017849 | 0 | 1 | 0 | 1 | 1 | 3 |
| hsa-miR-181a-5p | MIMAT0000256 | PRPF40A | XM_005246679 | 0 | 1 | 0 | 1 | 1 | 3 |
| hsa-miR-181a-5p | MIMAT0000256 | RBM22 | XM_005268479 | 0 | 1 | 0 | 1 | 1 | 3 |
| hsa-miR-181a-5p | MIMAT0000256 | CCDC88A | NM_001135597 | 1 | 0 | 0 | 1 | 1 | 3 |
| hsa-miR-181a-5p | MIMAT0000256 | ENAH | NM_001008493 | 1 | 1 | 0 | 0 | 1 | 3 |
| hsa-miR-181a-5p | MIMAT0000256 | TMEM184C | NM_018241 | 0 | 1 | 1 | 0 | 1 | 3 |
| hsa-miR-181a-5p | MIMAT0000256 | TRERF1 | NM_033502 | 0 | 1 | 0 | 1 | 1 | 3 |
| hsa-miR-181a-5p | MIMAT0000256 | CAND1 | NM_018448 | 1 | 1 | 0 | 0 | 1 | 3 |
| hsa-miR-181a-5p | MIMAT0000256 | TEX2 | XM_005257504 | 1 | 1 | 0 | 0 | 1 | 3 |
| hsa-miR-181a-5p | MIMAT0000256 | HDAC8 | NM_001166419 | 0 | 1 | 0 | 1 | 1 | 3 |
| hsa-miR-181a-5p | MIMAT0000256 | KLHL4 | NM_019117 | 1 | 0 | 0 | 1 | 1 | 3 |
| hsa-miR-181a-5p | MIMAT0000256 | PCDHB6 | NM_018939 | 1 | 1 | 0 | 0 | 1 | 3 |
| hsa-miR-181a-5p | MIMAT0000256 | ZNF253 | XM_005259990 | 1 | 1 | 0 | 0 | 1 | 3 |
| hsa-miR-181a-5p | MIMAT0000256 | ERO1LB | NM_019891 | 1 | 1 | 0 | 0 | 1 | 3 |
| hsa-miR-181a-5p | MIMAT0000256 | ASAH2 | NM_019893 | 0 | 1 | 0 | 1 | 1 | 3 |
| hsa-miR-181a-5p | MIMAT0000256 | TRIM39 | XM_005249228 | 1 | 0 | 0 | 1 | 1 | 3 |
| hsa-miR-181a-5p | MIMAT0000256 | MUC13 | NM_033049 | 1 | 1 | 0 | 0 | 1 | 3 |
| hsa-miR-181a-5p | MIMAT0000256 | ZC3HAV1 | XM_005250501 | 1 | 1 | 0 | 0 | 1 | 3 |
| hsa-miR-181a-5p | MIMAT0000256 | AGPAT4 | NM_020133 | 0 | 1 | 0 | 1 | 1 | 3 |
| hsa-miR-181a-5p | MIMAT0000256 | SPIRE1 | XM_005258122 | 0 | 1 | 0 | 1 | 1 | 3 |
| hsa-miR-181a-5p | MIMAT0000256 | SMARCAD1 | NM_001128429 | 1 | 1 | 0 | 0 | 1 | 3 |
| hsa-miR-181a-5p | MIMAT0000256 | SMCO4 | NM_020179 | 1 | 1 | 0 | 0 | 1 | 3 |
| hsa-miR-181a-5p | MIMAT0000256 | DUSP22 | XM_005249235 | 1 | 1 | 0 | 0 | 1 | 3 |
| hsa-miR-181a-5p | MIMAT0000256 | LHX9 | XM_005245350 | 1 | 1 | 0 | 0 | 1 | 3 |
| hsa-miR-181a-5p | MIMAT0000256 | RGMA | NM_020211 | 0 | 1 | 0 | 1 | 1 | 3 |
| hsa-miR-181a-5p | MIMAT0000256 | TULP4 | XM_005267057 | 0 | 1 | 0 | 1 | 1 | 3 |
| hsa-miR-181a-5p | MIMAT0000256 | ENTPD7 | NM_020354 | 1 | 1 | 0 | 0 | 1 | 3 |
| hsa-miR-181a-5p | MIMAT0000256 | ANKRD50 | NM_020337 | 1 | 1 | 0 | 0 | 1 | 3 |
| hsa-miR-181a-5p | MIMAT0000256 | RALGAPA2 | XM_005260768 | 1 | 1 | 0 | 0 | 1 | 3 |
| hsa-miR-181a-5p | MIMAT0000256 | THOC2 | XM_005262449 | 0 | 1 | 0 | 1 | 1 | 3 |
| hsa-miR-181a-5p | MIMAT0000256 | KIAA1199 | XM_005254567 | 1 | 1 | 0 | 0 | 1 | 3 |
| hsa-miR-181a-5p | MIMAT0000256 | SMEK2 | NM_001122964 | 1 | 1 | 0 | 0 | 1 | 3 |
| hsa-miR-181a-5p | MIMAT0000256 | LYRM2 | NM_020466 | 0 | 1 | 0 | 1 | 1 | 3 |
| hsa-miR-181a-5p | MIMAT0000256 | AICDA | NM_020661 | 1 | 1 | 0 | 0 | 1 | 3 |
| hsa-miR-181a-5p | MIMAT0000256 | TMEM27 | NM_020665 | 0 | 1 | 1 | 0 | 1 | 3 |
| hsa-miR-181a-5p | MIMAT0000256 | C3orf14 | XM_005265339 | 1 | 1 | 0 | 0 | 1 | 3 |
| hsa-miR-181a-5p | MIMAT0000256 | SLC24A3 | NM_020689 | 1 | 1 | 0 | 0 | 1 | 3 |
| hsa-miR-181a-5p | MIMAT0000256 | NDRG2 | XM_005267896 | 1 | 1 | 0 | 0 | 1 | 3 |
| hsa-miR-181a-5p | MIMAT0000256 | TBC1D24 | NM_001199107 | 0 | 1 | 1 | 0 | 1 | 3 |
| hsa-miR-181a-5p | MIMAT0000256 | SLC12A5 | NM_020708 | 1 | 1 | 0 | 0 | 1 | 3 |
| hsa-miR-181a-5p | MIMAT0000256 | ERMN | NM_020711 | 0 | 1 | 0 | 1 | 1 | 3 |
| hsa-miR-181a-5p | MIMAT0000256 | ARRDC3 | NM_020801 | 1 | 1 | 0 | 0 | 1 | 3 |
| hsa-miR-181a-5p | MIMAT0000256 | SIPA1L2 | XM_005273211 | 0 | 1 | 0 | 1 | 1 | 3 |
| hsa-miR-181a-5p | MIMAT0000256 | TRMT5 | XM_005267916 | 1 | 1 | 0 | 0 | 1 | 3 |
| hsa-miR-181a-5p | MIMAT0000256 | ZNF471 | XM_005259098 | 1 | 1 | 0 | 0 | 1 | 3 |
| hsa-miR-181a-5p | MIMAT0000256 | FNIP2 | XM_005263156 | 1 | 1 | 0 | 0 | 1 | 3 |
| hsa-miR-181a-5p | MIMAT0000256 | KIAA1462 | NM_020848 | 0 | 1 | 0 | 1 | 1 | 3 |
| hsa-miR-181a-5p | MIMAT0000256 | NYAP2 | XM_005246708 | 1 | 0 | 0 | 1 | 1 | 3 |
| hsa-miR-181a-5p | MIMAT0000256 | DPP10 | NM_001178036 | 0 | 1 | 0 | 1 | 1 | 3 |
| hsa-miR-181a-5p | MIMAT0000256 | SLC7A14 | NM_020949 | 1 | 1 | 0 | 0 | 1 | 3 |
| hsa-miR-181a-5p | MIMAT0000256 | RBAK | NM_021163 | 0 | 1 | 0 | 1 | 1 | 3 |
| hsa-miR-181a-5p | MIMAT0000256 | PTBP2 | NM_021190 | 0 | 1 | 1 | 0 | 1 | 3 |
| hsa-miR-181a-5p | MIMAT0000256 | RPRD1B | NM_021215 | 0 | 1 | 0 | 1 | 1 | 3 |
| hsa-miR-181a-5p | MIMAT0000256 | SELK | NM_021237 | 0 | 1 | 1 | 0 | 1 | 3 |
| hsa-miR-181a-5p | MIMAT0000256 | SENP2 | XM_005247689 | 1 | 1 | 0 | 0 | 1 | 3 |
| hsa-miR-181a-5p | MIMAT0000256 | AFAP1 | NM_001134647 | 1 | 0 | 0 | 1 | 1 | 3 |
| hsa-miR-181a-5p | MIMAT0000256 | MRPS35 | NM_021821 | 0 | 1 | 1 | 0 | 1 | 3 |
| hsa-miR-181a-5p | MIMAT0000256 | FAM204A | XM_005270024 | 1 | 1 | 0 | 0 | 1 | 3 |
| hsa-miR-181a-5p | MIMAT0000256 | NAPB | NM_022080 | 1 | 1 | 0 | 0 | 1 | 3 |
| hsa-miR-181a-5p | MIMAT0000256 | XPNPEP3 | NM_022098 | 0 | 1 | 0 | 1 | 1 | 3 |
| hsa-miR-181a-5p | MIMAT0000256 | SLC28A3 | NM_001199633 | 1 | 1 | 0 | 0 | 1 | 3 |
| hsa-miR-181a-5p | MIMAT0000256 | NCAPG | NM_022346 | 1 | 1 | 0 | 0 | 1 | 3 |
| hsa-miR-181a-5p | MIMAT0000256 | ERAP2 | NM_022350 | 0 | 1 | 1 | 0 | 1 | 3 |
| hsa-miR-181a-5p | MIMAT0000256 | NECAB1 | NM_022351 | 1 | 1 | 0 | 0 | 1 | 3 |
| hsa-miR-181a-5p | MIMAT0000256 | SOX17 | NM_022454 | 1 | 1 | 0 | 0 | 1 | 3 |
| hsa-miR-181a-5p | MIMAT0000256 | LMBR1 | XM_005249554 | 1 | 1 | 0 | 0 | 1 | 3 |
| hsa-miR-181a-5p | MIMAT0000256 | IKZF5 | NM_001271840 | 1 | 1 | 0 | 0 | 1 | 3 |
| hsa-miR-181a-5p | MIMAT0000256 | HHIP | XM_005263178 | 1 | 1 | 0 | 0 | 1 | 3 |
| hsa-miR-181a-5p | MIMAT0000256 | TMEM168 | XM_005250526 | 1 | 0 | 0 | 1 | 1 | 3 |
| hsa-miR-181a-5p | MIMAT0000256 | GIGYF1 | NM_022574 | 0 | 1 | 0 | 1 | 1 | 3 |
| hsa-miR-181a-5p | MIMAT0000256 | EBF2 | NM_022659 | 1 | 1 | 0 | 0 | 1 | 3 |
| hsa-miR-181a-5p | MIMAT0000256 | S100PBP | XM_005271121 | 0 | 1 | 0 | 1 | 1 | 3 |
| hsa-miR-181a-5p | MIMAT0000256 | TBC1D15 | NM_001146214 | 1 | 1 | 0 | 0 | 1 | 3 |
| hsa-miR-181a-5p | MIMAT0000256 | USP46 | NM_001134223 | 1 | 1 | 0 | 0 | 1 | 3 |
| hsa-miR-181a-5p | MIMAT0000256 | PAPOLG | NM_022894 | 0 | 1 | 1 | 0 | 1 | 3 |
| hsa-miR-181a-5p | MIMAT0000256 | WNK1 | XM_005253733 | 0 | 1 | 0 | 1 | 1 | 3 |
| hsa-miR-181a-5p | MIMAT0000256 | WNK3 | NM_020922 | 1 | 0 | 0 | 1 | 1 | 3 |
| hsa-miR-181a-5p | MIMAT0000256 | BOLL | NM_033030 | 0 | 1 | 1 | 0 | 1 | 3 |
| hsa-miR-181a-5p | MIMAT0000256 | OTUB2 | NM_023112 | 0 | 1 | 0 | 1 | 1 | 3 |
| hsa-miR-181a-5p | MIMAT0000256 | ZNF655 | XM_005250577 | 0 | 1 | 0 | 1 | 1 | 3 |
| hsa-miR-181a-5p | MIMAT0000256 | TSEN34 | NM_024075 | 1 | 1 | 0 | 0 | 1 | 3 |
| hsa-miR-181a-5p | MIMAT0000256 | SMIM7 | NM_024104 | 1 | 1 | 0 | 0 | 1 | 3 |
| hsa-miR-181a-5p | MIMAT0000256 | GLB1L | XM_005246850 | 0 | 1 | 0 | 1 | 1 | 3 |
| hsa-miR-181a-5p | MIMAT0000256 | CEP97 | XM_005247758 | 1 | 1 | 0 | 0 | 1 | 3 |
| hsa-miR-181a-5p | MIMAT0000256 | C22orf46 | NM_001142964 | 1 | 0 | 0 | 1 | 1 | 3 |
| hsa-miR-181a-5p | MIMAT0000256 | PANK3 | NM_024594 | 1 | 1 | 0 | 0 | 1 | 3 |
| hsa-miR-181a-5p | MIMAT0000256 | TBL1XR1 | NM_024665 | 0 | 1 | 1 | 0 | 1 | 3 |
| hsa-miR-181a-5p | MIMAT0000256 | LIN28A | NM_024674 | 0 | 1 | 1 | 0 | 1 | 3 |
| hsa-miR-181a-5p | MIMAT0000256 | MCTP1 | XM_005272083 | 1 | 1 | 0 | 0 | 1 | 3 |
| hsa-miR-181a-5p | MIMAT0000256 | CARF | NM_024744 | 0 | 1 | 0 | 1 | 1 | 3 |
| hsa-miR-181a-5p | MIMAT0000256 | VASH2 | XM_005273268 | 0 | 1 | 0 | 1 | 1 | 3 |
| hsa-miR-181a-5p | MIMAT0000256 | MOB3B | NM_024761 | 1 | 0 | 0 | 1 | 1 | 3 |
| hsa-miR-181a-5p | MIMAT0000256 | TUBAL3 | NM_024803 | 1 | 1 | 0 | 0 | 1 | 3 |
| hsa-miR-181a-5p | MIMAT0000256 | MAP9 | XM_005263223 | 1 | 0 | 0 | 1 | 1 | 3 |
| hsa-miR-181a-5p | MIMAT0000256 | LPCAT1 | NM_024830 | 0 | 1 | 0 | 1 | 1 | 3 |
| hsa-miR-181a-5p | MIMAT0000256 | SLC35E1 | NM_024881 | 0 | 1 | 0 | 1 | 1 | 3 |
| hsa-miR-181a-5p | MIMAT0000256 | ATAT1 | XM_005249420 | 0 | 1 | 0 | 1 | 1 | 3 |
| hsa-miR-181a-5p | MIMAT0000256 | DNAJB14 | NM_001031723 | 1 | 1 | 0 | 0 | 1 | 3 |
| hsa-miR-181a-5p | MIMAT0000256 | TRPM3 | XM_005252217 | 1 | 1 | 0 | 0 | 1 | 3 |
| hsa-miR-181a-5p | MIMAT0000256 | DCAF17 | NM_025000 | 1 | 0 | 0 | 1 | 1 | 3 |
| hsa-miR-181a-5p | MIMAT0000256 | PQLC1 | NM_025078 | 0 | 1 | 0 | 1 | 1 | 3 |
| hsa-miR-181a-5p | MIMAT0000256 | C4orf29 | XM_005263238 | 1 | 1 | 0 | 0 | 1 | 3 |
| hsa-miR-181a-5p | MIMAT0000256 | SPSB1 | NM_025106 | 1 | 1 | 0 | 0 | 1 | 3 |
| hsa-miR-181a-5p | MIMAT0000256 | FBXO11 | XM_005264572 | 0 | 1 | 0 | 1 | 1 | 3 |
| hsa-miR-181a-5p | MIMAT0000256 | CHD9 | XM_005256169 | 0 | 1 | 0 | 1 | 1 | 3 |
| hsa-miR-181a-5p | MIMAT0000256 | NAA50 | NM_025146 | 1 | 1 | 0 | 0 | 1 | 3 |
| hsa-miR-181a-5p | MIMAT0000256 | C16orf70 | NM_025187 | 0 | 1 | 0 | 1 | 1 | 3 |
| hsa-miR-181a-5p | MIMAT0000256 | CXXC4 | NM_025212 | 1 | 0 | 0 | 1 | 1 | 3 |
| hsa-miR-181a-5p | MIMAT0000256 | COASY | XM_005257702 | 1 | 1 | 0 | 0 | 1 | 3 |
| hsa-miR-181a-5p | MIMAT0000256 | ASXL3 | XM_005258356 | 1 | 1 | 0 | 0 | 1 | 3 |
| hsa-miR-181a-5p | MIMAT0000256 | KIAA1715 | NM_030650 | 0 | 1 | 1 | 0 | 1 | 3 |
| hsa-miR-181a-5p | MIMAT0000256 | SLC25A32 | NM_030780 | 0 | 1 | 1 | 0 | 1 | 3 |
| hsa-miR-181a-5p | MIMAT0000256 | SYNC | NM_030786 | 0 | 1 | 0 | 1 | 1 | 3 |
| hsa-miR-181a-5p | MIMAT0000256 | FAM49A | NM_030797 | 0 | 1 | 0 | 1 | 1 | 3 |
| hsa-miR-181a-5p | MIMAT0000256 | SNX27 | XM_005245511 | 0 | 1 | 0 | 1 | 1 | 3 |
| hsa-miR-181a-5p | MIMAT0000256 | TSPAN14 | XM_005270192 | 1 | 1 | 0 | 0 | 1 | 3 |
| hsa-miR-181a-5p | MIMAT0000256 | SPRY4 | XM_005268517 | 0 | 1 | 0 | 1 | 1 | 3 |
| hsa-miR-181a-5p | MIMAT0000256 | CHST9 | XM_005258363 | 1 | 1 | 0 | 0 | 1 | 3 |
| hsa-miR-181a-5p | MIMAT0000256 | DNAL1 | NM_001201366 | 1 | 1 | 0 | 0 | 1 | 3 |
| hsa-miR-181a-5p | MIMAT0000256 | JAM3 | NM_032801 | 0 | 1 | 1 | 0 | 1 | 3 |
| hsa-miR-181a-5p | MIMAT0000256 | SYT16 | XM_005268123 | 1 | 1 | 0 | 0 | 1 | 3 |
| hsa-miR-181a-5p | MIMAT0000256 | ZIC4 | NM_032153 | 1 | 1 | 0 | 0 | 1 | 3 |
| hsa-miR-181a-5p | MIMAT0000256 | USP42 | NM_032172 | 0 | 1 | 1 | 0 | 1 | 3 |
| hsa-miR-181a-5p | MIMAT0000256 | ZNRF3 | NM_001206998 | 1 | 0 | 0 | 1 | 1 | 3 |
| hsa-miR-181a-5p | MIMAT0000256 | MEX3B | NM_032246 | 1 | 1 | 0 | 0 | 1 | 3 |
| hsa-miR-181a-5p | MIMAT0000256 | CAMKK1 | XM_005256817 | 0 | 1 | 0 | 1 | 1 | 3 |
| hsa-miR-181a-5p | MIMAT0000256 | HSDL2 | NM_032303 | 1 | 1 | 0 | 0 | 1 | 3 |
| hsa-miR-181a-5p | MIMAT0000256 | BTBD10 | XM_005253164 | 0 | 1 | 0 | 1 | 1 | 3 |
| hsa-miR-181a-5p | MIMAT0000256 | DDI2 | XM_005246017 | 1 | 1 | 0 | 0 | 1 | 3 |
| hsa-miR-181a-5p | MIMAT0000256 | ZNF559 | NM_001202409 | 0 | 1 | 1 | 0 | 1 | 3 |
| hsa-miR-181a-5p | MIMAT0000256 | SRRM4 | NM_194286 | 0 | 1 | 0 | 1 | 1 | 3 |
| hsa-miR-181a-5p | MIMAT0000256 | ACSS1 | NM_032501 | 0 | 1 | 0 | 1 | 1 | 3 |
| hsa-miR-181a-5p | MIMAT0000256 | ST6GAL2 | NM_032528 | 1 | 1 | 0 | 0 | 1 | 3 |
| hsa-miR-181a-5p | MIMAT0000256 | CAPS2 | XM_005269188 | 1 | 1 | 0 | 0 | 1 | 3 |
| hsa-miR-181a-5p | MIMAT0000256 | GTPBP3 | XM_005260120 | 1 | 1 | 0 | 0 | 1 | 3 |
| hsa-miR-181a-5p | MIMAT0000256 | BEX2 | NM_001168399 | 0 | 1 | 0 | 1 | 1 | 3 |
| hsa-miR-181a-5p | MIMAT0000256 | ZNF514 | XM_005264033 | 0 | 1 | 0 | 1 | 1 | 3 |
| hsa-miR-181a-5p | MIMAT0000256 | FAM73B | XM_005252281 | 0 | 1 | 0 | 1 | 1 | 3 |
| hsa-miR-181a-5p | MIMAT0000256 | PLXDC2 | NM_032812 | 0 | 1 | 0 | 1 | 1 | 3 |
| hsa-miR-181a-5p | MIMAT0000256 | SLC35B4 | NM_032826 | 0 | 1 | 0 | 1 | 1 | 3 |
| hsa-miR-181a-5p | MIMAT0000256 | KIAA1671 | XM_005261793 | 0 | 1 | 0 | 1 | 1 | 3 |
| hsa-miR-181a-5p | MIMAT0000256 | EAF1 | NM_033083 | 0 | 1 | 0 | 1 | 1 | 3 |
| hsa-miR-181a-5p | MIMAT0000256 | DCLK3 | NM_033403 | 0 | 1 | 1 | 0 | 1 | 3 |
| hsa-miR-181a-5p | MIMAT0000256 | ZNF518B | NM_053042 | 1 | 0 | 0 | 1 | 1 | 3 |
| hsa-miR-181a-5p | MIMAT0000256 | FHDC1 | XM_005263319 | 0 | 1 | 0 | 1 | 1 | 3 |
| hsa-miR-181a-5p | MIMAT0000256 | EPT1 | NM_033505 | 0 | 1 | 0 | 1 | 1 | 3 |
| hsa-miR-181a-5p | MIMAT0000256 | GTPBP10 | NM_033107 | 0 | 1 | 0 | 1 | 1 | 3 |
| hsa-miR-181a-5p | MIMAT0000256 | HPS4 | NM_022081 | 1 | 1 | 0 | 0 | 1 | 3 |
| hsa-miR-181a-5p | MIMAT0000256 | KBTBD6 | NM_152903 | 0 | 1 | 0 | 1 | 1 | 3 |
| hsa-miR-181a-5p | MIMAT0000256 | ZSWIM1 | NM_080603 | 1 | 1 | 0 | 0 | 1 | 3 |
| hsa-miR-181a-5p | MIMAT0000256 | IL33 | NM_033439 | 1 | 0 | 0 | 1 | 1 | 3 |
| hsa-miR-181a-5p | MIMAT0000256 | DHX57 | NM_198963 | 0 | 1 | 1 | 0 | 1 | 3 |
| hsa-miR-181a-5p | MIMAT0000256 | C12orf29 | NM_001009894 | 0 | 1 | 1 | 0 | 1 | 3 |
| hsa-miR-181a-5p | MIMAT0000256 | CDKN2AIPNL | NM_080656 | 0 | 1 | 1 | 0 | 1 | 3 |
| hsa-miR-181a-5p | MIMAT0000256 | ANKRD40 | NM_052855 | 1 | 0 | 0 | 1 | 1 | 3 |
| hsa-miR-181a-5p | MIMAT0000256 | RASL10B | NM_033315 | 1 | 0 | 0 | 1 | 1 | 3 |
| hsa-miR-181a-5p | MIMAT0000256 | CHURC1 | NM_145165 | 1 | 1 | 0 | 0 | 1 | 3 |
| hsa-miR-181a-5p | MIMAT0000256 | LMBRD2 | NM_001007527 | 0 | 1 | 1 | 0 | 1 | 3 |
| hsa-miR-181a-5p | MIMAT0000256 | MOB1B | XM_005265709 | 1 | 0 | 0 | 1 | 1 | 3 |
| hsa-miR-181a-5p | MIMAT0000256 | PGAP3 | NM_033419 | 0 | 1 | 0 | 1 | 1 | 3 |
| hsa-miR-181a-5p | MIMAT0000256 | TBCK | NM_001163435 | 0 | 1 | 0 | 1 | 1 | 3 |
| hsa-miR-181a-5p | MIMAT0000256 | ARHGAP18 | XM_005267212 | 1 | 0 | 0 | 1 | 1 | 3 |
| hsa-miR-181a-5p | MIMAT0000256 | SFXN5 | XM_005264646 | 0 | 1 | 0 | 1 | 1 | 3 |
| hsa-miR-181a-5p | MIMAT0000256 | SYTL4 | XM_005262226 | 1 | 0 | 0 | 1 | 1 | 3 |
| hsa-miR-181a-5p | MIMAT0000256 | FNIP1 | NM_133372 | 0 | 1 | 1 | 0 | 1 | 3 |
| hsa-miR-181a-5p | MIMAT0000256 | DTD2 | NM_080664 | 1 | 1 | 0 | 0 | 1 | 3 |
| hsa-miR-181a-5p | MIMAT0000256 | NACC1 | NM_052876 | 0 | 1 | 0 | 1 | 1 | 3 |
| hsa-miR-181a-5p | MIMAT0000256 | TMEM106A | XM_005257003 | 0 | 1 | 0 | 1 | 1 | 3 |
| hsa-miR-181a-5p | MIMAT0000256 | ZNF257 | XM_005259723 | 0 | 1 | 0 | 1 | 1 | 3 |
| hsa-miR-181a-5p | MIMAT0000256 | SLC25A25 | NM_001265614 | 0 | 1 | 0 | 1 | 1 | 3 |
| hsa-miR-181a-5p | MIMAT0000256 | SLC26A9 | NM_052934 | 1 | 1 | 0 | 0 | 1 | 3 |
| hsa-miR-181a-5p | MIMAT0000256 | FCRL3 | XM_005244872 | 1 | 1 | 0 | 0 | 1 | 3 |
| hsa-miR-181a-5p | MIMAT0000256 | RAB3C | XM_005248418 | 1 | 1 | 0 | 0 | 1 | 3 |
| hsa-miR-181a-5p | MIMAT0000256 | RMI2 | NM_152308 | 1 | 1 | 0 | 0 | 1 | 3 |
| hsa-miR-181a-5p | MIMAT0000256 | LYSMD3 | XM_005248421 | 1 | 1 | 0 | 0 | 1 | 3 |
| hsa-miR-181a-5p | MIMAT0000256 | NUS1 | NM_138459 | 0 | 1 | 0 | 1 | 1 | 3 |
| hsa-miR-181a-5p | MIMAT0000256 | SLC18B1 | NM_052831 | 0 | 1 | 0 | 1 | 1 | 3 |
| hsa-miR-181a-5p | MIMAT0000256 | SSX2IP | NM_001166417 | 0 | 1 | 1 | 0 | 1 | 3 |
| hsa-miR-181a-5p | MIMAT0000256 | TMEM132D | NM_133448 | 1 | 0 | 0 | 1 | 1 | 3 |
| hsa-miR-181a-5p | MIMAT0000256 | BTBD11 | NM_001018072 | 0 | 1 | 0 | 1 | 1 | 3 |
| hsa-miR-181a-5p | MIMAT0000256 | C14orf28 | NM_001017923 | 0 | 1 | 1 | 0 | 1 | 3 |
| hsa-miR-181a-5p | MIMAT0000256 | SLC24A4 | XM_005267341 | 1 | 1 | 0 | 0 | 1 | 3 |
| hsa-miR-181a-5p | MIMAT0000256 | ZNF792 | XM_005258519 | 0 | 1 | 0 | 1 | 1 | 3 |
| hsa-miR-181a-5p | MIMAT0000256 | ANKLE1 | NM_152363 | 1 | 1 | 0 | 0 | 1 | 3 |
| hsa-miR-181a-5p | MIMAT0000256 | AADACL3 | NM_001103170 | 1 | 1 | 0 | 0 | 1 | 3 |
| hsa-miR-181a-5p | MIMAT0000256 | UHMK1 | NM_175866 | 0 | 1 | 0 | 1 | 1 | 3 |
| hsa-miR-181a-5p | MIMAT0000256 | RFTN2 | XM_005246294 | 1 | 1 | 0 | 0 | 1 | 3 |
| hsa-miR-181a-5p | MIMAT0000256 | AP1S3 | NM_001039569 | 0 | 1 | 1 | 0 | 1 | 3 |
| hsa-miR-181a-5p | MIMAT0000256 | ACVR1C | NM_145259 | 1 | 1 | 0 | 0 | 1 | 3 |
| hsa-miR-181a-5p | MIMAT0000256 | CPNE4 | XM_005247106 | 1 | 1 | 0 | 0 | 1 | 3 |
| hsa-miR-181a-5p | MIMAT0000256 | TRIM71 | NM_001039111 | 0 | 1 | 1 | 0 | 1 | 3 |
| hsa-miR-181a-5p | MIMAT0000256 | NUDT16 | NM_152395 | 1 | 0 | 0 | 1 | 1 | 3 |
| hsa-miR-181a-5p | MIMAT0000256 | CPEB2 | NM_001177382 | 1 | 0 | 0 | 1 | 1 | 3 |
| hsa-miR-181a-5p | MIMAT0000256 | EMB | NM_198449 | 0 | 1 | 1 | 0 | 1 | 3 |
| hsa-miR-181a-5p | MIMAT0000256 | DNAJC21 | NM_194283 | 1 | 1 | 0 | 0 | 1 | 3 |
| hsa-miR-181a-5p | MIMAT0000256 | STXBP5 | NM_001127715 | 0 | 1 | 1 | 0 | 1 | 3 |
| hsa-miR-181a-5p | MIMAT0000256 | NCOA7 | NM_001199620 | 1 | 1 | 0 | 0 | 1 | 3 |
| hsa-miR-181a-5p | MIMAT0000256 | PACRG | XM_005266825 | 1 | 1 | 0 | 0 | 1 | 3 |
| hsa-miR-181a-5p | MIMAT0000256 | MTPN | NM_145808 | 0 | 1 | 0 | 1 | 1 | 3 |
| hsa-miR-181a-5p | MIMAT0000256 | UBXN2B | NM_001077619 | 1 | 0 | 0 | 1 | 1 | 3 |
| hsa-miR-181a-5p | MIMAT0000256 | UNC5D | XM_005273404 | 1 | 1 | 0 | 0 | 1 | 3 |
| hsa-miR-181a-5p | MIMAT0000256 | DCAF12L1 | NM_178470 | 1 | 1 | 0 | 0 | 1 | 3 |
| hsa-miR-181a-5p | MIMAT0000256 | AMER1 | NM_152424 | 0 | 1 | 0 | 1 | 1 | 3 |
| hsa-miR-181a-5p | MIMAT0000256 | ASB5 | XM_005262759 | 1 | 1 | 0 | 0 | 1 | 3 |
| hsa-miR-181a-5p | MIMAT0000256 | ZFP28 | NM_020828 | 0 | 1 | 1 | 0 | 1 | 3 |
| hsa-miR-181a-5p | MIMAT0000256 | SREK1 | NM_139168 | 1 | 0 | 0 | 1 | 1 | 3 |
| hsa-miR-181a-5p | MIMAT0000256 | CACUL1 | NM_153810 | 0 | 1 | 0 | 1 | 1 | 3 |
| hsa-miR-181a-5p | MIMAT0000256 | LDLRAD3 | XM_005252796 | 1 | 1 | 0 | 0 | 1 | 3 |
| hsa-miR-181a-5p | MIMAT0000256 | FAM76B | NM_144664 | 1 | 1 | 0 | 0 | 1 | 3 |
| hsa-miR-181a-5p | MIMAT0000256 | SPTY2D1 | NM_194285 | 1 | 1 | 0 | 0 | 1 | 3 |
| hsa-miR-181a-5p | MIMAT0000256 | SLC2A14 | XM_005253313 | 1 | 1 | 0 | 0 | 1 | 3 |
| hsa-miR-181a-5p | MIMAT0000256 | ZNF664 | NM_152437 | 1 | 1 | 0 | 0 | 1 | 3 |
| hsa-miR-181a-5p | MIMAT0000256 | PRIMA1 | NM_178013 | 0 | 1 | 0 | 1 | 1 | 3 |
| hsa-miR-181a-5p | MIMAT0000256 | PTGR2 | NM_152444 | 1 | 1 | 0 | 0 | 1 | 3 |
| hsa-miR-181a-5p | MIMAT0000256 | LRFN5 | XM_005267368 | 1 | 1 | 0 | 0 | 1 | 3 |
| hsa-miR-181a-5p | MIMAT0000256 | TTBK2 | XM_005254171 | 1 | 1 | 0 | 0 | 1 | 3 |
| hsa-miR-181a-5p | MIMAT0000256 | CMTM4 | NM_181521 | 1 | 1 | 0 | 0 | 1 | 3 |
| hsa-miR-181a-5p | MIMAT0000256 | ZNF563 | NM_145276 | 0 | 1 | 1 | 0 | 1 | 3 |
| hsa-miR-181a-5p | MIMAT0000256 | C1orf52 | NM_198077 | 1 | 1 | 0 | 0 | 1 | 3 |
| hsa-miR-181a-5p | MIMAT0000256 | TMEM56 | NM_001199679 | 0 | 1 | 0 | 1 | 1 | 3 |
| hsa-miR-181a-5p | MIMAT0000256 | RC3H1 | NM_172071 | 0 | 1 | 0 | 1 | 1 | 3 |
| hsa-miR-181a-5p | MIMAT0000256 | PDIK1L | XM_005245743 | 1 | 1 | 0 | 0 | 1 | 3 |
| hsa-miR-181a-5p | MIMAT0000256 | SMYD1 | NM_198274 | 1 | 0 | 0 | 1 | 1 | 3 |
| hsa-miR-181a-5p | MIMAT0000256 | PROM2 | NM_001165978 | 0 | 1 | 0 | 1 | 1 | 3 |
| hsa-miR-181a-5p | MIMAT0000256 | FBXO41 | NM_001080410 | 0 | 1 | 0 | 1 | 1 | 3 |
| hsa-miR-181a-5p | MIMAT0000256 | SLC38A11 | NM_001199148 | 0 | 1 | 1 | 0 | 1 | 3 |
| hsa-miR-181a-5p | MIMAT0000256 | FAM84A | NM_145175 | 0 | 1 | 0 | 1 | 1 | 3 |
| hsa-miR-181a-5p | MIMAT0000256 | MB21D2 | NM_178496 | 0 | 1 | 1 | 0 | 1 | 3 |
| hsa-miR-181a-5p | MIMAT0000256 | PPP4R2 | XM_005264714 | 1 | 1 | 0 | 0 | 1 | 3 |
| hsa-miR-181a-5p | MIMAT0000256 | DAB2IP | XM_005251721 | 0 | 1 | 0 | 1 | 1 | 3 |
| hsa-miR-181a-5p | MIMAT0000256 | TMEM167A | NM_174909 | 1 | 0 | 0 | 1 | 1 | 3 |
| hsa-miR-181a-5p | MIMAT0000256 | SRFBP1 | NM_152546 | 1 | 0 | 0 | 1 | 1 | 3 |
| hsa-miR-181a-5p | MIMAT0000256 | MBOAT1 | NM_001080480 | 1 | 1 | 0 | 0 | 1 | 3 |
| hsa-miR-181a-5p | MIMAT0000256 | RNF217 | XM_005266832 | 1 | 1 | 0 | 0 | 1 | 3 |
| hsa-miR-181a-5p | MIMAT0000256 | NKAIN2 | NM_001040214 | 0 | 1 | 1 | 0 | 1 | 3 |
| hsa-miR-181a-5p | MIMAT0000256 | C7orf60 | NM_152556 | 0 | 1 | 0 | 1 | 1 | 3 |
| hsa-miR-181a-5p | MIMAT0000256 | VKORC1L1 | NM_173517 | 1 | 1 | 0 | 0 | 1 | 3 |
| hsa-miR-181a-5p | MIMAT0000256 | CAMSAP1 | XM_005263396 | 0 | 1 | 0 | 1 | 1 | 3 |
| hsa-miR-181a-5p | MIMAT0000256 | RASEF | XM_005251731 | 1 | 1 | 0 | 0 | 1 | 3 |
| hsa-miR-181a-5p | MIMAT0000256 | TTC39B | NM_152574 | 0 | 1 | 1 | 0 | 1 | 3 |
| hsa-miR-181a-5p | MIMAT0000256 | PRUNE2 | XM_005251745 | 1 | 1 | 0 | 0 | 1 | 3 |
| hsa-miR-181a-5p | MIMAT0000256 | SLC5A8 | NM_145913 | 0 | 1 | 1 | 0 | 1 | 3 |
| hsa-miR-181a-5p | MIMAT0000256 | GPR180 | NM_180989 | 1 | 1 | 0 | 0 | 1 | 3 |
| hsa-miR-181a-5p | MIMAT0000256 | TRPV3 | NM_001258205 | 1 | 1 | 0 | 0 | 1 | 3 |
| hsa-miR-181a-5p | MIMAT0000256 | ZNF781 | NM_152605 | 0 | 1 | 1 | 0 | 1 | 3 |
| hsa-miR-181a-5p | MIMAT0000256 | DENND1B | NM_001195215 | 1 | 1 | 0 | 0 | 1 | 3 |
| hsa-miR-181a-5p | MIMAT0000256 | RASSF6 | NM_177532 | 0 | 1 | 1 | 0 | 1 | 3 |
| hsa-miR-181a-5p | MIMAT0000256 | ZNF800 | XM_005250181 | 0 | 1 | 0 | 1 | 1 | 3 |
| hsa-miR-181a-5p | MIMAT0000256 | FAM46D | NM_001170574 | 0 | 1 | 0 | 1 | 1 | 3 |
| hsa-miR-181a-5p | MIMAT0000256 | COMMD6 | NM_203497 | 1 | 1 | 0 | 0 | 1 | 3 |
| hsa-miR-181a-5p | MIMAT0000256 | KCNG3 | NM_133329 | 1 | 1 | 0 | 0 | 1 | 3 |
| hsa-miR-181a-5p | MIMAT0000256 | METTL15 | NM_152636 | 1 | 1 | 0 | 0 | 1 | 3 |
| hsa-miR-181a-5p | MIMAT0000256 | MPZL3 | XM_005271427 | 1 | 1 | 0 | 0 | 1 | 3 |
| hsa-miR-181a-5p | MIMAT0000256 | CCER1 | NM_152638 | 1 | 1 | 0 | 0 | 1 | 3 |
| hsa-miR-181a-5p | MIMAT0000256 | TUBB | XM_005248883 | 1 | 1 | 0 | 0 | 1 | 3 |
| hsa-miR-181a-5p | MIMAT0000256 | FAM219A | NM_001184940 | 0 | 1 | 0 | 1 | 1 | 3 |
| hsa-miR-181a-5p | MIMAT0000256 | VMA21 | NM_001017980 | 0 | 1 | 0 | 1 | 1 | 3 |
| hsa-miR-181a-5p | MIMAT0000256 | C3orf58 | NM_173552 | 0 | 1 | 0 | 1 | 1 | 3 |
| hsa-miR-181a-5p | MIMAT0000256 | KIAA2018 | NM_001009899 | 0 | 1 | 0 | 1 | 1 | 3 |
| hsa-miR-181a-5p | MIMAT0000256 | SLC36A1 | NM_078483 | 0 | 1 | 0 | 1 | 1 | 3 |
| hsa-miR-181a-5p | MIMAT0000256 | SAMD9L | NM_152703 | 0 | 1 | 1 | 0 | 1 | 3 |
| hsa-miR-181a-5p | MIMAT0000256 | AMER2 | XM_005266279 | 1 | 1 | 0 | 0 | 1 | 3 |
| hsa-miR-181a-5p | MIMAT0000256 | OTUD1 | NM_001145373 | 1 | 1 | 0 | 0 | 1 | 3 |
| hsa-miR-181a-5p | MIMAT0000256 | SPATA13 | XM_005266289 | 1 | 1 | 0 | 0 | 1 | 3 |
| hsa-miR-181a-5p | MIMAT0000256 | CPNE2 | NM_152727 | 0 | 1 | 1 | 0 | 1 | 3 |
| hsa-miR-181a-5p | MIMAT0000256 | C6orf223 | NM_001171992 | 1 | 1 | 0 | 0 | 1 | 3 |
| hsa-miR-181a-5p | MIMAT0000256 | C7orf41 | NM_152793 | 0 | 1 | 1 | 0 | 1 | 3 |
| hsa-miR-181a-5p | MIMAT0000256 | SEMA3D | XM_005250229 | 1 | 1 | 0 | 0 | 1 | 3 |
| hsa-miR-181a-5p | MIMAT0000256 | VGLL2 | NM_182645 | 0 | 1 | 0 | 1 | 1 | 3 |
| hsa-miR-181a-5p | MIMAT0000256 | LCLAT1 | NM_182551 | 0 | 1 | 1 | 0 | 1 | 3 |
| hsa-miR-181a-5p | MIMAT0000256 | UBN2 | XM_005250248 | 1 | 1 | 0 | 0 | 1 | 3 |
| hsa-miR-181a-5p | MIMAT0000256 | EPGN | NM_001270989 | 1 | 1 | 0 | 0 | 1 | 3 |
| hsa-miR-181a-5p | MIMAT0000256 | C3orf43 | XM_005269315 | 0 | 1 | 0 | 1 | 1 | 3 |
| hsa-miR-181a-5p | MIMAT0000256 | PAN3 | XM_005266332 | 1 | 1 | 0 | 0 | 1 | 3 |
| hsa-miR-181a-5p | MIMAT0000256 | MAMDC2 | NM_153267 | 0 | 1 | 1 | 0 | 1 | 3 |
| hsa-miR-181a-5p | MIMAT0000256 | NEGR1 | NM_173808 | 1 | 1 | 0 | 0 | 1 | 3 |
| hsa-miR-181a-5p | MIMAT0000256 | SVIP | NM_148893 | 0 | 1 | 1 | 0 | 1 | 3 |
| hsa-miR-181a-5p | MIMAT0000256 | MAGI3 | NM_152900 | 1 | 1 | 0 | 0 | 1 | 3 |
| hsa-miR-181a-5p | MIMAT0000256 | PGM2L1 | NM_173582 | 1 | 0 | 0 | 1 | 1 | 3 |
| hsa-miR-181a-5p | MIMAT0000256 | ANKRD52 | NM_173595 | 0 | 1 | 0 | 1 | 1 | 3 |
| hsa-miR-181a-5p | MIMAT0000256 | KSR2 | NM_173598 | 1 | 1 | 0 | 0 | 1 | 3 |
| hsa-miR-181a-5p | MIMAT0000256 | FAM169B | NM_182562 | 0 | 1 | 0 | 1 | 1 | 3 |
| hsa-miR-181a-5p | MIMAT0000256 | LAMA1 | NM_005559 | 1 | 1 | 0 | 0 | 1 | 3 |
| hsa-miR-181a-5p | MIMAT0000256 | ZADH2 | NM_175907 | 1 | 1 | 0 | 0 | 1 | 3 |
| hsa-miR-181a-5p | MIMAT0000256 | ZNF776 | NM_173632 | 0 | 1 | 0 | 1 | 1 | 3 |
| hsa-miR-181a-5p | MIMAT0000256 | FAM126B | NM_173822 | 1 | 1 | 0 | 0 | 1 | 3 |
| hsa-miR-181a-5p | MIMAT0000256 | ZNF660 | XM_005265090 | 1 | 1 | 0 | 0 | 1 | 3 |
| hsa-miR-181a-5p | MIMAT0000256 | TPRG1 | NM_198485 | 1 | 1 | 0 | 0 | 1 | 3 |
| hsa-miR-181a-5p | MIMAT0000256 | LOC285556 | XM_001717423 | 1 | 1 | 0 | 0 | 1 | 3 |
| hsa-miR-181a-5p | MIMAT0000256 | ZNF454 | NM_001178089 | 0 | 1 | 1 | 0 | 1 | 3 |
| hsa-miR-181a-5p | MIMAT0000256 | SCAI | NM_173690 | 1 | 1 | 0 | 0 | 1 | 3 |
| hsa-miR-181a-5p | MIMAT0000256 | YIPF6 | NM_173834 | 1 | 1 | 0 | 0 | 1 | 3 |
| hsa-miR-181a-5p | MIMAT0000256 | UBAC2 | NM_001144072 | 1 | 1 | 0 | 0 | 1 | 3 |
| hsa-miR-181a-5p | MIMAT0000256 | MCIDAS | NM_001190787 | 1 | 1 | 0 | 0 | 1 | 3 |
| hsa-miR-181a-5p | MIMAT0000256 | ECT2L | NM_001077706 | 0 | 1 | 0 | 1 | 1 | 3 |
| hsa-miR-181a-5p | MIMAT0000256 | LRRTM3 | XM_005269771 | 1 | 1 | 0 | 0 | 1 | 3 |
| hsa-miR-181a-5p | MIMAT0000256 | GEN1 | XM_005262612 | 0 | 1 | 0 | 1 | 1 | 3 |
| hsa-miR-181a-5p | MIMAT0000256 | HCN1 | NM_021072 | 0 | 1 | 1 | 0 | 1 | 3 |
| hsa-miR-181a-5p | MIMAT0000256 | FAM101B | NM_182705 | 0 | 1 | 0 | 1 | 1 | 3 |
| hsa-miR-181a-5p | MIMAT0000256 | ZNF699 | XM_005259897 | 1 | 1 | 0 | 0 | 1 | 3 |
| hsa-miR-181a-5p | MIMAT0000256 | C3orf62 | NM_198562 | 0 | 1 | 1 | 0 | 1 | 3 |
| hsa-miR-181a-5p | MIMAT0000256 | MAST4 | NM_001164664 | 0 | 1 | 0 | 1 | 1 | 3 |
| hsa-miR-181a-5p | MIMAT0000256 | CA13 | NM_198584 | 0 | 1 | 0 | 1 | 1 | 3 |
| hsa-miR-181a-5p | MIMAT0000256 | THEMIS | NM_001164685 | 1 | 1 | 0 | 0 | 1 | 3 |
| hsa-miR-181a-5p | MIMAT0000256 | C12orf68 | NM_001013635 | 0 | 1 | 0 | 1 | 1 | 3 |
| hsa-miR-181a-5p | MIMAT0000256 | TMEM233 | XM_005253881 | 1 | 1 | 0 | 0 | 1 | 3 |
| hsa-miR-181a-5p | MIMAT0000256 | C16orf87 | NM_001001436 | 0 | 1 | 1 | 0 | 1 | 3 |
| hsa-miR-181a-5p | MIMAT0000256 | ZNF470 | NM_001001668 | 0 | 1 | 0 | 1 | 1 | 3 |
| hsa-miR-181a-5p | MIMAT0000256 | C17orf102 | NM_207454 | 1 | 0 | 0 | 1 | 1 | 3 |
| hsa-miR-181a-5p | MIMAT0000256 | SAMD12 | NM_001101676 | 1 | 1 | 0 | 0 | 1 | 3 |
| hsa-miR-181a-5p | MIMAT0000256 | SMCO3 | NM_001013698 | 1 | 1 | 0 | 0 | 1 | 3 |
| hsa-miR-181a-5p | MIMAT0000256 | ZNF716 | NM_001159279 | 1 | 1 | 0 | 0 | 1 | 3 |
| hsa-miR-181a-5p | MIMAT0000256 | ATXN7L3B | NM_001136262 | 1 | 1 | 0 | 0 | 1 | 3 |
| hsa-miR-181a-5p | MIMAT0000256 | ZNF674 | NM_001039891 | 0 | 1 | 1 | 0 | 1 | 3 |
| hsa-miR-181a-5p | MIMAT0000256 | SMIM15 | NM_001048249 | 1 | 0 | 0 | 1 | 1 | 3 |
| hsa-miR-181a-5p | MIMAT0000256 | ASAH2B | XM_005270059 | 0 | 1 | 0 | 1 | 1 | 3 |
| hsa-miR-181a-5p | MIMAT0000256 | TMEM236 | NM_001098844 | 0 | 1 | 0 | 1 | 1 | 3 |
| hsa-miR-181a-5p | MIMAT0000256 | ZBTB8B | NM_001145720 | 1 | 0 | 0 | 1 | 1 | 3 |
| hsa-miR-181a-5p | MIMAT0000256 | CNTNAP3B | NM_001201380 | 1 | 1 | 0 | 0 | 1 | 3 |
| hsa-miR-181a-5p | MIMAT0000256 | ZNF286B | NM_001145045 | 1 | 1 | 0 | 0 | 1 | 3 |
| hsa-miR-181a-5p | MIMAT0000256 | FAM160A1 | XM_005263199 | 1 | 1 | 0 | 0 | 1 | 3 |
| hsa-miR-181a-5p | MIMAT0000256 | TMEM229A | NM_001136002 | 1 | 1 | 0 | 0 | 1 | 3 |
| hsa-miR-181a-5p | MIMAT0000256 | METTL12 | NM_001043229 | 1 | 1 | 0 | 0 | 1 | 3 |
| hsa-miR-181a-5p | MIMAT0000256 | TVP23A | XM_005255563 | 0 | 1 | 0 | 1 | 1 | 3 |
| hsa-miR-181a-5p | MIMAT0000256 | POM121C | XM_005250082 | 0 | 1 | 0 | 1 | 1 | 3 |
| hsa-miR-181a-5p | MIMAT0000256 | ZNF737 | NM_001159293 | 1 | 1 | 0 | 0 | 1 | 3 |
| hsa-miR-181a-5p | MIMAT0000256 | POC1B-GALNT4 | NM_001199781 | 0 | 1 | 1 | 0 | 1 | 3 |
| hsa-miR-181a-5p | MIMAT0000256 | FSBP | NM_001256141 | 1 | 1 | 0 | 0 | 1 | 3 |
| hsa-miR-181a-5p | MIMAT0000256 | ACTC1 | NM_005159 | 1 | 1 | 0 | 0 | 1 | 3 |
| hsa-miR-181a-5p | MIMAT0000256 | ACTN2 | NM_001278344 | 1 | 1 | 0 | 0 | 1 | 3 |
| hsa-miR-181a-5p | MIMAT0000256 | ACVRL1 | XM_005269235 | 0 | 1 | 0 | 1 | 1 | 3 |
| hsa-miR-181a-5p | MIMAT0000256 | ADAR | NM_001193495 | 0 | 1 | 0 | 1 | 1 | 3 |
| hsa-miR-181a-5p | MIMAT0000256 | AKT2 | NM_001626 | 0 | 1 | 0 | 1 | 1 | 3 |
| hsa-miR-181a-5p | MIMAT0000256 | ANXA6 | NM_001155 | 1 | 1 | 0 | 0 | 1 | 3 |
| hsa-miR-181a-5p | MIMAT0000256 | APBA1 | NM_001163 | 1 | 1 | 0 | 0 | 1 | 3 |
| hsa-miR-181a-5p | MIMAT0000256 | FAS | NM_000043 | 0 | 1 | 1 | 0 | 1 | 3 |
| hsa-miR-181a-5p | MIMAT0000256 | AQP2 | NM_000486 | 0 | 1 | 0 | 1 | 1 | 3 |
| hsa-miR-181a-5p | MIMAT0000256 | ARL3 | NM_004311 | 1 | 1 | 0 | 0 | 1 | 3 |
| hsa-miR-181a-5p | MIMAT0000256 | ARRB2 | NM_001257328 | 1 | 1 | 0 | 0 | 1 | 3 |
| hsa-miR-181a-5p | MIMAT0000256 | ARSD | XM_005274516 | 1 | 1 | 0 | 0 | 1 | 3 |
| hsa-miR-181a-5p | MIMAT0000256 | ATRX | XM_005262158 | 1 | 1 | 0 | 0 | 1 | 3 |
| hsa-miR-181a-5p | MIMAT0000256 | AVPR1A | XM_005269002 | 1 | 1 | 0 | 0 | 1 | 3 |
| hsa-miR-181a-5p | MIMAT0000256 | BCAT1 | XM_005253460 | 1 | 1 | 0 | 0 | 1 | 3 |
| hsa-miR-181a-5p | MIMAT0000256 | BCKDHB | XM_005248757 | 1 | 1 | 0 | 0 | 1 | 3 |
| hsa-miR-181a-5p | MIMAT0000256 | BMP8B | NM_001720 | 1 | 1 | 0 | 0 | 1 | 3 |
| hsa-miR-181a-5p | MIMAT0000256 | KLF9 | NM_001206 | 1 | 1 | 0 | 0 | 1 | 3 |
| hsa-miR-181a-5p | MIMAT0000256 | CA5A | XM_005256134 | 1 | 1 | 0 | 0 | 1 | 3 |
| hsa-miR-181a-5p | MIMAT0000256 | CAPZA1 | NM_006135 | 0 | 1 | 0 | 1 | 1 | 3 |
| hsa-miR-181a-5p | MIMAT0000256 | CASP1 | NM_001257118 | 0 | 1 | 0 | 1 | 1 | 3 |
| hsa-miR-181a-5p | MIMAT0000256 | LRBA | NM_006726 | 0 | 1 | 1 | 0 | 1 | 3 |
| hsa-miR-181a-5p | MIMAT0000256 | CDC5L | NM_001253 | 0 | 1 | 0 | 1 | 1 | 3 |
| hsa-miR-181a-5p | MIMAT0000256 | CDH13 | NM_001220488 | 1 | 1 | 0 | 0 | 1 | 3 |
| hsa-miR-181a-5p | MIMAT0000256 | CDKN3 | NM_001130851 | 0 | 1 | 1 | 0 | 1 | 3 |
| hsa-miR-181a-5p | MIMAT0000256 | CKS1B | NM_001826 | 0 | 1 | 0 | 1 | 1 | 3 |
| hsa-miR-181a-5p | MIMAT0000256 | CLCN4 | NM_001830 | 1 | 1 | 0 | 0 | 1 | 3 |
| hsa-miR-181a-5p | MIMAT0000256 | CCR3 | NM_001837 | 1 | 1 | 0 | 0 | 1 | 3 |
| hsa-miR-181a-5p | MIMAT0000256 | CNGB1 | NM_001297 | 0 | 1 | 0 | 1 | 1 | 3 |
| hsa-miR-181a-5p | MIMAT0000256 | CNGA4 | XM_005252793 | 1 | 1 | 0 | 0 | 1 | 3 |
| hsa-miR-181a-5p | MIMAT0000256 | CRKL | NM_005207 | 0 | 1 | 0 | 1 | 1 | 3 |
| hsa-miR-181a-5p | MIMAT0000256 | CYP26A1 | NM_057157 | 1 | 1 | 0 | 0 | 1 | 3 |
| hsa-miR-181a-5p | MIMAT0000256 | DARS | NM_001349 | 0 | 1 | 1 | 0 | 1 | 3 |
| hsa-miR-181a-5p | MIMAT0000256 | DBT | NM_001918 | 1 | 1 | 0 | 0 | 1 | 3 |
| hsa-miR-181a-5p | MIMAT0000256 | DIAPH1 | XM_005268384 | 0 | 1 | 0 | 1 | 1 | 3 |
| hsa-miR-181a-5p | MIMAT0000256 | DLD | NM_000108 | 1 | 1 | 0 | 0 | 1 | 3 |
| hsa-miR-181a-5p | MIMAT0000256 | TOR1A | NM_000113 | 1 | 1 | 0 | 0 | 1 | 3 |
| hsa-miR-181a-5p | MIMAT0000256 | EGR3 | XM_005273425 | 0 | 1 | 0 | 1 | 1 | 3 |
| hsa-miR-181a-5p | MIMAT0000256 | ENSA | NM_207168 | 1 | 1 | 0 | 0 | 1 | 3 |
| hsa-miR-181a-5p | MIMAT0000256 | EPHA3 | NM_005233 | 1 | 1 | 0 | 0 | 1 | 3 |
| hsa-miR-181a-5p | MIMAT0000256 | EVX1 | XM_005249639 | 1 | 1 | 0 | 0 | 1 | 3 |
| hsa-miR-181a-5p | MIMAT0000256 | F11 | NM_000128 | 0 | 1 | 1 | 0 | 1 | 3 |
| hsa-miR-181a-5p | MIMAT0000256 | FDFT1 | XM_005272374 | 1 | 1 | 0 | 0 | 1 | 3 |
| hsa-miR-181a-5p | MIMAT0000256 | FLOT2 | NM_004475 | 0 | 1 | 0 | 1 | 1 | 3 |
| hsa-miR-181a-5p | MIMAT0000256 | FPR3 | NM_002030 | 1 | 1 | 0 | 0 | 1 | 3 |
| hsa-miR-181a-5p | MIMAT0000256 | CENPI | XM_005262111 | 1 | 1 | 0 | 0 | 1 | 3 |
| hsa-miR-181a-5p | MIMAT0000256 | FUCA1 | NM_000147 | 0 | 1 | 1 | 0 | 1 | 3 |
| hsa-miR-181a-5p | MIMAT0000256 | FUT4 | NM_002033 | 0 | 1 | 0 | 1 | 1 | 3 |
| hsa-miR-181a-5p | MIMAT0000256 | FUT5 | NM_002034 | 0 | 1 | 0 | 1 | 1 | 3 |
| hsa-miR-181a-5p | MIMAT0000256 | LRRC32 | NM_001128922 | 0 | 1 | 1 | 0 | 1 | 3 |
| hsa-miR-181a-5p | MIMAT0000256 | GATA2 | NM_001145661 | 1 | 1 | 0 | 0 | 1 | 3 |
| hsa-miR-181a-5p | MIMAT0000256 | GATA6 | NM_005257 | 0 | 1 | 1 | 0 | 1 | 3 |
| hsa-miR-181a-5p | MIMAT0000256 | GNAT1 | NM_144499 | 1 | 1 | 0 | 0 | 1 | 3 |
| hsa-miR-181a-5p | MIMAT0000256 | GOT1 | NM_002079 | 0 | 1 | 0 | 1 | 1 | 3 |
| hsa-miR-181a-5p | MIMAT0000256 | GTF2E1 | XM_005247401 | 1 | 1 | 0 | 0 | 1 | 3 |
| hsa-miR-181a-5p | MIMAT0000256 | GUCA1B | NM_002098 | 1 | 1 | 0 | 0 | 1 | 3 |
| hsa-miR-181a-5p | MIMAT0000256 | HELLS | XM_005269724 | 0 | 1 | 0 | 1 | 1 | 3 |
| hsa-miR-181a-5p | MIMAT0000256 | HGD | XM_005247413 | 1 | 1 | 0 | 0 | 1 | 3 |
| hsa-miR-181a-5p | MIMAT0000256 | HLA-DQB2 | XM_005249051 | 0 | 1 | 0 | 1 | 1 | 3 |
| hsa-miR-181a-5p | MIMAT0000256 | HMBS | XM_005271531 | 0 | 1 | 0 | 1 | 1 | 3 |
| hsa-miR-181a-5p | MIMAT0000256 | HOXD3 | NM_006898 | 0 | 1 | 1 | 0 | 1 | 3 |
| hsa-miR-181a-5p | MIMAT0000256 | HRH1 | NM_001098213 | 0 | 1 | 1 | 0 | 1 | 3 |
| hsa-miR-181a-5p | MIMAT0000256 | HSD3B1 | NM_000862 | 1 | 1 | 0 | 0 | 1 | 3 |
| hsa-miR-181a-5p | MIMAT0000256 | HTR2C | NM_001256761 | 1 | 1 | 0 | 0 | 1 | 3 |
| hsa-miR-181a-5p | MIMAT0000256 | IGF1 | NM_001111283 | 0 | 1 | 0 | 1 | 1 | 3 |
| hsa-miR-181a-5p | MIMAT0000256 | IGF2 | NM_000612 | 0 | 1 | 0 | 1 | 1 | 3 |
| hsa-miR-181a-5p | MIMAT0000256 | IL6ST | NM_175767 | 1 | 1 | 0 | 0 | 1 | 3 |
| hsa-miR-181a-5p | MIMAT0000256 | IL10RA | NM_001558 | 0 | 1 | 0 | 1 | 1 | 3 |
| hsa-miR-181a-5p | MIMAT0000256 | KCNB1 | NM_004975 | 1 | 1 | 0 | 0 | 1 | 3 |
| hsa-miR-181a-5p | MIMAT0000256 | KCNJ5 | XM_005271543 | 1 | 1 | 0 | 0 | 1 | 3 |
| hsa-miR-181a-5p | MIMAT0000256 | KCNJ15 | XM_005260977 | 1 | 1 | 0 | 0 | 1 | 3 |
| hsa-miR-181a-5p | MIMAT0000256 | KCNMA1 | XM_005269799 | 1 | 1 | 0 | 0 | 1 | 3 |
| hsa-miR-181a-5p | MIMAT0000256 | KCNQ3 | NM_004519 | 1 | 1 | 0 | 0 | 1 | 3 |
| hsa-miR-181a-5p | MIMAT0000256 | KIR2DL1 | NM_014218 | 0 | 1 | 0 | 1 | 1 | 3 |
| hsa-miR-181a-5p | MIMAT0000256 | LDLR | NM_000527 | 0 | 1 | 0 | 1 | 1 | 3 |
| hsa-miR-181a-5p | MIMAT0000256 | LEPR | NM_002303 | 1 | 1 | 0 | 0 | 1 | 3 |
| hsa-miR-181a-5p | MIMAT0000256 | LMX1B | NM_001174146 | 0 | 1 | 0 | 1 | 1 | 3 |
| hsa-miR-181a-5p | MIMAT0000256 | LRPAP1 | NM_002337 | 1 | 1 | 0 | 0 | 1 | 3 |
| hsa-miR-181a-5p | MIMAT0000256 | LY75 | NM_002349 | 0 | 1 | 0 | 1 | 1 | 3 |
| hsa-miR-181a-5p | MIMAT0000256 | SMAD2 | NM_001003652 | 1 | 1 | 0 | 0 | 1 | 3 |
| hsa-miR-181a-5p | MIMAT0000256 | MAN2A2 | NM_006122 | 0 | 1 | 0 | 1 | 1 | 3 |
| hsa-miR-181a-5p | MIMAT0000256 | MBP | NM_001025092 | 1 | 1 | 0 | 0 | 1 | 3 |
| hsa-miR-181a-5p | MIMAT0000256 | MC2R | NM_000529 | 0 | 1 | 0 | 1 | 1 | 3 |
| hsa-miR-181a-5p | MIMAT0000256 | CD46 | NM_172353 | 1 | 1 | 0 | 0 | 1 | 3 |
| hsa-miR-181a-5p | MIMAT0000256 | MMP8 | NM_002424 | 1 | 1 | 0 | 0 | 1 | 3 |
| hsa-miR-181a-5p | MIMAT0000256 | MTF1 | XM_005270879 | 1 | 1 | 0 | 0 | 1 | 3 |
| hsa-miR-181a-5p | MIMAT0000256 | MYBPC1 | NM_206821 | 1 | 1 | 0 | 0 | 1 | 3 |
| hsa-miR-181a-5p | MIMAT0000256 | MYO5B | NM_001080467 | 1 | 1 | 0 | 0 | 1 | 3 |
| hsa-miR-181a-5p | MIMAT0000256 | MYO10 | NM_012334 | 1 | 1 | 0 | 0 | 1 | 3 |
| hsa-miR-181a-5p | MIMAT0000256 | NEB | XM_005246616 | 1 | 1 | 0 | 0 | 1 | 3 |
| hsa-miR-181a-5p | MIMAT0000256 | NDUFA9 | NM_005002 | 1 | 1 | 0 | 0 | 1 | 3 |
| hsa-miR-181a-5p | MIMAT0000256 | NFYC | XM_005270894 | 1 | 1 | 0 | 0 | 1 | 3 |
| hsa-miR-181a-5p | MIMAT0000256 | CNOT4 | NM_013316 | 1 | 1 | 0 | 0 | 1 | 3 |
| hsa-miR-181a-5p | MIMAT0000256 | NPC1 | NM_000271 | 0 | 1 | 0 | 1 | 1 | 3 |
| hsa-miR-181a-5p | MIMAT0000256 | NUCB2 | XM_005252945 | 1 | 1 | 0 | 0 | 1 | 3 |
| hsa-miR-181a-5p | MIMAT0000256 | PCDH7 | XM_005248163 | 1 | 1 | 0 | 0 | 1 | 3 |
| hsa-miR-181a-5p | MIMAT0000256 | PF4V1 | NM_002620 | 1 | 1 | 0 | 0 | 1 | 3 |
| hsa-miR-181a-5p | MIMAT0000256 | PHKG2 | NM_000294 | 1 | 1 | 0 | 0 | 1 | 3 |
| hsa-miR-181a-5p | MIMAT0000256 | PIN4 | NM_006223 | 1 | 1 | 0 | 0 | 1 | 3 |
| hsa-miR-181a-5p | MIMAT0000256 | PLCB3 | NM_000932 | 1 | 1 | 0 | 0 | 1 | 3 |
| hsa-miR-181a-5p | MIMAT0000256 | PLCG2 | XM_005255986 | 1 | 1 | 0 | 0 | 1 | 3 |
| hsa-miR-181a-5p | MIMAT0000256 | POLH | NM_006502 | 1 | 1 | 0 | 0 | 1 | 3 |
| hsa-miR-181a-5p | MIMAT0000256 | PPP2R5C | NM_178587 | 1 | 1 | 0 | 0 | 1 | 3 |
| hsa-miR-181a-5p | MIMAT0000256 | PRCP | NM_199418 | 1 | 1 | 0 | 0 | 1 | 3 |
| hsa-miR-181a-5p | MIMAT0000256 | PRKAA1 | XM_005248320 | 1 | 1 | 0 | 0 | 1 | 3 |
| hsa-miR-181a-5p | MIMAT0000256 | DNAJC3 | NM_006260 | 1 | 1 | 0 | 0 | 1 | 3 |
| hsa-miR-181a-5p | MIMAT0000256 | PRPSAP2 | NM_001243940 | 1 | 1 | 0 | 0 | 1 | 3 |
| hsa-miR-181a-5p | MIMAT0000256 | KLK7 | NM_005046 | 1 | 1 | 0 | 0 | 1 | 3 |
| hsa-miR-181a-5p | MIMAT0000256 | PSMA5 | NM_002790 | 1 | 1 | 0 | 0 | 1 | 3 |
| hsa-miR-181a-5p | MIMAT0000256 | PSMC2 | NM_002803 | 1 | 1 | 0 | 0 | 1 | 3 |
| hsa-miR-181a-5p | MIMAT0000256 | PTGIS | NM_000961 | 1 | 1 | 0 | 0 | 1 | 3 |
| hsa-miR-181a-5p | MIMAT0000256 | PVR | NM_006505 | 1 | 1 | 0 | 0 | 1 | 3 |
| hsa-miR-181a-5p | MIMAT0000256 | PXMP2 | NM_018663 | 1 | 1 | 0 | 0 | 1 | 3 |
| hsa-miR-181a-5p | MIMAT0000256 | RAD51B | XM_005267962 | 1 | 1 | 0 | 0 | 1 | 3 |
| hsa-miR-181a-5p | MIMAT0000256 | RDX | NM_001260496 | 1 | 1 | 0 | 0 | 1 | 3 |
| hsa-miR-181a-5p | MIMAT0000256 | RGS13 | NM_002927 | 1 | 1 | 0 | 0 | 1 | 3 |
| hsa-miR-181a-5p | MIMAT0000256 | RPL15 | XM_005265363 | 1 | 1 | 0 | 0 | 1 | 3 |
| hsa-miR-181a-5p | MIMAT0000256 | MRPL12 | NM_002949 | 1 | 1 | 0 | 0 | 1 | 3 |
| hsa-miR-181a-5p | MIMAT0000256 | RPS29 | NM_001030001 | 1 | 1 | 0 | 0 | 1 | 3 |
| hsa-miR-181a-5p | MIMAT0000256 | SORT1 | XM_005271102 | 1 | 1 | 0 | 0 | 1 | 3 |
| hsa-miR-181a-5p | MIMAT0000256 | SALL2 | XM_005267983 | 1 | 1 | 0 | 0 | 1 | 3 |
| hsa-miR-181a-5p | MIMAT0000256 | SC5D | NM_001024956 | 1 | 1 | 0 | 0 | 1 | 3 |
| hsa-miR-181a-5p | MIMAT0000256 | SCN8A | NM_014191 | 1 | 1 | 0 | 0 | 1 | 3 |
| hsa-miR-181a-5p | MIMAT0000256 | CXCL12 | NM_001178134 | 1 | 1 | 0 | 0 | 1 | 3 |
| hsa-miR-181a-5p | MIMAT0000256 | SRSF1 | NM_001078166 | 1 | 1 | 0 | 0 | 1 | 3 |
| hsa-miR-181a-5p | MIMAT0000256 | SCG5 | NM_003020 | 1 | 1 | 0 | 0 | 1 | 3 |
| hsa-miR-181a-5p | MIMAT0000256 | SHB | NM_003028 | 1 | 1 | 0 | 0 | 1 | 3 |
| hsa-miR-181a-5p | MIMAT0000256 | SHOX | NM_000451 | 1 | 1 | 0 | 0 | 1 | 3 |
| hsa-miR-181a-5p | MIMAT0000256 | SLC1A5 | XM_005259167 | 1 | 1 | 0 | 0 | 1 | 3 |
| hsa-miR-181a-5p | MIMAT0000256 | SLC7A1 | NM_003045 | 1 | 1 | 0 | 0 | 1 | 3 |
| hsa-miR-181a-5p | MIMAT0000256 | SPAG1 | NM_003114 | 1 | 1 | 0 | 0 | 1 | 3 |
| hsa-miR-181a-5p | MIMAT0000256 | SRF | NM_003131 | 1 | 1 | 0 | 0 | 1 | 3 |
| hsa-miR-181a-5p | MIMAT0000256 | SSBP1 | XM_005250048 | 1 | 1 | 0 | 0 | 1 | 3 |
| hsa-miR-181a-5p | MIMAT0000256 | CDKL5 | XM_005274584 | 1 | 1 | 0 | 0 | 1 | 3 |
| hsa-miR-181a-5p | MIMAT0000256 | SYN2 | NM_003178 | 1 | 1 | 0 | 0 | 1 | 3 |
| hsa-miR-181a-5p | MIMAT0000256 | TACR1 | NM_001058 | 1 | 1 | 0 | 0 | 1 | 3 |
| hsa-miR-181a-5p | MIMAT0000256 | LEFTY2 | NM_003240 | 1 | 1 | 0 | 0 | 1 | 3 |
| hsa-miR-181a-5p | MIMAT0000256 | TRAF3 | NM_145725 | 1 | 1 | 0 | 0 | 1 | 3 |
| hsa-miR-181a-5p | MIMAT0000256 | TRAF6 | NM_145803 | 1 | 1 | 0 | 0 | 1 | 3 |
| hsa-miR-181a-5p | MIMAT0000256 | TSHR | NM_001018036 | 1 | 1 | 0 | 0 | 1 | 3 |
| hsa-miR-181a-5p | MIMAT0000256 | TTF1 | NM_007344 | 1 | 1 | 0 | 0 | 1 | 3 |
| hsa-miR-181a-5p | MIMAT0000256 | TXK | NM_003328 | 1 | 1 | 0 | 0 | 1 | 3 |
| hsa-miR-181a-5p | MIMAT0000256 | USH2A | NM_206933 | 1 | 1 | 0 | 0 | 1 | 3 |
| hsa-miR-181a-5p | MIMAT0000256 | UVRAG | NM_003369 | 1 | 1 | 0 | 0 | 1 | 3 |
| hsa-miR-181a-5p | MIMAT0000256 | VEGFB | NM_003377 | 1 | 1 | 0 | 0 | 1 | 3 |
| hsa-miR-181a-5p | MIMAT0000256 | WNT10B | XM_005269137 | 1 | 1 | 0 | 0 | 1 | 3 |
| hsa-miR-181a-5p | MIMAT0000256 | ZNF69 | XM_005260058 | 1 | 1 | 0 | 0 | 1 | 3 |
| hsa-miR-181a-5p | MIMAT0000256 | ZNF75D | XM_005262469 | 1 | 1 | 0 | 0 | 1 | 3 |
| hsa-miR-181a-5p | MIMAT0000256 | ZNF99 | NM_001080409 | 1 | 1 | 0 | 0 | 1 | 3 |
| hsa-miR-181a-5p | MIMAT0000256 | ZNF121 | XM_005260062 | 1 | 1 | 0 | 0 | 1 | 3 |
| hsa-miR-181a-5p | MIMAT0000256 | ZNF135 | NM_001164529 | 1 | 1 | 0 | 0 | 1 | 3 |
| hsa-miR-181a-5p | MIMAT0000256 | PCGF2 | XM_005257640 | 1 | 1 | 0 | 0 | 1 | 3 |
| hsa-miR-181a-5p | MIMAT0000256 | ZBTB16 | XM_005271657 | 1 | 1 | 0 | 0 | 1 | 3 |
| hsa-miR-181a-5p | MIMAT0000256 | ZNF189 | NM_001278240 | 1 | 1 | 0 | 0 | 1 | 3 |
| hsa-miR-181a-5p | MIMAT0000256 | SEMA3B | NM_004636 | 1 | 1 | 0 | 0 | 1 | 3 |
| hsa-miR-181a-5p | MIMAT0000256 | CUBN | NM_001081 | 1 | 1 | 0 | 0 | 1 | 3 |
| hsa-miR-181a-5p | MIMAT0000256 | CUL5 | XM_005271682 | 1 | 1 | 0 | 0 | 1 | 3 |
| hsa-miR-181a-5p | MIMAT0000256 | GAN | NM_022041 | 0 | 1 | 0 | 1 | 1 | 3 |
| hsa-miR-181a-5p | MIMAT0000256 | ACOX3 | XM_005248011 | 1 | 1 | 0 | 0 | 1 | 3 |
| hsa-miR-181a-5p | MIMAT0000256 | HIST1H3E | XM_005249443 | 1 | 1 | 0 | 0 | 1 | 3 |
| hsa-miR-181a-5p | MIMAT0000256 | PPFIBP2 | XM_005253174 | 1 | 1 | 0 | 0 | 1 | 3 |
| hsa-miR-181a-5p | MIMAT0000256 | DDO | NM_003649 | 1 | 1 | 0 | 0 | 1 | 3 |
| hsa-miR-181a-5p | MIMAT0000256 | PIAS1 | XM_005254734 | 1 | 1 | 0 | 0 | 1 | 3 |
| hsa-miR-181a-5p | MIMAT0000256 | JRK | NM_003724 | 1 | 1 | 0 | 0 | 1 | 3 |
| hsa-miR-181a-5p | MIMAT0000256 | GALNT4 | NM_003774 | 0 | 1 | 1 | 0 | 1 | 3 |
| hsa-miR-181a-5p | MIMAT0000256 | CRADD | NM_003805 | 1 | 1 | 0 | 0 | 1 | 3 |
| hsa-miR-181a-5p | MIMAT0000256 | TNFSF10 | NM_001190943 | 1 | 1 | 0 | 0 | 1 | 3 |
| hsa-miR-181a-5p | MIMAT0000256 | MTMR1 | NM_003828 | 1 | 1 | 0 | 0 | 1 | 3 |
| hsa-miR-181a-5p | MIMAT0000256 | RIOK3 | NM_003831 | 0 | 1 | 0 | 1 | 1 | 3 |
| hsa-miR-181a-5p | MIMAT0000256 | TNFRSF10B | NM_003842 | 0 | 1 | 1 | 0 | 1 | 3 |
| hsa-miR-181a-5p | MIMAT0000256 | CCNK | NM_001099402 | 0 | 1 | 1 | 0 | 1 | 3 |
| hsa-miR-181a-5p | MIMAT0000256 | WISP1 | NM_003882 | 1 | 1 | 0 | 0 | 1 | 3 |
| hsa-miR-181a-5p | MIMAT0000256 | TSC22D1 | XM_005266583 | 1 | 1 | 0 | 0 | 1 | 3 |
| hsa-miR-181a-5p | MIMAT0000256 | FUBP1 | XM_005271310 | 1 | 1 | 0 | 0 | 1 | 3 |
| hsa-miR-181a-5p | MIMAT0000256 | HERC3 | XM_005263331 | 1 | 1 | 0 | 0 | 1 | 3 |
| hsa-miR-181a-5p | MIMAT0000256 | P4HA2 | NM_004199 | 1 | 1 | 0 | 0 | 1 | 3 |
| hsa-miR-181a-5p | MIMAT0000256 | KALRN | NM_003947 | 0 | 1 | 1 | 0 | 1 | 3 |
| hsa-miR-181a-5p | MIMAT0000256 | TAF1A | NM_005681 | 0 | 1 | 1 | 0 | 1 | 3 |
| hsa-miR-181a-5p | MIMAT0000256 | CLDN10 | NM_182848 | 0 | 1 | 0 | 1 | 1 | 3 |
| hsa-miR-181a-5p | MIMAT0000256 | CLDN8 | NM_199328 | 0 | 1 | 1 | 0 | 1 | 3 |
| hsa-miR-181a-5p | MIMAT0000256 | DIRAS3 | NM_004675 | 0 | 1 | 1 | 0 | 1 | 3 |
| hsa-miR-181a-5p | MIMAT0000256 | SLC16A7 | NM_001270623 | 1 | 1 | 0 | 0 | 1 | 3 |
| hsa-miR-181a-5p | MIMAT0000256 | TGFBRAP1 | NM_001142621 | 0 | 1 | 1 | 0 | 1 | 3 |
| hsa-miR-181a-5p | MIMAT0000256 | GRAP2 | XM_005261836 | 1 | 1 | 0 | 0 | 1 | 3 |
| hsa-miR-181a-5p | MIMAT0000256 | NCR2 | NM_001199509 | 1 | 1 | 0 | 0 | 1 | 3 |
| hsa-miR-181a-5p | MIMAT0000256 | MED20 | NM_004275 | 0 | 1 | 1 | 0 | 1 | 3 |
| hsa-miR-181a-5p | MIMAT0000256 | TBPL1 | NM_004865 | 0 | 1 | 1 | 0 | 1 | 3 |
| hsa-miR-181a-5p | MIMAT0000256 | H2AFY | XM_005272134 | 1 | 1 | 0 | 0 | 1 | 3 |
| hsa-miR-181a-5p | MIMAT0000256 | WTAP | NM_004906 | 1 | 1 | 0 | 0 | 1 | 3 |
| hsa-miR-181a-5p | MIMAT0000256 | RNF14 | NM_004290 | 1 | 1 | 0 | 0 | 1 | 3 |
| hsa-miR-181a-5p | MIMAT0000256 | ABCG1 | XM_005261209 | 1 | 1 | 0 | 0 | 1 | 3 |
| hsa-miR-181a-5p | MIMAT0000256 | SEC24C | NM_198597 | 0 | 1 | 1 | 0 | 1 | 3 |
| hsa-miR-181a-5p | MIMAT0000256 | CEP135 | NM_025009 | 1 | 1 | 0 | 0 | 1 | 3 |
| hsa-miR-181a-5p | MIMAT0000256 | IPO13 | XM_003846624 | 1 | 1 | 0 | 0 | 1 | 3 |
| hsa-miR-181a-5p | MIMAT0000256 | FAM53B | XM_005270300 | 0 | 1 | 0 | 1 | 1 | 3 |
| hsa-miR-181a-5p | MIMAT0000256 | GREB1 | XM_005246198 | 1 | 1 | 0 | 0 | 1 | 3 |
| hsa-miR-181a-5p | MIMAT0000256 | NUP93 | XM_005256263 | 1 | 1 | 0 | 0 | 1 | 3 |
| hsa-miR-181a-5p | MIMAT0000256 | POM121 | NM_172020 | 0 | 1 | 1 | 0 | 1 | 3 |
| hsa-miR-181a-5p | MIMAT0000256 | SLC35E2 | NM_182838 | 1 | 1 | 0 | 0 | 1 | 3 |
| hsa-miR-181a-5p | MIMAT0000256 | ZBTB40 | NM_001083621 | 0 | 1 | 0 | 1 | 1 | 3 |
| hsa-miR-181a-5p | MIMAT0000256 | EXOG | XM_005265635 | 1 | 1 | 0 | 0 | 1 | 3 |
| hsa-miR-181a-5p | MIMAT0000256 | TNFSF15 | NM_005118 | 1 | 1 | 0 | 0 | 1 | 3 |
| hsa-miR-181a-5p | MIMAT0000256 | SH2B3 | NM_005475 | 0 | 1 | 1 | 0 | 1 | 3 |
| hsa-miR-181a-5p | MIMAT0000256 | GPC6 | NM_005708 | 1 | 1 | 0 | 0 | 1 | 3 |
| hsa-miR-181a-5p | MIMAT0000256 | HHLA1 | NM_001145095 | 0 | 1 | 1 | 0 | 1 | 3 |
| hsa-miR-181a-5p | MIMAT0000256 | AASS | NM_005763 | 1 | 1 | 0 | 0 | 1 | 3 |
| hsa-miR-181a-5p | MIMAT0000256 | RNF41 | NM_194358 | 1 | 1 | 0 | 0 | 1 | 3 |
| hsa-miR-181a-5p | MIMAT0000256 | BET1 | NM_005868 | 1 | 1 | 0 | 0 | 1 | 3 |
| hsa-miR-181a-5p | MIMAT0000256 | B3GALT5 | XM_005260912 | 0 | 1 | 0 | 1 | 1 | 3 |
| hsa-miR-181a-5p | MIMAT0000256 | CRTAP | NM_006371 | 1 | 1 | 0 | 0 | 1 | 3 |
| hsa-miR-181a-5p | MIMAT0000256 | NCOA2 | NM_006540 | 0 | 1 | 1 | 0 | 1 | 3 |
| hsa-miR-181a-5p | MIMAT0000256 | HYOU1 | NM_006389 | 0 | 1 | 0 | 1 | 1 | 3 |
| hsa-miR-181a-5p | MIMAT0000256 | ARPC1A | NM_001190996 | 0 | 1 | 1 | 0 | 1 | 3 |
| hsa-miR-181a-5p | MIMAT0000256 | AGPAT1 | XM_005248805 | 0 | 1 | 0 | 1 | 1 | 3 |
| hsa-miR-181a-5p | MIMAT0000256 | TCFL5 | XM_005260186 | 1 | 1 | 0 | 0 | 1 | 3 |
| hsa-miR-181a-5p | MIMAT0000256 | CHL1 | NM_006614 | 1 | 1 | 0 | 0 | 1 | 3 |
| hsa-miR-181a-5p | MIMAT0000256 | GJB6 | NM_001110219 | 0 | 1 | 0 | 1 | 1 | 3 |
| hsa-miR-181a-5p | MIMAT0000256 | CFHR3 | XM_005244849 | 1 | 1 | 0 | 0 | 1 | 3 |
| hsa-miR-181a-5p | MIMAT0000256 | IFI44L | NM_006820 | 1 | 1 | 0 | 0 | 1 | 3 |
| hsa-miR-181a-5p | MIMAT0000256 | MID2 | NM_012216 | 0 | 1 | 1 | 0 | 1 | 3 |
| hsa-miR-181a-5p | MIMAT0000256 | DIDO1 | NM_080796 | 1 | 1 | 0 | 0 | 1 | 3 |
| hsa-miR-181a-5p | MIMAT0000256 | ADAMTS8 | NM_007037 | 0 | 1 | 0 | 1 | 1 | 3 |
| hsa-miR-181a-5p | MIMAT0000256 | IKZF3 | NM_012481 | 1 | 1 | 0 | 0 | 1 | 3 |
| hsa-miR-181a-5p | MIMAT0000256 | ZNF652 | NM_001145365 | 1 | 1 | 0 | 0 | 1 | 3 |
| hsa-miR-181a-5p | MIMAT0000256 | VASH1 | NM_014909 | 1 | 1 | 0 | 0 | 1 | 3 |
| hsa-miR-181a-5p | MIMAT0000256 | RUFY3 | NM_001130709 | 1 | 1 | 0 | 0 | 1 | 3 |
| hsa-miR-181a-5p | MIMAT0000256 | TRAK1 | NM_001042646 | 0 | 1 | 1 | 0 | 1 | 3 |
| hsa-miR-181a-5p | MIMAT0000256 | NCBP2 | XM_005269313 | 1 | 1 | 0 | 0 | 1 | 3 |
| hsa-miR-181a-5p | MIMAT0000256 | TPX2 | XM_005260339 | 0 | 1 | 0 | 1 | 1 | 3 |
| hsa-miR-181a-5p | MIMAT0000256 | MYH15 | NM_014981 | 0 | 1 | 1 | 0 | 1 | 3 |
| hsa-miR-181a-5p | MIMAT0000256 | CNOT1 | NM_016284 | 1 | 1 | 0 | 0 | 1 | 3 |
| hsa-miR-181a-5p | MIMAT0000256 | GGA2 | NM_015044 | 0 | 1 | 0 | 1 | 1 | 3 |
| hsa-miR-181a-5p | MIMAT0000256 | KDM4C | NM_001146694 | 0 | 1 | 1 | 0 | 1 | 3 |
| hsa-miR-181a-5p | MIMAT0000256 | RAP1GAP2 | XM_005256544 | 1 | 1 | 0 | 0 | 1 | 3 |
| hsa-miR-181a-5p | MIMAT0000256 | SPG20 | NM_015087 | 1 | 1 | 0 | 0 | 1 | 3 |
| hsa-miR-181a-5p | MIMAT0000256 | CLCC1 | NM_001048210 | 1 | 1 | 0 | 0 | 1 | 3 |
| hsa-miR-181a-5p | MIMAT0000256 | SNX13 | XM_005249672 | 1 | 1 | 0 | 0 | 1 | 3 |
| hsa-miR-181a-5p | MIMAT0000256 | 8-Sep | NM_001098812 | 0 | 1 | 1 | 0 | 1 | 3 |
| hsa-miR-181a-5p | MIMAT0000256 | SOGA2 | XM_005258101 | 1 | 1 | 0 | 0 | 1 | 3 |
| hsa-miR-181a-5p | MIMAT0000256 | CLUH | XM_005256565 | 0 | 1 | 0 | 1 | 1 | 3 |
| hsa-miR-181a-5p | MIMAT0000256 | DNAJC13 | NM_015268 | 0 | 1 | 1 | 0 | 1 | 3 |
| hsa-miR-181a-5p | MIMAT0000256 | KHNYN | XM_005267476 | 1 | 1 | 0 | 0 | 1 | 3 |
| hsa-miR-181a-5p | MIMAT0000256 | USP24 | XM_005270689 | 1 | 1 | 0 | 0 | 1 | 3 |
| hsa-miR-181a-5p | MIMAT0000256 | NCAPH | NM_015341 | 1 | 1 | 0 | 0 | 1 | 3 |
| hsa-miR-181a-5p | MIMAT0000256 | ABCA5 | NM_018672 | 0 | 1 | 0 | 1 | 1 | 3 |
| hsa-miR-181a-5p | MIMAT0000256 | PHF3 | NM_015153 | 0 | 1 | 1 | 0 | 1 | 3 |
| hsa-miR-181a-5p | MIMAT0000256 | RUSC1 | NM_001105203 | 1 | 1 | 0 | 0 | 1 | 3 |
| hsa-miR-181a-5p | MIMAT0000256 | EID1 | NM_014335 | 0 | 1 | 0 | 1 | 1 | 3 |
| hsa-miR-181a-5p | MIMAT0000256 | PANX1 | XM_005273862 | 1 | 1 | 0 | 0 | 1 | 3 |
| hsa-miR-181a-5p | MIMAT0000256 | TBC1D22A | NM_014346 | 1 | 1 | 0 | 0 | 1 | 3 |
| hsa-miR-181a-5p | MIMAT0000256 | USP49 | XM_005248991 | 0 | 1 | 0 | 1 | 1 | 3 |
| hsa-miR-181a-5p | MIMAT0000256 | ABI3BP | NM_015429 | 0 | 1 | 1 | 0 | 1 | 3 |
| hsa-miR-181a-5p | MIMAT0000256 | METTL21B | NM_206914 | 0 | 1 | 0 | 1 | 1 | 3 |
| hsa-miR-181a-5p | MIMAT0000256 | THUMPD3 | XM_005265022 | 0 | 1 | 0 | 1 | 1 | 3 |
| hsa-miR-181a-5p | MIMAT0000256 | NSL1 | NM_001042549 | 1 | 1 | 0 | 0 | 1 | 3 |
| hsa-miR-181a-5p | MIMAT0000256 | SAMHD1 | NM_015474 | 0 | 1 | 1 | 0 | 1 | 3 |
| hsa-miR-181a-5p | MIMAT0000256 | TENM4 | XM_005273900 | 0 | 1 | 0 | 1 | 1 | 3 |
| hsa-miR-181a-5p | MIMAT0000256 | ACOT11 | XM_005270730 | 0 | 1 | 0 | 1 | 1 | 3 |
| hsa-miR-181a-5p | MIMAT0000256 | HERC4 | NM_001278187 | 1 | 1 | 0 | 0 | 1 | 3 |
| hsa-miR-181a-5p | MIMAT0000256 | ZBTB20 | XM_005247336 | 1 | 1 | 0 | 0 | 1 | 3 |
| hsa-miR-181a-5p | MIMAT0000256 | FBXW2 | NM_012164 | 1 | 1 | 0 | 0 | 1 | 3 |
| hsa-miR-181a-5p | MIMAT0000256 | PTPN22 | NM_015967 | 0 | 1 | 1 | 0 | 1 | 3 |
| hsa-miR-181a-5p | MIMAT0000256 | FBXO22 | NM_147188 | 1 | 1 | 0 | 0 | 1 | 3 |
| hsa-miR-181a-5p | MIMAT0000256 | VCX | NM_013452 | 0 | 1 | 1 | 0 | 1 | 3 |
| hsa-miR-181a-5p | MIMAT0000256 | OR1J2 | XM_005251920 | 1 | 1 | 0 | 0 | 1 | 3 |
| hsa-miR-181a-5p | MIMAT0000256 | ATP5S | XM_005267538 | 0 | 1 | 0 | 1 | 1 | 3 |
| hsa-miR-181a-5p | MIMAT0000256 | NAAA | NM_014435 | 0 | 1 | 1 | 0 | 1 | 3 |
| hsa-miR-181a-5p | MIMAT0000256 | SALL3 | XM_005266680 | 1 | 1 | 0 | 0 | 1 | 3 |
| hsa-miR-181a-5p | MIMAT0000256 | DISC1 | NM_001164537 | 0 | 1 | 0 | 1 | 1 | 3 |
| hsa-miR-181a-5p | MIMAT0000256 | OXGR1 | NM_080818 | 0 | 1 | 1 | 0 | 1 | 3 |
| hsa-miR-181a-5p | MIMAT0000256 | GPR78 | NM_080819 | 1 | 1 | 0 | 0 | 1 | 3 |
| hsa-miR-181a-5p | MIMAT0000256 | COQ2 | NM_015697 | 0 | 1 | 1 | 0 | 1 | 3 |
| hsa-miR-181a-5p | MIMAT0000256 | MRPS18B | XM_005249030 | 1 | 1 | 0 | 0 | 1 | 3 |
| hsa-miR-181a-5p | MIMAT0000256 | TMEM14A | NM_014051 | 0 | 1 | 1 | 0 | 1 | 3 |
| hsa-miR-181a-5p | MIMAT0000256 | DROSHA | NM_013235 | 0 | 1 | 0 | 1 | 1 | 3 |
| hsa-miR-181a-5p | MIMAT0000256 | CTNNA3 | XM_005269717 | 1 | 1 | 0 | 0 | 1 | 3 |
| hsa-miR-181a-5p | MIMAT0000256 | HILPDA | NM_013332 | 0 | 1 | 0 | 1 | 1 | 3 |
| hsa-miR-181a-5p | MIMAT0000256 | PURG | XM_005273484 | 1 | 1 | 0 | 0 | 1 | 3 |
| hsa-miR-181a-5p | MIMAT0000256 | SCHIP1 | NM_014575 | 1 | 1 | 0 | 0 | 1 | 3 |
| hsa-miR-181a-5p | MIMAT0000256 | BAZ2B | NM_013450 | 0 | 1 | 1 | 0 | 1 | 3 |
| hsa-miR-181a-5p | MIMAT0000256 | SOCS7 | NM_014598 | 1 | 1 | 0 | 0 | 1 | 3 |
| hsa-miR-181a-5p | MIMAT0000256 | EXOSC3 | NM_016042 | 0 | 1 | 0 | 1 | 1 | 3 |
| hsa-miR-181a-5p | MIMAT0000256 | ABHD5 | NM_016006 | 1 | 1 | 0 | 0 | 1 | 3 |
| hsa-miR-181a-5p | MIMAT0000256 | TFB1M | XM_005267005 | 1 | 1 | 0 | 0 | 1 | 3 |
| hsa-miR-181a-5p | MIMAT0000256 | PDZD11 | NM_016484 | 1 | 1 | 0 | 0 | 1 | 3 |
| hsa-miR-181a-5p | MIMAT0000256 | DNAJC27 | NM_016544 | 0 | 1 | 0 | 1 | 1 | 3 |
| hsa-miR-181a-5p | MIMAT0000256 | PLAC8 | NM_001130716 | 1 | 1 | 0 | 0 | 1 | 3 |
| hsa-miR-181a-5p | MIMAT0000256 | CDC40 | NM_015891 | 0 | 1 | 1 | 0 | 1 | 3 |
| hsa-miR-181a-5p | MIMAT0000256 | VCX3A | NM_016379 | 0 | 1 | 1 | 0 | 1 | 3 |
| hsa-miR-181a-5p | MIMAT0000256 | GTSE1 | XM_005261627 | 1 | 1 | 0 | 0 | 1 | 3 |
| hsa-miR-181a-5p | MIMAT0000256 | UPB1 | XM_005261633 | 1 | 1 | 0 | 0 | 1 | 3 |
| hsa-miR-181a-5p | MIMAT0000256 | CSNK1G1 | NM_022048 | 0 | 1 | 0 | 1 | 1 | 3 |
| hsa-miR-181a-5p | MIMAT0000256 | RIN2 | NM_018993 | 0 | 1 | 1 | 0 | 1 | 3 |
| hsa-miR-181a-5p | MIMAT0000256 | MRPS21 | NM_018997 | 1 | 1 | 0 | 0 | 1 | 3 |
[truncated: 60,719 more chars]
